# Diverse *N*‐Heterocyclic Ring Systems via Aza‐Heck Cyclizations of *N*‐(Pentafluorobenzoyloxy)sulfonamides

**DOI:** 10.1002/anie.201605152

**Published:** 2016-07-27

**Authors:** Ian R. Hazelden, Xiaofeng Ma, Thomas Langer, John F. Bower

**Affiliations:** ^1^ School of Chemistry University of Bristol Bristol BS8 1TS UK; ^2^ Pharmaceutical Technology & Development, AstraZeneca Charter Way Macclesfield SK10 2NA UK

**Keywords:** aza-Heck reaction, cascade reactions, N-heterocycles, palladium

## Abstract

Aza‐Heck cyclizations initiated by oxidative addition of Pd^0^‐catalysts into the N−O bond of *N*‐(pentafluoro‐benzoyloxy)sulfonamides are described. These studies, which encompass only the second class of aza‐Heck reaction developed to date, provide direct access to diverse *N*‐heterocyclic ring systems.

There has been a resurgence of interest in the development of processes based on the Mizoroki–Heck reaction.^1^ Notable contributions include boryl‐Heck alkene functionalizations^2^ and remote redox relay Heck C−C bond formations.^3^ Our focus has been on the development of aza‐variants of the Heck reaction, because of the importance of *N*‐containing ring systems in drug discovery.^4^, ^5^, ^6^, ^7^ Within this context, the Narasaka process,^4^ which involves the Pd‐catalyzed cyclization of O‐pentafluorobenzoyl ketoxime esters with alkenes, is unique in harnessing key steps that are analogous to the conventional Heck reaction: 1) an unusual oxidative addition into the N−O bond of **1** to afford cationic imino‐Pd intermediate **2**;^7^, ^8^ 2) C−N bond forming alkene migratory insertion;^9^ and 3) β‐hydride elimination (Scheme 1 A). Imino‐Pd^II^ intermediates **2** can also be exploited more widely in redox neutral processes, such as diverse alkene 1,2‐carboaminations,^8^ aryl C−H aminations,7a alkene aziridinations,^10^ alkene 1,2‐iodoaminations,^11^ aryne aminofunctionalizations,^12^ and C−C bond activations.^13^

**Scheme 1:**
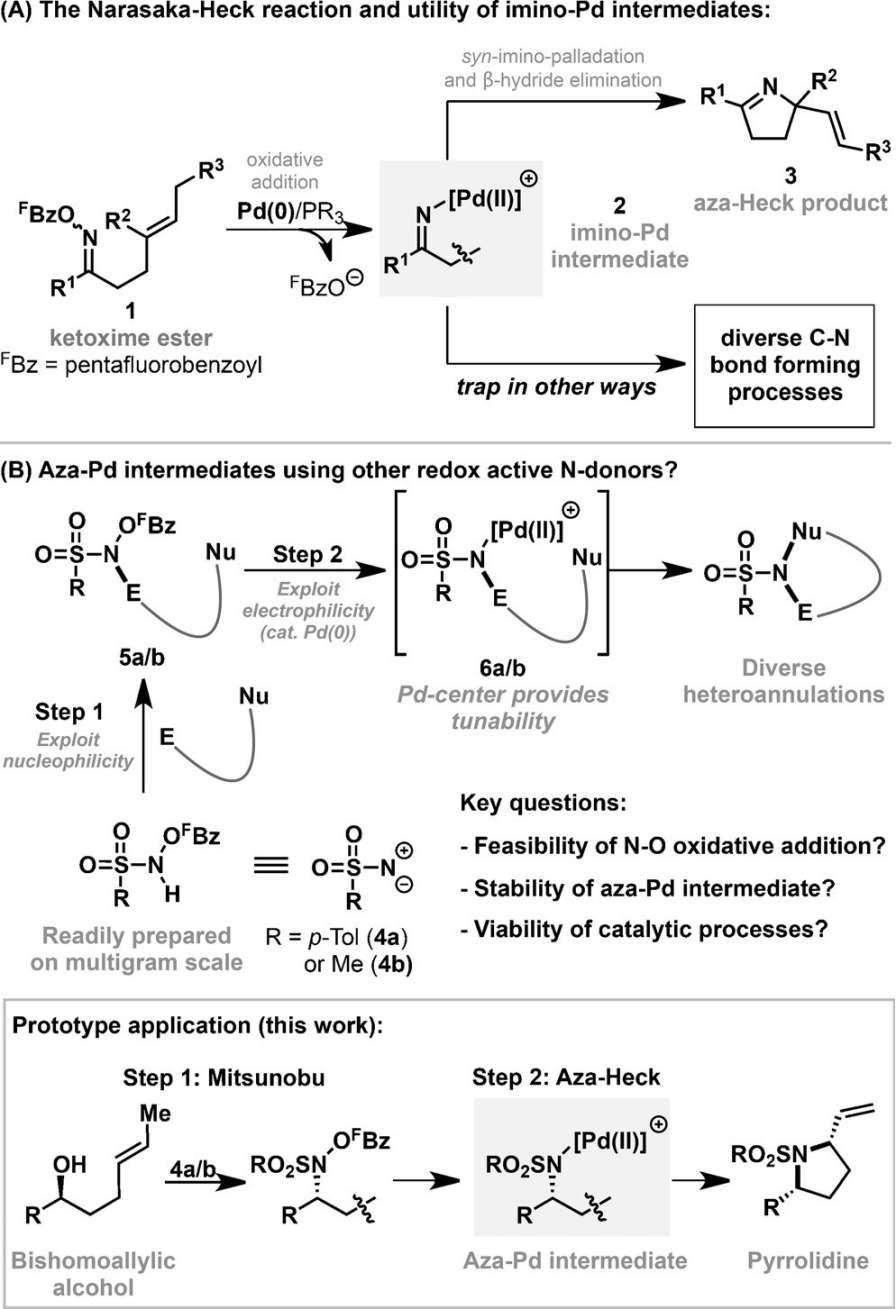
Aza‐Pd intermediates via redox‐active N‐donors.

Efforts to expand the range of redox active donors available for accessing aza‐Pd^II^ intermediates led us to consider whether activated hydroxysulfonamide derivatives might be viable (Scheme 1 B).^14^ In this approach, *N*‐(pentafluorobenzoyloxy)sulfonamides **4 a**/**b**, which we have found easy to prepare on gram scale,^15^ act as a formal nitrene equivalent, but with key distinguishing aspects. First, as with nitrenes, **4 a**/**b** function as both a nucleophile and electrophile, but, importantly, these features are decoupled, such that their unveiling can be orchestrated in a controlled manner. Second, nucleophilic modification of **4 a**/**b** can be achieved under stereospecific Mitsunobu conditions and this allows readily available enantiopure secondary alcohols to be exploited in synthetic sequences.^16^ Third, and most importantly, **5 a**/**b** do not function as an electrophile by direct reaction at nitrogen, with this reactivity facet instead controlled by the Pd‐center of aza‐Pd^II^ species **6 a**/**b**. Consequently, alkylated derivatives **5 a**/**b** can, in principle, be adapted to asymmetric cyclizations^17^ and cascade sequences,^18^ as well as other processes typical of Pd‐catalysis. Herein, we delineate preliminary studies towards this broad goal by reporting what is, to the best of our knowledge, only the second class of aza‐Heck reaction developed to date (Scheme 1 B, box).^19^ The process provides high versatility for the synthesis of complex *N*‐heterocyclic ring systems^20^ and can be integrated into cascade sequences to provide alkene 1,2‐carboamination products. This validates the broader *N*‐heteroannulation strategy outlined in Scheme 1 B.

Initial studies focused on aza‐Heck cyclization of monosubstituted alkene **7 a**, which was prepared in 70 % yield by Mitsunobu alkylation of **4 a** with pent‐4‐enol (Scheme 2).^15^ Under conditions related to those previously optimized for aza‐Heck cyclizations of oxime esters, where P(3,5‐(CF_3_)_2_C_6_H_3_)_3_ was identified as a privileged ligand,^5^ ketone **8 a**′ was isolated in 82 % yield. ^1^H NMR analysis of crude reaction mixtures indicated that **8 a′** forms via hydrolysis of initial aza‐Heck product **8 a**.

**Scheme 2:**
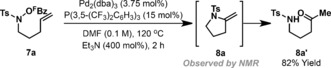
A feasibility experiment.

Cyclization of **7 a** was considered relatively easy as both the N−O bond and alkene are sterically accessible. To integrate the new process into synthetically attractive settings we sought substrates where β‐hydride elimination to form hydrolytically sensitive enamides was not possible. Accordingly we focused on cyclic alkene **7 ba**, which was expected to deliver bicyclic system **8 b**, due to the presumed mechanistic constraints of *syn*‐amino palladation and *syn*‐β‐hydride elimination (Table 1). In the event, this system was challenging, with initial attempts generating **8 b** in only 34 % yield as a 3:1 mixture with regioisomer *iso*‐**8 b** (entry 1); this likely arises via Pd‐hydride mediated isomerization of **8 b**. Inefficiencies were attributed to competing protodepalladation and β‐hydride elimination at the stage of the aza‐Pd^II^ intermediate; this latter pathway led to the isolation of the corresponding aldehyde.^21^ Optimization was undertaken focusing on activating group, solvent, and ligand. O‐Trifluoroacetyl activated variant **7 bc** offered marginal efficiency gains (entry 3), whereas an O‐Ms activated system **7 bb** was less effective. Less dissociating activating groups, such as O‐Bz, were completely ineffective (see below). Fortunately, it was found that solvent effects were pronounced, with *n*‐BuCN, MeCN, and THF all promoting cyclization of **7 ba** to target **8 b** in useful yield (entries 4,6,7). The most efficient method used a mixed‐solvent system and sub‐stoichiometric quantities of Et_3_N (see below; entry 5). The process is highly sensitive to the nature of the phosphine ligand, and, from an exhaustive screen of commercial variants, the only other systems found to provide greater than 20 % yield were PPh_3_, dppp, and P(4‐(CF_3_)C_6_H_4_)_3_.

**Table 1:** Optimization of a demanding cyclization. 
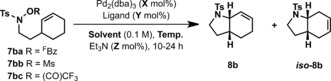

Entry	R	Ligand	Solvent	*X*	*Y*	*Z*	*T* [°C]	Yield [%]^[a]^
1	^F^Bz	P(3,5‐(CF_3_)_2_C_6_H_3_)_3_	DMF	4	15	400	80	34 (3:1)
2	Ms	P(3,5‐(CF_3_)_2_C_6_H_3_)_3_	DMF	4	15	400	80	4 (n.d.)
3	(CO)CF_3_	P(3,5‐(CF_3_)_2_C_6_H_3_)_3_	DMF	5	20	200	80	46 (3:1)
4	^F^Bz	P(3,5‐(CF_3_)_2_C_6_H_3_)_3_	*n*‐BuCN	2.5	12.5	50	110	80 (17:1)
5	^F^Bz	P(3,5‐(CF_3_)_2_C_6_H_3_)_3_	*n*‐BuCN/DMF (6:1)	2.5	12.5	50	110	91 (12:1)
6	^F^Bz	P(3,5‐(CF_3_)_2_C_6_H_3_)_3_	MeCN	5	20	100	100	76 (3:1)
7	^F^Bz	P(3,5‐(CF_3_)_2_C_6_H_3_)_3_	THF	5	20	100	100	62 (1:0)
8	^F^Bz	P(3,5‐(CF_3_)_2_C_6_H_3_)_3_	*n*‐BuCN	5	20	100	110	77 (24:1)
9	^F^Bz	PPh_3_	*n*‐BuCN	5	20	100	110	51 (13:1)
10	^F^Bz	dppp	*n*‐BuCN	5	10	100	110	24 (12:1)
11	^F^Bz	P(4‐(CF_3_)C_6_H_4_)_3_	*n*‐BuCN	5	20	100	110	33 (3:1)

[a] In situ yield; **8 b**:*iso*‐**8 b** ratio is given in parentheses.

The scope of the aza‐Heck process is outlined in Table 2, with fine tuning of reaction solvent required on a case‐by‐case basis. Cyclization of **7 c**, which involves a cyclopentene, generated bicyclic system **8 c** in high yield and as a single diastereomer. Efficient cyclizations were observed for processes involving 1,2‐disubstituted alkenes. For example, **7 d** delivered **8 d** in 81 % yield and with complete selectivity over the corresponding enamide (cf. **7 a** to **8 a**). 1,1‐Disubstituted alkenes are also tolerated, albeit with greater variation in efficiency. Cyclization of **7 f** generated the challenging tetrasubstituted stereocenter of pyrrolidine **8 f** in 80 % yield. More sterically demanding systems **7 g** and **7 h** were less effective, but still delivered targets **8 g** and **8 h** in workable yields. Systems with substitution on the alkene tether can provide diastereoselective processes. For example, **7 k** generated *cis*‐2,5‐disubstituted pyrrolidine **8 k** in 58 % yield and more than 10:1 d.r; for this process, an *N*‐tosyl protecting group was less effective.^15^ Similar efficiencies were observed for **7 j**, **7 l**, and **7 m**, with the latter affording complex 2,2,5‐trisubstituted pyrrolidine **8 m** in high diastereoselectivity. Electron‐deficient alkenes also participate: cyclization of acrylate **7 n** provided **8 n** in 78 % yield, thereby validating a novel entry to versatile alkylidene pyrrolidines.

**Table 2:** Scope of the aza‐Heck process. 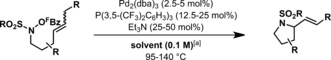

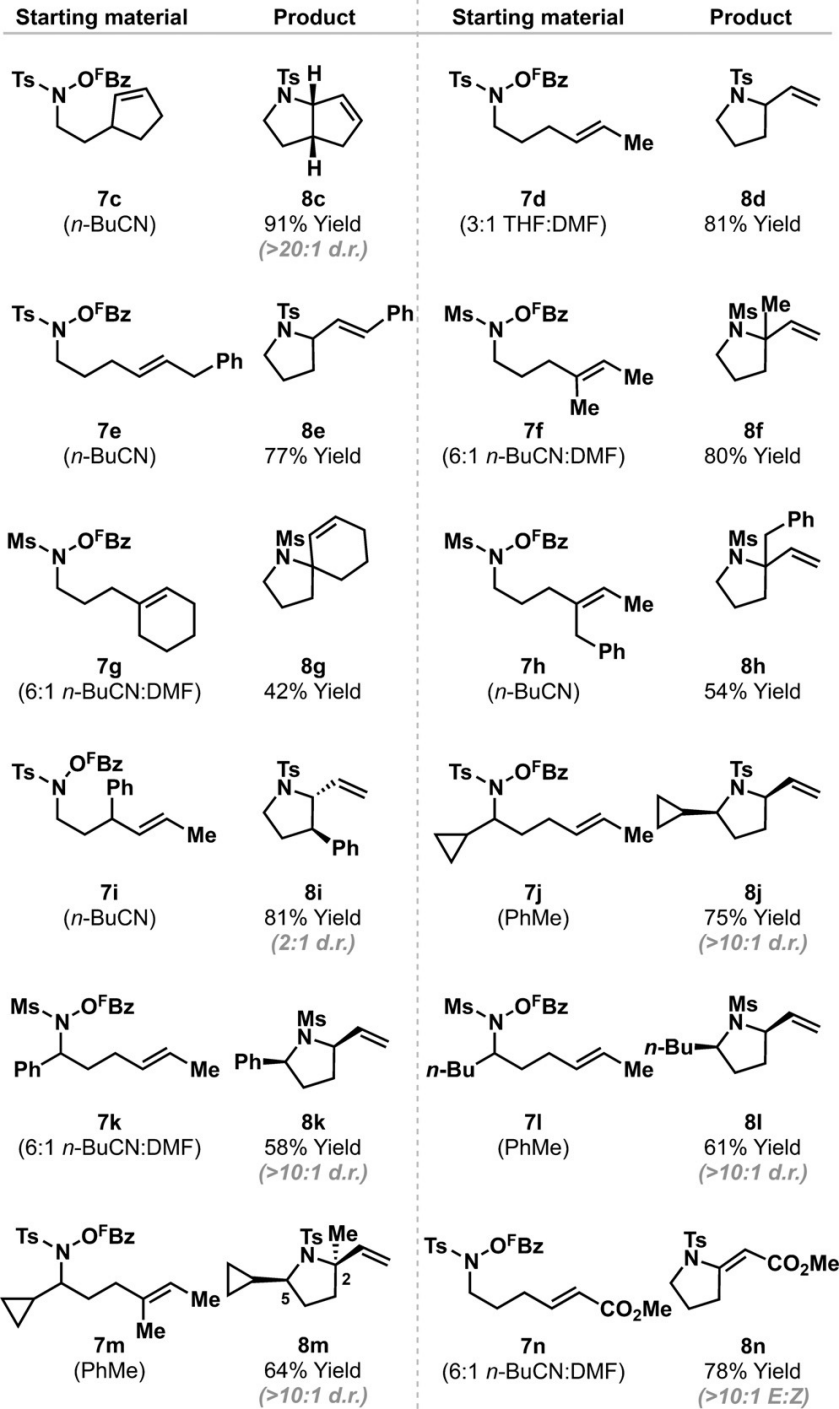

[a] Reaction solvent is specified in parentheses under each starting material. Full details are given in the Supporting Information.

The chemistry can be used to provide challenging bridged ring systems common to many alkaloid targets (Scheme 3). For example, cyclization of **7 o**, which involves a cycloheptene constructed by RCM,^15^ provided tropane **8 o** in 60 % yield; this is the core structure of multiple natural products including cocaine.^22^ Alternatively, cyclization of **7 p** generated regioisomeric 6‐azabicyclo[3.2.1]octene scaffold **8 p** in 76 % yield.^23^ The structures of **8 o** and **8 p** were confirmed by X‐ray diffraction.^15^

**Scheme 3:**
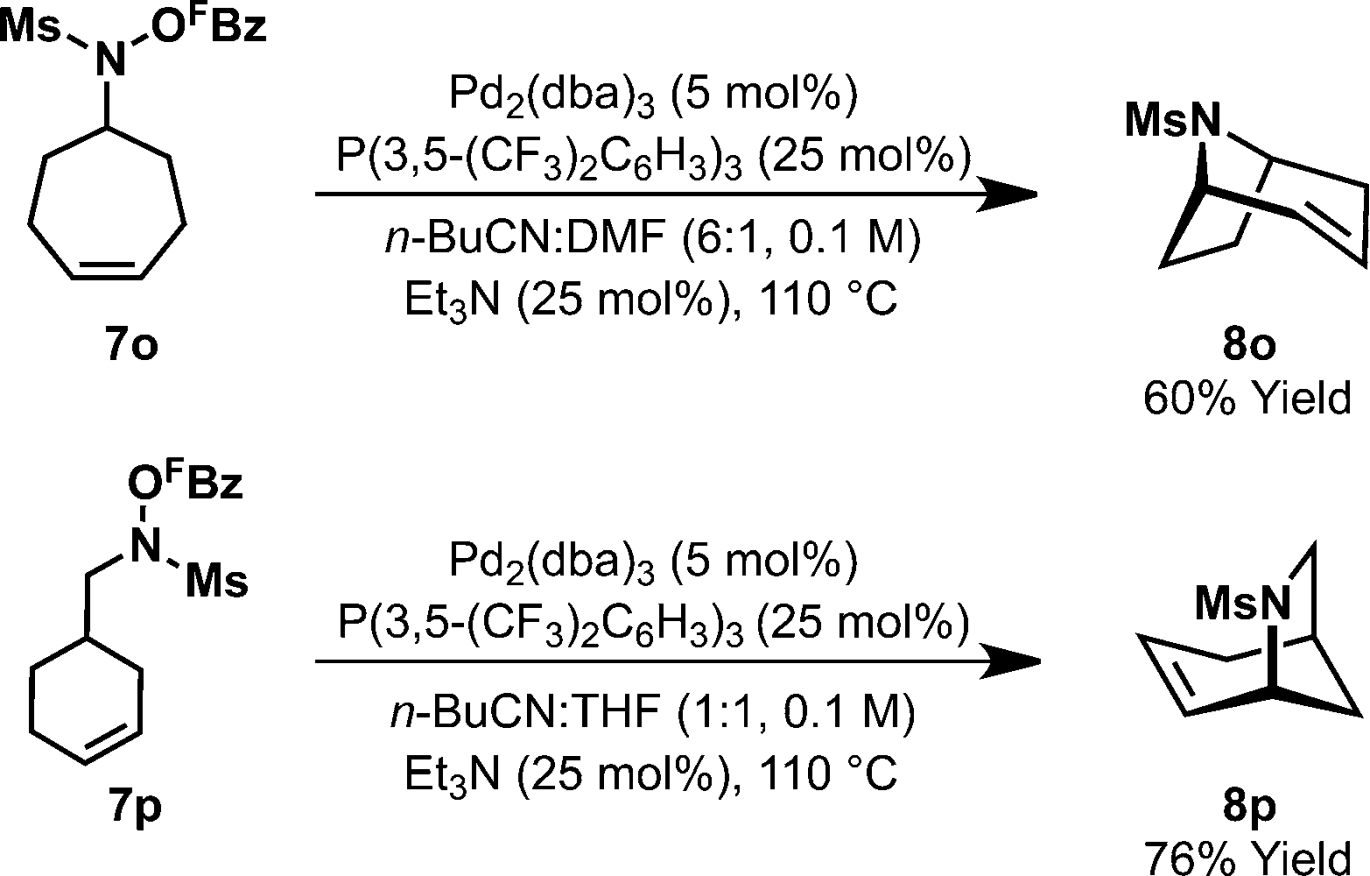
Bridged ring systems by aza‐Heck cyclization.

Preliminary studies show that the chemistry will be of utility in other contexts. All aza‐Heck processes described so far involve 5‐*exo* cyclization; however, even at the present level of development, 6‐*exo* cyclization is possible (Scheme 4 A). Indeed, exposure of styrenyl system **7 q** to optimized conditions provided tetrahydroisoquinoline **8 q** in 42 % yield. We have also assessed the possibility of alkene 1,2‐carboamination processes by trapping the alkyl‐Pd^II^ intermediate generated after migratory insertion (Scheme 4 B). Exposure of **7 r** to aza‐Heck conditions afforded bicycle **8 r** in 86 % yield, via Heck trapping of **7 r′**. The development of further alkene aza‐functionalizations will be a focus of future work.

**Scheme 4:**
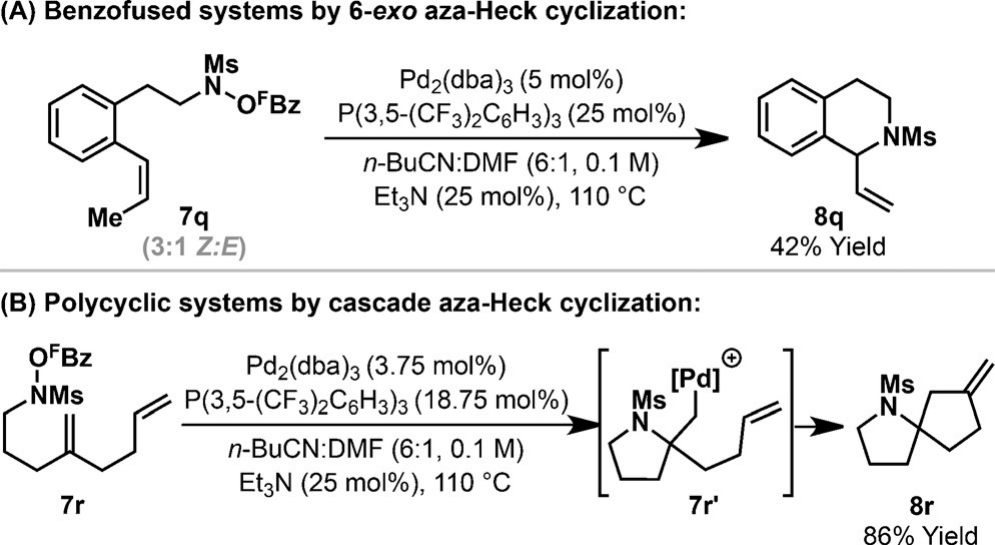
Examples of further reactivity.

The mechanism of the aza‐Heck processes is likely akin to that of the Narasaka cyclization of O‐pentafluorobenzoyl ketoxime esters (Scheme 5, **7 d** to **8 d**).^5^, ^8^ Pd^0^L_*n*_ (L=P(3,5‐(CF_3_)_2_C_6_H_3_)_3_) generated in situ effects *N*‐O oxidative addition of **7 d** to provide **I**; despite extensive efforts, we have so far been unable to isolate aza‐Pd^II^ intermediates related to **I**. Efficient aza‐Heck cyclization requires dissociation of pentafluorobenzoate from **I** to access cationic intermediate **II**.^8^ This assertion is based on the observation that less dissociating leaving groups (for example, O‐Bz) are ineffective, and chloride additives (for example, *n*‐Bu_4_NCl) completely suppress cyclization; in both cases protodepalladation to the corresponding sulfonamide predominates. From **II**, *syn*‐migratory insertion of the alkene generates alkyl‐Pd intermediate **III**. The intermediacy of **III** is corroborated by the cyclization of **7 r** to **8 r**, while support for the feasibility of *syn*‐stereospecific alkene migratory insertion is found in studies on aza‐Wacker cyclizations.^24^, ^25^ From **III**, β‐hydride elimination releases the product (**8 d**) and Pd^II^‐hydride **IV**, which undergoes base (Et_3_N) induced reductive elimination to close the catalytic cycle. The equilibrium between neutral and cationic complexes **I** and **II** is shifted forward by triethylammonium mediated protodecarboxylation of the otherwise inhibitory pentafluorobenzoate leaving group. We have previously shown that this process is rapid,^8^ and ^19^F NMR analysis of crude reaction mixtures has confirmed that it is operative in the current scenario. This also accounts for the use of sub‐stoichiometric (catalytic) quantities of Et_3_N under optimized conditions.

**Scheme 5:**
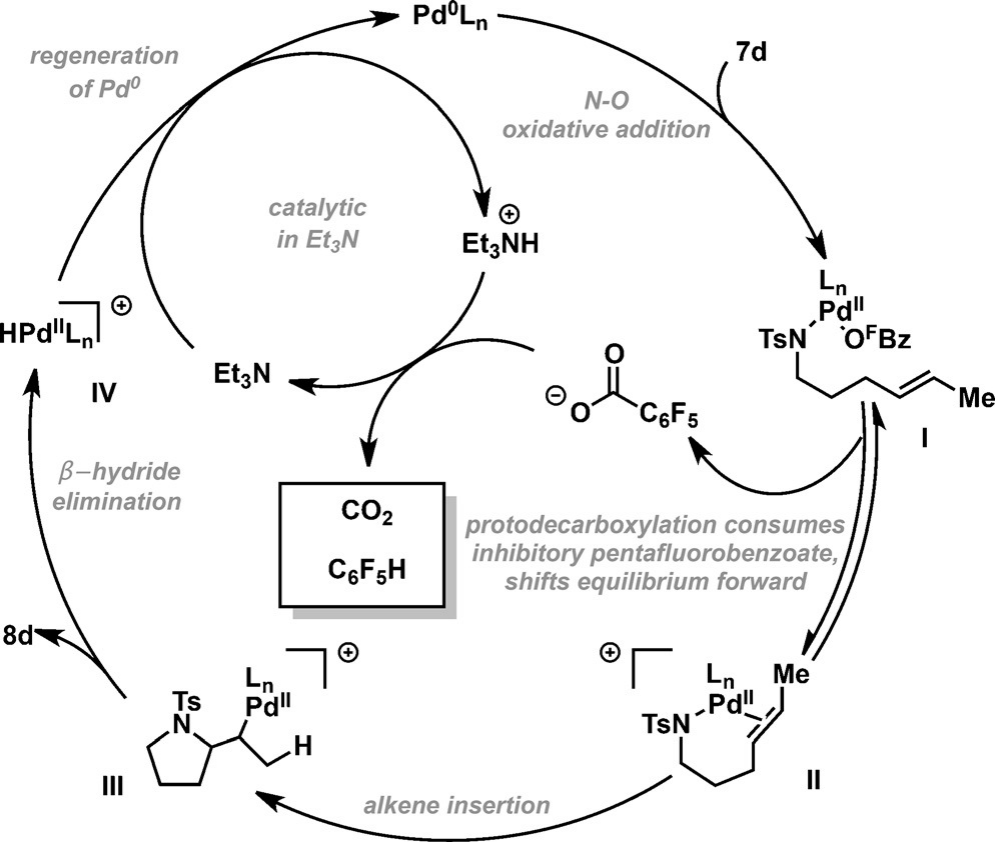
Preliminary mechanism based on observations from current and previous work.

It is pertinent to comment on the synthetic scope of the prototype 5‐*exo* aza‐Heck processes outlined here versus complementary 5‐*exo* aza‐Wacker cyclizations of alkenyl NH‐sulfonamides, which require an external oxidant (for example, air or oxygen).^24^ Despite extensive development, this latter method still has key limitations; for example, cyclization of systems with large α‐substituents (larger than methyl) have not been achieved (cf. **7 j**–**m**), hindered acyclic olefins do not participate (cf. **7 h**), and electron‐deficient alkenes cannot be used due to competing conjugate addition (cf. **7 n**). Additionally, aza‐Heck cyclization seems uniquely suited to demanding systems (Scheme 3) and cascade polycyclizations (Scheme 4 B). Earlier work using oxime esters has also established N‐O oxidative addition as a unified platform for the design of diverse redox‐neutral alkene 1,2‐carboamination processes that cannot be achieved using an aza‐Wacker approach.^8^ From a practical viewpoint, a pre‐installed internal oxidant may be preferable for scale‐up or redox sensitive substrates. Importantly, this unit can be brought in directly by Mitsunobu reaction of **4 a**/**b**, enabling a two‐step conversion of (enantiopure) alcohols to heterocyclic targets. Alkenyl NH‐sulfonamides required for aza‐Wacker cyclization are not usually prepared directly from the alcohol because the requisite primary sulfonamides do not engage efficiently in conventional Mitsunobu reactions.^26^ Further potential advantages of the aza‐Heck approach are that highly tunable phosphine ligands can be used (because oxidative conditions are avoided) and predictable *syn*‐migratory insertion of the alkene can be expected.24c

In summary, we report aza‐Heck cyclizations initiated by oxidative addition of Pd^0^‐catalysts into the N−O bond of *N*‐(pentafluorobenzoyloxy)sulfonamides. These studies provide direct access to *N*‐heterocyclic ring systems that are not accessible using the Narasaka aza‐Heck procedure.^20^ The approach exploits stepwise unveiling of the nitrenoid character embedded within *N*‐(pentafluorobenzoyloxy)sulfonamide reagents. Sequential nucleophilic‐electrophilic C−N bond forming strategies of this type, which involve the intermediacy of a tunable aza‐Pd^II^ intermediate, should enable a wide array of *N*‐heteroannulation processes. By analogy to the utility of oxime ester derived imino‐Pd intermediates (**2**),^4^, ^5^, ^8^, ^9^, ^10^, ^11^, ^12^, ^13^ we also anticipate that the catalysis platform outlined here, which involves a rare example of oxidative addition of Pd^0^ into an N−O bond,^7^ should find broad applicability in the design of redox neutral C−N bond forming methods outside the immediate area of *N*‐heterocyclic chemistry.

## Supporting information

As a service to our authors and readers, this journal provides supporting information supplied by the authors. Such materials are peer reviewed and may be re‐organized for online delivery, but are not copy‐edited or typeset. Technical support issues arising from supporting information (other than missing files) should be addressed to the authors.

Supplementary
