# Supplementary material for: Diverse N‐Heterocyclic Ring Systems via Aza‐Heck Cyclizations of N‐(Pentafluorobenzoyloxy)sulfonamides
Source: Angew Chem Int Ed Engl. 2016 Jul 27;55(37):11198–202. doi: 10.1002/anie.201605152 (PMC5010782; doi:10.1002/anie.201605152)
Supplement: Supplementary file 1 — Supplementary [file ANIE-55-11198-s001.pdf]

Angewandte Chemie
Eine Zeitschrift der Gesellschaft Deutscher Chemiker

Supporting Information

Diverse *N*-Heterocyclic Ring Systems via Aza-Heck Cyclizations of *N*-(Pentafluorobenzoyloxy)sulfonamides

*Ian R. Hazelden[+], Xiaofeng Ma[+], Thomas Langer, and John F. Bower**

anie_201605152_sm_miscellaneous_information.pdf

Supporting Information

Contents

General Experimental Details 1
Experimental Procedures and Data 2
Hydroxylamine reagents 3
Substrate synthesis 6
Catalysis products 42
Mitsunobu inversion study 55
References 57
^{1}H and ^{13}C NMR spectra of novel compounds 58

General Experimental Details

Unless stated, all materials were purchased from commercial sources (Acros, Aldrich, Alfa Aesar, Fluorochem and Strem) and used without any further treatment. Reagents requiring purification were purified using standard laboratory techniques according to methods published by Perrin, Armarego, and Perrin (Pergamon Press, 1966). Catalytic reactions were carried out in Young-type re-sealable tubes. Anhydrous solvents were obtained by distillation using standard procedures or by passage through drying columns supplied by Anhydrous Engineering Ltd. High-boiling solvents were removed from the reaction crudes employing rotary evaporators connected with high-vacuum pumps. Flash column chromatography (FCC) was performed using silica gel (Aldrich 40-63 µm, 230-400 mesh). Thin layer chromatography was performed using aluminium backed $60F_{254}$ silica plates. Visualization was achieved by UV fluorescence or a basic $KMnO_4$ solution and heat. Proton nuclear magnetic resonance spectra (NMR) were recorded at 400 MHz or 500 MHz. ^{13}C NMR spectra were recorded at 100 MHz or 125 MHz as stated. Chemical shifts (δ) are given in parts per million (ppm). Peaks are described as singlets (s), doublets (d), triplets (t), quartets (q), multiplets (m) and broad (br.). Coupling constants (J) are quoted to the nearest 0.5 Hz. All assignments of NMR spectra were based on 2D NMR data (COSY, HSQC and HMBC). In situ yields were determined by employing 1,3,5-trimethoxybenzene as internal standard. Mass spectra were recorded using a Brüker Daltonics FT-ICR-MS Apex 4e 7.0T FT-MS (ESI^+ mode) and a Shimadzu GCMS QP2010+ (EI^+ mode). Infrared spectra were recorded on a Perkin Elmer Spectrum Two FTIR spectrometer as thin films or solids compressed on a diamond plate. Melting points were determined using Stuart SMP30 melting point apparatus and are reported uncorrected. Enantiomeric excess was determined by integration of chromatogram peaks. Chiral SFC was performed on an Agilent 1260 Infinity SFC Control Module system equipped with a quaternary pump, diode array detector and column thermostat under the conditions specified. The numbering of compound structures does not necessarily reflect the numbering contained in the systematic names.

Experimental Procedures and Data

General procedure A: Mitsunobu reaction employing diisopropyl azodicarboxylate

To a solution of alcohol (1.0 eq.), hydroxylamine-derived pronucleophile (1.3 eq.) and PPh_3 (2.0 eq.) in anhydrous THF:PhMe (2:1, 30 mL/mmol) at 0 °C was added a solution of DIAD (1.5 eq.) in anhydrous PhMe (10 mL/mmol) dropwise. The reaction mixture was stirred at room temperature overnight before being concentrated *in vacuo* and loaded directly onto silica gel for purification by FCC.

General procedure B: Mitsunobu reaction employing diethyl azodicarboxylate

To a solution of alcohol (1.0 eq.), hydroxylamine-derived pronucleophile (1.5 eq.) and PPh_3 (2.0 eq.) in anhydrous PhMe:THF (3:1, 8 mL/mmol) at 0 °C was added a solution of DEAD (2.0 eq.) in anhydrous PhMe (2 mL/mmol) dropwise. The reaction mixture was stirred at room temperature overnight before being concentrated *in vacuo* and loaded directly onto silica gel for purification by FCC.

General procedure C: Alkylation of diethyl malonate

To a suspension of NaH (60% weight in mineral oil, 2.0 eq.) in anhydrous THF (*approx.* 3 mL/mmol) at 0 °C was added diethyl malonate (2.0 eq.) dropwise, the reaction mixture was stirred at this temperature for 1 hour before dropwise addition of allylic bromide (1.0 eq.). The reaction mixture was warmed to room temperature and monitored by TLC. Upon completion, the reaction mixture was poured into a solution of KOH (12 eq.) in water:MeOH (1:1) and stirred for 30 minutes. The reaction mixture was acidified with 10 M aqueous HCl (20 eq.), concentrated to an aqueous solution and extracted with EtOAc (*approx.* 3 × 5 mL/mmol).

The crude mixture of malonic acids was dissolved in DMF and heated to reflux for 3 hours before being concentrated *in vacuo* to afford the crude decarboxylated product.

General procedure D: Reduction of carboxylic acids or esters

To a solution of carboxylic acid/ester (1.0 eq.) in anhydrous THF or Et_2O (*approx.* 5 mL/mmol) at 0 °C was added $LiAlH_4$ (*equivalents specified*) dropwise. The reaction mixture was warmed to room temperature and monitored by TLC. Upon completion, the reaction mixture was cooled to 0 °C before addition of water (1 mL/g of $LiAlH_4$), 15 % aq. NaOH (1 mL/g of $LiAlH_4$) and a final portion of water (3 mL/g of $LiAlH_4$), the resulting mixture was filtered through celite® and washed with DCM. The phases were separated and the aqueous phase extracted with DCM (*approx.* 2 × 5 mL/mmol), the organic phases were dried over Na_2SO_4 and concentrated *in vacuo* to afford the product.

General procedure E: Bromination of allylic alcohols

To a solution of alcohol (1.0 eq.) in Et_2O (*approx*. 5 mL/mmol) at 0 °C was added PBr_3 (0.50 eq.), the reaction mixture was warmed to room temperature and monitored by TLC. Upon completion the reaction mixture was poured into an aqueous solution of K_2CO_3 (1.0 eq.), the phases were separated and the aqueous phase was extracted with Et_2O (*approx*. 2 × 4 mL/mmol). The Et_2O phases were dried over Na_2SO_4 and concentrated *in vacuo* afford the product.

General procedure F: Johnson-Claisen rearrangement

A solution of propionic acid (0.20 eq.) in triethyl orthoacetate (10 eq.) was heated to 110 °C for 1 hour, after this time allylic alcohol (1.0 eq.) was added and the reaction mixture heated to reflux overnight. Upon cooling to room temperature the reaction mixture was concentrated *in vacuo* to afford the crude product which was purified by FCC.

General procedure G: Palladium-catalyzed cyclization

A flame-dried re-sealable tube, fitted with a rubber septum, was charged with cyclization substrate, $Pd_2(dba)_3$ and $P(3,5\text{-}(CF_3)_2C_6H_3)_3$. The tube was purged with nitrogen, anhydrous solvent and Et_3N were added *via* syringe. The tube was sealed and heated at the specified temperature for the time noted. The reaction mixture was concentrated *in vacuo* and the crude mixture was purified by FCC to afford the pure product.

Hydroxylamine reagents:

***N*-Tosyl hydroxylamine**

TsNHOH

This compound was prepared according to a literature procedure.[1]

The spectroscopic properties were consistent with the data available in the literature.[2]

***N*-Tosyl-*O*-pentafluorobenzoyl hydroxylamine (4a)**

TsNHO^{F}Bz

To a suspension of *N*-tosylhydroxylamine (1.00 g, 5.34 mmol) and pentafluorobenzoic acid (1.13 g, 5.34 mmol) in DCM (50 mL) at 0 °C was added a solution of *N*-*N'*-dicyclohexylcarbodiimide (1.21 g, 5.88 mmol) in DCM (50 mL) dropwise. The resulting mixture was stirred at 0 °C overnight before

filtration to remove the white precipitate. The filtrate was concentrated *in vacuo* and the crude mixture was purified by FCC (gradient elution 2:1 – 1:1 hexane:EtOAc) to afford **4a** (1.46 g, yield 72%) as a colorless crystalline solid.

m.p. 102-104 °C (DCM:hexane, *needles*)

ν_{max} / cm^{-1}: (solid) 3189 (br s), 1781 (s), 1653 (s), 1597 (m), 1500 (s), 1163 (s).

δ_H (400 MHz, $CDCl_3$) 9.01 (1H, s, N$\underline{H}$), 7.85 (2H, d, *J* = 8.5 Hz, Ts ArC$\underline{H}$), 7.36 (2H, d, *J* = 8.5 Hz, Ts ArC$\underline{H}$), 2.45 (3H, s, Ts C$\underline{H}_3$).

δ_C (101 MHz, $CDCl_3$) 158.0 (^{F}Bz $\underline{C}$=O), 146.4 (Ts Ar$\underline{C}$), 132. (Ts Ar$\underline{C}$), 130.2 (Ts Ar$\underline{C}$H), 129.1 (Ts Ar$\underline{C}$H), 21.9 (Ts $\underline{C}H_3$).

The aromatic signals corresponding to the pentafluorobenzoyl group could not be resolved due to their weak intensity.

δ_F (377 MHz, $CDCl_3$) -134.7 – -134.8 (2F, m), -144.1 (1F, tt, *J* = 21.0, 6.5 Hz), -158.7 – -158.8 (2F, m).

HRMS: (ESI^+) Calculated for $C_{14}H_8F_5NNaO_4$: 403.9986. Found $[M+ Na]^+$: 403.9992.

***N*-Tosyl-*O*-*tert*-butyldimethylsilyl hydroxylamine**

TsNHOTBS

To a suspension of *N*-tosylhydroxylamine (5.00 g, 26.7 mmol) in anhydrous DCM (*aprrox.* 200 mL) at 0 °C was added TBSCl (6.03 g, 40.0 mmol) followed by Et_3N (5.58 mL, 40.0 mmol). The reaction mixture was stirred at room temperature before addition of water (200 mL). The phases were separated and the aqueous phase extracted with DCM (2 × 150 mL). The combined organic phases were washed with brine (200 mL), dried over Na_2SO_4 and concentrated *in vacuo*. The crude mixture was purified by FCC (eluent 3:1 hexane:EtOAc) to afford the title compound (7.16 g, 89 %) as a crystalline colorless solid.

The spectroscopic properties were consistent with the data available in the literature.[3]

***N*-Mesyl hydroxylamine**

MsNHOH

This compound was prepared according to a literature procedure.[1]

ν_{max} / cm^{-1}: (solid) 3373 (br s), 3253 (s), 3036 (m), 1302 (s), 1154 (s).

δ_H (400 MHz, CD_3CN) 7.45 (2H, m, N<u>H</u> and O<u>H</u>), 2.95 (3H, s, C<u>H</u>$_3$).

δ_C (101 MHz, CD_3CN) 36.0 (<u>C</u>H$_3$).

HRMS: (ESI^+) Calculated for CH_5NNaO_3S: 133.9882. Found $[M+Na]^+$: 133.9888.

***N*-Mesyl-*O*-pentafluorobenzoyl hydroxylamine (4b)**

MsNHO^{F}Bz

To a suspension of *N*-mesylhydroxylamine (3.48 g, 31.3 mmol) and pentafluorobenzoic acid (6.64 g, 31.3 mmol) in DCM (150 mL) at 0 °C was added a solution of *N*-*N'*-dicyclohexylcarbodiimide (7.00 g, 34.5 mmol) in DCM (150 mL) dropwise. The resulting mixture was stirred at 0 °C overnight before filtration to remove the white precipitate. The filtrate was concentrated *in vacuo* and the crude mixture was purified by FCC (gradient elution 2:1 – 1:1 hexane:EtOAc) to afford **4b** (7.20 g, 83%) as a colorless solid.

ν_{max} / cm^{-1}: (solid) 3151 (s), 2940 (m), 1759 (s), 1653 (m), 1500 (s), 1324 (s), 1165 (s).

δ_H (400 MHz, $CDCl_3$) 8.72 (1H, br s, N<u>H</u>), 3.20 (3H, d, *J* = 1.0 Hz, Ms C<u>H</u>$_3$).

δ_C (101 MHz, $CDCl_3$) 39.3 (Ms <u>C</u>H$_3$).

The signals corresponding to the pentafluorobenzoyl group could not be resolved due to their weak intensity.

δ_F (377 MHz, $CDCl_3$) -134.5 – -134.6 (2F, m), -143.5 (1F, tt, *J* = 21.0, 6.5 Hz), -158.4 – -158.6 (2F, m).

HRMS: (ESI^+) Calculated for $C_8H_4F_5NNaO_4S$: 327.9673. Found $[M+Na]^+$: 327.9676.

Substrate synthesis:

N-Pent-4-en-1-yl-N-(pentafluorobenzoyloxy)-4-toluenesulfonamide (7a)

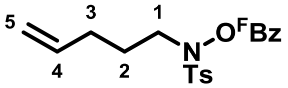

General procedure A: 4-Penten-1-ol (48 μL, 0.475 mmol) and **4a** were employed. FCC (eluents 250:1 hexane:EtOAc followed by PhMe) afforded **7a** (139 mg, 70 %) as a crystalline colorless solid.

m.p. 89-90 °C (Et_2O:hexane)

ν_{max} / cm^{-1}: (solid) 2981 (m), 2927 (m), 1787 (s), 1654 (m), 1595 (m), 1505 (s), 1367 (s), 1170 (s), 1076 (s).

δ_H (400 MHz, $CDCl_3$) 7.80 (2H, d, J = 8.0 Hz, ArCH), 7.38 (2H, d, J = 8.0 Hz, ArCH), 5.75 (1H, ddt, J = 17.0, 10.0, 7.0 Hz, C**4**-H), 5.07 – 4.97 (2H, m, C**5**-H_2), 3.23 (2H, br s, C**1**-H_2), 2.47 (3H, s, Ts CH_3), 2.20 (2H, dt, J = 7.0, 7.0 Hz, C**3**-H_2), 1.67 (tt, J = 7.0, 7.0 Hz, C**2**-H_2).

δ_C (126 MHz, $CDCl_3$) 156.5 (^{F}Bz C=O), 146.0 (ArC), 137.0 (C**4**), 130.2 (ArC), 130.0 (ArCH), 129.8 (ArCH), 116.1 (C**5**), 52.1 (C**1**) , 30.5 (C**3**), 25.9 (C**2**), 21.9 (Ts CH_3).

The aromatic signals corresponding to the pentafluorobenzoyl group could not be resolved due to their weak intensity.

δ_F (377 MHz, $CDCl_3$) -136.0 (2F, dt, J = 19.5, 5.5 Hz), -146.0 (1F, tt, J = 21.0, 5.5 Hz), -158.9 – -159.1 (2F, m).

HRMS: (ESI^+) Calculated for $C_{19}H_{17}F_5NO_4S$: 450.0793. Found $[M+H]^+$: 450.0794.

2-(Cyclohex-2-en-1-yl)acetic acid

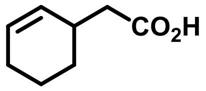

General procedure C: 3-Bromocyclohexene (6.90 mL, 60.0 mmol) was employed to afford the title compound as an orange oil (6.23 g, 74 %) which was used without further purification.

The spectroscopic properties were consistent with the data available in the literature.[4]

2-(Cyclohex-2-en-1-yl)ethan-1-ol

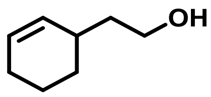

General procedure D: The preceding carboxylic acid (6.20 g, 44.0 mmol) was employed using anhydrous THF as solvent and 1.7 eq. $LiAlH_4$ (1M in THF). The crude product was filtered through a short plug of silica to afford the title compound (4.85 g, 87 %) as a yellow oil.

δ_H (400 MHz, $CDCl_3$) 5.67 (1H, dtd, *J* = 9.5, 3.5, 2.5 Hz), 5.56 (1H, ddt, *J* = 9.5, 2.0, 2.0 Hz), 3.71 (2H, tt, *J* = 7.0, 3.5 Hz), 2.22 (1H, dtdt, *J* = 11.5, 5.5, 2.5, 2.5 Hz), 1.96 (2H, tdd, *J* = 8.0, 4.0, 2.5 Hz), 1.79 (1H, dtd, *J* = 12.0, 6.0, 5.5, 2.5 Hz), 1.71 (1H, dqd, *J* = 12.0, 5.0, 2.5 Hz), 1.61 (1H, dt, *J* = 13.5, 6.5 Hz), 1.56 – 1.50 (1H, m), 1.49 (1H, s), 1.24 (1H, dddd, *J* = 12.5, 11.0, 8.5, 2.5 Hz).

δ_C (101 MHz, $CDCl_3$) 131.6, 127.4, 60.9, 39.2, 31.9, 29.1, 25.3, 21.4.

HRMS: (ESI^+) Calculated for $C_8H_{14}NaO$: 149.0937. Found $[M+Na]^+$: 149.0932.

The spectroscopic properties were consistent with the data available in the literature.[5]

***N*-2-(Cyclohex-2-en-1-yl)ethan-1-yl-*N*-(pentafluorobenzoyloxy)-4-toluenesulfonamide (7ba)**

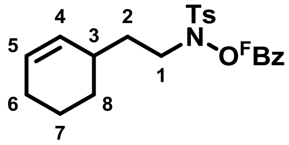

General procedure B: The preceding alcohol (60.0 mg, 0.475 mmol) and **4a** were employed, FCC (eluents 250:1 hexane:EtOAc followed by PhMe) afforded **7ba** (203 mg, 88 %) as a crystalline colorless solid.

m.p. 81-82 °C (Et_2O:hexane)

ν_{max} / cm^{-1}: (solid) 2988 (m), 2929 (m), 1790 (s), 1654 (m), 1596 (m), 1503 (s), 1370 (s), 1170 (s).

δ_H NMR (400 MHz, $CDCl_3$) 7.78 (2H, d, *J* = 8.0 Hz, Ts ArC<u>H</u>), 7.38 (2H, d, *J* = 8.0 Hz, Ts ArC<u>H</u>), 5.67 (1H, dtd, *J* = 10.0, 3.5, 2.5 Hz, C**5**-<u>H</u>), 5.46 (1H, ddt, *J* = 10.0, 2.5, 2.5 Hz, C**4**-<u>H</u>), 3.26 (2H, br s, C**1**-$\underline{H}_2$), 2.46 (3H, s, Ts C$\underline{H}_3$), 2.29 (1H, m, C**3**-<u>H</u>), 1.94 (2H, dddd, *J* = 7.0, 5.0, 3.5, 2.5 Hz, C**6**-$\underline{H}_2$), 1.83 – 1.73 (1H, m, C**8**-<u>H</u>), 1.72 – 1.44 (4H, m, C**2**-$\underline{H}_2$ and C**7**-$\underline{H}_2$), 1.29 – 1.13 (1H, m, C**8**-<u>H</u>’).

δ_C (101 MHz, $CDCl_3$) 156.4 (^{F}Bz C=O), 145.9 (Ts ArC), 130.3 (C**4**), 129.9 x 2 (Ts ArC + Ts ArCH), 129.5 (Ts ArCH), 127.9 (C**5**), 50.6 (C**1**), 32.8 (C**2** or C**7**), 32.2 (C**3**), 28.5 (C**8**), 25.1 (C**6**), 21.7 (Ts CH_3), 21.1 (C**2** or C**7**).

The aromatic signals corresponding to the pentafluorobenzoyl group could not be resolved due to their weak intensity.

δ_F (377 MHz, $CDCl_3$) -136.0 – -136.2 (2F, m), -146.2 (1F, tt, J = 21.0, 5.5 Hz), -159.0 – -159.2 (2F, m).

HRMS: (ESI^+) Calculated for $C_{22}H_{20}F_5NNaO_4S$: 512.0925. Found $[M+Na]^+$: 512.0902.

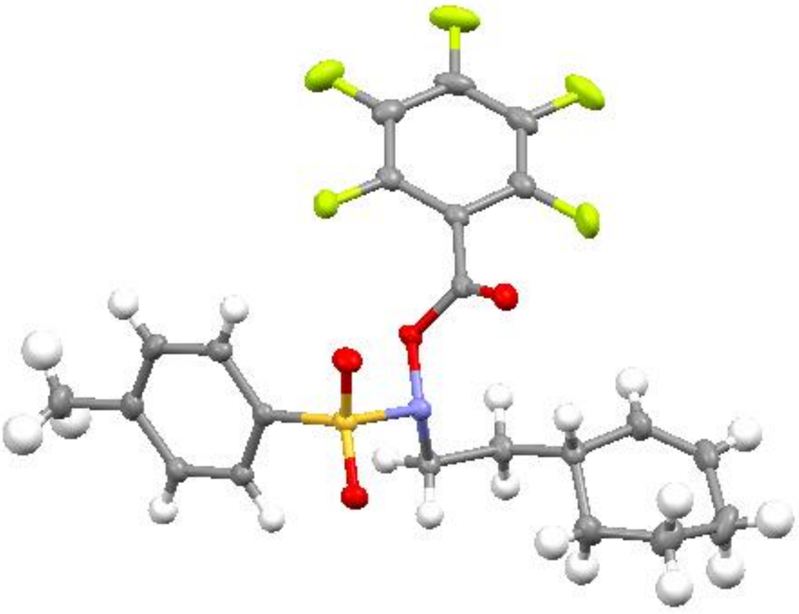

ORTEP view of ***7ba***

2-(Cyclohex-2-en-1-yl)ethan-1-yl tosylate

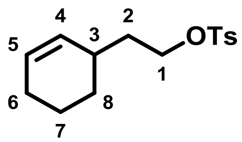

To a solution of 2-(cyclohex-2-en-1-yl)ethan-1-ol (3.95 g, 31.3 mmol) and Et_3N (7.00 mL, 50.2 mmol) in anhydrous DCM (*approx.* 100 mL) at 0 °C was added TsCl (9.24 g, 48.5 mmol) in two portions. The reaction mixture was stirred at room temperature for 22 hours before addition of saturated aqueous $NaHCO_3$ (100 mL), the phases were separated and the aqueous phase was extracted

with DCM (2 x 50 mL). The DCM extracts were dried over Na_2SO_4 and concentrated *in vacuo*. The crude mixture was purified by FCC (eluent 9:1 petroleum ether 40/60:EtOAc) to afford the title compound (8.43 g, 96 %) as a colorless oil.

ν_{max} / cm^{-1}: (film) 3017 (m), 2927 (m), 2860 (m), 1598 (m), 1448 (m), 1361 (s), 1176 (s).

δ_H (500 MHz, $CDCl_3$) 7.80 (2H, d, *J* = 8.5 Hz, ArCH), 7.35 (2H, d, *J* = 8.5 Hz, ArCH), 5.66 (1H, dtd, *J* = 10.0, 4.0, 3.5 Hz, C**5**-H), 5.42 (1H, ddt, *J* = 10.0, 2.5, 2.5 Hz, C**4**-H), 4.10 (2H, t, *J* = 6.5 Hz, C**1**-H), 2.45 (3H, s, Ts CH_3), 2.17 (1H, m, C**3**-H), 1.94 (2H, dddd, *J* = 5.5, 5.5, 4.0, 2.5 Hz, C**6**-H_2), 1.74 – 1.54 (4H, m, C**2**-H, C**2**-H', C**7**-H and C**8**-H), 1.52 – 1.42 (1H, m, C**7**-H'), 1.18 – 1.11 (1H, m, C**8**-H').

δ_C (101 MHz, $CDCl_3$) 144.7 (ArC), 133.12 (ArC), 130.09 (C**4**), 129.79 (ArCH), 127.99 (C**5**), 127.86 (ArCH), 68.64 (C**1**), 35.01 (C**2**), 31.35 (C**3**), 28.45 (C**8**), 25.08 (C**6**), 21.61 (Ts CH_3), 21.02 (C**7**).

HRMS: (ESI^+) Calculated for $C_{15}H_{20}NaO_3S$: 303.1025. Found $[M+Na]^+$: 303.1025.

_N_-(2-(Cyclohex-2-en-1-yl)ethyl)-_N_-((_tert_-butyldimethylsilyl)oxy)-4-toluenesulfonamide

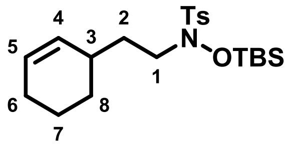

To a solution of the preceding tosylate (1.82 g, 6.49 mmol) and TsNHOTBS (2.35 g, 7.79 mmol) in DMF (40 mL) was added Cs_2CO_3 (3.81 g, 11.7 mmol). The reaction mixture was stirred for 17 hours before addition of saturated aqueous NH_4Cl (50 mL) and water (50 mL). The reaction mixture was extracted with EtOAc (3 x 80 mL) and the combined organic phases were dried over Na_2SO_4 and concentrated *in vacuo*. The crude mixture was purified by FCC (gradient elution 29:1 – 19:1 hexane:EtOAc) to afford the title compound (1.95 g, 73 %) as a colorless oil that solidified upon standing.

ν_{max} / cm^{-1}: (solid) 3021 (m), 2928 (s), 2858 (m), 1597 (m), 1461 (m), 1351 (s), 1249 (s), 1164 (s).

δ_H (500 MHz, $CDCl_3$) 7.73 (2H, d, *J* = 8.5 Hz, ArCH), 7.34 (2H, d, *J* = 8.5 Hz, ArCH), 5.67 (1H, ddt, *J* = 10.0, 3.5, 3.5 Hz, C**5**-H), 5.47 (1H, ddt, *J* = 10.0, 2.0, 2.0 Hz, C**4**-H), 3.04 – 2.89 (2H, m, C**1**-H), 2.45 (3H, s, Ts CH_3), 2.08 (1H, dddt, *J* = 8.5, 6.0, 3.0 Hz, C**3**-H), 1.95 (2H, tdd, *J* = 6.0, 3.0, 1.5 Hz, C**6**-H), 1.78 – 1.71 (1H, m, C**8**-H), 1.71 – 1.58 (2H, m, C**2**-H and C**7**-H), 1.54 – 1.44 (m, 2H C**2**-H'

and C**7**-H’), 1.16 (1H, dddd, *J* = 12.5, 11.0, 8.5, 2.5 Hz, C**8**-H’), 0.92 (9H, s, SiC(CH_3)$_3$), 0.30 (3H, s, SiCH_3), 0.29 (3H, s, SiC’H_3).

δ_C (101 MHz, CDCl$_3$) 144.4 (ArC), 130.8 (C**4**), 130.0 (ArC), 129.9 (ArCH), 129.2 (ArCH), 127.7 (C**5**), 53.9 (C**1**), 33.3 (C**2**), 32.9 (C**3**), 28.7 (C**8**), 26.0 (SiC(CH_3)$_3$), 25.2 (C**6**), 21.6 (Ts CH_3), 21.2 (C**7**), 18.3 (SiC(CH_3)$_3$), -4.2 (2 signals, SiCH_3 + SiC’H_3).

HRMS: (ESI$^+$) Calculated for $C_{21}H_{36}NO_3SSi$: 410.2180. Found [M+H]$^+$: 410.2197.

_N_-(2-(Cyclohex-2-en-1-yl)ethyl)-_N_-hydroxy-4-toluenesulfonamide

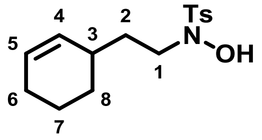

To a solution of the preceding *N*-alkyl-*N*-sulfonyl-*O*-TBS hydroxylamine (1.45 g, 3.54 mmol) in anhydrous THF (30 mL) was added HF·pyridine (70% weight HF, 7.30 mL). The reaction mixture was stirred at room temperature overnight before being quenched with a solution of K_2CO_3 (20.0 g) in water (100 mL). The resulting phases were separated the aqueous phase extracted with DCM (3 × 100 mL). The combined organic phases were washed with 1 M aqueous HCl, dried over Na_2SO_4, filtered through a short plug of silica and concentrated *in vacuo* to afford the title compound as a colorless crystalline solid (1.01 g, 98 %) which was used in the next step without further purification.

ν_{max} / cm^{-1}: (solid) 3356 (br s), 2921 (m), 1597 (m), 1437 (m), 1330 (s), 1163 (s), 1088 (s).

δ_H (500 MHz, CDCl$_3$) 7.80 (2H, d, *J* = 8.5 Hz, ArCH), 7.39 (2H, d, *J* = 8.5 Hz, ArCH), 6.28 (1H, s, OH), 5.73 – 5.67 (1H, m, C**5**-H), 5.57 – 5.52 (1H, m, C**4**-H), 3.07 – 2.93 (2H, m, C**1**-H_2), 2.48 (3H, s, Ts CH_3), 2.21 (1H, dddd, *J* = 11.5, 5.5, 5.5, 2.5 Hz, C**3**-H), 1.98 (2H, dddd, *J* = 6.5, 4.5, 3.0, 1.5 Hz, C**6**-H_2), 1.86 – 1.78 (1H, m, C**8**-H), 1.73 – 1.64 (2H, m, C**2**-H and C**7**-H), 1.59 – 1.46 (2H, m, C**2**-H’ and C**7**-H’), 1.22 (1H, dddd, *J* = 12.5, 11.5, 8.5, 3.0 Hz, C**8**-H’).

δ_C (126 MHz, CDCl$_3$) 144.9 (ArC), 130.9 (C**4**), 129.7 (ArCH), 129.6 (ArCH), 129.4 (ArC), 127.7 (C**5**), 50.3 (C**1**), 33.0 (C**2**), 32.5 (C**3**), 28.7 (C**8**), 25.2 (C**6**), 21.7 (Ts CH_3), 21.2 (C**7**).

HRMS: (ESI$^+$) Calculated for $C_{15}H_{21}NNaO_3S$: 318.1134. Found [M+Na]$^+$: 318.1123.

N-2-(Cyclohex-2-en-1-yl)ethyl-N-(methanesulfonyloxy)-4-toluenesulfonamide (7bb)

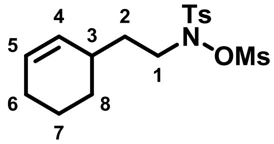

To a solution of *N*-2-(cyclohex-2-en-1-yl)ethyl-*N*-hydroxy-4-toluenesulfonamide (630 mg, 2.13 mmol) in anhydrous DCM (30 mL) at 0°C was added MsCl (325 µL, 4.19 mmol) followed by Et_3N (590 µL, 4.2 mmol). The reaction mixture was warmed to room temperature and stirred overnight before addition of MeOH (15 mL), saturated aqueous $NaHCO_3$ (30 mL) and brine (30 mL). The resulting phases were separated and the aqueous phase was extracted with DCM (3 × 30 mL). The organic phases were dried over Na_2SO_4 and concentrated *in vacuo*. The crude mixture was purified by FCC (eluent 49:1 toluene:EtOAc) to afford **7bb** (660 mg, 83 %) as a crystalline colorless solid.

m.p. 101-102 °C (Et_2O:hexane)

ν_{max} / cm^{-1}: (solid) 2921 (m), 2861 (m), 1598 (m), 1451 (m), 1373 (s), 1355 (s), 1175 (s), 1088 (s).

δ_H (400 MHz, $CDCl_3$) 7.78 (2H, d, *J* = 8.0 Hz, ArCH), 7.41 (2H, d, *J* = 8.0 Hz, ArCH), 5.68 (1H, ddt, *J* = 10.0, 3.5, 3.0 Hz, C**5**-H), 5.51-5.46 (1H, m, C**4**-H), 3.37 (3H, s, Ms CH_3), 3.23 (2H, br s, C**1**-H_2). 2.49 (3H, s, Ts CH_3), 2.21 – 2.13 (1H, m, C**3**-H), 1.94 (2H, ddd, *J* = 5.5, 5.5, 3.0 Hz, C**6**-H_2), 1.82 – 1.61 (4H, m, C**2**-H, C**2**-H', C**7**-H and C**8**-H), 1.57 – 1.44 (1H, m, C**7**-H'), 1.28 – 1.13 (1H, m, C**8**-H').

δ_C (126 MHz, $CDCl_3$) 146.4 (ArC), 130.6 (C**4**), 130.2 (ArCH), 129.9 (ArCH), 129.3 (ArC), 128.0 (C**5**), 54.4 (C**1**), 37.9 (Ms CH_3), 33.2 (C**2**), 32.7 (C**3**), 28.7 (C**8**), 25.3 (C**6**), 21.9 (Ts CH_3), 21.3 (C**7**).

HRMS: (ESI^+) Calculated for $C_{16}H_{23}NNaO_5S_2$: 396.0910. Found $[M+Na]^+$: 396.0924.

N-2-(Cyclohex-2-en-1-yl)ethyl-N-(trifluoroacetyloxy)-4-toluenesulfonamide (7bc)

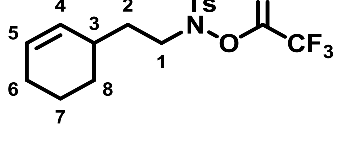

N-2-(Cyclohex-2-en-1-yl)ethyl-*N*-hydroxy-4-toluenesulfonamide (200 mg, 0.677 mmol) was dissolved in trifluoroacetic anhydride (1.00 mL) and stirred for one hour before being concentrated *in*

vacuo and analyzed by NMR. These steps were repeated until complete conversion was achieved. **7bc** (263 mg, 0.672 mmol, 99 %) was isolated as an amorphous orange solid. This product was employed in the catalytic reactions immediately due to its instability to hydrolysis.

ν_{max} / cm^{-1}: (solid) 3024 (m), 2926 (m), 1820 (s), 1597 (m), 1371 (s), 1220 (s), 1165 (s), 1109 (s).

δ_H (400 MHz, $CDCl_3$) 7.77 (2H, d, *J* = 8.5 Hz, ArCH), 7.42 (2H, d, *J* = 8.5 Hz, ArCH), 5.70 (1H, ddt, *J* = 10.0, 3.5, 3.0 Hz, C**5**-H), 5.46 (1H, ddt, *J* = 10.0, 2.5, 2.5 Hz, C**4**-H), 3.23 (2H, br s, C**1**-H_2), 2.49 (3H, s, Ts CH_3), 2.30 – 2.19 (1H, m, C**3**-H), 1.96 (2H, m, C**6**-H_2), 1.81 – 1.73 (1H, m, C**8**-H), 1.72 – 1.64 (1H, m, C**7**-H), 1.60 – 1.41 (3H, m, C**2**-H, C**2**-H' and C**7**-H'), 1.18 (1H, dddd, *J* = 13.0, 10.5, 8.0, 2.5 Hz, C**8**-H').

δ_C (101 MHz, $CDCl_3$) 146.3 (ArC), 130.1 (ArCH), 130.0 (C**4**), 129.6 (ArCH), 129.0 (ArC), 128.2 (C**5**), 50.8 (C**1**), 32.7 (C**2**), 32.2 (C**3**), 28.5 (C**8**), 25.1 (C**6**), 21.8 (Ts CH_3), 21.0 (C**7**).

The signal corresponding to the trifluoroacetyl group could not be resolved due to its weak intensity.

δ_F (377 MHz, C_6D_6) -73.2 (3F, s).

HRMS: (ESI^+) Calculated for $C_{17}H_{20}F_3NNaO_4S$: 414.0957. Found $[M+Na]^+$: 414.0965.

2-(Cyclopent-2-en-1-yl)ethan-1-ol

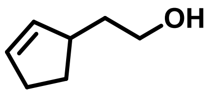

General procedure D: 2-Cyclopentene-1-acetic acid (806 mg, 6.81 mmol) was employed, using anhydrous Et_2O as the solvent and 2.0 eq. $LiAlH_4$ (1M in Et_2O). The title compound (713 mg, 93 %) was isolated as a pale yellow oil.

ν_{max} / cm^{-1}: (film) 3327 (br s), 3051 (m), 2928 (s), 2851 (s), 1614 (m), 1057 (s).

δ_H (400 MHz, $CDCl_3$) 5.74 (1H, ddt, *J* = 6.0, 2.0, 2.0 Hz), 5.69 (1H, ddt, *J* = 6.0, 2.0, 2.0 Hz), 3.76 – 3.64 (2H, m), 2.82 – 2.72 (1H, m), 2.41 – 2.22 (2H, m), 2.12 – 2.02 (1H, m, 1H), 1.70 (1H, ddt, *J* = 13.5, 6.5, 6.5 Hz), 1.62 – 1.52 (1H, m), 1.49 – 1.36 (2H, m).

δ_C (101 MHz, $CDCl_3$) 134.6, 130.7, 61.8, 42.1, 38.9, 31.9, 29.8.

The spectroscopic properties were consistent with the data available in the literature.[6]

N-2-(Cyclopent-2-en-1-yl)ethyl-N-(pentafluorobenzoyloxy)-4-toluenesulfonamide (7c)

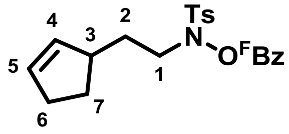

General procedure B: The preceding alcohol (53.3 mg, 0.475 mmol) and **4a** were employed. FCC (eluents 250:1 hexane:EtOAc followed by PhMe) afforded **7c** (200 mg, 89 %) as a crystalline colorless solid.

m.p. 95-96 °C (Et_2O:hexane, *needles*)

ν_{max} / cm^{-1}: (solid) 3056 (m), 2936 (m), 1792 (s), 1655 (m), 1595 (m), 1504 (s), 1667 (s).

δ_H (400 MHz, $CDCl_3$) 7.80 (2H, d, *J* = 8.5 Hz, ArCH), 7.38 (2H, d, *J* = 8.5 Hz, ArCH), 5.74 (1H, ddt, *J* = 5.5, 2.0, 2.0 Hz, C**5**-H), 5.61 (1H, ddt, *J* = 5.5, 2.0, 2.0 Hz, C**4**-H), 3.26 (2H, br s, C**1**-H_2), 2.88 – 2.76 (1H, m, C**3**-H), 2.47 (3H, s, Ts CH_3), 2.39 – 2.21 (2H, m, C**6**-H_2), 2.06 (1H, dtd, *J* = 13.0, 8.5, 5.0 Hz, C**7**-H), 1.70 (1H, ddt, *J* = 13.5, 7.0, 7.0 Hz, C**2**-H), 1.63 – 1.55 (1H, m, C**2**-H’), 1.37 (1H, ddt, *J* = 13.0, 9.0, 6.5 Hz, C**7**-H’).

δ_C (101 MHz, $CDCl_3$) 146.0 (ArC), 133.7 (C**4**), 131.6 (C**5**), 130.2 (ArC), 130.0 (ArCH), 129.8 (ArCH), 51.5 (C**1**), 42.7 (C**3**), 32.8 (C**2**), 32.0 (C**6**), 29.7 (C**7**), 21.9 (Ts CH_3).

The signals corresponding to the pentafluorobenzoyl group could not be resolved due to their weak intensity.

δ_F (377 MHz, $CDCl_3$) -136.0 – -136.1 (2F, m), -146.1 (1F, tt, *J* = 21.0, 5.0 Hz), -159.0 – -159.2 (2F, m).

HRMS: (ESI^+) Calculated for $C_{21}H_{18}F_5NNaO_4S$: 498.0769. Found $[M+Na]^+$: 498.0786.

(E)-N-Hex-4-en-1-yl-N-(pentafluorobenzoyloxy)-4-toluenesulfonamide (7d)

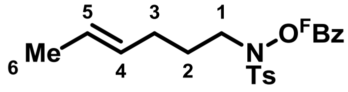

General procedure A: The preceding alcohol (58 μL, 0.475 mmol) and **4a** were employed. FCC (eluents 250:1 hexane:EtOAc followed by PhMe) afforded **7d** (156 mg, 71 %, 11:1 mixture of *E* and *Z* isomers) as a crystalline colorless solid.

ν_{max} / cm^{-1}: (solid) 2936 (m), 1787 (s), 1652 (m), 1597 (m), 1504 (s), 1368 (s), 1325 (s), 1170 (s).

HRMS: (ESI^+) Calculated for $C_{20}H_{18}F_5NNaO_4S$: 486.0769. Found $[M+Na]^+$: 486.0760.

Spectroscopic data for the major E isomer:

δ_H (500 MHz, $CDCl_3$) 7.79 (2H, d, *J* = 8.5 Hz, ArC<u>H</u>), 7.37 (2H, d, *J* = 8.5 Hz, ArC<u>H</u>), 5.44 (1H, dqt, *J* = 15.0, 6.0, 1.5 Hz, C**5**-<u>H</u>), 5.34 (1H, dtq, *J* = 15.0, 7.0, 1.5 Hz, C**4**-<u>H</u>), 3.21 (2H, br s, C**1**-$\underline{H}_2$), 2.46 (3H, s, Ts C$\underline{H}_3$), 2.11 (2H, dtt, *J* = 7.0, 7.0, 1.5 Hz, C**3**-$\underline{H}_2$), 1.65 – 1.59 (5H, m, C**2**-$\underline{H}_2$ and C**6**-$\underline{H}_3$).

δ_C (126 MHz, $CDCl_3$) 156.3 (^{F}Bz <u>C</u>=O), 145.8 (Ar<u>C</u>), 130.1 (Ar<u>C</u>), 129.8 (Ar<u>C</u>H), 129.6 (Ar<u>C</u>H), 129.3 (C**4**), 126.5 (C**5**), 52.0 (C**1**), 29.2 (C**3**), 26.4 (C**2**), 21.7 (Ts C$\underline{H}_3$), 17.9 (C**6**).

The signals corresponding to the pentafluorobenzoyl group could not be resolved due to their weak intensity.

δ_F (377 MHz, $CDCl_3$) -135.9 – -136.0 (2F, m), -146.1 (1F, tt, *J* = 21.0, 5.5 Hz), -158.9 – -159.1 (2F, m).

Characteristic signals for the minor Z isomer (obtained from 1D TOCSY, irradiated signal at 2.19 ppm):

δ_H (500 MHz, $CDCl_3$) 5.53 – 5.46 (m), 5.35 – 5.25 (m), 3.20 (s), 2.19 (dt, *J* = 7.5, 7.5 Hz), 1.66 – 1.58 (m).

(*E*)-6-Phenylhex-4-en-1-ol

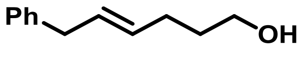

To a solution of Hoveyda-Grubbs 2nd generation catalyst (109 mg, 0.174 mmol) in anhydrous, degassed DCM (80 mL) was added simultaneously 4-penten-1-ol (1.20 mL, 11.6 mmol) and allyl benzene (5.80 mL, 44.0 mmol). The reaction mixture was stirred for 2 days at room temperature before being concentrated *in vacuo* and purified by FCC (eluent 4:1 toluene:EtOAc) to afford the title compound (0.840 g, 41 %, 7:1 mixture of *E* and *Z* isomers) as a brown oil (the coloration was due to the presence of trace amounts of Ru-impurities).

ν_{max} / cm^{-1}: (film) 3334 (s), 3026 (m), 2932 (m), 1603 (m), 1494 (s), 1452 (s), 1054 (s).

Spectroscopic data for the major E isomer:

δ_H (400 MHz, $CDCl_3$) 7.36 – 7.28 (2H, m), 7.25 – 7.18 (3H, m), 5.65 (1H, dt, J = 15.0, 6.5 Hz), 5.55 (1H, dt, J = 15.0, 6.5 Hz), 3.66 (2H, t, J = 6.5 Hz), 3.37 (2H, d, J = 6.5 Hz), 2.15 (2H, dt, J = 7.5, 6.5 Hz), 1.96 (1H, br s), 1.71 – 1.63 (2H, m).

δ_C (101 MHz, $CDCl_3$) 140.9, 131.1, 129.6, 128.5, 128.4, 125.6, 62.4, 39.1, 32.4, 28.8.

Characteristic signals for the minor Z isomer:

δ_H (400 MHz, $CDCl_3$) 3.45 (2H, d, J = 7.0 Hz), 2.28 (1H, dt, J = 7.0, 7.0 Hz).

The spectroscopic properties were consistent with the data available in the literature.[7,8]

(*E*)-*N*-6-Phenylhex-4-en-1-yl-*N*-(pentafluorobenzoyloxy)-4-toluenesulfonamide (7e)

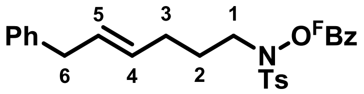

<u>General procedure B:</u> The preceding alcohol (39.4 mg, 0.223 mmol) and **4a** were employed. FCC (eluents 250:1 hexane:EtOAc followed by PhMe) afforded **7e** (69.2 mg, 57 %, 7:1 mixture of *E* and *Z* isomers) as a crystalline colorless solid.

ν_{max} / cm^{-1}: (solid) 2970 (m), 2920 (m), 1784 (s), 1655 (m), 1598 (m), 1494 (s), 1167 (s).

Spectroscopic data for the major E isomer:

δ_H (400 MHz, $CDCl_3$) 7.76 (2H, d, J = 8.5 Hz, Ts ArC<u>H</u>), 7.35 (2H, d, J = 8.5 Hz, Ts ArC<u>H</u>), 7.30 – 7.23 (2H, m, ArC<u>H</u>), 7.21 – 7.13 (3H, m, ArC<u>H</u>), 5.60 (1H, dt, J = 15.0, 6.5 Hz, C**5**-<u>H</u>), 5.43 (1H, dt, J = 15.0, 7.0 Hz, C**4**-<u>H</u>), 3.31 (2H, d, J = 6.5 Hz, C**6**-$\underline{H_2}$), 3.20 (2H, br s, C**1**-$\underline{H_2}$), 2.45 (3H, s, Ts $C\underline{H_3}$), 2.17 (2H, dt, J = 7.0, 7.0 Hz, C**3**-$\underline{H_2}$), 1.65 (2H, tt, J = 7.0, 7.0 Hz, C**2**-$\underline{H_2}$).

δ ^{13}C (101 MHz, $CDCl_3$) 146.0 (Ts Ar<u>C</u>), 140.8 (Ar<u>C</u>), 130.8 (C**5**), 130.2 (Ts Ar<u>C</u>), 130.0 (Ts Ar<u>C</u>H), 129.9 (C**4**), 129.7 (Ts Ar<u>C</u>H), 128.6 (Ar<u>C</u>H), 128.5 (Ar<u>C</u>H), 126.1 (Ar<u>C</u>H), 52.2 (C**1**), 39.1 (C**6**), 29.3 (C**3**), 26.5 (C**2**), 21.9 (Ts <u>C</u>H_3).

The signals corresponding to the pentafluorobenzoyl group could not be resolved due to their weak intensity.

δ_F (377 MHz, $CDCl_3$) -135.9 – -136.0 (2F, m), -146.1 (1F, tt, *J* = 21.0, 5.5 Hz), -158.9 – -159.1 (2F, m).

Characteristic signals for the minor Z isomer:

δ_H (400 MHz, $CDCl_3$) 3.40 (2H, d, *J* = 7.5 Hz), 2.32 (2H, q, *J* = 7.0 Hz).

HRMS: (ESI^+) Calculated for $C_{26}H_{22}F_5NNaO_4S$: 562.1082. Found $[M+Na]^+$: 562.1072.

(*E*)-2-Methylbut-2-en-1-ol

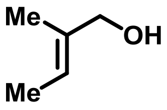

<u>General procedure D:</u> Tiglic acid (15.0 g, 150 mmol) was employed, using anhydrous Et_2O as the solvent and 1.1 eq. $LiAlH_4$ (1M in Et_2O). The title compound (10.8 g, 84 %) was isolated as a colorless oil.

The spectroscopic properties were consistent with the data available in the literature.[9]

(*E*)-1-Bromo-2-methylbut-2-ene

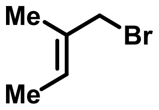

<u>General procedure E:</u> The preceding allylic alcohol (10.4 g, 121 mmol) was employed, the title compound (11.6 g, 63 %) was isolated as a colorless oil.

The spectroscopic properties were consistent with the data available in the literature.[10]

(*E*)-4-Methylhex-4-enoic acid

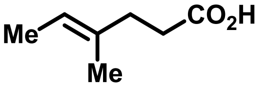

<u>General procedure C:</u> The preceding allylic bromide (1.18 ml, 10.0 mmol) was employed, the crude product was used in the next step without further purification.

The ^{1}H NMR spectrum was consistent with the data available in the literature.[11]

(*E*)-4-Methylhex-4-en-1-ol

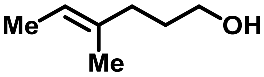

<u>General procedure D:</u> The preceding crude carboxylic acid was employed, using anhydrous Et_2O as the solvent and 2.0 eq. $LiAlH_4$ (1M in Et_2O). The crude mixture was purified by FCC (eluent 4:1 pentane:Et_2O) to afford the title compound (618 mg, 54 % over two steps) as a pale yellow oil.

ν_{max} / cm^{-1}: (film) 3327 (br s), 2936 (s), 2863 (s), 1444 (s), 1381 (s), 1059 (s).

δ_H (400 MHz, $CDCl_3$) 5.28 – 5.22 (1H, m), 3.63 (2H, t, *J* = 6.5 Hz), 2.09 – 2.03 (2H, m), 1.71 – 1.63 (2H, m), 1.61 (3H, t, *J* = 1.0 Hz), 1.57 (3H, dq, *J* = 6.5, 1.0 Hz), 1.34 (1H, br s, OH).

δ_C (101 MHz, $CDCl_3$) 135.6, 119.0, 63.1, 36.1, 30.9, 15.7, 13.5.

The spectroscopic properties were consistent with the data available in the literature.[12]

(*E*)-*N*-4-Methylhex-4-en-1-yl-*N*-(pentafluorobenzoyloxy)-methanesulfonamide (7f)

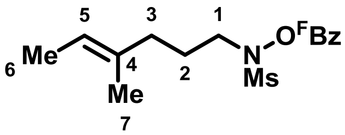

<u>General procedure B:</u> The preceding alcohol (54.2 mg, 0.475 mmol) and **4b** were employed. FCC (eluents 250:1 hexane:EtOAc followed by PhMe) afforded **7f** (121 mg, 63 %) as a crystalline colorless solid.

m.p. 67-68 °C (Et_2O:hexane, *plates*)

ν_{max} / cm^{-1}: (solid) 2936 (m), 1780 (s), 1653 (m), 1502 (s), 1354 (s), 1163 (s).

δ_H (400 MHz, $CDCl_3$) 5.26 (1H, q, *J* = 6.5 Hz, C**5**-H), 3.43 (2H, br s, C**1**-H_2), 3.04 (3H, s, Ms CH_3), 2.15 (2H, t, *J* = 7.5 Hz, C**3**-H_2), 1.78 (2H, tt, *J* = 7.5 Hz, C**2**-H_2), 1.61 – 1.56 (6H, m, C**6**-H_3 and C**7**-H_3).

δ_C (101 MHz, $CDCl_3$) 133.9 (C**4**), 120.2 (C**5**), 52.2 (C**1**), 36.3 (C**3**), 34.5 (Ms CH_3), 25.1 (C**2**), 15.5 (C**7**), 13.5 (C**6**).

The signals corresponding to the pentafluorobenzoyl group could not be resolved due to their weak intensity.

δ_F (377 MHz, $CDCl_3$) -135.8 – -135.9 (2F, m), -145.3 (1F, tt, *J* = 21.0, 5.5 Hz), -158.8 – -159.0 (2F, m).

HRMS: (ESI$^+$) Calculated for $C_{15}H_{16}F_5NNaO_4S$: 424.0612. Found $[M+Na]^+$: 424.0610.

Cyclohex-1-en-ylmethanol

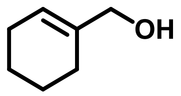

To a solution of methyl 1-cyclohexene-1-carboxylate (5.00 g, 35.6 mmol) in anhydrous DCM (*approx.* 100 mL) at -78 °C was added diisobutyl aluminium hydride (1.0 M in DCM, 78 mL, 78 mmol). The reaction mixture was stirred at this temperature for 2 hours before addition of MeOH (70 mL) and saturated aqueous Rochelle's salt (70 mL). The mixture was warmed to room temperature and stirred overnight before the resulting phases were separated and the aqueous phase extracted with EtOAc (2 × 100 mL). The EtOAc extracts were washed with brine (100 mL) and saturated aqueous Rochelle's salt (100 mL), dried over Na_2SO_4 and concentrated *in vacuo*. The crude mixture was purified by FCC (gradient elution 8:1 – 4:1 hexane:EtOAc) to afford the title compound (3.98 g, 100 %) as a colorless oil.

The spectroscopic properties were consistent with the data available in the literature.[10]

1-(Bromomethyl)cyclohex-1-ene

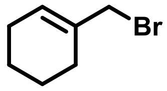

General procedure E: The preceding allylic alcohol (3.50 g, 31.1 mmol) was employed to afford the title compound (4.26 g, 78 %) as a colorless oil.

The spectroscopic properties were consistent with the data available in the literature.[10]

3-(Cyclohex-1-en-yl)propanoic acid

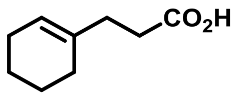

General procedure C: The preceding allylic bromide (1.36 mL, 10.0 mmol) was employed, the crude product was used in the next step without further purification.

The spectroscopic properties were consistent with the data available in the literature.[13]

3-(Cyclohex-1-en-yl)propan-1-ol

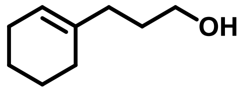

General procedure D: The preceding crude carboxylic acid was employed, using anhydrous THF as the solvent and 1.5 eq. $LiAlH_4$ (1M in Et_2O). The crude mixture was purified by FCC (eluent 4:1 hexane:EtOAc) to afford the title compound (1.26 g, 90 % over two steps) as a pale yellow oil.

ν_{max} / cm^{-1}: (film) 3326 (br s), 2923 (s), 2834 (s), 1438 (m), 1058 (s).

δ_H (400 MHz, $CDCl_3$) 5.44 – 5.40 (1H, m), 3.61 (2H, t, *J* = 6.5 Hz), 2.02 – 1.88 (6H, m), 1.74 (1H, s), 1.69 – 1.48 (6H, m).

δ_C (101 MHz, $CDCl_3$) 137.5, 121.4, 63.0, 34.5, 30.6, 28.3, 25.3, 23.1, 22.6.

The spectroscopic properties were consistent with the data available in the literature.[13]

***N*-3-(Cyclohex-1-en-yl)propan-1-yl-*N*-(pentafluorobenzoyloxy)-methanesulfonamide (7g)**

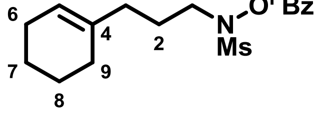

General procedure B: The preceding alcohol (76.1 mg, 0.543 mmol) and **4b** were employed. FCC (eluents 250:1 hexane:EtOAc followed by PhMe) afforded **7g** (121 mg, 52 %) as a crystalline colorless solid.

m.p. 98-99 °C (Et_2O:hexane, *needles*)

ν_{max} / cm^{-1}: (solid) 3023 (m), 2932 (m), 1779 (s), 1653 (m), 1501 (s), 1323 (s), 1162 (s).

δ_H (400 MHz, $CDCl_3$) 5.46 – 5.41 (1H, m, C**5**-H), 3.45 (3H, br t, *J* = 7.0 Hz, C**1**-H_2), 3.04 (3H, s, Ms CH_3), 2.10 (2H, t, *J* = 7.5 Hz, C**3**-H_2), 2.01 – 1.94 (2H, m, C**6**-H_2), 1.92 – 1.87 (2H, m, C**9**-H_2), 1.78 (2H, tt, *J* = 7.5, 7.0 Hz, C**2**-H_2), 1.65 – 1.58 (2H, m, C**7**-H_2), 1.58 – 1.50 (2H, m, C**8**-H_2).

δ_C (101 MHz, $CDCl_3$) 136.0 (C**4**), 122.5 (C**5**), 52.3 (C**1**), 34.7 (C**3**), 34.6 (Ms CH_3), 28.2 (C**9**), 25.3 (C**6**), 24.9 (C**2**), 23.0 (C**8**), 22.6 (C**7**).

The signals corresponding to the pentafluorobenzoyl group could not be resolved due to their weak intensity.

δ_F (377 MHz, $CDCl_3$) -135.8 – -136.0 (2F, m), -145.3 (1F, tt, *J* = 21.0, 5.5 Hz), -158.7 – -158.9 (2F, m).

HRMS: (ESI^+) Calculated for $C_{17}H_{18}F_5NNaO_4S$: 450.0769. Found $[M+Na]^+$: 450.0769.

2-Benzylacrylaldehyde

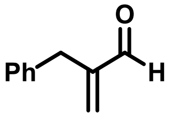

This compound was prepared according to a literature procedure.[14]

The spectroscopic properties were consistent with the data available in the literature.[14]

3-Benzylbut-3-en-2-ol

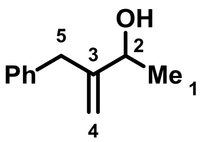

To a solution of MeLi (1.6 M in Et_2O, 16.0 mL, 25.6 mmol) in anhydrous THF (20 mL) at 0 °C was added a solution of the preceding aldehyde (2.50 g, 17.1 mmol) in anhydrous THF (15 mL) dropwise. The reaction mixture was stirred for 1 hour at room temperature before addition of saturated aqueous NH_4Cl (50 mL). The resulting phases were separated and the aqueous phase extracted with Et_2O (2 × 50 mL), the organic phases were dried over Na_2SO_4 and concentrated *in vacuo* to afford the title compound (2.67 g, 96 %) as a pale yellow oil.

ν_{max} / cm^{-1}: (film) 3349 (br s), 3027 (m), 2975 (m), 1647 (m), 1453 (m), 1070 (s).

δ_H (400 MHz, $CDCl_3$) 7.33 – 7.27 (2H, m, ArCH), 7.24 – 7.19 (3H, m, ArCH), 5.16 – 5.14 (1H, m, C**4**-H), 4.75 (1H, d, *J* = 1.5 Hz, C**4**-H’), 4.26 (1H, q, *J* = 6.5 Hz, C**2**-H), 3.48 (1H, d, *J* = 15.5 Hz, C**5**-H), 3.36 (1H, d, *J* = 15.5 Hz, C**5**-H’), 1.52 (1H, br s, OH), 1.31 (3H, d, *J* = 6.5 Hz, C**1**-H_3).

δ_C (101 MHz, $CDCl_3$) 152.7 (C**3**), 139.5 (ArC), 129.3 (ArCH), 128.5 (ArCH), 126.3 (ArCH), 111.0 (C**4**), 70.3 (C**2**), 39.1 (C**5**), 22.4 (C**1**).

HRMS: (ESI^+) Calculated for $C_{11}H_{14}NaO$: 185.0369. Found $[M+ Na]^+$: 185.09415.

Ethyl (*Z*)-4-benzylhex-4-enoate

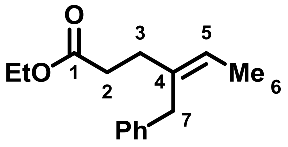

General procedure F: The preceding allylic alcohol (2.43 g, 15.0 mmol) was employed, FCC (eluent 29:1 hexane:EtOAc) afforded title compound (2.98 g, 86 %) as a colorless oil.

ν_{max} / cm^{-1}: (film) 3027 (m), 2980 (m), 2920 (m), 1732 (s), 1602 (m), 1494 (m), 1452 (s), 1164 (s).

δ_H (400 MHz, $CDCl_3$) 7.30 – 7.24 (2H, m, ArCH), 7.21 – 7.13 (3H, m, ArCH), 5.45 (1H, q, *J* = 6.5 Hz, C**5**-H), 4.09 (2H, q, *J* = 7.0 Hz, OCH_2CH_3), 3.42 (2H, s, C**7**-H_2), 2.39 – 2.31 (2H, m, C**2**-H_2), 2.29 – 2.23 (2H, m, C**3**-H_2), 1.72 (3H, d, *J* = 6.5 Hz, C**6**-H_3), 1.22 (3H, t, *J* = 7.0 Hz, OCH_2CH_3).

δ_C (101 MHz, $CDCl_3$) 173.5 (C**1**), 140.0 (ArC), 137.0 (C**4**), 128.6 (ArCH), 128.5 (ArCH), 126.1 (ArCH), 121.1 (C**5**), 60.3 (OCH_2CH_3), 35.8 (C**7**), 33.2 (C**2**), 31.9 (C**3**), 14.4 (OCH_2CH_3), 13.8 (C**6**).

HRMS: (ESI^+) Calculated for $C_{15}H_{20}NaO_2$: 255.1356. Found $[M+Na]^+$: 255.1354.

(*Z*)-4-Benzylhex-en-1-ol

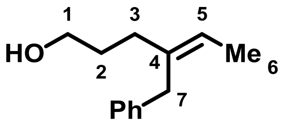

General procedure D: The preceding ester (1.50 g, 6.46 mmol) was employed, using anhydrous THF as the solvent and 0.8 eq. $LiAlH_4$ (1M in THF). The crude mixture was purified by FCC (eluent 4:1 hexane:EtOAc) afforded the title compound (1.12 g, 91 %) as a colorless oil.

ν_{max} / cm^{-1}: (film) 3330 (br s), 3026 (m), 2929 (m), 1601 (m), 1452 (m), 1055 (s).

δ_H (400 MHz, $CDCl_3$) 7.33 – 7.24 (2H, m, ArCH), 7.22 – 7.12 (3H, m, ArCH), 5.46 (1H, q, *J* = 7.0 Hz, C**5**-H), 3.58 (2H, t, *J* = 6.5 Hz, C**1**-H_2), 3.42 (2H, s, C**7**-H_2), 2.02 – 1.97 (2H, m, C**3**-H_2), 1.73 (3H, d, *J* = 7.0 Hz, C**6**-H_3), 1.68 – 1.60 (2H, m, C**2**-H_2), 1.35 (1H, s, OH).

δ_C (101 MHz, $CDCl_3$) 140.3 (ArC), 138.2 (C**4**), 128.6 (ArCH), 128.5 (ArCH), 126.0 (ArCH), 120.7 (C**5**), 62.9 (C**1**), 35.7 (C**7**), 32.9 (C**3**), 31.0 (C**2**), 13.8 (C**6**).

HRMS: (ESI^+) Calculated for $C_{13}H_{18}NaO$: 213.1250. Found $[M+Na]^+$: 213.1248.

(*Z*)-*N*-4-Benzylhex-en-1-yl-*N*-(pentafluorobenzoyloxy)-methanesulfonamide (7h)

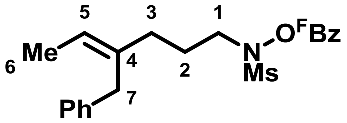

General procedure B: The preceding alcohol (90.4 mg, 0.475 mmol) and **4b** were employed. FCC (eluents 250:1 hexane:EtOAc followed by PhMe) afforded **7h** (124 mg, 55 %) as a crystalline colorless solid.

m.p. 82-83 °C (Et_2O:hexane, *needles*)

ν_{max} / cm^{-1}: (solid) 3028 (m), 2934 (m), 1780 (s), 1655 (m), 1597 (m), 1499 (s), 1169 (s).

δ_H (400 MHz, $CDCl_3$) 7.27 – 7.22 (2H, m, ArC<u>H</u>), 7.18 – 7.13 (3H, m, ArC<u>H</u>), 5.48 (1H, q, *J* = 7.0 Hz, C**5**-<u>H</u>), 3.43 – 3.33 (4H, m, C**1**-$\underline{H}_2$ and C**7**-$\underline{H}_2$), 3.00 (3H, s, Ms C$\underline{H}_3$), 2.10 (2H, t, *J* = 7.0 Hz, C**3**-$\underline{H}_2$), 1.77 – 1.68 (5H, m, C**2**-$\underline{H}_2$ and C**6**-$\underline{H}_3$).

δ_C (101 MHz, $CDCl_3$) 140.0 (Ar<u>C</u>), 136.8 (C**4**), 128.6 (Ar<u>C</u>H), 128.5 (Ar<u>C</u>H), 126.0 (Ar<u>C</u>H), 122.0 (C**5**), 52.2 (C**1**), 35.7 (C**7**), 34.5 (Ms $\underline{C}H_3$), 33.5 (C**3**), 25.2 (C**2**), 13.9 (C**6**).

The signals corresponding to the pentafluorobenzoyl group could not be resolved due to their weak intensity.

δ_F (377 MHz, $CDCl_3$) -135.7 – -135.9 (2F, m), -145.3 (1F, tt, *J* = 20.5, 5.5 Hz), -158.7 – -158.9 (2F, m).

HRMS: (ESI^+) Calculated for $C_{21}H_{20}F_5NNaO_4S$: 500.0925. Found $[M+Na]^+$: 500.0919.

(*E*)-4-Phenylbut-3-en-2-ol

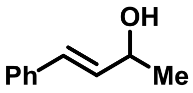

To a solution of cinnamaldehyde (10.0 g, 75.6 mmol) in anhydrous Et_2O (*approx.* 250 mL) at 0 °C was added MeLi (1.6 M in Et_2O, 56.7 mL, 90.7 mmol). The reaction mixture was stirred for 2 hours at room temperature before addition of water (10 mL) followed by saturated aqueous NH_4Cl (150 mL). The resulting phases were separated and the aqueous phase extracted with Et_2O (2 × 100 mL), the organic phases were dried over Na_2SO_4 and concentrated *in vacuo*. FCC (eluent 4:1 hexane:EtOAc) afforded the title compound (8.87 g, 79 %) as a yellow oil.

The spectroscopic properties were consistent with the data available in the literature.[15]

Ethyl (*E*)-3-phenylhex-4-enoate

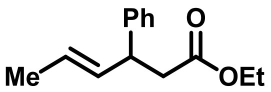

<u>**General procedure F:**</u> The preceding allylic alcohol (2.00 g, 13.5 mmol) was employed. FCC (gradient elution 49:1 – 9:1 hexane:EtOAc) afforded the title compound (1.75 g, 59 %) as a colorless oil.

The spectroscopic properties were consistent with the data available in the literature.[16]

(*E*)-3-Phenylhex-4-en-1-ol

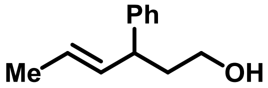

General procedure D: The preceding ester (1.67 g, 7.65 mmol) was employed, using anhydrous THF as the solvent and 1.2 eq. $LiAlH_4$ (1M in THF). The crude mixture was purified by FCC (gradient elution 7:1 – 4:1 hexane:EtOAc) to afford the title compound (480 mg, 36 %) as a colorless oil.

δ_H (400 MHz, $CDCl_3$) 7.33 – 7.28 (2H, m), 7.23 – 7.17 (3H, m), 5.60 (1H, ddq, *J* = 15.0, 8.0, 1.5 Hz), 5.56 – 5.46 (1H, m), 3.67 – 3.57 (2H, m), 3.41 (1H, br dt, *J* = 8.0 Hz), 2.00 – 1.90 (2H, m), 1.68 (3H, ddd, *J* = 5.5, 1.5, 1.0 Hz), 1.42 (1H, br s).

δ_C (101 MHz, $CDCl_3$) 144.8, 134.8, 128.7, 127.6, 126.3, 125.3, 61.3, 45.6, 38.8, 18.1.

The spectroscopic properties were consistent with the data available in the literature.[17]

(*E*)-*N*-3-Phenylhex-4-en-1-yl-*N*-(pentafluorobenzoyloxy)-4-toluenesulfonamide (7i)

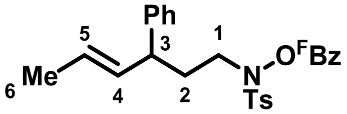

General procedure B: The preceding alcohol (23.3 mg, 0.132 mmol) and **4a** were employed. FCC (eluents 250:1 hexane:EtOAc followed by PhMe) afforded **7i** (58.7 mg, 83 %) as a crystalline colorless solid.

m.p. 121-122 °C (Et_2O:hexane, *cubes*)

ν_{max} / cm^{-1}: (solid) 2907 (m), 1780 (s), 1656 (m), 1596 (m), 1505 (s), 1171 (s).

δ_H (400 MHz, $CDCl_3$) 7.76 (2H, d, *J* = 8.0 Hz, Ts ArCH), 7.36 (2H, d, *J* = 8.0 Hz, Ts ArCH), 7.32 – 7.25 (2H, m, ArCH), 7.21 – 7.15 (3H, m, ArCH), 5.59 – 5.46 (2H, m, C**4**-H and C**5**-H), 3.53 (1H, dt, *J* = 7.0, 6.0 Hz, C**3**-H), 3.18 (2H, br s, C**1**-H_2), 2.46 (3H, s, Ts CH_3), 1.89 (2H, dt, *J* = 7.0, 7.0 Hz, C**2**-H_2), 1.66 (3H, d, *J* = 4.5 Hz, C**6**-H_3).

δ_C (101 MHz, $CDCl_3$) 146.0 (Ts ArC), 144.0 (ArC), 133.3 (C**4** or C**5**), 130.2 (Ts ArC), 130.0 (Ts ArCH), 129.8 (Ts ArCH), 128.8 (ArCH), 127.6 (ArCH), 2 x 126.5 (ArCH and C**4** or C**5**), 50.9 (C**1**), 45.6 (C**3**), 33.0 (C**2**), 21.9 (Ts CH_3), 18.1 (C**6**).

The signals corresponding to the pentafluorobenzoyl group could not be resolved due to their weak intensity.

δ_F (283 MHz, $CDCl_3$) -135.7 – -136.0 (2F, m), -146.0 (1F, tt, *J* = 21.0, 5.0 Hz), -158.8 – -159.2 (2F, m).

HRMS: (ESI^+) Calculated for $C_{26}H_{22}F_5NNaO_4S$: 562.1082. Found $[M+Na]^+$: 562.1077.

(*E*)-Hex-4-enal

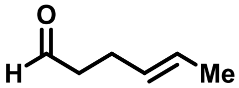

This compound was prepared according to a literature procedure.[18]

The spectroscopic properties were consistent with the data available in the literature.[18]

(*E*)-1-Cyclopropylhex-4-en-1-ol

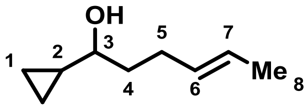

To a suspension of Mg turnings (413 mg, 17.0 mmol) activated with a few crystals of iodine in anhydrous Et_2O (15 mL) was added bromocyclopropane (1.28 mL, 16.0 mmol). The reaction mixture was heated to reflux and diluted with more Et_2O (20 mL). After 2 hours the reaction mixture was cooled to room temperature before addition of a solution of (*E*)-hex-4-enal (981 mg, 10.0 mmol) in anhydrous Et_2O (10 mL). The reaction mixture was heated to reflux overnight before addition of saturated aqueous NH_4Cl (30 mL). The phases were separated and the aqueous phase extracted with Et_2O (2 × 30 mL), the organic phases were dried over Na_2SO_4 and concentrated *in vacuo*, FCC (gradient elution, 5:1 – 3:1 pentane:Et_2O) afforded the title compound (993 mg, 71 %) as a pale yellow oil.

ν_{max} / cm^{-1}: (film) 3362 (m), 3080 (m), 2924 (s), 1435 (s), 1042 (s).

δ_H (400 MHz, $CDCl_3$) 5.51 – 5.38 (2H, m, C**6**-H and C**7**-H), 2.91 – 2.82 (1H, m, C**3**-H), 2.22 – 2.04 (2H, m, C**5**-H_2), 1.69 – 1.61 (5H, m, C**4**-H_2 and C**8**-H_3), 1.56 (1H, br s, OH), 0.89 (1H, dtt, *J* = 8.5, 8.5, 5.0 Hz, C**2**-H), 0.56 – 0.45 (2H, m, C**1**-H), 0.31 – 0.16 (2H, m, C**1**-H').

δ_C (101 MHz, $CDCl_3$) 131.2 (C**6** or C**7**), 125.3 (C**6** or C**7**), 76.5 (C**3**), 37.1 (C**4**), 29.0 (C**5**), 18.1 (2 signals, C**2** and C**8**), 2.8 (C**1**), 2.6 (C**1**').

HRMS: (EI^+) Calculated for C_9H_{14}: 122.1096. Found $[M-H_2O]^+$: 122.1100.

(*E*)-*N*-1-Cyclopropylhex-4-en-1-yl-*N*-(pentafluorobenzoyloxy)-4-toluenesulfonamide (7j)

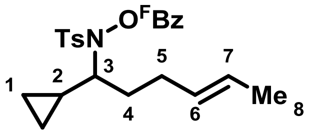

General procedure B: The preceding alcohol (66.6 mg, 0.475 mmol) and **4a** were employed. FCC (eluents 250:1 hexane:EtOAc followed by PhMe) afforded **7j** (203 mg, 85 %) as a colorless oil.

ν_{max} / cm^{-1}: (film) 2929 (m), 1786 (s), 1653 (m), 1598 (m), 1498 (s), 1325 (s), 1165 (s).

δ_H (500 MHz, $CDCl_3$) 7.85 (2H, d, *J* = 8.0 Hz, ArCH), 7.35 (2H, d, *J* = 8.0 Hz, ArCH), 5.53 – 5.43 (1H, m, C**7**-H), 5.42 – 5.32 (1H, m, C**6**-H), 3.53 – 3.33 (1H, m, C**3**-H), 2.47 (3H, s, Ts CH_3), 2.38 – 2.14 (2H, m, C**5**-H_2), 1.80 – 1.64 (5H, m, C**4**-H_2 and C**8**-H_3), 1.05 (1H, br s, C**2**-H), 0.67 (1H, br s, C**1**-H), 0.63 – 0.54 (2H, m, C**1**-H' and C**1**'-H'), 0.34 (1H, br s, C**1**'-H).

δ_C (126 MHz, $CDCl_3$) 156.4 (^{F}Bz C=O), 145.6 (ArC), 133.5 (ArC), 130.2 (C**6**), 129.8 (ArCH), 129.5 (ArCH), 126.1 (C**7**), 67.3 (C**3**), 32.8 (C**4**), 29.4 (C**5**), 21.9 (Ts CH_3), 18.1 (C**8**), 13.0 (C**2**), 5.8 (2 signals, C**1** and C**1**').

The aromatic signals corresponding to the pentafluorobenzoyl group could not be resolved due to their weak intensity.

δ_F (377 MHz, $CDCl_3$) -136.3 – -136.5 (2F, m), -146.3 (1F, tt, *J* = 21.0, 5.0 Hz), -158.9 – -159.1 (2F, m).

HRMS: (ESI^+) Calculated for $C_{23}H_{22}F_5NNaO_4S$: 526.1082. Found $[M+Na]^+$: 526.1087.

(*E*)-1-Phenylhex-4-ene-1-one

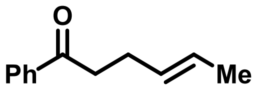

To a suspension of NaH (60 % weight in mineral oil, 1.12 g, 28.0 mmol) in anhydrous THF (*approx.* 60 mL) at 0 °C was added ethyl benzoylacetate (4.85 mL, 28.0 mmol) dropwise. The reaction mixture was stirred at room temperature for 1 hour before dropwise addition of crotyl bromide (2.06 mL, 20.0 mmol). After 16 hours the reaction mixture was concentrated *in vacuo* and the mono- and bis-alkylated products were separated by FCC (gradient elution, 24:1 – 19:1 hexane:EtOAc) to afford a mixture of mono-alkylated dicarbonyl and unconsumed ethyl benzoylacetate (3.48 g). This mixture was hydrolyzed in a solution of KOH (4.1 g, 78 mmol) in water:MeOH (1:1, 40 mL) for 6 hours before addition of 10 M aqueous HCl (16 mL, 160 mmol). The reaction mixture was concentrated to an aqueous solution before being extracted with EtOAc (3 x 60 mL). The organic phases were dried over Na_2SO_4, concentrated *in vacuo* and the crude mixture was purified by FCC (eluent 40:1 hexane:EtOAc) to afford the title compound (1.15 g, 33 %) as a colorless oil.

The spectroscopic properties were consistent with the data available in the literature.[19]

(*E*)-1-Phenylhex-4-ene-1-ol

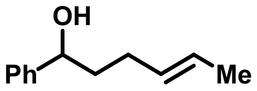

To a solution of preceding ketone (741 mg, 4.25 mmol) in MeOH (30 mL) at 0 °C was added $NaBH_4$ (241 mg, 6.38 mmol), the reaction mixture was stirred at this temperature for 1 hour before addition of water (20 mL) and extraction with DCM (3 x 30 mL). The DCM extracts were dried over Na_2SO_4, concentrated *in vacuo* and filtered through a short plug of silica. The filtrate was concentrated *in vacuo* to afford the title compound (708 mg, 95 %) as a colorless oil.

δ_H (400 MHz, $CDCl_3$) 7.37 – 7.32 (4H, m), 7.31 – 7.24 (1H, m), 5.52 – 5.39 (2H, m), 4.68 (1H, ddd, *J* = 7.5, 5.5, 3.5 Hz), 2.17 – 1.99 (2H, m), 1.98 – 1.94 (m, 1H), 1.91 – 1.71 (2H, m), 1.67 – 1.63 (3H, m).

δ_C (101 MHz, $CDCl_3$) 144.8, 130.7, 128.6, 127.6, 126.0, 125.7, 74.2, 38.9, 29.1, 18.1.

HRMS: (ESI^+) Calculated for $C_{12}H_{16}NaO$: 199.1093. Found $[M+Na]^+$: 199.1087.

The spectroscopic properties were consistent with the data available in the literature.[20]

(*E*)-*N*-1-Phenylhex-4-ene-1-yl-*N*-(pentafluorobenzoyloxy)-methanesulfonamide (7k)

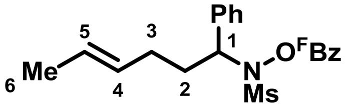

General procedure B: The preceding alcohol (12 mg, 0.068 mmol) and **4b** were employed. FCC (eluents 250:1 hexane:EtOAc followed by PhMe) afforded **7k** (33.9 mg, 62 %) as a crystalline colorless solid.

ν_{max} / cm^{-1}: (solid) 3030 (m), 2945 (m), 1775 (s), 1651 (m), 1502 (s), 1367 (s), 1165 (s).

δ_H (500 MHz, $CDCl_3$) 7.38 (5H, s, ArC<u>H</u>), 5.45 – 5.32 (2H, m, C**4**-<u>H</u> and C**5**-<u>H</u>), 5.02 (1H, dd, *J* = 8.5, 6.0 Hz, C**1**-<u>H</u>), 2.38 – 2.18 (4H, m, C**2**-<u>H</u> and Ms C<u>H</u>$_3$), 2.07 – 1.88 (3H, m, C**2**-<u>H</u>' and C**3**-<u>H</u>$_2$), 1.64 (3H, d, *J* = 5.0 Hz, C**6**-<u>H</u>$_3$).

δ_C (126 MHz, $CDCl_3$) 156.6 (^{F}Bz <u>C</u>=O), 135.8 (Ar<u>C</u>), 129.4 (Ar<u>C</u>H or C**4**), 129.3 (Ar<u>C</u>H or C**4**), 129.2 (Ar<u>C</u>H or C**4**), 129.0 (Ar<u>C</u>H or C**4**), 127.0 (C**5**), 66.0 (C**1**), 36.8 (Ms <u>C</u>H$_3$), 32.8 (C**2**), 29.0 (C**3**), 18.1 (C**6**).

The aromatic signals corresponding to the pentafluorobenzoyl group could not be resolved due to their weak intensity.

δ_F (283 MHz, $CDCl_3$) -135.5 – -135.7 (2F, m), -144.9 – -145.2 (1F, m), -158.4 – -158.7 (2F, m).

HRMS: (ESI^+) Calculated for $C_{20}H_{18}F_5NNaO_4S$: 486.0769. Found $[M+Na]^+$: 486.0756.

(*E*)-Dec-8-en-5-ol

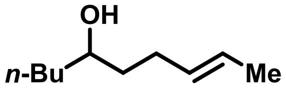

To a solution of *n*-BuLi (1.55 M in hexane, 10.0 mL, 15.5 mmol) in anhydrous THF (30 mL) at 0 °C was added a solution of (*E*)-hex-4-enal (1.40 g, 14.3 mmol) in anhydrous THF (5 mL) dropwise. The

reaction mixture was stirred for 1 hour before addition of saturated aqueous NH_4Cl (25 mL), the resulting phases were separated and the aqueous phase was extracted with Et_2O (2 x 40 mL). The organic phases were dried over Na_2SO_4 and concentrated *in vacuo* to afford the title compound (1.75 g, 78 %) as a pale yellow oil.

ν_{max} / cm^{-1}: (film) 3344 (br s), 2930 (s), 2858 (s), 1482 (s), 964 (s).

δ_H (400 MHz, $CDCl_3$) 5.52 – 5.40 (2H, m), 3.65 – 3.57 (1H, m), 2.21 – 2.01 (2H, m), 1.65 (3H, d, *J* = 4.5 Hz), 1.59 – 1.24 (9H, m), 0.91 (3H, t, *J* = 7.0 Hz).

δ_C (101 MHz, $CDCl_3$) 131.1, 125.3, 71.6, 37.2, 37.1, 28.9, 27.8, 22.7, 17.9, 14.1.

The spectroscopic properties were consistent with the data available in the literature.[21]

(*E*)-*N*-Dec-8-en-5-yl-*N*-(pentafluorobenzoyloxy)-methanesulfonamide (7l)

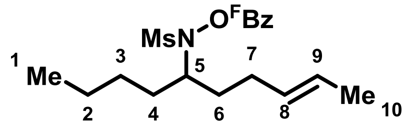

General procedure B: The preceding alcohol (74.2 mg, 0.475 mmol) and **4b** were employed. FCC (eluents 250:1 hexane:EtOAc followed by PhMe) afforded **7l** (156 mg, 65 %) as a crystalline colorless solid.

ν_{max} / cm^{-1}: (solid) 2944 (m), 1784 (s), 1651 (m), 1496 (s), 1160 (s).

δ_H (400 MHz, $CDCl_3$) 5.54 – 5.44 (1H, m, C**9**-H), 5.43 – 5.34 (1H, m, C**8**-H), 3.98 (1H, tt, *J* = 6.5, 6.5 Hz, C**5**-H), 3.09 (3H, s, Ms CH_3), 2.30 – 2.07 (2H, m, C**7**-H_2), 1.75 – 1.23 (11H, m, C**2**-H_2, C**3**-H_2, C**4**-H_2, C**6**-H_2 and C**10**-H_3), 0.91 (3H, t, *J* = 7.0 Hz, C**1**-H_3).

δ_C (101 MHz, $CDCl_3$) 129.9 (C**8**), 126.4 (C**9**), 62.3 (C**5**), 40.0 (Ms CH_3), 31.8 (C**2**, C**3**, C**4** or C**6**), 31.5 (C**2**, C**3**, C**4** or C**6**), 29.5 (C**7**), 28.7 (C**2**, C**3**, C**4** or C**6**), 22.6 (C**2**, C**3**, C**4** or C**6**), 18.1 (C**10**), 14.0 (C**1**).

The signals corresponding to the pentafluorobenzoyl group could not be resolved due to their weak intensity.

δ_F (377 MHz, $CDCl_3$) -135.9 – -136.1 (2F, m), -145.4 (1F, tt, *J* = 21.0, 5.5 Hz), -158.6 – -158.8 (2F, m).

HRMS: (ESI^+) Calculated for $C_{18}H_{22}F_5NNaO_4S$: 466.1082. Found $[M+ Na]^+$: 466.1070.

Methyl 3-cyclopropyl-3-oxopropanoate

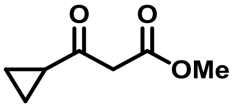

This compound was prepared according to a literature procedure.[10]

The spectroscopic properties were consistent with the data available in the literature.[10]

Methyl (*E*)-2-(cyclopropanecarbonyl)-4-methylhex-4-enoate

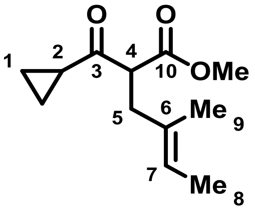

To a suspension of NaH (60 % weight in mineral oil, 720 mg, 18.0 mmol) in anhydrous DMF (35 mL) was added the preceding β-keto ester (2.31 mL, 18.0 mmol) dropwise. The reaction mixture was stirred at room temperature for 1 hour before addition of (*E*)-1-bromo-2-methylbut-2-ene (1.42 mL, 12.0 mmol) and heated to 80 °C overnight. The reaction mixture was cooled to room temperature before addition of saturated aqueous NH_4Cl (50 mL) and extraction with Et_2O (3 × 50 mL). The organic phases were dried over Na_2SO_4 and concentrated *in vacuo*, the crude mixture was purified by FCC (gradient elution 19:1 – 14:1 hexane:EtOAc) to afford the title compound (2.01 g, 80 %) as a colorless oil.

ν_{max} / cm^{-1}: (film) 2954 (m), 1740 (s), 1701 (s), 1436 (s), 1382 (s), 1160 (s).

δ_H (400 MHz, $CDCl_3$) 5.26 (1H, qq, *J* = 7.0, 1.5 Hz, C**7**-H), 3.77 (1H, t, *J* = 7.5 Hz, C**4**-H), 3.72 (3H, s, OCH_3), 2.61 – 2.54 (2H, m, C**5**-H_2), 2.05 (1H, tt, *J* = 8.0, 4.5 Hz, C**2**-H), 1.61 (3H, br s, C**9**-H_3), 1.55 (3H, dq, *J* = 7.0, 1.0 Hz, C**8**-H_3), 1.08 – 1.03 (2H, m, C**1**-H), 0.95 – 0.88 (2H, m, C**1**-H').

δ_C (101 MHz, $CDCl_3$) 205.1 (C**3**), 170.2 (C**10**), 131.6 (C**6**), 121.4 (C**7**), 58.6 (C**4**), 52.3 (OCH_3), 38.0 (C**5**), 19.7 (C**2**), 15.5 (C**9**), 13.5 (C**8**), 11.8 (C**1**), 11.6 (C**1**').

HRMS: (ESI^+) Calculated for $C_{12}H_{18}NaO_3$: 233.1148. Found $[M+Na]^+$: 233.1151.

(*E*)-1-Cyclopropyl-4-methylhex-4-en-1-ol

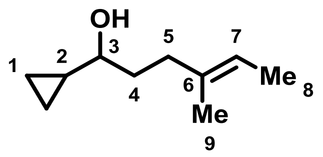

To a solution of the preceding β-keto ester (1.94 g, 9.23 mmol) in MeOH:water (5:3, 40 mL) was added KOH (2.07 g, 36.9 mmol). The reaction mixture was stirred at room temperature for 40 minutes before addition of 2 M aqueous HCl (25 mL) and extraction with Et_2O (3 × 70 mL). The organic phases were dried over Na_2SO_4 and concentrated *in vacuo*. The crude mixture was dissolved in EtOAc (30 mL) and heated to reflux for 3 hours before being concentrated *in vacuo*. The resulting oil was dissolved in MeOH (20 mL) and cooled to 0 °C before addition of $NaBH_4$ (349 mg, 9.23 mmol). The reaction mixture was stirred at this temperature for 2 hours before addition of 1 M aqueous HCl (20 mL) and extraction with Et_2O (3 × 40 mL). The organic phases were dried over Na_2SO_4 and concentrated *in vacuo*, the crude mixture was purified by FCC (eluent 5:2 pentane:Et_2O) to afford the title compound (688 mg, 48 %) as a colorless oil.

ν_{max} / cm^{-1}: (film) 3360 (br s), 3080 (m), 2918 (m), 1432 (m), 1019 (s).

δ_H (400 MHz, $CDCl_3$) 5.24 (1H, qq, *J* = 6.5, 1.0 Hz, C**7**-H), 2.84 (1H, ddd, *J* = 8.5, 7.5, 5.0 Hz, C**3**-H), 2.20 – 2.00 (2H, m, C**5**-H_2), 1.73 – 1.65 (2H, m, C**4**-H_2), 1.63 (1H, br s, OH), 1.60 (3H, br s, C**9**-H_3), 1.56 (3H, dq, *J* = 6.5, 1.0 Hz, C**8**-H_3), 0.89 (1H, dtt, *J* = 8.5, 8.5, 5.0 Hz, C**2**-H), 0.55 – 0.44 (2H, m, C**1**-H), 0.29 – 0.16 (2H, m, C**1**-H').

δ_C (101 MHz, $CDCl_3$) 135.7 (C**6**), 118.51 (C**7**), 76.6 (C**3**), 35.8 (C**4**), 35.3 (C**5**), 17.9 (C**9**), 15.6 (C**8**), 13.3 (C**2**), 2.7 (C**1**), 2.5 (C**1**').

HRMS: (ESI^+) Calculated for $C_{10}H_{18}NaO$: 177.1250. Found $[M+Na]^+$: 177.1246.

(*E*)-*N*-(1-Cyclopropyl-4-methylhex-4-en-1-yl)-*N*-(pentafluorobenzoyloxy)-4-toluenesulfonamide (7m)

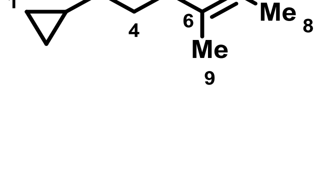

General procedure B: The preceding alcohol (540 mg, 3.50 mmol) and **4a** were employed. FCC (gradient elution, 49:1 – 24:1 hexane:EtOAc) afforded **7m** (1.09 g, 60 %) as a pale yellow oil.

ν_{max} / cm^{-1}: (film) 2926 (m), 1786 (s), 1653 (m), 1598 (m), 1497 (s), 1164 (s).

δ_H (500 MHz, $CDCl_3$) 7.86 (2H, d, *J* = 8.0 Hz, ArCH), 7.35 (2H, d, *J* = 8.0 Hz, ArCH), 5.25 (1H, br s, C**7**-H), 3.50 – 3.28 (1H, m, C**3**-H), 2.47 (3H, s, Ts CH_3), 2.39 – 2.13 (2H, m, C**5**-H_2), 1.85 – 1.69 (2H, m, C**4**-H_2), 1.63 – 1.54 (6H, m, C**8**-H_3 and C**9**-H_3), 1.18 – 0.95 (1H, m, C**2**-H), 0.72 – 0.55 (3H, m, C**1**-H and C**1'**-H_2), 0.33 (1H, br s, C**1**-H').

δ_C (126 MHz, $CDCl_3$) 156.2 (^{F}Bz C=O), 145.4 (ArC), 134.7 (C**6**), 133.4 (ArC), 129.6 (ArCH), 129.3 (ArCH), 119.3 (C**7**), 67.4 (C**3**), 36.3 (C**5**), 31.2 (C**4**), 21.7 (Ts CH_3), 15.5 (C**9**), 13.4 (C**8**), 12.8 (C**2**), 5.7 (C**1'**), 4.8 (C**1**).

The aromatic signals corresponding to the pentafluorobenzoyl group could not be resolved due to their weak intensity.

δ_F (377 MHz, $CDCl_3$) -136.3 – -136.5 (2F, m), -146.4 (1F, tt, *J* = 21.0, 5.0 Hz), -158.9 – -159.1 (2F, m).

HRMS: (ESI^+) Calculated for $C_{24}H_{24}F_5NNaO_4S$: 540.1238. Found $[M+Na]^+$: 540.1232.

Methyl (*E*)-6-hydroxyhex-2-enoate

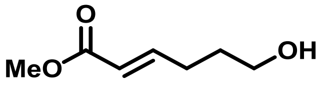

To a solution of Hoveyda-Grubbs 2nd generation catalyst (15.7 mg, 25.0 μmol) in anhydrous, degassed DCM (40 mL) was added methyl acrylate (2.25 mL, 25.0 mmol) and pent-4-en-1-ol (0.26 mL, 2.50 mmol). The reaction mixture was heated to reflux overnight before being concentrated *in vacuo*. The crude mixture was purified by FCC (eluent 2:1 hexane:EtOAc) to afford the title compound (357 mg, 99 %) as a light brown oil (the coloration was due to the presence of trace amounts of Ru-impurities).

ν_{max} / cm^{-1}: (film) 3417 (br s), 2950 (m), 1720 (s), 1656 (s), 1436 (s), 1272 (s).

δ_H (400 MHz, $CDCl_3$) 6.98 (1H, dt, *J* = 15.5, 7.0 Hz), 5.85 (1H, dt, *J* = 15.5, 1.5 Hz), 3.72 (3H, s), 3.67 (2H, t, *J* = 6.5 Hz), 2.30 (2H, dtd, *J* = 7.0, 7.0, 1.5 Hz), 1.77 – 1.68 (2H, m), 1.51 (1H, br s).

δ_C (101 MHz, $CDCl_3$) 167.2, 148.9, 121.4, 62.1, 51.6, 31.0, 28.7.

The spectroscopic properties were consistent with the data available in the literature.[22]

Methyl (*E*)-6-((*N*-(pentafluorobenzoyloxy)-4-tolyl)sulfonamido)hex-2-enoate (7n)

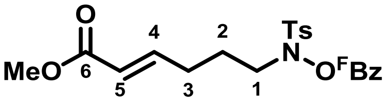

General procedure B: The preceding alcohol (115 mg, 0.800 mmol) and **4a** were employed. FCC (eluent 49:1 PhMe:EtOAc) afforded **7n** (310 mg, 76 %) as a crystalline colorless solid.

ν_{max} / cm^{-1}: (solid) 2953 (m), 1790 (s), 1706 (s), 1595 (m), 1501 (s), 1168 (s).

δ_H (400 MHz, $CDCl_3$) 7.79 (2H, d, *J* = 8.0 Hz, ArCH), 7.38 (2H, d, *J* = 8.0 Hz, ArCH), 6.90 (1H, dt, *J* = 15.5, 7.0 Hz, C**4**-H), 5.85 (1H, dt, *J* = 15.5, 1.5 Hz, C**5**-H), 3.72 (3H, s, OCH_3), 3.24 (2H, br s, C**1**-H_2), 2.47 (3H, s, Ts CH_3), 2.42 (2H, tdd, *J* = 7.5, 7.0, 1.5 Hz, C**3**-H_2), 1.72 (2H, tt, *J* = 7.5, 7.0 Hz, C**2**-H_2).

δ_C (101 MHz, $CDCl_3$) 166.8 (C**6**), 147.2 (C**4**), 146.0 (ArC), 129.9 (2 signals, ArC and ArCH), 129.6 (ArCH), 122.1 (C**5**), 51.8 (C**1**), 51.5 (OCH_3), 28.8 (C**3**), 25.0 (C**2**), 21.7 (Ts CH_3).

The signals corresponding to the pentafluorobenzoyl group could not be resolved due to their weak intensity.

δ_F (377 MHz, $CDCl_3$) -135.8 – -136.0 (2F, m), -145.7 (1F, tt, *J* = 20.5, 5.0 Hz), -158.7 – -159.0 (2F, m).

HRMS: (ESI^+) Calculated for $C_{21}H_{18}F_5NNaO_6S$: 530.0667. Found $[M+Na]^+$: 530.0662.

Nona-1,8-dien-5-ol

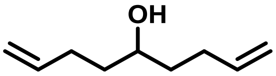

This compound was prepared according to a literature procedure.[23]

ν_{max} / cm^{-1}: (film) 3345 (br s), 3078 (m), 2931 (m), 2852 (m), 1641 (m), 1449 (m).

δ_H (400 MHz, $CDCl_3$) 5.83 (2H, ddt, *J* = 17.0, 10.0, 6.5 Hz), 5.04 (2H, ddt, *J* = 17.0, 1.5, 1.5 Hz), 4.96 (2H, ddt, *J* = 10.0, 1.5, 1.5 Hz), 3.65 (1H, tt, *J* = 7.5, 4.5 Hz), 2.25 – 2.06 (4H, m), 1.91 (1H, s), 1.62 – 1.46 (4H, m).

δ_C (101 MHz, $CDCl_3$) 138.6, 114.8, 71.0, 36.5, 30.1.

The spectroscopic properties were consistent with the data available in the literature.[23]

_N_-(Nona-1,8-dien-5-yl)-_N_-(pentafluorobenzoyloxy)methanesulfonamide

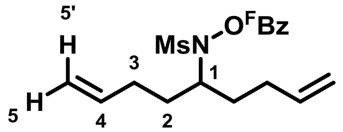

General procedure B: The preceding alcohol (28.0 mg, 0.200 mmol) and **4b** were employed. FCC (eluents 250:1 hexane:EtOAc followed by PhMe) afforded the title compound (121 mg, 63 %) as a crystalline colorless solid.

ν_{max} / cm^{-1}: (film) 2939 (m), 1783 (s), 1653 (m), 1499 (s) 1325 (s), 1161 (s).

δ_H (400 MHz, $CDCl_3$) 5.78 (2H, ddt, *J* = 17.0, 10.0, 6.5 Hz, C**4**-H), 5.06 (1h, ddt, *J* = 17.0, 1.5, 1.5 Hz, C**5**-H'), 5.03 – 4.98 (2H, m, C**5**-H), 4.03 (1H, tt, *J* = 6.5, 6.5 Hz, C**1**-H), 3.09 (3H, s, Ms CH_3), 2.35 – 2.12 (4H, m, C**3**-H_2), 1.80 – 1.61 (4H, m, C**2**-H_2).

δ_C (101 MHz, $CDCl_3$) 157.2 (^{F}Bz C=O), 137.2 (C**4**), 115.7 (C**5**), 61.2 (C**1**), 39.9 (Ms CH_3), 30.8 (C**2**), 30.4 (C**3**).

The aromatic signals corresponding to the pentafluorobenzoyl group could not be resolved due to their weak intensity.

δ_F (377 MHz, $CDCl_3$) -135.9 – -136.1 (2F, m), -145.3 (1F, tt, *J* = 21.0, 5.5 Hz), -158.6 – -158.8 (2F, m).

HRMS: (ESI^+) Calculated for $C_{17}H_{18}F_5NNaO_4S$: 450.0769. Found $[M+ Na]^+$: 450.0749.

***N*-(Cyclohept-4-en-1-yl)-*N*-(pentafluorobenzoyloxy)methanesulfonamide (7o)**

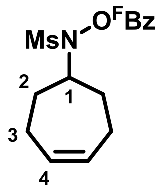

To a solution of the preceding compound (63.2 mg, 0.148 mmol) in anhydrous, degassed DCM (80 mL) was added Hoveyda-Grubbs 2nd generation catalyst (2.0 mg, 3.2 μmol). The reaction mixture was heated to reflux overnight before being concentrated *in vacuo*. The crude mixture was purified by FCC (eluent 1:1 PhMe:hexane) to afford **7o** (51.7 mg, 87 %) as a colorless crystalline solid.

ν_{max} / cm^{-1}: (solid) 2939 (m), 2845 (m), 1768 (s), 1651 (s), 1591 (s), 1157 (s).

δ_H (500 MHz, $CDCl_3$) 5.83 – 5.79 (2H, m, C**4**-H), 4.20 (1H, tt, *J* = 10.5, 3.5 Hz, C**1**-H), 3.09 (3H, s, Ms C$\underline{H}_3$), 2.32 – 2.24 (2H, m, C**3**-H), 2.21 – 2.14 (2H, m, C**2**-H), 2.12 – 2.04 (2H, m, C**3**-H'), 1.69 – 1.59 (2H, m, C**2**-H').

δ_C (126 MHz, $CDCl_3$) 157.3 (^{F}Bz C=O), 131.6 (C**4**), 65.6 (C**1**), 39.7 (Ms $\underline{C}H_3$), 31.0 (C**3**), 24.8 (C**2**).

The aromatic signals corresponding to the pentafluorobenzoyl group could not be resolved due to their weak intensity.

δ_F (377 MHz, $CDCl_3$) -135.8 – -136.0 (2F, m), -145.2 (1F, tt, *J* = 21.0, 5.5 Hz), -158.5 – -158.7 (2F, m).

HRMS: (ESI^+) Calculated for $C_{15}H_{14}F_5NNaO_4S$: 422.0456. Found $[M+Na]^+$: 422.0459.

Cyclohex-3-en-1-ylmethanol

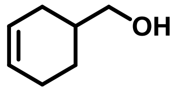

To a solution of 3-cyclohexene-1-carboxaldehyde (11.0 g, 100 mmol) in MeOH (150 mL) at 0 °C was added $NaBH_4$ (1.51 g, 40.0 mmol), the reaction mixture was stirred at this temperature for 1 hour before addition of water (100 mL) and extraction with Et_2O (3 x 150 mL). The organic phases were

dried over Na_2SO_4 and concentrated *in vacuo*, FCC (gradient elution 3:1 – 1:1 pentane:Et_2O) afforded the title compound (8.00 g, 71 %) as a pale yellow oil.

δ_H (400 MHz, $CDCl_3$) 5.70 – 5.62 (2H, m), 3.56 – 3.46 (2H, m), 2.14 – 2.02 (3H, m), 1.86 – 1.67 (4H, m), 1.32 – 1.20 (1H, m).

δ_C (101 MHz, $CDCl_3$) 127.2, 126.0, 67.9, 36.4, 28.2, 25.3, 24.7.

The spectroscopic properties were consistent with the data available in the literature.[24]

_N_-(Cyclohex-3-en-1-ylmethyl)-_N_-(pentafluorobenzoyloxy)methanesulfonamide (7p)

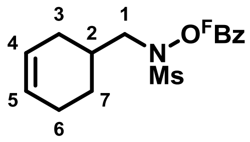

<u>General procedure B:</u> The preceding alcohol (53.3 mg, 0.475 mmol) and **4b** were employed. FCC (eluents 250:1 hexane:EtOAc followed by PhMe) afforded **7p** (100 mg, 53 %) as a crystalline colorless solid.

m.p. 109-110 °C (DCM:hexane, *needles*)

ν_{max} / cm^{-1}: (solid) 3029 (m), 2917 (m), 1782 (s), 1653 (s), 1505 (s), 1353 (s), 1162 (s).

δ_H (400 MHz, $CDCl_3$) 5.73 – 5.62 (2H, m, C**4**-<u>H</u> and C**5**-<u>H</u>), 3.45 – 3.28 (2H, m, C**1**-<u>H</u>$_2$), 3.04 (3H, s, Ms C<u>H</u>$_3$), 2.36 – 2.24 (1H, m, C**3**-<u>H</u>), 2.16 – 2.02 (2H, m, C**6**-<u>H</u>$_2$), 2.02 – 1.79 (3H, m, C**2**-<u>H</u>, C**3**-<u>H</u>’ and C**7**-<u>H</u>), 1.50 – 1.38 (1H, m, C**7**-<u>H</u>’).

δ_C (101 MHz, $CDCl_3$) 156.4 (^{F}Bz <u>C</u>=O), 127.3 (C**4** or C**5**), 125.2 (C**4** or C**5**), 57.7 (C**1**), 34.2 (Ms <u>C</u>H$_3$), 31.6 (C**2**), 29.2 (C**3**), 25.9 (C**7**), 24.1 (C**6**).

The aromatic signals corresponding to the pentafluorobenzoyl group could not be resolved due to their weak intensity.

δ_F (377 MHz, $CDCl_3$) -135.7 – -135.9 (2F, m), -145.3 (1F, tt, *J* = 21.0, 5.5 Hz), -158.8 – -159.0 (2F, m).

HRMS: (ESI^+) Calculated for $C_{15}H_{14}F_5NNaO_4S$: 422.0456. Found $[M+Na]^+$: 422.0455.

1-Bromo-2-(prop-1-en-1-yl)benzene

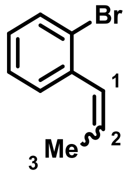

This compound was prepared according to a literature procedure.[25]

ν_{max} / cm^{-1}: (film) 3022 (m), 2913 (m), 1467 (s), 1431 (s). 1023 (s).

Spectroscopic data for the major Z isomer:

δ_H (400 MHz, $CDCl_3$) 7.61 – 7.56 (1H, m, ArCH), 7.33 – 7.21 (2H, m, ArCH), 7.14 – 7.07 (1H, m, ArCH), 6.49 (1H, dq, *J* = 11.5, 2.0 Hz, C**1**-H), 5.90 (1H, dq, *J* = 11.5, 7.0 Hz, C**2**-H), 1.79 (3H, dd, *J* = 7.0, 2.0 Hz, C**3**-H_3).

δ_C (101 MHz, $CDCl_3$) 137.5 (ArC), 132.7 (ArCH), 130.8 (ArCH), 129.5 (C**1**), 128.3 (ArCH), 128.2 (C**2**), 126.9 (ArCH), 124.2 (ArC), 14.5 (C**3**).

Spectroscopic data for the minor E isomer:

δ_H (400 MHz, $CDCl_3$) 7.54 – 7.50 (1H, m, ArCH), 7.49 – 7.45 (1H, m, ArCH), 7.32 – 7.21 (1H, m, ArCH), 7.08 – 7.02 (1H, m, ArCH), 6.74 (1H, dq, *J* = 15.5, 2.0 Hz, C**1**-H), 6.19 (1H, dq, *J* = 15.5, 6.5 Hz, C**2**-H), 1.93 (3H, dd, *J* = 6.5, 2.0 Hz, C**3**-H_3).

δ_C (101 MHz, $CDCl_3$) 137.8 (ArC), 132.9 (ArCH), 130.0 (C**1**), 129.0 (C**2**), 128.2 (ArCH), 127.5 (ArCH), 126.9 (ArCH), 123.1 (ArC), 18.8 (C**3**).

HRMS: (EI^+) Calculated for C_9H_9Br: 195.9888. Found $[M]^+$: 195.9986.

2-(2-(Prop-1-en-1-yl)phenyl)ethan-1-ol

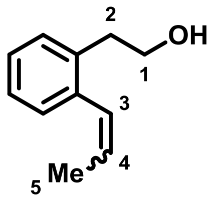

To a solution of the preceding bromide (2.96 g, 15.0 mmol) in anhydrous THF (40 mL) at -78 °C was added *n*-BuLi (1.55 M in hexane, 10.6 mL, 16.5 mmol). The reaction mixture was stirred at this temperature for 2 hours before addition ethylene oxide (*approx.* 3 M in THF, 7.5 mL, 22.5 mmol). The reaction mixture was slowly warmed to room temperature and stirred overnight before addition of saturated aqueous NH_4Cl (30 mL). The resulting phases were separated and the aqueous phase extracted with Et_2O (2 × 40 mL). The organic phases were dried over Na_2SO_4 and concentrated *in vacuo,* FCC (eluent 3:1 hexane:EtOAc) afforded the title compound (1.50 g, 62 %, 3:1 mixture of *Z* and *E* isomers) as a pale yellow oil.

ν_{max} / cm^{-1}: (film) 3322 (br s), 3017 (m), 2937 (m), 2876 (m), 1484 (s), 1446 (s), 1041 (s).

Spectroscopic data for the major Z isomer:

δ_H (400 MHz, $CDCl_3$) 7.25 – 7.15 (4H, m, ArC<u>H</u>), 6.55 (1H, dq, *J* = 11.5, 2.0 Hz, C**3**-<u>H</u>), 5.86 (1H, dq, *J* = 11.5, 7.0 Hz, C**4**-<u>H</u>), 3.78 (2H, t, *J* = 7.0 Hz, C**1**-$\underline{H}_2$), 2.88 (2H, t, *J* = 7.0 Hz, C**2**-$\underline{H}_2$), 1.73 (3H, dd, *J* = 7.0, 2.0 Hz, C**5**-$\underline{H}_3$), 1.46 (1H, br s, O<u>H</u>).

δ_C (101 MHz, $CDCl_3$) 136.9 (Ar<u>C</u>), 136.6 (Ar<u>C</u>), 130.0 (2 signals, Ar<u>C</u>H), 128.7 (C**3**), 127.8 (C**4**), 127.1 (Ar<u>C</u>H), 126.3 (Ar<u>C</u>H), 63.1 (C**1**), 36.9 (C**2**), 14.4 (C**5**).

Spectroscopic data for the minor E isomer:

δ_H (400 MHz, $CDCl_3$) 7.46 – 7.41 (1H, m, ArC<u>H</u>), 7.25 – 7.15 (3H, m, ArC<u>H</u>), 6.66 (1H, dq, *J* = 15.5, 2.0 Hz, C**3**-<u>H</u>), 6.12 (1H, dq, *J* = 15.5, 6.5 Hz, C**4**-<u>H</u>), 3.82 (2H, t, *J* = 7.0 Hz, C**1**-$\underline{H}_2$), 2.96 (2H, t, *J* = 7.0 Hz, C**2**-$\underline{H}_2$), 1.91 (3H, dd, *J* = 6.5, 2.0 Hz, C**5**-$\underline{H}_3$), 1.46 (1H, br s, O<u>H</u>).

δ_C (101 MHz, $CDCl_3$) 137.5 (Ar<u>C</u>), 135.0 (Ar<u>C</u>), 130.3 (Ar<u>C</u>H), 128.5 (C**3**), 128.0 (C**4**), 127.1 (Ar<u>C</u>H), 127.0 (Ar<u>C</u>H), 126.3 (Ar<u>C</u>H), 63.3 (C**1**), 36.9 (C**2**), 18.9 (C**5**).

HRMS: (ESI^+) Calculated for $C_{11}H_{14}NaO$: 185.0937. Found $[M+Na]^+$: 185.0930.

_N_-(2-(Prop-1-en-1-yl)phenethyl)-_N_-(pentafluorobenzoyloxy)-methanesulfonamide (7q)

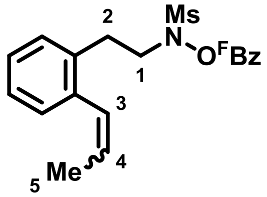

General procedure B: The preceding alcohol (59.3 mg, 0.366 mmol) was employed with **4b** (1.9 eq.), PPh_3 (2.6 eq.) and DEAD (2.6 eq.). FCC (eluent PhMe) afforded **7q** (93.9 mg, 57 %) as a crystalline colorless solid.

ν_{max} / cm^{-1}: (solid) 3024 (m), 2943 (m), 1787 (s), 1653 (m), 1498 (s), 1165 (s).

Spectroscopic data for the major Z isomer:

δ_H (400 MHz, $CDCl_3$) 7.26 – 7.10 (4H, m, ArCH), 6.49 (1H, dq, *J* = 11.5, 2.0 Hz, C**3**-H), 5.86 (1H, dq, *J* = 11.5, 7.0 Hz, C**4**-H), 3.66 – 3.58 (2H, m, C**1**-H_2), 3.05 – 3.02 (2H, m, C**2**-H_2), 3.00 (3H, s, Ms CH_3), 1.69 (3H, dd, *J* = 7.0, 2.0 Hz, C**5**-H_3).

δ_C (101 MHz, $CDCl_3$) 136.6 (ArC), 135.1 (ArC), 129.9 (ArCH), 129.7 (ArCH), 128.4 (C**4**), 127.8 (C**3**), 127.2 (ArCH), 126.7 (ArCH), 52.7 (C**1**), 34.7 (Ms CH_3), 31.4 (C**2**), 14.3 (C**5**).

The signals corresponding to the pentafluorobenzoyl group could not be resolved due to their weak intensity.

δ_F (377 MHz, $CDCl_3$) -135.6 – -135.8 (2F, m), -144.9 – -145.2 (1F, m), -158.6 – -158.8 (2F, m).

Spectroscopic data for the minor E isomer:

δ_H (400 MHz, $CDCl_3$) 7.41 – 7.37 (1H, m, ArCH), 7.24 – 7.13 (3H, m, ArCH), 6.59 (1H, dq, *J* = 15.5, 2.0 Hz, C**3**-H), 6.13 (1H, dq, *J* = 15.5, 6.5 Hz, C**4**-H), 3.67 – 3.57 (2H, m, C**1**-H_2), 3.13 – 3.08 (2H, m, C**2**-H_2), 3.02 (3H, s, Ms CH_3), 1.89 (3H, dd, *J* = 6.5, 2.0 Hz, C**5**-H_3).

δ_C (101 MHz, $CDCl_3$) 137.2 (ArC), 133.5 (ArC), 129.9 (ArCH), 128.9 (C**4**), 127.6 (C**3**), 127.4 (ArCH), 127.2 (ArCH), 126.4 (ArCH), 53.1 (C**1**), 34.7 (Ms CH_3), 31.2 (C**2**), 18.7 (C**5**).

The signals corresponding to the pentafluorobenzoyl group could not be resolved due to their weak intensity.

δ_F (377 MHz, $CDCl_3$) -135.6 – -135.8 (2F, m), -144.9 – -145.2 (1F, m), -158.6 – -158.8 (2F, m).

HRMS: (ESI^+) Calculated for $C_{19}H_{16}F_5NNaO_4S$: 472.0612. Found $[M+Na]^+$: 472.0614.

2-Methylenehex-5-en-1-ol

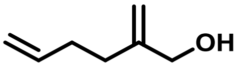

This compound was prepared according to a literature procedure.[26]

The spectroscopic properties were consistent with the data available in the literature.[26,27]

Ethyl 4-methyleneoct-7-enoate

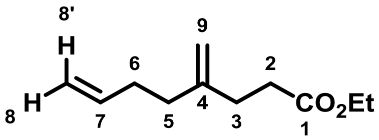

General procedure F: The preceding allylic alcohol (2.24 g, 20.0 mmol) was employed. FCC (eluent 29:1 hexane:EtOAc) afforded the title compound (2.26 g, 62 %) as a colorless oil.

ν_{max} / cm^{-1}: (film) 3079 (m), 2981 (s), 2934 (s), 1785 (s), 1643 (s), 1445 (s), 1371 (s), 1154 (s).

δ_H (400 MHz, $CDCl_3$) 5.80 (1H, ddt, *J* = 17.0, 10.5, 6.5 Hz, C**7**-H), 5.02 (1H, ddt, *J* = 17.0, 2.0, 1.5 Hz, C**8**-H'), 4.95 (1H, ddt, *J* = 10.5, 2.0, 1.0 Hz, C**8**-H), 4.76 (1H, br s, C**9**-H), 4.74 (1H, br s, C**9**-H'), 4.12 (2H, q, *J* = 7.0 Hz, $OC\underline{H}_2CH_3$), 2.47 – 2.42 (2H, m, C**2**-H_2), 2.36 – 2.30 (2H, m, C**3**-H_2), 2.23 – 2.16 (2H, m, C**6**-H_2), 2.14 – 2.08 (2H, m, C**5**-H_2), 1.25 (3H, t, *J* = 7.0 Hz, $OCH_2C\underline{H}_3$).

δ_C (101 MHz, $CDCl_3$) 173.4 (C**1**), 147.5 (C**4**), 138.3 (C**7**), 114.8 (C**8**), 109.7 (C**9**), 60.5 ($O\underline{C}H_2CH_3$), 35.7 (C**5**), 32.9 (C**2**), 32.1 (C**6**), 31.1 (C**3**), 14.4 ($OCH_2\underline{C}H_3$).

HRMS: (ESI^+) Calculated for $C_{11}H_{18}NaO_2$: 205.1199. Found $[M+Na]^+$: 205.1190.

4-Methyleneoct-7-en-1-ol

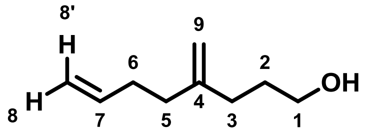

General procedure D: The preceding ester (1.00 g, 5.49 mmol) was employed, using anhydrous Et_2O as the solvent and 0.8 eq. $LiAlH_4$ (1M in Et_2O). The title compound (765 mg, 99 %) was isolated as a colorless oil.

ν_{max} / cm^{-1}: (film) 3323 (br s), 3078 (m), 2932 (s), 1642 (s), 1442 (s), 1057 (s).

δ_H (400 MHz, $CDCl_3$) 5.81 (1H, ddt, *J* = 17.0, 10.0, 6.5 Hz, C**7**-H), 5.01 (1H, ddt, *J* = 17.0, 1.5, 1.5 Hz, C**8**-H'), 4.97 – 4.92 (1H, m, C**8**-H), 4.76 (1H, br s, C**9**-H), 4.75 (1H, br s, C**9**-H'), 3.64 (2H, t, *J* = 6.5 Hz, C**1**-H_2), 2.23 – 2.15 (2H, m, C**6**-H_2), 2.14 – 2.05 (4H, m, C**3**-H_2 and C**5**-H_2), 1.77 (1H, br s, OH), 1.70 (2H, tt, *J* = 7.5, 6.5 Hz, C**2**-H_2).

δ_C (101 MHz, $CDCl_3$) 148.7 (C**4**), 138.5 (C**7**), 114.7 (C**8**), 109.5 (C**9**), 62.7 (C**1**), 35.4 (C**5**), 32.5 (C**3**), 32.1 (C**6**), 30.7 (C**2**).

HRMS: (EI^+) Calculated for C_9H_{14}: 122.1096. Found $[M\text{-}H_2O]^+$: 122.1091.

***N*-4-Methyleneoct-7-en-1-yl-*N*-(pentafluorobenzoyloxy)methanesulfonamide (7r)**

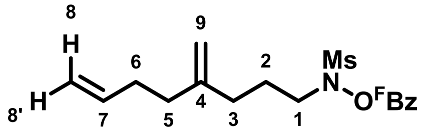

General procedure B: The preceding alcohol (66.6 mg, 0.475 mmol) and **4b** were employed. FCC (eluents 250:1 hexane:EtOAc followed by PhMe) afforded **7r** (128 mg, 63 %) as a crystalline colorless solid.

ν_{max} / cm^{-1}: (solid) 3078 (m), 2940 (s), 1782 (s), 1656 (m), 1500 (s), 1355 (s), 1162 (s).

δ_H (400 MHz, $CDCl_3$) 5.81 (1H, ddt, *J* = 17.0, 10.0, 7.0 Hz, C**7**-H), 5.02 (1H, ddt, *J* = 17.0, 2,0, 1.0 Hz, C**8**-H), 4.96 (1H, ddt, *J* = 10.0, 2.0, 1.0 Hz, C**8**-H'), 4.80 (1H, s, C**9**-H), 4.78 (1H, s, C**9**-H), 3.48 (2H, t, *J* = 7.0 Hz, C**1**-H_2), 3.04 (3H, s, Ms CH_3), 2.20 (4H, m, C**3**-H_2 and C**6**-H_2), 2.10 (2H, t, *J* = 7.5 Hz, C**5**-H_2), 1.82 (2H, tt, *J* = 7.5, 7.0 Hz, C**2**-H_2).

δ_C (101 MHz, $CDCl_3$) 156.4 (^{F}Bz <u>C</u>=O), 147.3 (C**4**), 138.3 (C**7**), 114.8 (C**8**), 110.5 (C**9**), 52.2 (C**1**), 35.3 (C**5**), 34.4 (Ms <u>C</u>H_3), 32.7 (C**3**), 32.0 (C**6**), 24.9 (C**2**).

The aromatic signals corresponding to the pentafluorobenzoyl group could not be resolved due to their weak intensity.

δ_F (377 MHz, $CDCl_3$) -135.8 – -136.0 (2F, m), -145.2 (1F, tt, *J* = 21.0, 5.5 Hz), -158.7 – -158.9 (2F, m).

HRMS: (ESI$^+$) Calculated for $C_{17}H_{18}F_5NNaO_4S$: 450.0769. Found $[M+Na]^+$: 450.0764.

Catalysis products:

_N_-(4-oxopentyl)-4-toluenesulfonamide (8a')

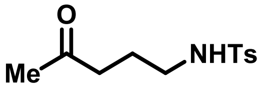

<u>**General procedure G:**</u> Conditions: 3.75 mol % $Pd_2(dba)_3$; 15 mol % $P(3,5\text{-}(CF_3)_2C_6H_3)_3$; 200 mol % Et_3N; DMF (0.1 M); 120 °C; 2 hours. Substrate **7a** (50.0 mg, 0.111 mmol) was employed. FCC (gradient elution, 19:1 – 9:1 – 4:1 – 0:1 hexane:EtOAc) afforded **8a'** as a colorless oil (23 mg, 81 %).

ν_{max} / cm^{-1}: (film) 3278 (br s), 2925 (m), 1709 (s), 1598 (m), 1324 (s), 1155 (s), 1092 (s).

δ_H (400 MHz, $CDCl_3$) 7.72 (2H, d, *J* = 8.5 Hz), 7.29 (2H, d, *J* = 8.5 Hz), 4.83 (1H, t, *J* = 6.5 Hz), 2.93 (2H, dt, *J* = 6.5, 6.5 Hz), 2.50 (2H, t, *J* = 6.5 Hz), 2.41 (3H, s), 2.11 (3H, s), 1.72 (2H, tt, *J* = 6.5, 6.5 Hz).

δ_C (101 MHz, $CDCl_3$) 208.5, 143.6, 137.0, 129.9, 127.2, 42.7, 40.3, 30.2, 23.4, 21.6.

HRMS: (ESI$^+$) Calculated for $C_{12}H_{18}NO_3S$: 256.1002. Found $[M+H]^+$: 256.1003.

The spectroscopic properties were consistent with the data available in the literature.[28]

(3a*R,7a*S**)-1-Tosyl-2,3,3a,4,5,7a-hexahydro-1*H*-indole (8b) and (*iso*-8b)**

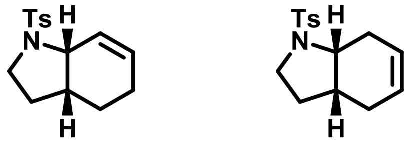

8b ***iso*-8b**

General procedure G: Conditions: 2.5 mol % $Pd_2(dba)_3$; 12.5 mol % $P(3,5\text{-}(CF_3)_2C_6H_3)_3$; 50 mol % Et_3N; 6:1 *n*-BuCN:DMF (0.1 M); 110 °C; 17 hours. Substrate **7ba** (68.5 mg, 0.140 mmol) was employed. FCC (9:1 hexane:EtOAc) afforded **8b** and ***iso*-8b** (35.4 mg, 91 %, 12:1 **8b**:***iso*-8b**) as a pale yellow oil.

ν_{max} / cm^{-1}: (film) 3031 (m), 2923 (m), 1598 (m), 1450 (m), 1338 (s), 1157 (s), 1091 (s), 1048 (s).

^{1}H (500 MHz, $CDCl_3$) δ 7.73 (2H, d, *J* = 8.0 Hz), 7.30 (2H, d, *J* = 8.0 Hz), 5.83 (1H, dddd, *J* = 10.5, 2.5, 2.5, 2.5 Hz), 5.80 – 5.72 (1H, m), 3.99 (1H, dd, *J* = 5.5, 2.5 Hz), 3.47 (1H, ddd, *J* = 10.0, 7.5, 4.0 Hz), 3.16 (1H, ddd, *J* = 10.0, 7.5, 7.5 Hz), 2.42 (3H, s), 2.04 – 1.95 (2H, m), 1.95 – 1.87 (1H, m), 1.76 (1H, dddd, *J* = 12.0, 8.0, 7.5, 7.5 Hz), 1.71 – 1.51 (3H, m).

^{13}C (126 MHz, $CDCl_3$) δ 143.3, 135.1, 129.7, 128.4, 127.7, 127.6, 57.6, 47.4, 36.6, 27.8, 23.0, 21.6, 21.0.

The spectroscopic properties were consistent with the data available in the literature.[29]

Characteristic signals for ***iso-8b*** *(obtained from 1D TOCSY, irradiated signal at 3.70 ppm):*

δ_H NMR (500 MHz, $CDCl_3$) 5.63 – 5.60 (m), 3.70 (ddd, *J* = 7.5, 7.5, 7.5 Hz, 3.50 – 3.45 (m), 3.12 (ddd, *J* = 10.0, 10.0, 7.5 Hz), 2.53 – 2.45 (m), 2.31 – 2.22 (m), 2.14 – 2.05 (m), 1.97 – 1.91 (m), 1.91 – 1.84 (m), 1.84 – 1.77 (m), 1.76 – 1.69 (m).

HRMS: (ESI^+) Calculated for $C_{15}H_{20}NO_2S$: 278.1209. Found $[M+H]^+$: 278.1207.

(3a*R,6a*S**)-1-Tosyl-1,2,3,3a,4,6a-hexahydrocyclopenta[*b*]pyrrole (8c)**

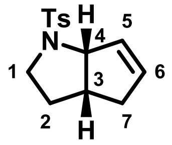

General procedure G: Conditions: 2.5 mol % $Pd_2(dba)_3$; 12.5 mol % $P(3,5\text{-}(CF_3)_2C_6H_3)_3$; 25 mol % Et_3N; *n*-BuCN (0.1 M); 110 °C; 17 hours. Substrate **7c** (66.5 mg, 0.140 mmol) was employed. FCC (gradient elution, 9:1 – 4:1 hexane:EtOAc) afforded **8c** (33.7 mg, 91 %) as a crystalline colorless solid.

m.p. 66-67 °C (Et_2O:hexane)

ν_{max} / cm^{-1}: (solid) 2865 (m), 1596 (m), 1339 (s), 1155 (s).

δ_H (400 MHz, $CDCl_3$) 7.73 (2H, d, *J* = 8.5 Hz, ArCH), 7.31 (2H, d, *J* = 8.5 Hz, ArCH), 5.83 – 5.79 (1H, m, C**5**-H), 5.76 – 5.71 (1H, m, C**6**-H), 4.55 (1H, dd, *J* = 8.0, 2.0 Hz, C**4**-H), 3.36 (1H, ddd, *J* = 9.5, 7.0, 4.5 Hz, C**1**-H), 3.10 – 3.02 (1H, m, C**1**-H'), 2.61 (1H, ddddd, *J* = 8.0, 8.0, 8.0, 7.5, 2.0 Hz, C**3**-H), 2.53 – 2.44 (1H, m, C**7**-H), 2.42 (3H, s, Ts CH_3), 2.10 (1H, ddd, *J* = 17.0, 2.0, 2.0 Hz, 1H, C**7**-H'), 1.88 – 1.79 (1H, m, C**2**-H), 1.51 (1H, dddd, *J* = 12.5, 7.5, 7.5, 7.0 Hz, C**2**-H').

δ_C (101 MHz, $CDCl_3$) 143.4 (ArC), 134.9 (ArC), 132.0 (C**6**), 131.4 (C**5**), 129.7 (ArCH), 127.7 (ArCH), 70.2 (C**4**), 48.4 (C**1**), 40.0 (C**3**), 38.1 (C**7**), 32.5 (C**2**), 21.6 (Ts CH_3).

HRMS: (ESI^+) Calculated for $C_{14}H_{17}NNaO_2S$: 286.0872. Found $[M+Na]^+$: 286.0873.

1-Tosyl-2-vinylpyrrolidine (8d)

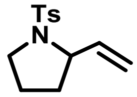

General procedure G: Conditions: 2.5 mol % $Pd_2(dba)_3$; 12.5 mol % $P(3,5\text{-}(CF_3)_2C_6H_3)_3$; 25 mol % Et_3N; 3:1 THF:DMF (0.1 M); 110 °C; 8 hours. Substrate **7d** (65.0 mg, 0.140 mmol) was employed. FCC (9:1 hexane:EtOAc) afforded **8d** (28.4 mg, 81 %) as a crystalline colorless solid.

A yield of 80 % was achieved when this reaction was performed on a 1.40 mmol scale.

m.p. 69-70 °C (Et_2O:hexane) [Lit., 70 °C (no recrystallization solvent quoted)][30]

ν_{max} / cm^{-1}: (solid) 2986 (m), 2957 (m), 1595 (m), 1460 (m), 1335 (s), 1154 (s), 1088 (s), 1000 (s).

δ_H (400 MHz, $CDCl_3$) 7.71 (2H, d, *J* = 8.0 Hz), 7.30 (2H, d, *J* = 8.0 Hz), 5.80 (1H, ddd, *J* = 17.0, 10.0, 6.0 Hz), 5.27 (1H, ddd, *J* = 17.0, 1.5, 1.5 Hz), 5.11 (1H, ddd, *J* = 10.0, 1.5, 1.5 Hz), 4.13 (1H, ddddd, *J* = 6.0, 6.0, 6.0, 1.5, 1.5 Hz), 3.44 (1H, ddd, *J* = 10.0, 7.0, 4.5 Hz), 3.23 (1H, ddd, *J* = 10.0, 7.5, 7.5 Hz), 2.42 (3H, s), 1.87 – 1.73 (1H, m), 1.73 – 1.55 (3H, m).

δ_C (101 MHz, $CDCl_3$) 143.4, 138.8, 135.3, 129.7, 127.6, 115.4, 62.0, 48.9, 32.4, 23.9, 21.6.

m/z (ESI^+) 274 ($[M+Na]^+$, 100 %), 252 ($[M+H]^+$, 20 %).

The spectroscopic properties were consistent with the data available in the literature.[30]

(*E*)-2-Styryl-1-tosylpyrrolidine (8e)

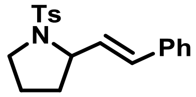

<u>General procedure G:</u> Conditions: 2.5 mol % $Pd_2(dba)_3$; 12.5 mol % $P(3,5-(CF_3)_2C_6H_3)_3$; 25 mol % Et_3N; *n*-BuCN (0.1 M); 110 °C; 15 hours. Substrate **7e** (75.5 mg, 0.140 mmol) was employed. FCC (9:1 hexane:EtOAc) afforded **8e** (35.4 mg, 77 %) as a pale yellow oil.

δ_H (400 MHz, $CDCl_3$) 7.72 (2H, d, *J* = 8.0 Hz), 7.33 – 7.18 (7H, m), 6.54 (1H, d, *J* = 16.0 Hz), 6.04 (1H, dd, *J* = 16.0, 6.5 Hz), 4.34 (1H, ddd, *J* = 7.0, 6.5, 4.5 Hz), 3.48 (1H, ddd, *J* = 10.0, 7.0, 4.5 Hz), 3.34 (1H, ddd, *J* = 10.0, 7.0, 7.0 Hz), 2.39 (3H, s), 1.94 – 1.79 (2H, m), 1.77 – 1.65 (2H, m).

δ_C (101 MHz, $CDCl_3$) 143.3, 136.7, 135.7, 130.7, 130.1, 129.7, 128.6, 127.7, 127.6, 126.6, 61.8, 48.8, 32.9, 24.1, 21.6.

The spectroscopic properties were consistent with the data available in the literature.[31]

2-Methyl-1-mesyl-2-vinylpyrrolidine (8f)

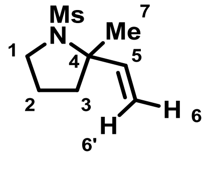

General procedure G: Conditions: 3.75 mol % $Pd_2(dba)_3$; 18.75 mol % $P(3,5\text{-}(CF_3)_2C_6H_3)_3$; 25 mol % Et_3N; 6:1 *n*-BuCN:DMF (0.1 M); 110 °C. Substrate **7f** (56.7 mg, 0.140 mmol) was employed. FCC (10:1 PhMe:EtOAc) afforded **8f** (21.3 mg, 80 %) as a pale yellow oil.

ν_{max} / cm^{-1}: (film) 2976 (m), 2928 (m), 1330 (s), 1148 (s).

δ_H (400 MHz, $CDCl_3$) 5.98 (1H, dd, *J* = 17.5, 10.5 Hz, C**5**-H), 5.24 (1H, dd, *J* = 17.5, 0.5 Hz, C**6**-H'), 5.15 (1H, dd, *J* = 10.5, 0.5 Hz, C**6**-H), 3.53 – 3.44 (2H, m, C**1**-H_2), 2.86 (3H, s, Ms CH_3), 2.04 – 1.97 (1H, m, C**3**-H), 1.94 – 1.81 (3H, m, C**2**-H_2 and C**3**-H'), 1.57 (3H, s, C**7**-H_3).

δ_C (101 MHz, $CDCl_3$) 142.1 (C**5**), 114.0 (C**6**), 67.0 (C**4**), 49.5 (C**1**), 41.7 (C**3**), 39.7 (Ms CH_3), 25.1 (C**7**), 22.4 (C**2**).

HRMS: (ESI^+) Calculated for $C_8H_{15}NNaO_2S$: 212.0716. Found $[M+Na]^+$: 212.0714.

1-Mesyl-1-azaspiro[4.5]dec-6-ene (8g)

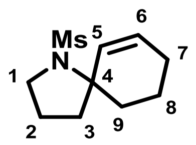

General procedure G: Conditions: 3.75 mol % $Pd_2(dba)_3$; 18.75 mol % $P(3,5\text{-}(CF_3)_2C_6H_3)_3$; 25 mol % Et_3N; 6:1 *n*-BuCN:DMF (0.1 M); 110 °C; 16 hours. Substrate **7g** (59.8 mg, 0.140 mmol) was employed. FCC (29:1 PhMe:acetone) afforded **8g** (12.8 mg, 42 %) as a yellow crystalline solid.

ν_{max} / cm^{-1}: (solid) 3023 (m), 2929 (m), 1447 (m), 1317 (s), 1143 (s).

δ_H (400 MHz, $CDCl_3$) 5.82 (1H, ddd, *J* = 10.0, 5.5, 2.5 Hz, C**6**-H), 5.62 – 5.57 (1H, m, C**5**-H), 3.57 (1H, ddd, *J* = 10.5, 6.0, 3.5 Hz, C**1**-H), 3.42 – 3.34 (1H, m, C**1**-H'), 2.89 (3H, s, Ms CH_3), 2.35 (1H, ddd, *J* = 13.0, 13.0, 3.5 Hz, C**9**-H), 2.09 (1H, dddd, *J* = 16.5, 11.0, 5.5, 2.5 Hz, C**7**-H), 2.02 – 1.78 (6H, m, C**2**-H_2, C**3**-H, C**3**-H' C**7**-H' and C**8**-H), 1.77 – 1.68 (1H, m, C**9**-H'), 1.58 – 1.47 (1H, m, C**8**-H').

δ_C (101 MHz, $CDCl_3$) 131.5 (C**5**), 129.2 (C**6**), 66.6 (C**4**), 49.2 (C**1**), 41.1 (C**3**), 40.0 (Ms CH_3), 34.4 (C**9**), 24.3 (C**7**), 22.6 (C**2**), 21.6 (C**8**).

HRMS: (ESI^+) Calculated for $C_{10}H_{17}NNaO_2S$: 238.0872. Found $[M+Na]^+$: 238.0878.

2-Benzyl-1-mesyl-2-vinylpyrrolidine (8h)

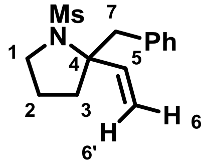

General procedure G: Conditions: 5.0 mol % $Pd_2(dba)_3$; 25 mol % $P(3,5\text{-}(CF_3)_2C_6H_3)_3$; 25 mol % Et_3N; *n*-BuCN (0.1 M); 95 °C; 16 hours. Substrate **7h** (66.8 mg, 0.140 mmol) was employed. FCC (gradient elution 5:1 – 3:1 hexane:EtOAc) afforded **8h** (19.9 mg, 54 %) as a pale yellow oil.

ν_{max} / cm^{-1}: (film) 3027 (m), 2978 (m), 1602 (m), 1496 (m), 1318 (s), 1144 (s).

δ_H (500 MHz, $CDCl_3$) 7.32 – 7.27 (4H, m, ArC<u>H</u>), 7.25 – 7.21 (1H, m, ArC<u>H</u>), 6.24 (1H, dd, *J* = 17.5, 11.0 Hz, C**5**-<u>H</u>), 5.27 (1H, dd, *J* = 11.0, 0.5 Hz, C**6**-<u>H</u>), 5.26 (1H, dd, *J* = 17.5, 0.5 Hz, C**6**-<u>H</u>'), 3.40 (1H, d, *J* = 13.5 Hz, C**7**-<u>H</u>), 3.35 – 3.26 (2H, m, C**1**-<u>H</u>$_2$), 3.06 (1H, d, *J* = 13.5 Hz, C**7**-<u>H</u>'), 2.90 (3H, s, Ms C<u>H</u>$_3$), 2.05 (1H, ddd, *J* = 13.0, 7.0, 6.5 Hz, C**3**-<u>H</u>), 1.93 (1H, ddd, *J* = 13.0, 7.0, 7.0 Hz, C**3**-<u>H</u>'), 1.71 – 1.62 (1H, m, C**2**-<u>H</u>), 1.34 (1H, dddd, *J* = 14.0, 12.5, 7.0, 7.0 Hz, C**2**-<u>H</u>').

δ_C (126 MHz, $CDCl_3$) 140.3 (C**5**), 137.3 (Ar<u>C</u>), 131.1 (Ar<u>C</u>H), 128.2 (Ar<u>C</u>H), 126.7 (Ar<u>C</u>H), 115.5 (C**6**), 70.1 (C**4**), 49.9 (C**1**), 44.7 (C**7**), 39.4 (Ms <u>C</u>H$_3$), 36.4 (C**3**), 22.4 (C**2**).

HRMS: (ESI^+) Calculated for $C_{14}H_{19}NNaO_2S$: 288.1029. Found $[M+Na]^+$: 288.1017.

3-Phenyl-1-tosyl-2-vinylpyrrolidine (8i)

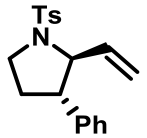

General procedure G: Conditions: 2.5 mol % $Pd_2(dba)_3$; 12.5 mol % $P(3,5\text{-}(CF_3)_2C_6H_3)_3$; 25 mol % Et_3N; *n*-BuCN (0.1 M); 110 °C; 16 hours. Substrate **7i** (75.5 mg, 0.140 mmol) was employed. FCC (gradient elution, 9:1 – 4:1 hexane:EtOAc) afforded **8i** (37.2 mg, 81 %, 2:1 mixture of *trans* and *cis* diastereomers) as a colorless oil.

ν_{max} / cm^{-1}: (film) 3029 (m), 2979 (m), 1598 (m), 1345 (s), 1159 (s).

Spectroscopic data for the major trans diastereomer:

δ_H (400 MHz, $CDCl_3$) 7.74 (2H, d, *J* = 8.0 Hz), 7.38 – 7.16 (5H, m), 6.95 – 6.89 (2H, m), 5.86 (1H, ddd, *J* = 17.0, 10.5, 7.0 Hz), 5.08 (1H, d, *J* = 10.5 Hz), 5.06 (1H, d, *J* = 17.0 Hz), 3.97 (1H, dd, *J* = 7.0, 7.0 Hz), 3.74 – 3.63 (1H, m), 3.53 (1H, ddd, *J* = 11.0, 9.0, 6.5 Hz), 3.07 (1H, ddd, *J* = 9.5, 7.0, 7.0 Hz), 2.47 (3H, s), 2.18 – 2.01 (1H, m), 1.76 – 1.59 (1H, m).

δ_C (101 MHz, $CDCl_3$) 143.4, 140.1, 137.6, 135.4, 129.6, 128.5, 127.6, 127.4, 127.0, 116.2, 69.5, 52.0, 48.6, 32.3, 21.6.

Characteristic signals for the minor cis diastereomer:

δ_H (400 MHz, $CDCl_3$) 7.80 (2H, d, *J* = 8.0 Hz), 5.38 – 5.24 (2H, m), 4.52 (1H, dd, *J* = 8.0, 4.5 Hz), 3.35 (1H, ddd, *J* = 10.0, 10.0, 7.0 Hz), 2.31 – 2.20 (1H, m).

The spectroscopic properties were consistent with the data available in the literature.[32]

***cis*-2-Cyclopropyl-1-tosyl-5-vinylpyrrolidine (8j)**

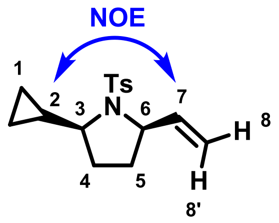

General procedure G: Conditions: 5.0 mol % $Pd_2(dba)_3$; 25 mol % $P(3,5-(CF_3)_2C_6H_3)_3$; 25 mol % Et_3N; PhMe (0.1 M); 140 °C; 15 hours. Substrate **7j** (76.3 mg, 0.152 mmol, added as a solution in PhMe) was employed. FCC (9:1 hexane:EtOAc) afforded **8j** (33.2 mg, 75 %, 13:1 mixture of *cis* and *trans* diastereomers) as a colorless crystalline solid.

ν_{max} / cm^{-1}: (film) 3007 (m), 2967 (m), 1598 (m), 1494 (m), 1344 (s), 1158 (s).

Spectroscopic data for the major cis diastereomer:

δ_H (400 MHz, $CDCl_3$) 7.71 (2H, d, *J* = 8.0 Hz, ArC<u>H</u>), 7.27 (2H, d, *J* = 8.0 Hz, ArC<u>H</u>), 5.86 (1H, ddd, *J* = 17.0, 10.5, 6.0 Hz, C**7**-<u>H</u>), 5.29 (1H, ddd, *J* = 17.0, 1.5, 1.5 Hz, C**8**-<u>H</u>'), 5.10 (1H, ddd, *J* = 10.5, 1.5, 1.5 Hz, C**8**-<u>H</u>), 4.17 – 4.10 (1H, m, C**6**-<u>H</u>), 3.26 (1H, ddd, *J* = 8.0, 8.0, 4.0 Hz, C**3**-<u>H</u>), 2.41 (3H, s, Ts C$\underline{H}_3$), 1.78 – 1.71 (2H, m, C**5**-$\underline{H}_2$), 1.70 – 1.61 (1H, m, C**4**-<u>H</u>), 1.57 – 1.46 (1H, m, C**4**-<u>H</u>'), 0.96 (1H, dddt, *J* = 8.0, 8.0, 8.0, 5.0 Hz, C**2**-<u>H</u>), 0.62 – 0.49 (2H, m, C**1**-$\underline{H}_2$), 0.48 – 0.39 (1H, m, C**1**'-<u>H</u>), 0.25 – 0.17 (1H, m, C**1**'-<u>H</u>').

δ_C (101 MHz, $CDCl_3$) 143.1 (Ar<u>C</u>), 139.5 (C**7**), 136.2 (Ar<u>C</u>), 129.4 (Ar<u>C</u>H), 127.6 (Ar<u>C</u>H), 115.4 (C**8**), 65.9 (C**3**), 63.3 (C**6**), 31.2 (C**5**), 30.3 (C**4**), 21.5 (Ts <u>C</u>H_3), 16.9 (C**2**), 4.7 (C**1**), 2.8 (C**1**').

Characteristic signals for the minor trans diastereomer:

δ_H (400 MHz, $CDCl_3$) 5.77 (1H, ddd, *J* = 17.0, 10.0, 7.5 Hz), 5.20 (1H, ddd, *J* = 17.0, 1.0, 1.0 Hz), 5.06 (1H, ddd, *J* = 10.0, 1.0, 1.0 Hz), 4.42 (1H, dd, *J* = 7.5, 7.5 Hz).

HRMS: (ESI$^+$) Calculated for $C_{16}H_{22}NO_2S$: 292.1366. Found $[M+H]^+$: 292.1376.

***cis*-1-Mesyl-2-phenyl-5-vinylpyrrolidine (8k)**

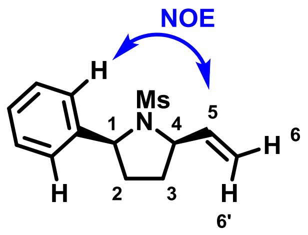

<u>General procedure G:</u> Conditions: 5.0 mol % $Pd_2(dba)_3$; 25 mol % $P(3,5-(CF_3)_2C_6H_3)_3$; 25 mol % Et_3N; 6:1 *n*-BuCN:DMF (0.1 M); 120 °C; 19 hours. Substrate **7k** (64.9 mg, 0.140 mmol) was employed. FCC (gradient elution, 10:1 – 5:1 – 2:1 hexane:EtOAc) afforded **8k** (20.4 mg, 58 %) as a pale yellow oil.

ν_{max} / cm^{-1}: (film) 3029 (m), 2934 (m), 1451 (m), 1328 (s), 1145 (s).

δ_H (400 MHz, $CDCl_3$) 7.41 – 7.32 (4H, m, ArC<u>H</u>), 7.31 – 7.26 (1H, m, ArC<u>H</u>), 6.02 (1H, ddd, *J* = 17.0, 10.0, 7.0 Hz, C**5**-<u>H</u>), 5.40 (1H, ddd, *J* = 17.0, 1.0, 1.0 Hz, C**6**-<u>H</u>'), 5.27 (1H, ddd, *J* = 10.0, 1.0, 1.0 Hz, C**6**-<u>H</u>), 5.00 (1H, dd, *J* = 7.0, 7.0 Hz, C**1**-<u>H</u>), 4.59 (1H, br ddd, *J* = 7.5, 7.0, 7.0 Hz, C**4**-<u>H</u>), 2.66 (3H, s, Ms C<u>H</u>$_3$), 2.40 – 2.30 (1H, m, C**2**-<u>H</u>), 2.17 (1H, dddd, *J* = 12.0, 7.5, 7.5, 6.0 Hz, C**3**-<u>H</u>), 2.01 (1H, dddd, *J* = 12.5, 7.0, 6.0, 6.0 Hz, C**2**-<u>H</u>'), 1.95 – 1.86 (1H, m, C**3**-<u>H</u>').

δ_C (101 MHz, $CDCl_3$) 142.2 (Ar<u>C</u>), 138.7 (C**5**), 128.7 (Ar<u>C</u>H), 127.7 (Ar<u>C</u>H), 126.9 (Ar<u>C</u>H), 117.2 (C**6**), 64.7 (C**1**), 63.3 (C**4**), 41.3 (Ms <u>C</u>H$_3$), 35.1 (C**2**), 31.5 (C**3**).

HRMS: (ESI$^+$) Calculated for $C_{13}H_{17}NNaO_2S$: 274.0872. Found $[M+Na]^+$: 274.0868.

***cis*-2-Butyl-1-mesyl-5-vinylpyrrolidine (8l)**

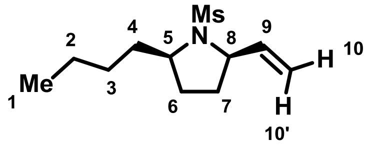

<u>General procedure G:</u> Conditions: 5.0 mol % $Pd_2(dba)_3$; 25 mol % $P(3,5\text{-}(CF_3)_2C_6H_3)_3$; 25 mol % Et_3N; PhMe (0.1 M); 140 °C; 20 hours. Substrate **7l** (62.1 mg, 0.140 mmol) was employed. FCC (5:1 hexane:EtOAc) afforded **8l** (19.9 mg, 61 %, 14:1 ratio of *cis* and *trans* diastereomers) as a pale yellow oil.

ν_{max} / cm^{-1}: (film) 2932 (m), 1331 (s), 1148 (s).

Spectroscopic data for the major cis diastereomer:

δ_H (400 MHz, $CDCl_3$) 5.79 (1H, ddd, *J* = 17.0, 10.0, 6.5 Hz, C**9**-<u>H</u>), 5.30 (1H, ddd, *J* = 17.0, 1.5, 1.5 Hz, C**10**-<u>H</u>’), 5.14 (1H, ddd, *J* = 10.0, 1.5, 1.5 Hz, C**10**-<u>H</u>), 4.29 – 4.23 (1H, m, C**8**-<u>H</u>), 3.81 (dddd, *J* = 9.5, 7.0, 5.0, 5.0 Hz, C**5**-<u>H</u>), 2.84 (3H, s, Ms C<u>H</u>$_3$), 2.10 – 1.93 (2H, m, C**6**-<u>H</u> and C**7**-<u>H</u>), 1.92 – 1.76 (2H, m, C**4**-<u>H</u> and C**7**-<u>H</u>’), 1.72 – 1.62 (1H, m, C**6**-<u>H</u>’), 1.47 – 1.22 (5H, m, C**2**-<u>H</u>$_2$, C**3**-<u>H</u>$_2$ and C**4**-<u>H</u>’), 0.90 (3H, t, *J* = 7.0 Hz, C**1**-H_3).

δ_C (101 MHz, $CDCl_3$) 139.4 (C**9**), 116.1 (C**10**), 62.9 (C**8**), 61.8 (C**5**), 38.8 (Ms <u>C</u>H_3), 36.5 (C**4**), 31.7 (C**7**), 30.2 (C**6**), 28.7 (C**2** or C**3**), 22.7 (C**2** or C**3**), 14.2 (C**1**).

^1H NMR spectrum for the minor trans diastereomer:

δ_H (500 MHz, $CDCl_3$) 5.79 (1H, ddd, *J* = 17.0, 10.0, 8.5 Hz), 5.31 (1H, ddd, *J* = 17.0, 1.0, 1.0 Hz), 5.18 (1H, ddd, *J* = 10.0, 1.0, 1.0 Hz), 4.30 (1H, dd, *J* = 8.5 Hz), 3.74 – 3.68 (1H, m), 2.86 (3H, s), 2.26 – 2.18 (1H, m), 2.11 – 1.95 (2H, m), 1.80 (1H, ddt, *J* = 13.0, 6.5, 1.5 Hz), 1.70 (1H, ddt, *J* = 12.5, 6.5, 1.5 Hz), 1.48 – 1.39 (1H, m), 1.38 – 1.19 (5H, m), 0.90 (3H, t, *J* = 7.0 Hz).

HRMS: (ESI^+) Calculated for $C_{11}H_{21}NNaO_2S$: 254.1185. Found $[M+Na]^+$: 254.1173.

(2*S,5*R**)-5-Cyclopropyl-2-methyl-1-tosyl-2-vinylpyrrolidine (8m)**

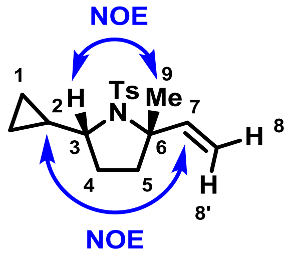

General procedure G: Conditions: 5.0 mol % $Pd_2(dba)_3$; 25 mol % $P(3,5\text{-}(CF_3)_2C_6H_3)_3$; 25 mol % Et_3N; PhMe (0.1 M); 140 °C; 14 hours. Substrate **7m** (79.6 mg, 0.154 mmol, added as a solution in PhMe) was employed. FCC (*two times*: first eluent 19:1 hexane:EtOAc, second eluent 99:1 PhMe:EtOAc) afforded **8m** (30.1 mg, 64 %, 20:1 mixture of diastereomers) as a pale yellow oil.

ν_{max} / cm^{-1}: (film) 3084 (m), 2972 (m), 1599 (m), 1496 (m), 1327 (s), 1151 (s).

Spectroscopic data for the major diastereomer:

δ_H (400 MHz, $CDCl_3$) 7.77 (2H, d, *J* = 8.0 Hz, ArCH), 7.22 (2H, d, *J* = 8.0 Hz, ArCH), 6.10 (1H, dd, *J* = 17.5, 10.5 Hz, C**7**-H), 5.23 (1H, dd, *J* = 17.5, 1.0 Hz, C**8**-H'), 5.08 (1H, dd, *J* = 10.5, 1.0 Hz, C**8**-H), 3.33 (1H, ddd, *J* = 8.5, 7.5, 1.5 Hz, C**3**-H), 2.39 (3H, s, Ts CH_3), 2.15 (1H, ddd, *J* = 12.5, 12.0, 6.5 Hz, C**5**-H), 2.01 (1H, dddd, *J* = 12.5, 12.5, 8.5, 6.5 Hz, C**4**-H), 1.77 – 1.66 (2H, m, C**4**-H' and C**5**-H'), 1.55 (3H, s, C**9**-H_3), 0.90 (1H, ddddd, *J* = 8.5, 8.5, 8.5, 5.0, 5.0 Hz, C**2**-H), 0.53 – 0.46 (1H, m, C**1**-H), 0.44 – 0.32 (2H, m, C**1**-H' and C**1'**-H), 0.09 – 0.00 (1H, m, C**1'**-H').

δ_C (101 MHz, $CDCl_3$) 145.1 (C**7**), 142.4 (ArC), 140.7 (ArC), 129.1 (ArCH), 127.7 (ArCH), 112.9 (C**8**), 69.0 (C**6**), 66.7 (C**3**), 40.1 (C**5**), 29.6 (C**4**), 24.4 (C**9**), 21.6 (Ts CH_3), 17.1 (C**2**), 7.2 (C**1**), 2.8 (C**1'**).

Characteristic signals for the minor diastereomer:

δ_H (500 MHz, $CDCl_3$) 5.91 (1H, dd, *J* = 17.5, 11.0 Hz), 5.17 (1H, dd, *J* = 17.5, 1.0 Hz), 5.02 (1H, dd, *J* = 11.0, 1.0 Hz).

HRMS: (ESI^+) Calculated for $C_{17}H_{23}NNaO_2S$: 328.1342. Found $[M+Na]^+$: 328.1353.

Methyl (*E*)-2-(1-tosylpyrrolidin-2-ylidene)acetate (8n)

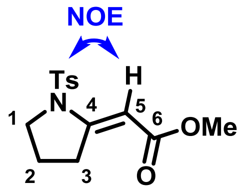

General procedure G: Conditions: 2.5 mol % $Pd_2(dba)_3$; 12.5 mol % $P(3,5\text{-}(CF_3)_2C_6H_3)_3$; 25 mol % Et_3N; 6:1 *n*-BuCN:DMF (0.1 M); 110 °C; 15 hours. Substrate **7n** (71.0 mg, 0.140 mmol) was employed. FCC (gradient elution, 3:1 – 2:1 hexane:EtOAc) afforded **8n** (32.4 mg, 78 %) as a pale yellow oil.

ν_{max} / cm^{-1}: (film) 2950 (m), 1707 (s), 1620 (s), 1346 (s), 1129 (s).

δ_H (400 MHz, $CDCl_3$) 7.75 (2H, d, *J* = 8.0 Hz, ArCH), 7.32 (2H, d, *J* = 8.0 Hz, ArCH), 6.03 (1H, t, *J* = 2.0 Hz, C**5**-H), 3.76 (2H, t, *J* = 7.0 Hz, C**1**-H_2), 3.64 (3H, s, OCH_3), 3.03 (2H, td, *J* = 7.5, 2.0 Hz, C**3**-H_2), 2.42 (3H, s, Ts CH_3), 1.88 (2H, tt, *J* = 7.5, 7.0 Hz, C**2**-H_2).

δ_C (101 MHz, $CDCl_3$) 168.3 (C**6**), 156.9 (C**4**), 145.0 (ArC), 134.3 (ArC), 130.0 (ArCH), 127.4 (ArCH), 94.9 (C**5**), 51.6 (C**1**), 51.0 (OCH_3), 32.4 (C**3**), 21.7 (Ts CH_3), 21.3 (C**2**).

HRMS: (ESI^+) Calculated for $C_{14}H_{17}NNaO_4S$: 318.0770. Found $[M+Na]^+$: 318.0780.

8-Mesyl-8-azabicyclo[3.2.1]oct-2-ene (8o)

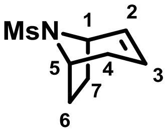

General procedure G: Conditions: 5.0 mol % $Pd_2(dba)_3$; 25 mol % $P(3,5\text{-}(CF_3)_2C_6H_3)_3$; 25 mol % Et_3N; 6:1 *n*-BuCN:DMF (0.1 M); 110 °C; 16 hours. Substrate **7o** (55.9 mg, 0.140 mmol) was employed. FCC (gradient elution, 5:1 – 4:1 – 3:1 hexane:EtOAc) afforded **8o** (15.6 mg, 60 %) as a colorless crystalline solid.

ν_{max} / cm^{-1}: (solid) 3046 (m), 2958 (m), 2926 (m), 1458 (m), 1321 (s), 1137 (s).

δ_H (400 MHz, $CDCl_3$) 5.99 (1H, dddd, *J* = 9.5, 5.5, 2.0, 2.0 Hz, C**2**-H), 5.62 – 5.56 (1H, m, C**3**-H), 4.33 – 4.25 (2H, m, C**1**-H and C**5**-H), 2.93 (3H, s, Ms CH_3), 2.77 – 2.67 (1H, m, C**4**-H), 2.31 – 2.20 (1H, m, C**6**-H), 2.13 – 1.90 (3H, m, C**4**-H’ and C**7**-H_2), 1.77 – 1.67 (1H, m, C**6**-H’).

δ_C (101 MHz, $CDCl_3$) 131.5 (C**2**), 124.1 (C**3**), 55.6 (C**1** or C**5**), 55.5 (C**1** or C**5**), 40.9 (Ms CH_3), 36.1 (C**7**), 35.6 (C**4**), 30.8 (C**6**).

HRMS: (ESI^+) Calculated for $C_8H_{14}NO_2S$: 188.0740. Found $[M+H]^+$: 188.0744.

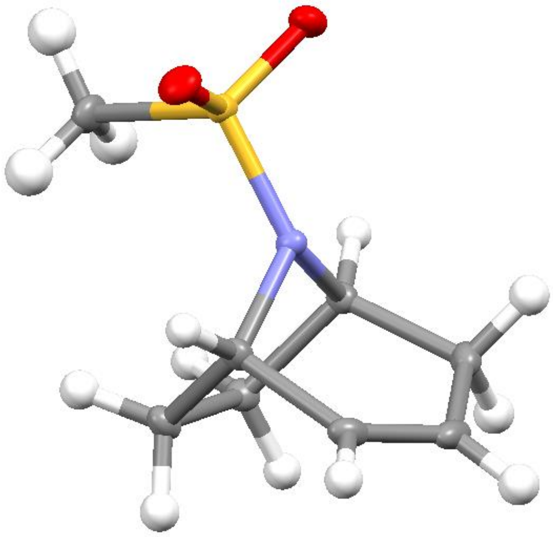

*ORTEP view of **8o***

6-Mesyl-6-azabicyclo[3.2.1]oct-3-ene (8p)

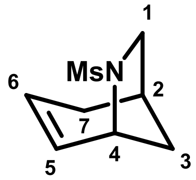

General procedure G: Conditions: 5.0 mol % $Pd_2(dba)_3$; 25 mol % $P(3,5\text{-}(CF_3)_2C_6H_3)_3$; 25 mol % Et_3N; 1:1 *n*-BuCN:THF (0.1 M); 110 °C, 17 hours. Substrate **7p** (55.9 mg, 0.140 mmol) was employed, FCC (9:1 PhMe:EtOAc) afforded **8p** (19.9 mg, 76 %) as a colorless crystalline solid.

m.p. 61-62 °C (Et_2O:hexane, *cubes*)

ν_{max} / cm^{-1}: (solid) 3034 (m), 2967 (m), 1312 (s), 1133 (s).

δ_H (400 MHz, $CDCl_3$) 6.06 (1H, dddd, *J* = 10.5, 5.5, 2.0, 1.0 Hz, C**5**-H), 5.73 – 5.68 (1H, m, C**6**-H), 4.17 (1H, ddd, *J* = 5.5, 5.0, 1.0 Hz, C**4**-H), 3.64 – 3.59 (1H, m, C**1**-H), 3.09 (1H, d, *J* = 10.0 Hz, C**1**-H'), 2.81 (3H, s, Ms CH_3), 2.72 – 2.67 (1H, m, C**2**-H), 2.55 – 2.47 (1H, m, C**7**-H), 2.16 – 2.09 (1H, m, C**7**-H'), 1.95 (1H, ddd, *J* = 11.0, 5.0, 5.0 Hz, C**3**-H), 1.83 (1H, ddd, *J* = 11.0, 1.0, 1.0 Hz, C**3**-H').

δ_C (101 MHz, $CDCl_3$) 129.8 (C**5**), 128.5 (C**6**), 54.1 (C**1**), 54.0 (C**4**), 37.7 (Ms <u>C</u>H_3), 35.6 (C**7**), 34.7 (C**3**), 33.4 (C**2**).

HRMS: (ESI^+) Calculated for $C_8H_{13}NNaO_2S$: 210.0559. Found $[M+Na]^+$: 210.0561.

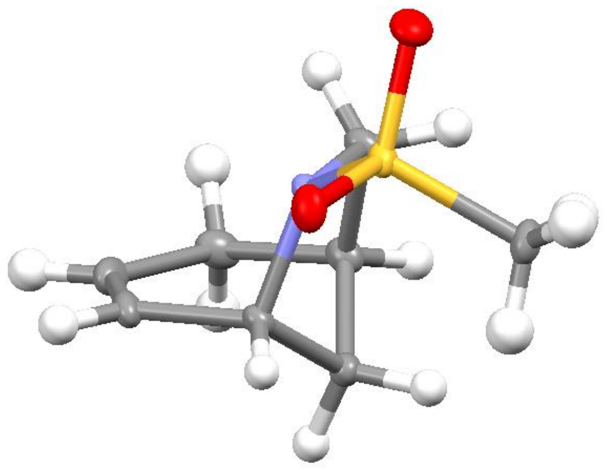

*ORTEP view of **8p***

2-Mesyl-1-vinyl-1,2,3,4-tetrahydroisoquinoline (8q)

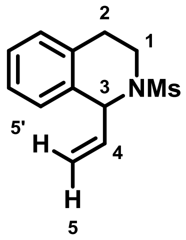

<u>General procedure G:</u> Conditions: 5.0 mol % $Pd_2(dba)_3$; 25 mol % P(3,5-$(CF_3)_2C_6H_3)_3$; 25 mol % Et_3N; 6:1 *n*-BuCN:DMF (0.1 M); 110 °C; 19 hours. Substrate **7q** (62.9 mg, 0.140 mmol) was employed. FCC (gradient elution, 9:1 – 7:1 – 4:1 – 2:1 hexane:EtOAc) afforded **8q** (14.1 mg, 42 %) as a pale yellow oil.

ν_{max} / cm^{-1}: (film) 3022 (m), 2931 (m), 1492 (m), 1452 (m), 1327 (s), 1151 (s).

δ_H (500 MHz, $CDCl_3$) 7.22 – 7.18 (2H, m, ArC<u>H</u>), 7.17 – 7.13 (1H, m, ArC<u>H</u>), 7.12 – 7.08 (1H, m, ArC<u>H</u>), 6.02 (1H, ddd, *J* = 17.0, 10.0, 6.5 Hz, C**4**-<u>H</u>), 5.40 (1H, br d, *J* = 6.5 Hz, C**3**-<u>H</u>), 5.28 (1H, ddd, *J* = 10.0, 1.5, 1.5 Hz, C**5**-<u>H</u>), 5.21 (1H, ddd, *J* = 17.0, 1.5, 1.5 Hz, C**5**-<u>H</u>'), 3.91 (1H, ddd, *J* =

13.5, 6.5, 2.5 Hz, C**1**-H), 3.37 (1H, ddd, *J* = 13.5, 11.5, 4.5 Hz, C**1**-H'), 3.07 (1H, ddd, *J* = 16.5, 11.5, 6.5 Hz, C**2**-H), 2.83 (3H, s, Ms CH$_3$), 2.79 (1H, ddd, *J* = 16.5, 4.5, 2.5 Hz, C**2**-H').

δ_C (126 MHz, $CDCl_3$) 136.8 (C**4**), 133.7 (ArC), 133.3 (ArC), 129.2 (ArCH), 128.0 (ArCH), 127.3 (ArCH), 126.4 (ArCH), 118.1 (C**5**), 58.2 (C**3**), 39.7 (Ms CH$_3$), 39.2 (C**1**), 28.2 (C**2**).

HRMS: (ESI$^+$) Calculated for $C_{12}H_{15}NNaO_2S$: 260.0716. Found $[M+Na]^+$: 260.0716.

7-Methylene-1-mesyl-1-azaspiro[4.4]nonane (8r)

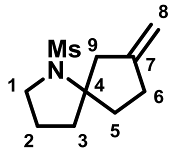

General procedure G: Conditions: 3.75 mol % $Pd_2(dba)_3$; 18.75 mol % $P(3,5\text{-}(CF_3)_2C_6H_3)_3$; 25 mol % Et_3N; 6:1 *n*-BuCN:DMF (0.1 M); 110 °C; 16 hours. Substrate **7r** (59.8 mg, 0.140 mmol) was employed. FCC (gradient elution 4:1 – 2:1 hexane:EtOAc) afforded **8r** (25.9 mg, 86 %) as a colorless crystalline solid.

m.p. 64-66 °C (Et_2O:hexane, *cubes*)

ν_{max} / cm^{-1}: (solid) 3069 (m), 2930 (m), 1655 (m), 1311 (s), 1143 (s).

δ_H (400 MHz, $CDCl_3$) 4.89 – 4.86 (1H, m, C**8**-H), 4.83 – 4.80 (1H, m, C**8**-H'), 3.51 – 3.37 (2H, m, C**1**-H$_2$), 3.09 (1H, dddd, *J* = 15.5, 3.0, 3.0, 3.0 Hz, C**9**-H), 2.86 (3H, s, Ms CH$_3$), 2.59 – 2.42 (2H, m, C**5**-H and C**6**-H), 2.27 – 2.16 (2H, m, C**6**-H' and C**9**-H'), 1.90 – 1.74 (4H, m, C**2**-H$_2$ and C**3**-H$_2$), 1.65 (1H, ddd, *J* = 10.0, 6.0, 2.0 Hz, C**5**-H').

δ_C (101 MHz, $CDCl_3$) 147.8 (C**7**), 107.4 (C**8**), 72.0 (C**4**), 50.0 (C**1**), 44.4 (C**9**), 40.1 (C**3**), 39.3 (Ms CH$_3$), 36.3 (C**5**), 29.8 (C**6**), 22.6 (C**2**).

HRMS: (ESI$^+$) Calculated for $C_{10}H_{17}NNaO_2S$: 238.0872. Found $[M+Na]^+$: 238.0878.

Mitsunobu inversion study:

(*R*)-*N*-(1-phenylethyl)-*N*-(pentafluorobenzoyloxy)-4-toluenesulfonamide

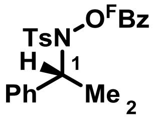

General procedure A: (*S*)-1-Phenylethanol (58.0 mg, 0.475 mmol, 99 % e.e.) and **4a** were employed, FCC (eluents 250:1 hexane:EtOAc followed by PhMe) afforded the title compound (178 mg, 74 %, 96 % e.e.) as a crystalline colorless solid.

$[\alpha]_D^{24}$ = -9.1 (*c* 4.25, $CHCl_3$)

SFC conditions: *column*: CHIRALPACK IC, *elute*: 10 % MeOH/CO_2, *detector*: 250 nm, *flow rate*: 3 mL/min, *temperature*: 40 °C, *retention times*: (*R*) t_1 = 3.6 min, (*S*) t_2 = 3.9 min.

ν_{max} / cm^{-1}: (solid) 2988 (m), 1791 (s), 1658 (m), 1597 (m), 1509 (s), 1167 (s).

Two species were observed in the ^1H and ^{13}C NMR spectra in an approximately 2:1 ratio. These are presumably a pair of diastereomers generated as a result of the nitrogen center not being sp^2 hybridized. The signals were also very broad, likely due to slow inversion of the nitrogen centre.

δ_H (500 MHz, $CDCl_3$) 7.79 (2H, br s, ArC<u>H</u>), 7.52 – 7.12 (7H, m, ArC<u>H</u>), 5.41 – 4.73 (1H, m, C**1**-<u>H</u>), 2.46 (3H, s, Ts C<u>H</u>$_3$), 1.65 (3H, br s, C**2**-<u>H</u>$_3$).

δ_C (126 MHz, $CDCl_3$) 155.9 (^{F}Bz <u>C</u>=O), 145.6 (Ar<u>C</u>), 138.5 (Ar<u>C</u>), 132.5 (Ar<u>C</u>), 129.7 (Ar<u>C</u>H), 129.3 (Ar<u>C</u>H), 128.4 (Ar<u>C</u>H), 128.2 (Ar<u>C</u>H), 127.9 (Ar<u>C</u>H), 63.1 (C**1**), 60.4 (C**1**'), 21.7 (Ts <u>C</u>H$_3$), 21.0 (C**2**), 15.4 (C**2**').

The aromatic signals corresponding to the pentafluorobenzoyl group could not be resolved due to their weak intensity.

δ_F (377 MHz, $CDCl_3$) -135.9 (2F, br s), -146.0 – -146.5 (1F, m), -159.2 (2F, br s).

HRMS: (ESI$^+$) Calculated for $C_{22}H_{16}F_5NNaO_4S$: 508.0612. Found $[M+Na]^+$: 508.0594.

References

(1) Porcheddu, A.; De Luca, L.; Giacomelli, G. *Synlett* **2009**, *2009*, 2149-2153.

(2) Oae, S.; Shinhama, K. i.; Fujimori, K.; Kim, Y. H. *B. Chem. Soc. Jpn.* **1980**, *53*, 775-784.

(3) Kitahara, K.; Toma, T.; Shimokawa, J.; Fukuyama, T. *Org. Lett.* **2008**, *10*, 2259-2261.

(4) Armstrong, A.; Barsanti, P. A.; Clarke, P. A.; Wood, A. *J. Chem. Soc., Perkin Trans. 1* **1996**, 1373-1380.

(5) Lysenko, I. L.; Kim, K.; Lee, H. G.; Cha, J. K. *J. Am. Chem. Soc.* **2008**, *130*, 15997-16002.

(6) Banwell, M. G.; Harvey, J. E.; Hockless, D. C. R.; Wu, A. W. *J. Org. Chem.* **2000**, *65*, 4241-4250.

(7) Wang, Z.-X.; Shi, Y. *J. Org. Chem.* **1998**, *63*, 3099-3104.

(8) Kwon, H. Y.; Park, C. M.; Lee, S. B.; Youn, J.-H.; Kang, S. H. *Chem. Eur. J.* **2008**, *14*, 1023-1028.

(9) Jecs, E.; Diver, S. T. *Org. Lett.* **2015**, *17*, 3510-3513.

(10) Faulkner, A.; Scott, J. S.; Bower, J. F. *Chem. Commun.* **2013**, *49*, 1521-1523.

(11) Paull, D. H.; Fang, C.; Donald, J. R.; Pansick, A. D.; Martin, S. F. *J. Am. Chem. Soc.* **2012**, *134*, 11128-11131.

(12) Gotoh, A.; Sakaeda, T.; Kimura, T.; Shirakawa, T.; Wada, Y.; Wada, A.; Kimachi, T.; Takemoto, Y.; Iida, A.; Iwakawa, S.; Hirai, M.; Tomita, H.; Okamura, N.; Nakamura, T.; Okumura, K. *Biol. Pharm. Bull.* **2004**, *27*, 1070-1074.

(13) Klein, J. E. M. N.; Muller-Bunz, H.; Evans, P. *Org. Biomol. Chem.* **2009**, *7*, 986-995.

(14) Erkkilä, A.; Pihko, P. M. *Eur. J. Org. Chem.* **2007**, *2007*, 4205-4216.

(15) Kelly, C. B.; Ovian, J. M.; Cywar, R. M.; Gosselin, T. R.; Wiles, R. J.; Leadbeater, N. E. *Org. Biomol. Chem.* **2015**, *13*, 4255-4259.

(16) Carman, L.; Kwart, L. D.; Hudlicky, T. *Synth. Commun* **1986**, *16*, 169-182.

(17) Ocejo, M.; Carrillo, L.; Badía, D.; Vicario, J. L.; Fernández, N.; Reyes, E. *J. Org. Chem.* **2009**, *74*, 4404-4407.

(18) Hoover, J. M.; Stahl, S. S. *J. Am. Chem. Soc.* **2011**, *133*, 16901-16910.

(19) Hay, M. B.; Wolfe, J. P. *Tetrahedron Lett.* **2006**, *47*, 2793-2796.

(20) Kimura, M.; Ezoe, A.; Mori, M.; Iwata, K.; Tamaru, Y. *J. Am. Chem. Soc.* **2006**, *128*, 8559-8568.

(21) Ayrey, P. M.; Bolton, M. A.; Buss, A. D.; Greeves, N.; Levin, D.; Wallace, P.; Warren, S. *J. Chem. Soc., Perkin Trans. 1* **1992**, 3407-3417.

(22) Bull, J. A.; Charette, A. B. *J. Am. Chem. Soc.* **2010**, *132*, 1895-1902.

(23) Funahashi, M.; Sonoda, A. *Org. Electron.* **2012**, *13*, 1633-1640.

(24) Bothwell, J. M.; Angeles, V. V.; Carolan, J. P.; Olson, M. E.; Mohan, R. S. *Tetrahedron Lett.* **2010**, *51*, 1056-1058.

(25) Shao, Y.; Yang, C.; Gui, W.; Liu, Y.; Xia, W. *Chem. Commun.* **2012**, *48*, 3560-3562.

(26) Trost, B. M.; Shi, Y. *J. Am. Chem. Soc.* **1993**, *115*, 9421-9438.

(27) Tsimelzon, A.; Braslau, R. *J. Org. Chem.* **2005**, *70*, 10854-10859.

(28) Zhou, M.-B.; Song, R.-J.; Wang, C.-Y.; Li, J.-H. *Angew. Chem. Int. Ed.* **2013**, *52*, 10805-10808.

(29) Rönn, M.; Bäckvall, J.-E.; Andersson, P. G. *Tetrahedron Lett.* **1995**, *36*, 7749-7752.

(30) Cochet, T.; Bellosta, V.; Roche, D.; Ortholand, J.-Y.; Greiner, A.; Cossy, J. *Chem. Commun.* **2012**, *48*, 10745-10747.

(31) Larock, R. C.; Yang, H.; Weinreb, S. M.; Herr, R. J. *J. Org. Chem.* **1994**, *59*, 4172-4178.

(32) Gallagher, T.; Jones, S. W.; Mahon, M. F.; Molloy, K. C. *J. Chem. Soc., Perkin Trans. 1* **1991**, 2193-2198.

^{1}H and ^{13}C NMR spectra of novel compounds

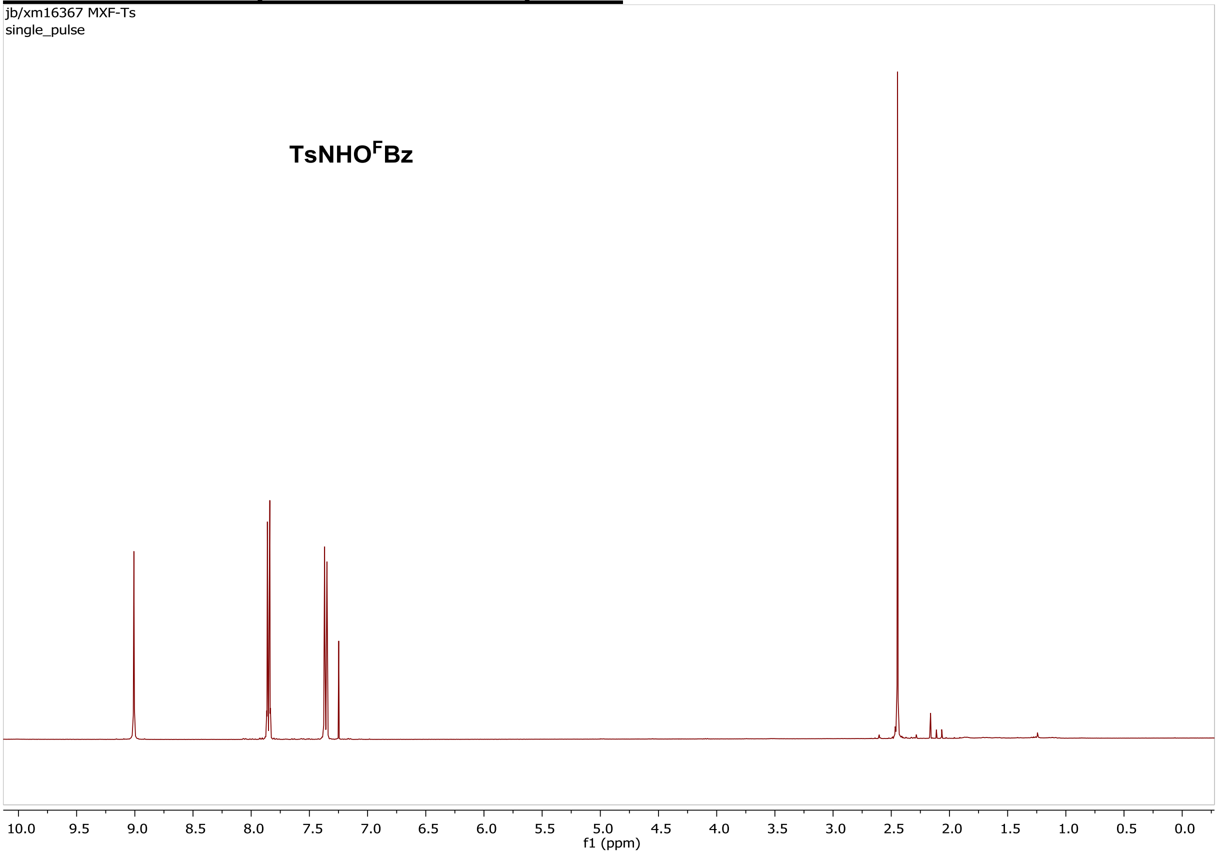

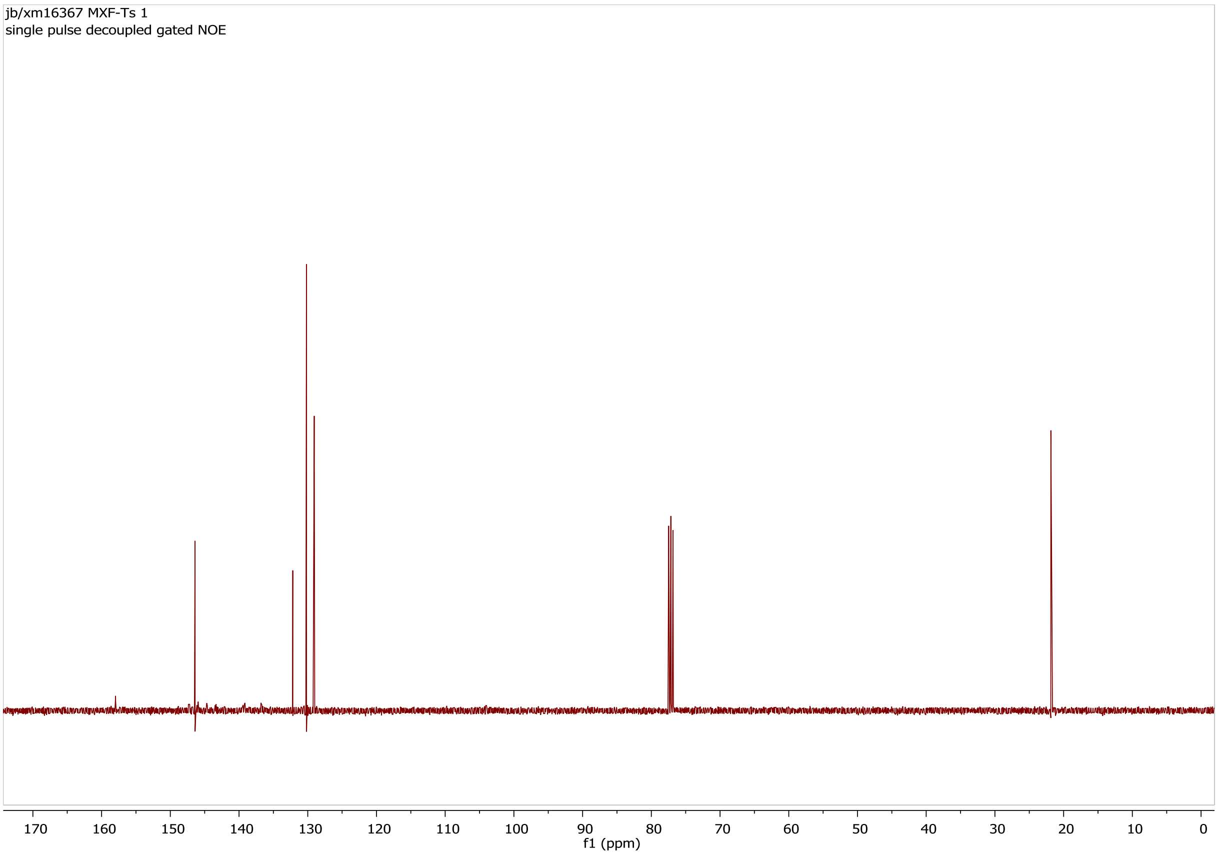

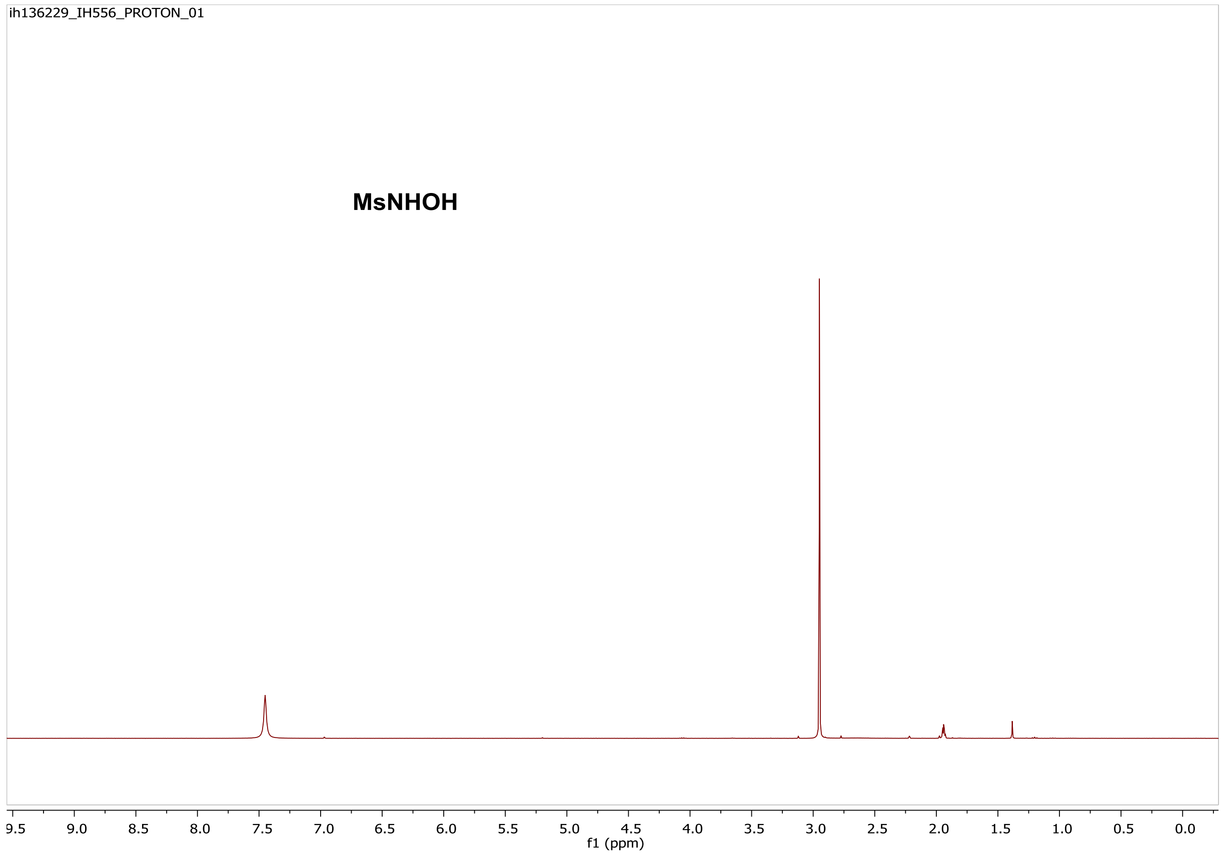
ih136229_IH556_PROTON_01
MsNHOH
9.5
9.0
8.5
8.0
7.5
7.0
6.5
6.0
5.5
5.0
4.5
4.0
3.5
3.0
2.5
2.0
1.5
1.0
0.5
0.0
f1 (ppm)

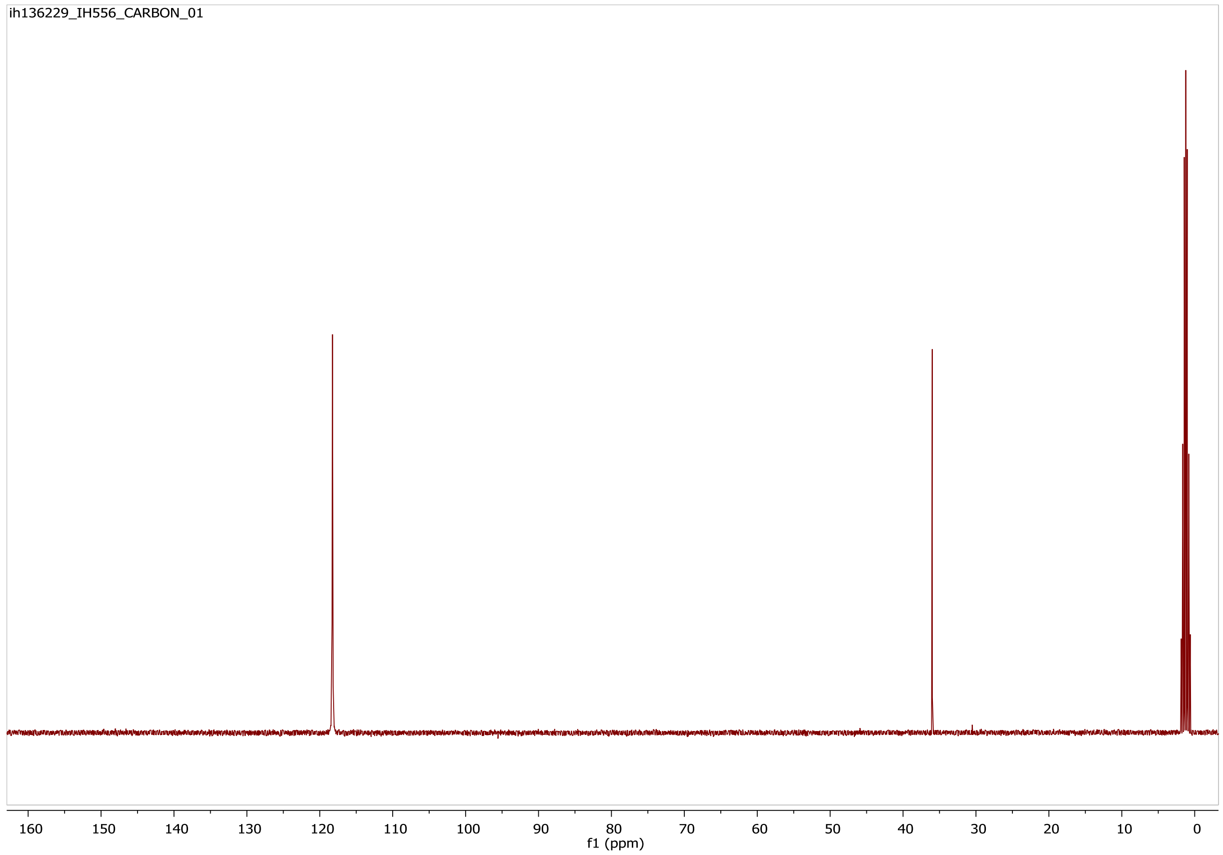
ih136229_IH556_CARBON_01
160
150
140
130
120
110
100
90
80
70
60
50
40
30
20
10
0
f1 (ppm)

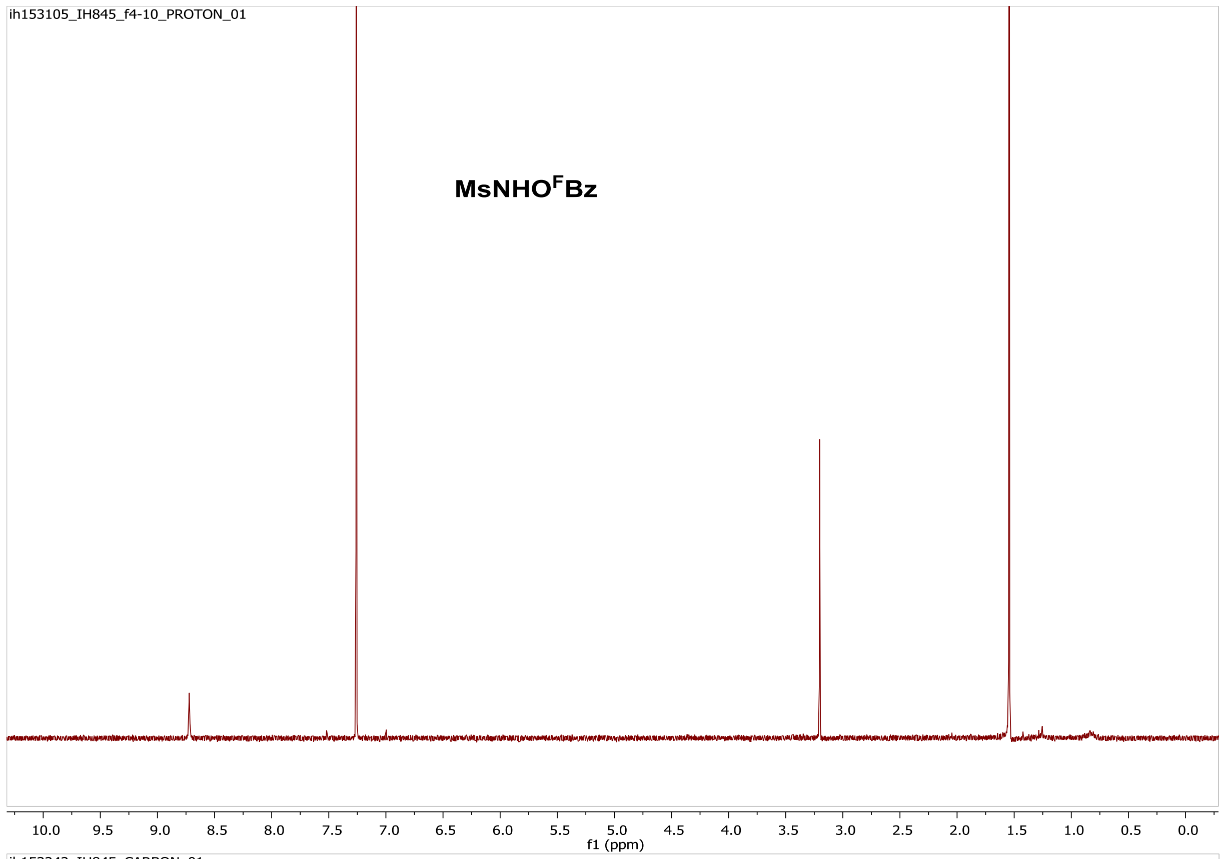
ih153105_IH845_f4-10_PROTON_01
MsNHO^{F}Bz
10.0 9.5 9.0 8.5 8.0 7.5 7.0 6.5 6.0 5.5 5.0 4.5 4.0 3.5 3.0 2.5 2.0 1.5 1.0 0.5 0.0
f1 (ppm)

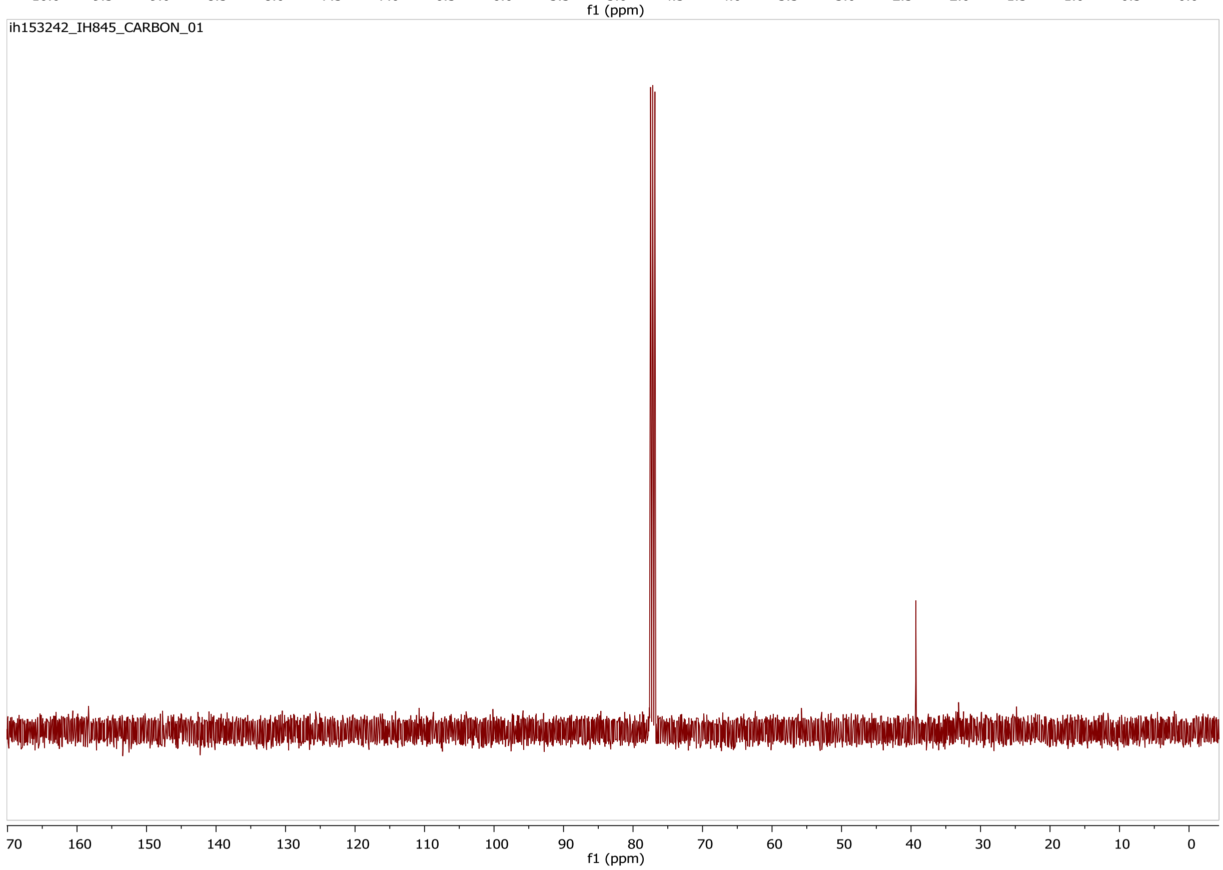
ih153242_IH845_CARBON_01
70 160 150 140 130 120 110 100 90 80 70 60 50 40 30 20 10 0
f1 (ppm)

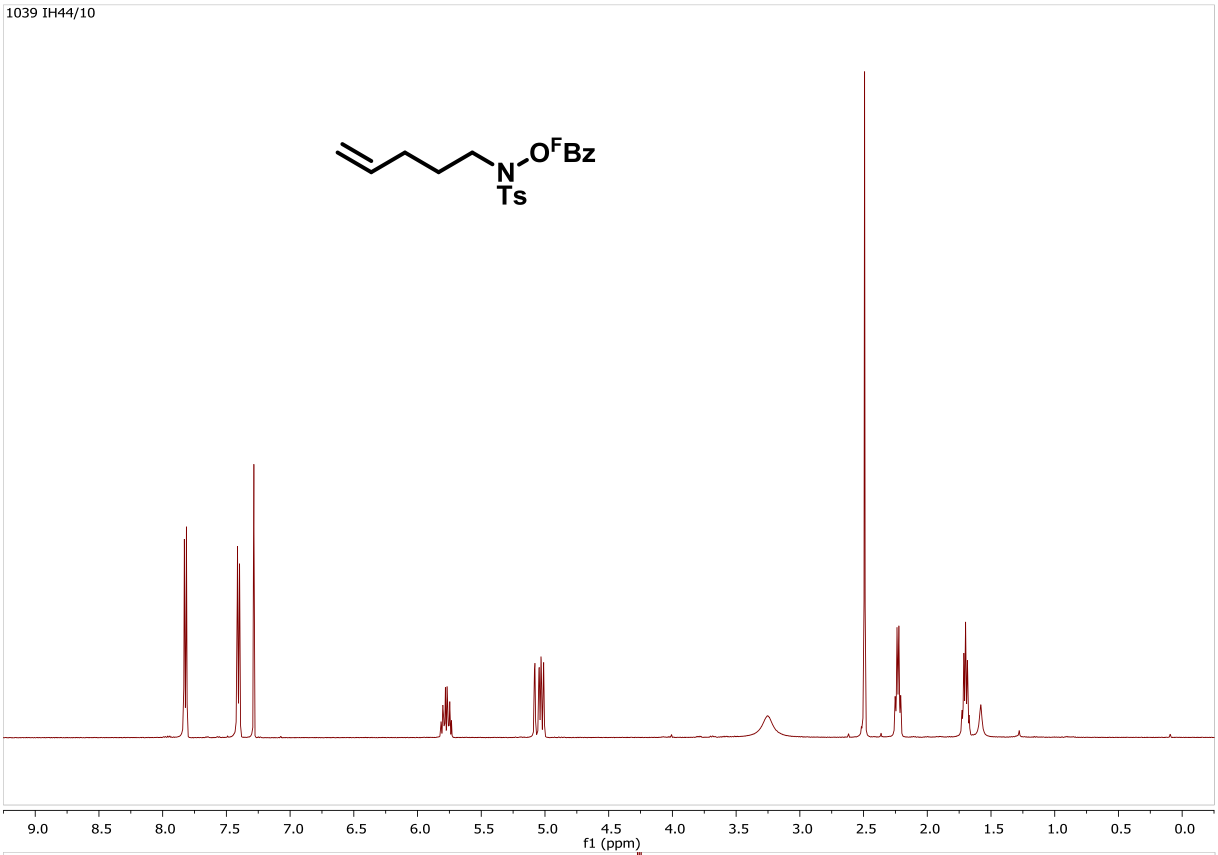
1039 IH44/10
N
O
F
Bz
Ts
9.0
8.5
8.0
7.5
7.0
6.5
6.0
5.5
5.0
4.5
4.0
3.5
3.0
2.5
2.0
1.5
1.0
0.5
0.0
f1 (ppm)

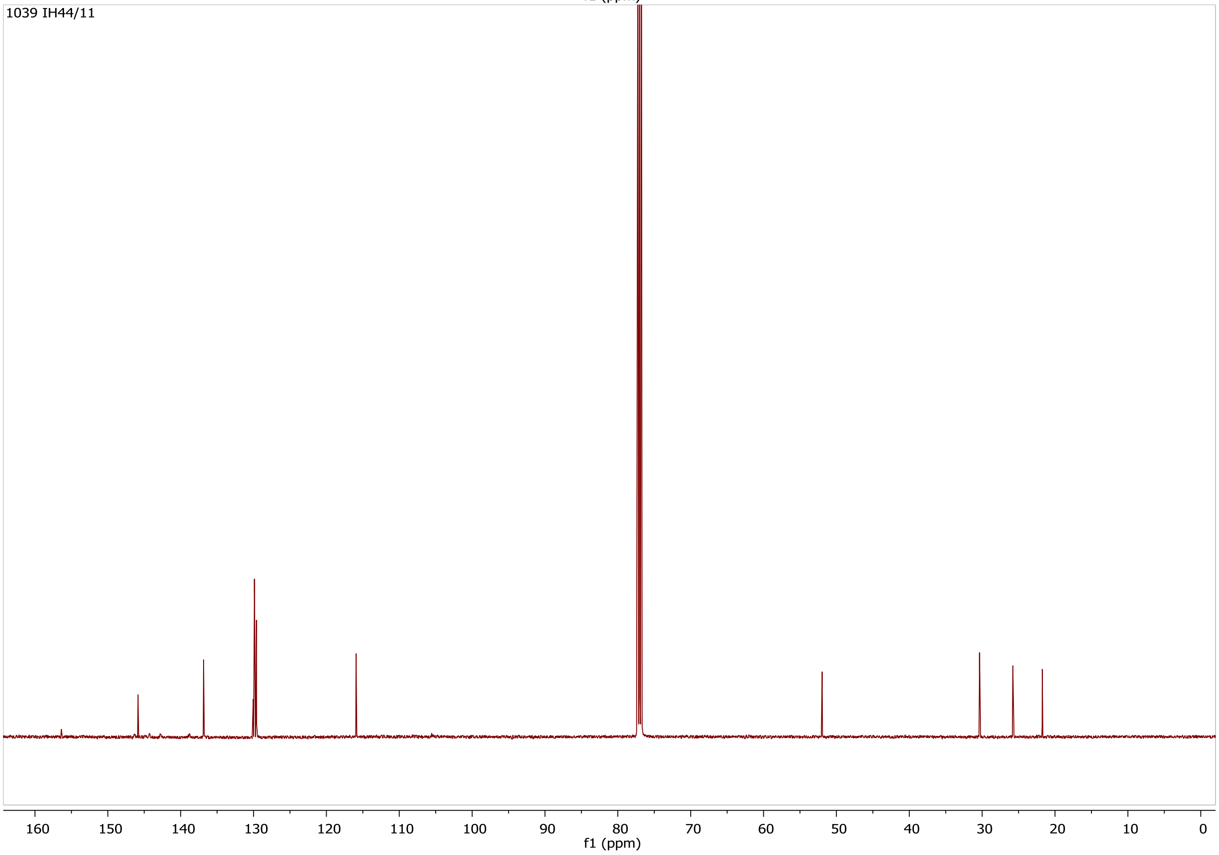
1039 IH44/11
160
150
140
130
120
110
100
90
80
70
60
50
40
30
20
10
0
f1 (ppm)

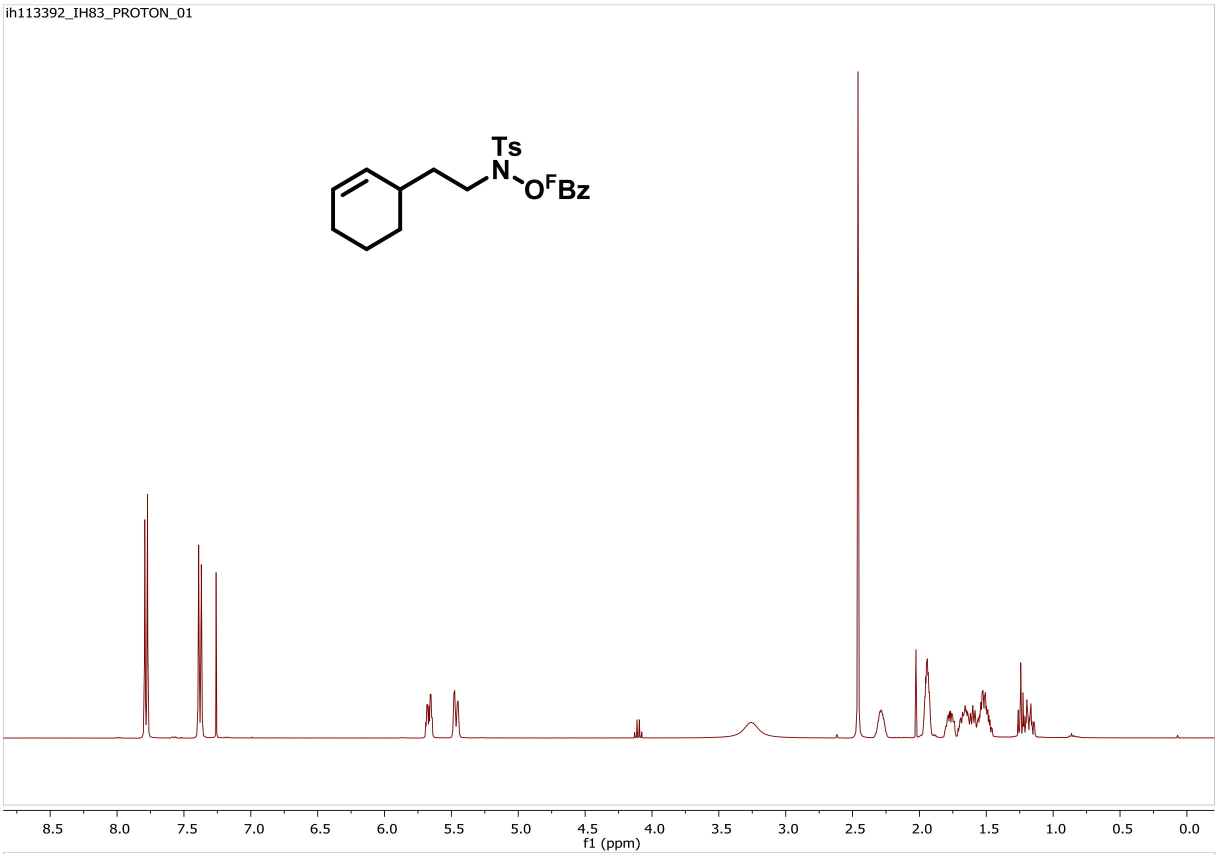
ih113392_IH83_PROTON_01
Ts
N
O^F Bz
8.5 8.0 7.5 7.0 6.5 6.0 5.5 5.0 4.5 4.0 3.5 3.0 2.5 2.0 1.5 1.0 0.5 0.0
f1 (ppm)

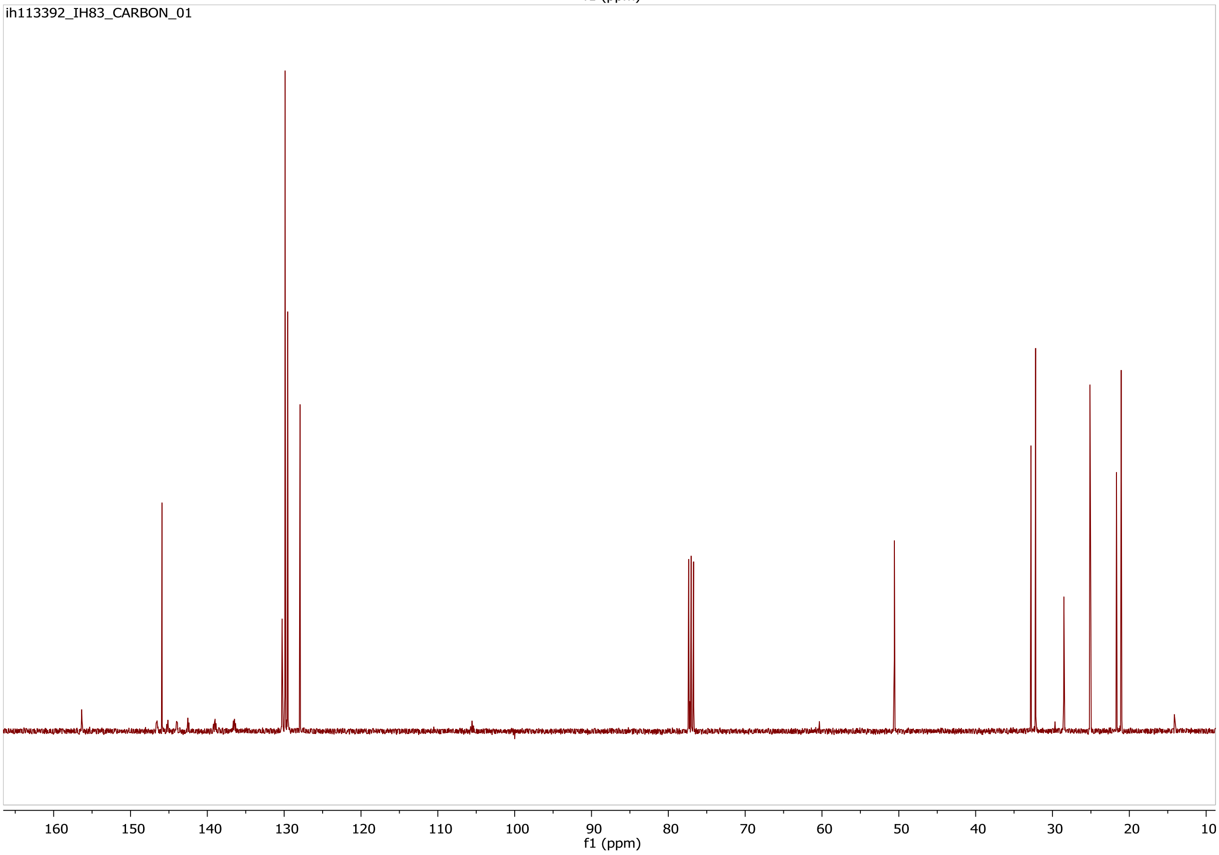
ih113392_IH83_CARBON_01
160 150 140 130 120 110 100 90 80 70 60 50 40 30 20 10
f1 (ppm)

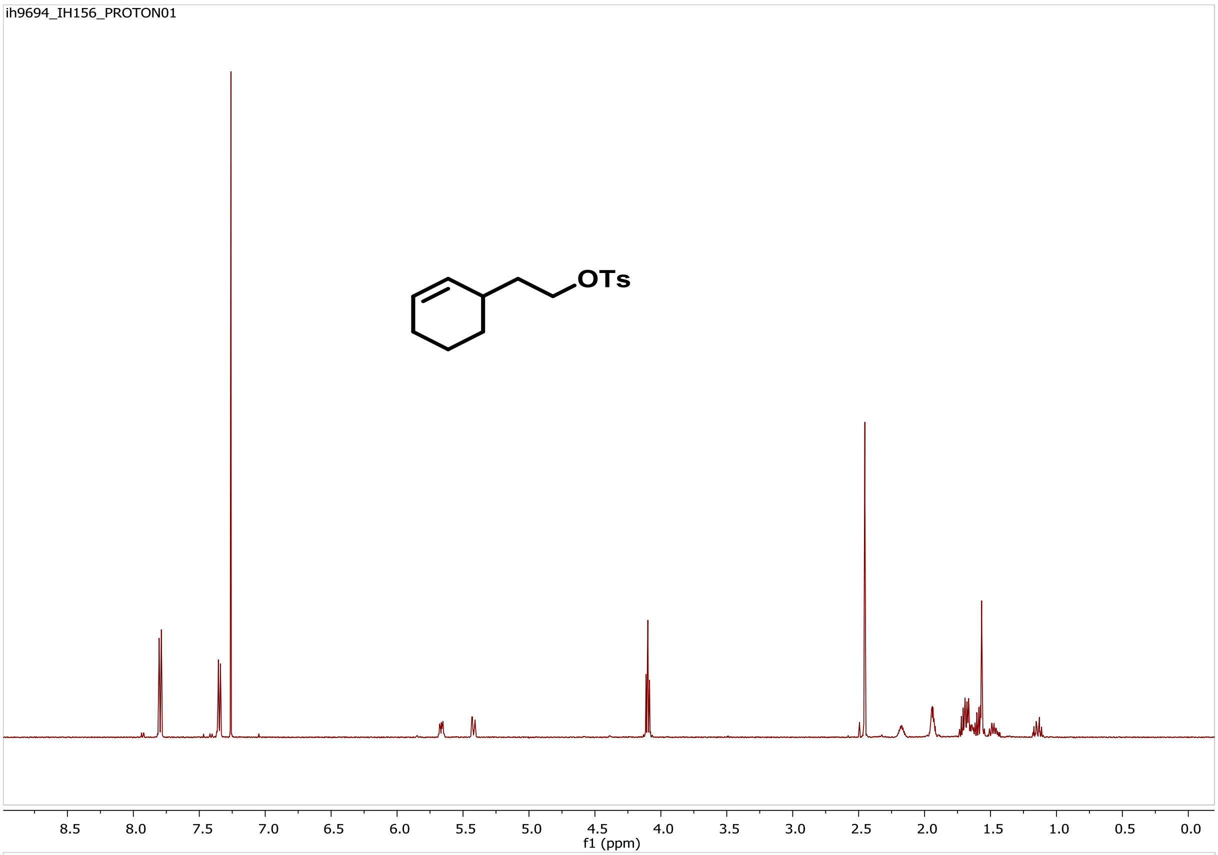
ih9694_IH156_PROTON01
OTs
8.5 8.0 7.5 7.0 6.5 6.0 5.5 5.0 4.5 4.0 3.5 3.0 2.5 2.0 1.5 1.0 0.5 0.0
f1 (ppm)

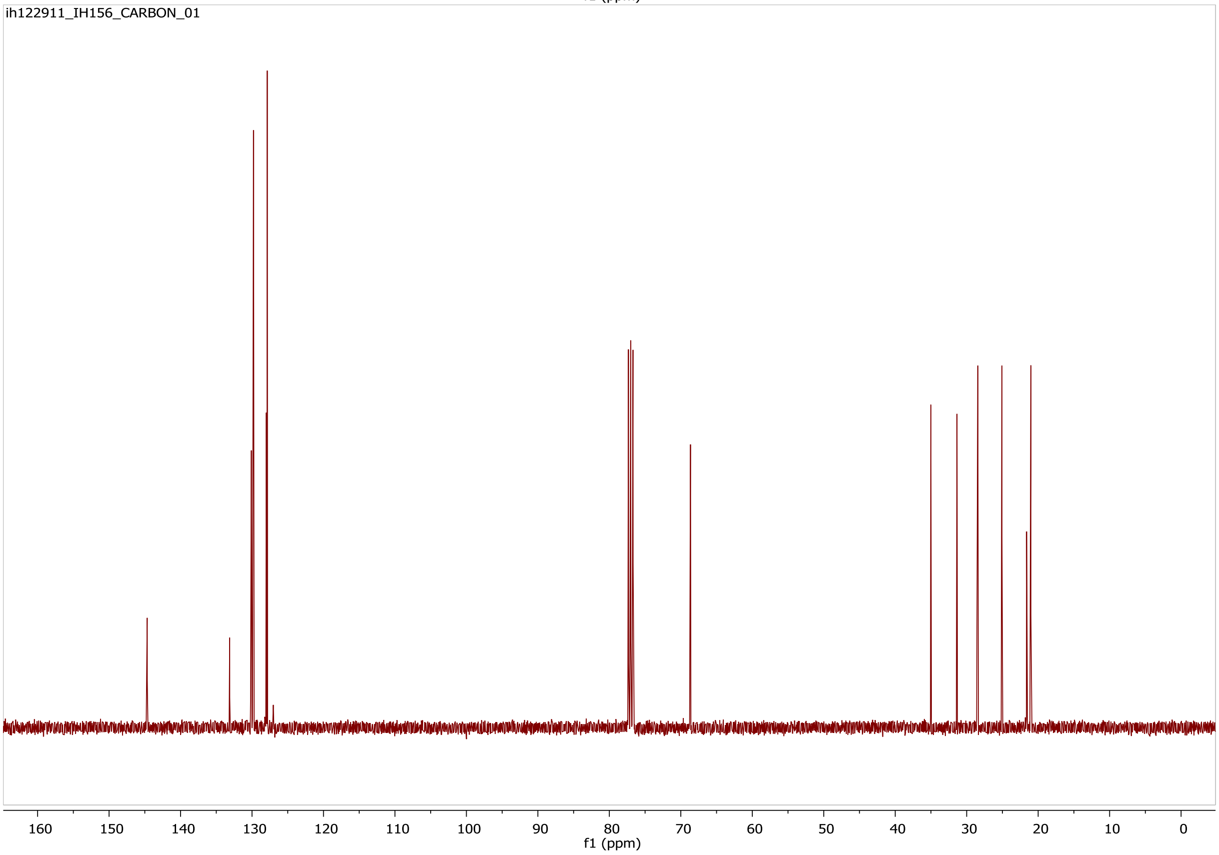
ih122911_IH156_CARBON_01
160 150 140 130 120 110 100 90 80 70 60 50 40 30 20 10 0
f1 (ppm)

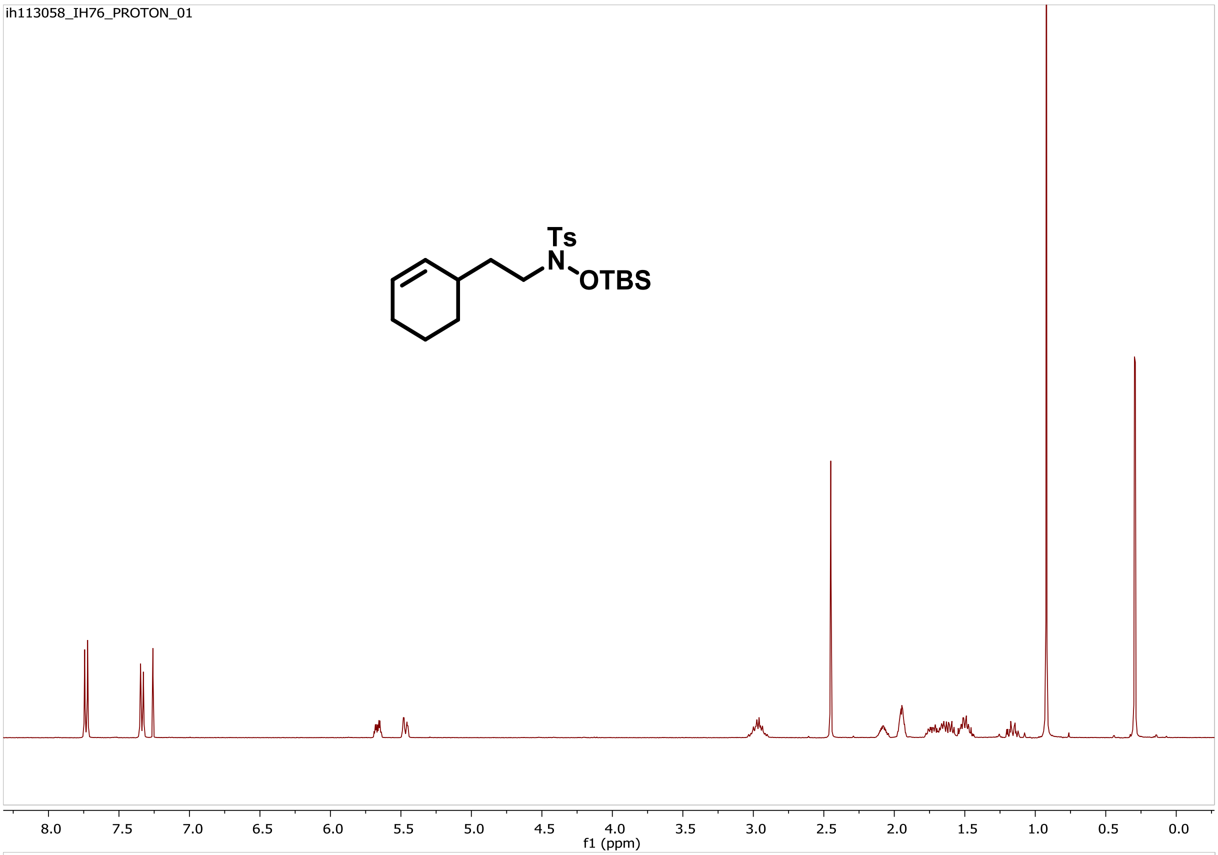
ih113058_IH76_PROTON_01
Ts
N
OTBS
8.0 7.5 7.0 6.5 6.0 5.5 5.0 4.5 4.0 3.5 3.0 2.5 2.0 1.5 1.0 0.5 0.0
f1 (ppm)

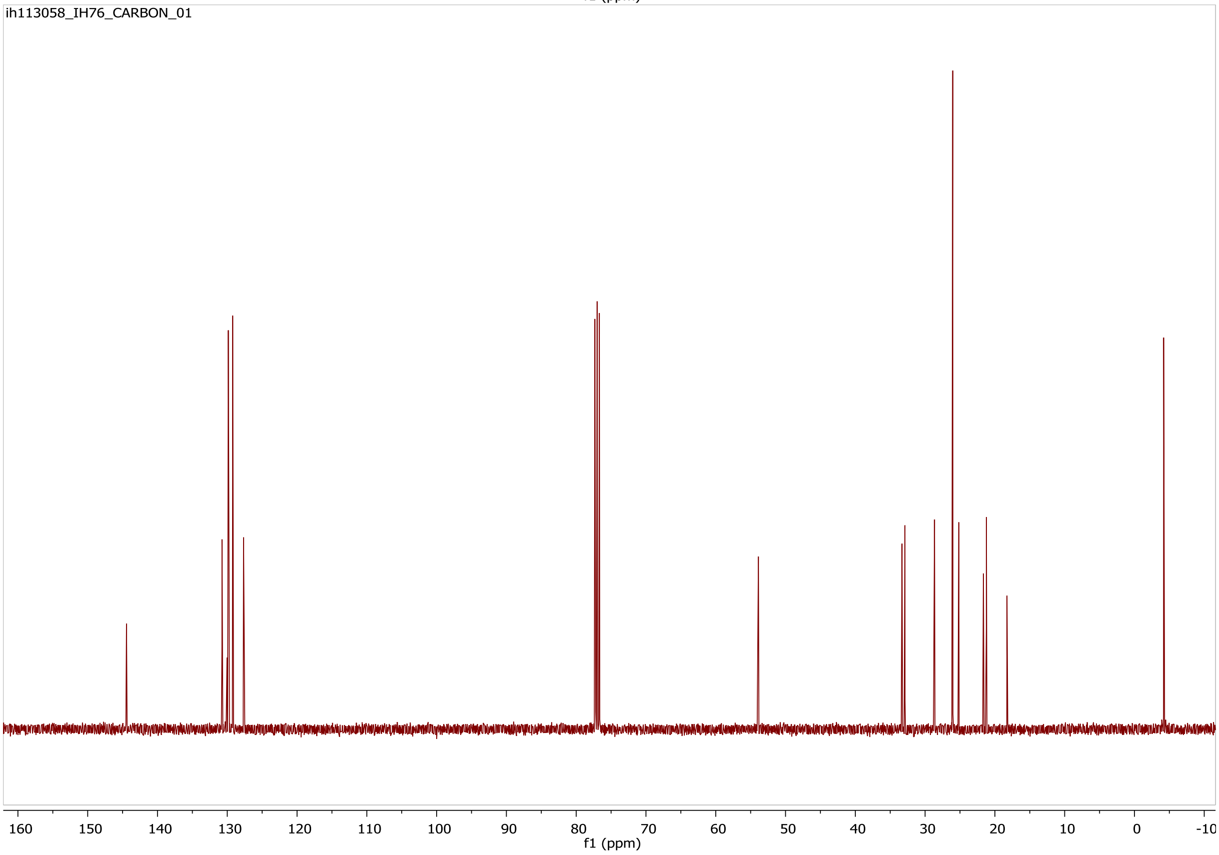
ih113058_IH76_CARBON_01
160 150 140 130 120 110 100 90 80 70 60 50 40 30 20 10 0 -10
f1 (ppm)

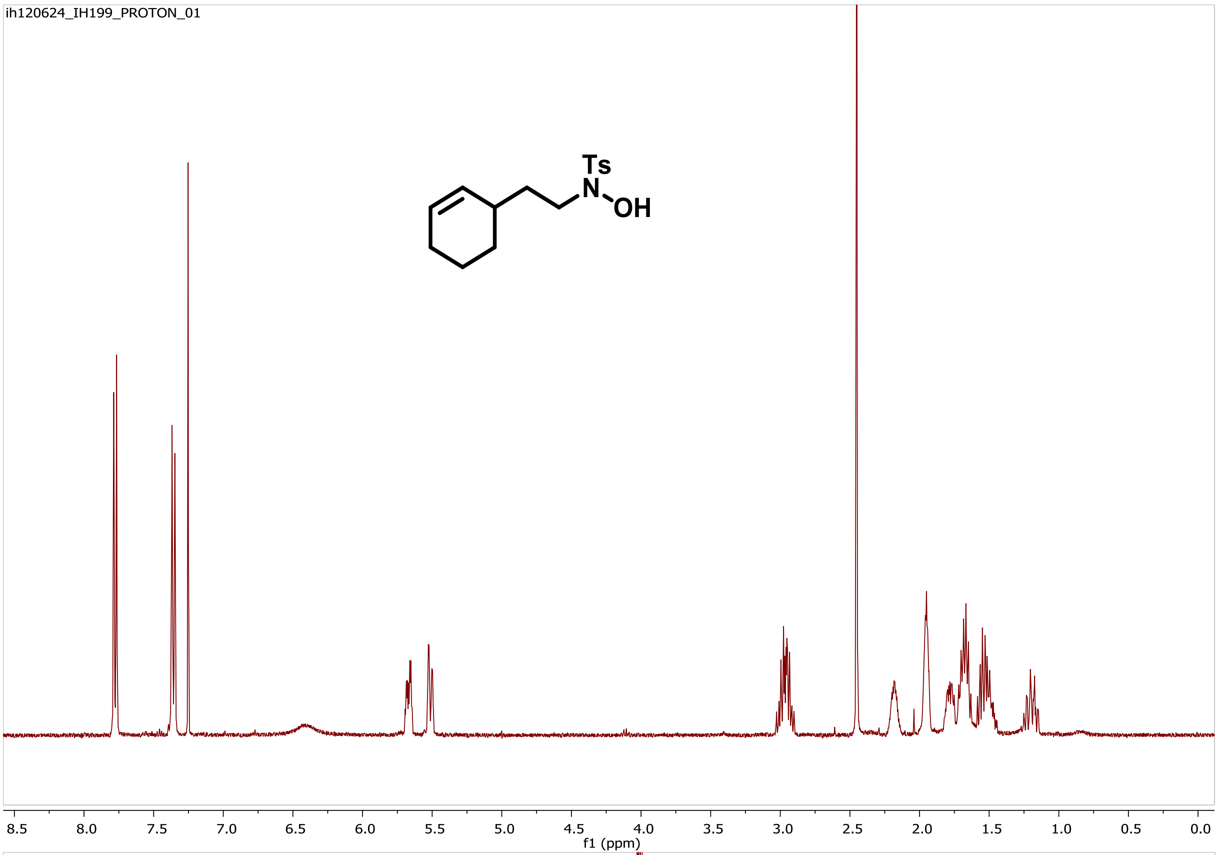
ih120624_IH199_PROTON_01
Ts
N
OH
8.5
8.0
7.5
7.0
6.5
6.0
5.5
5.0
4.5
4.0
3.5
3.0
2.5
2.0
1.5
1.0
0.5
0.0
f1 (ppm)

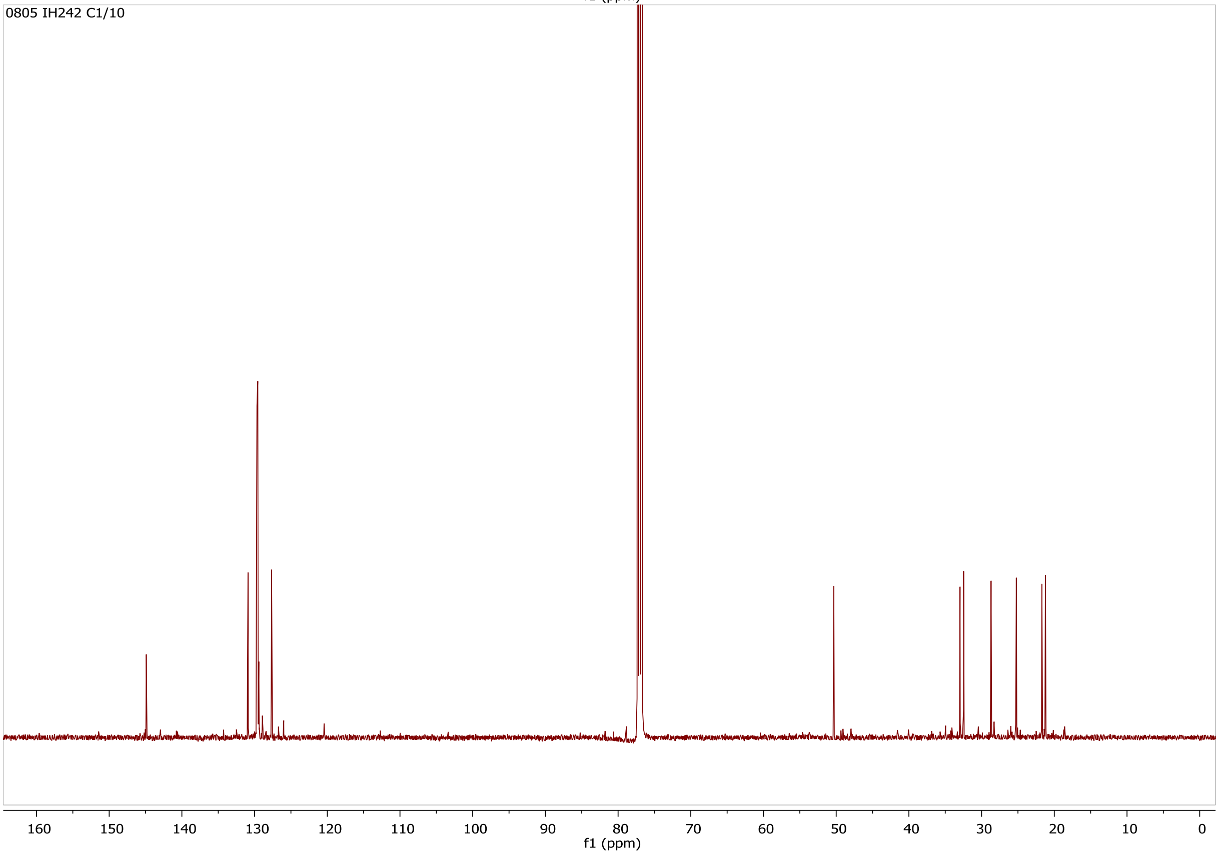
0805 IH242 C1/10
160
150
140
130
120
110
100
90
80
70
60
50
40
30
20
10
0
f1 (ppm)

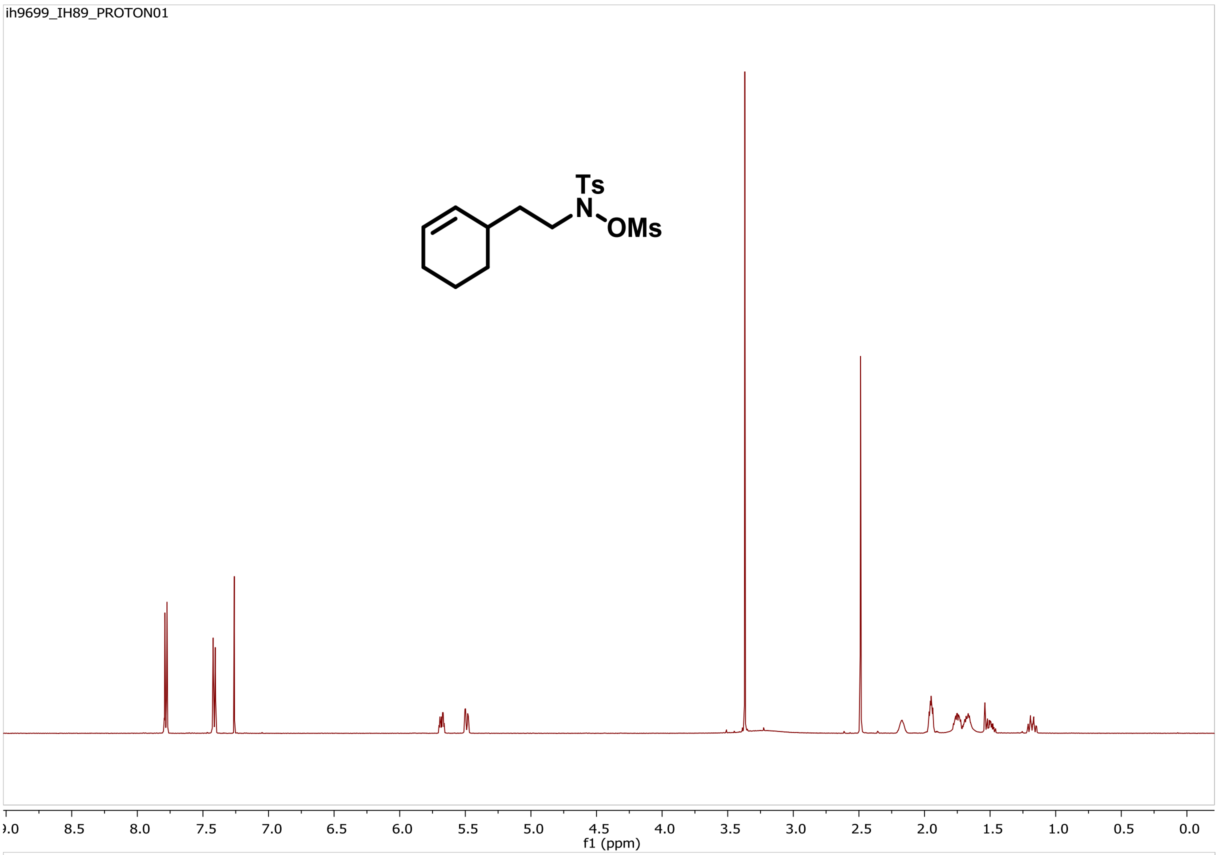
ih9699_IH89_PROTON01
Ts
N
OMs
f1 (ppm)

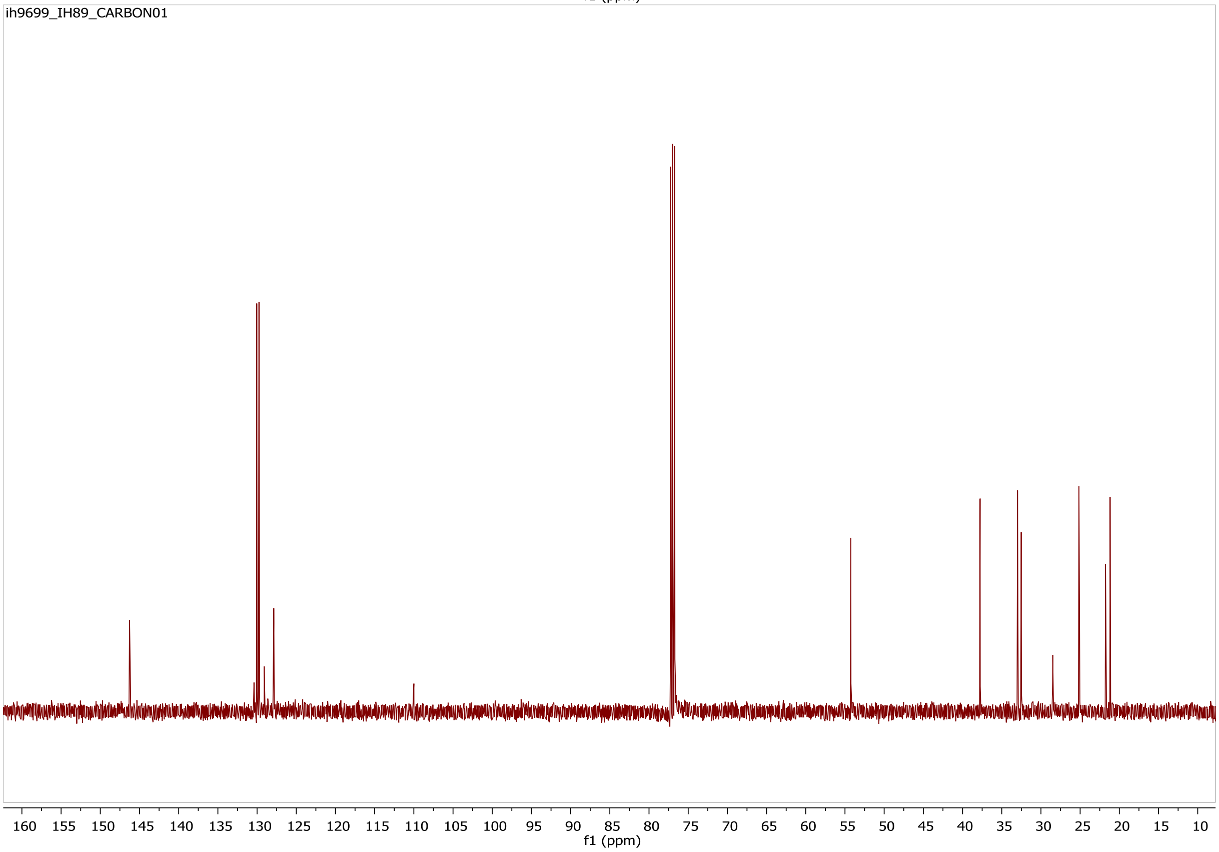
ih9699_IH89_CARBON01
f1 (ppm)

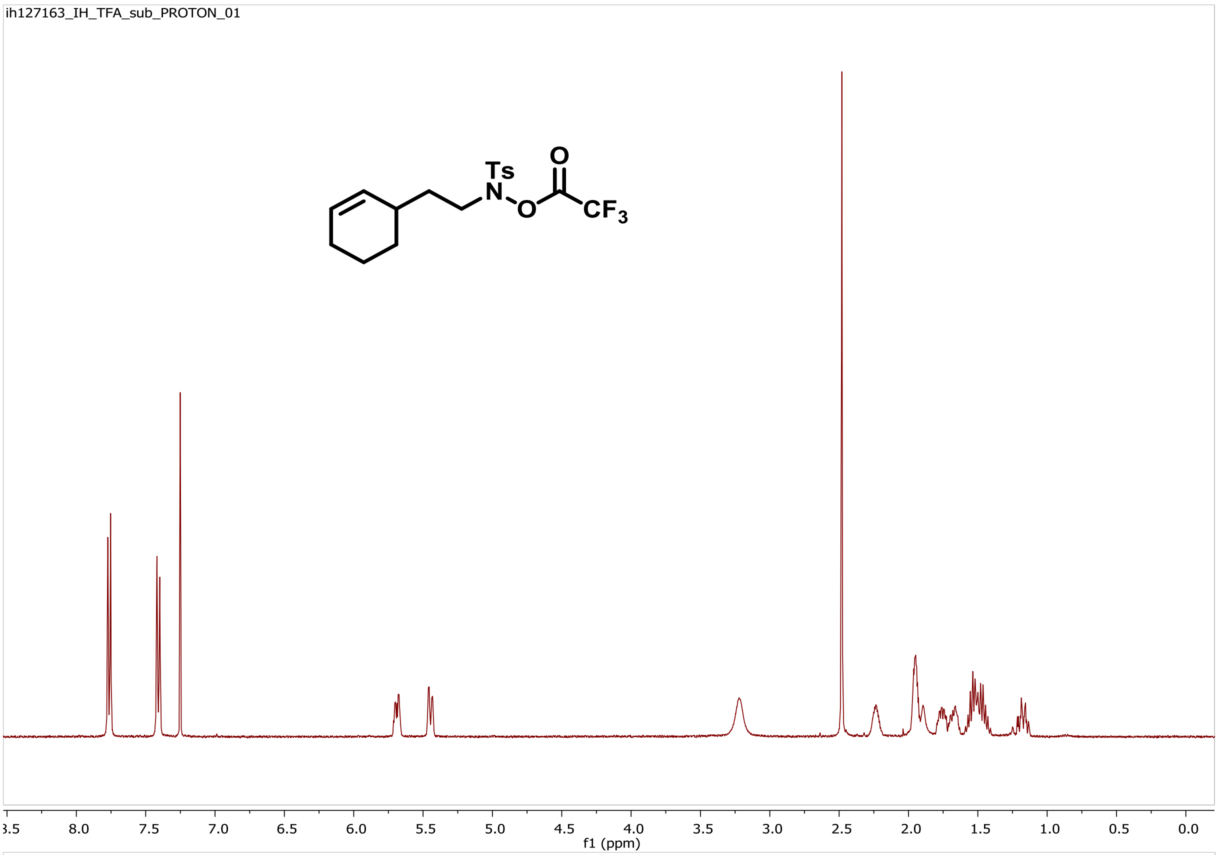
ih127163_IH_TFA_sub_PROTON_01
Ts
N
O
O
CF_3
8.0 7.5 7.0 6.5 6.0 5.5 5.0 4.5 4.0 3.5 3.0 2.5 2.0 1.5 1.0 0.5 0.0
f1 (ppm)

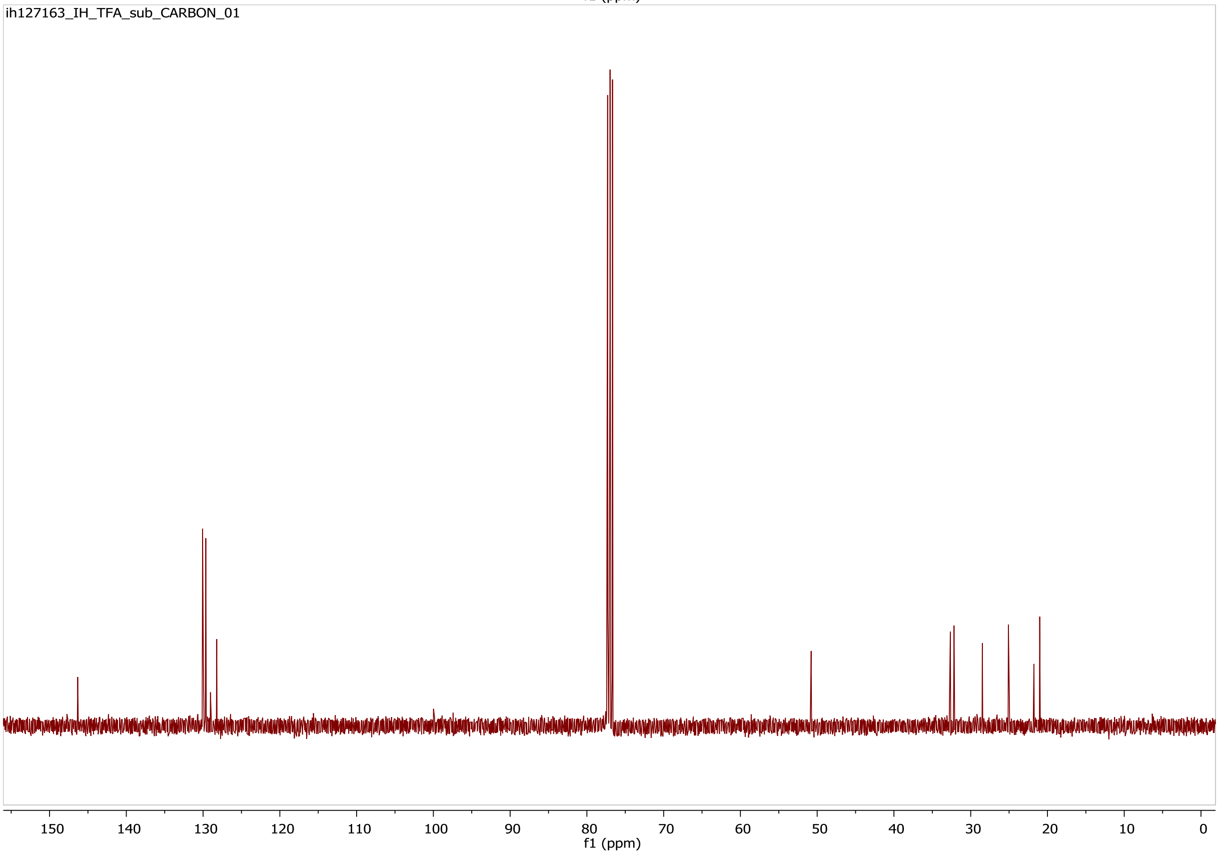
ih127163_IH_TFA_sub_CARBON_01
150 140 130 120 110 100 90 80 70 60 50 40 30 20 10 0
f1 (ppm)

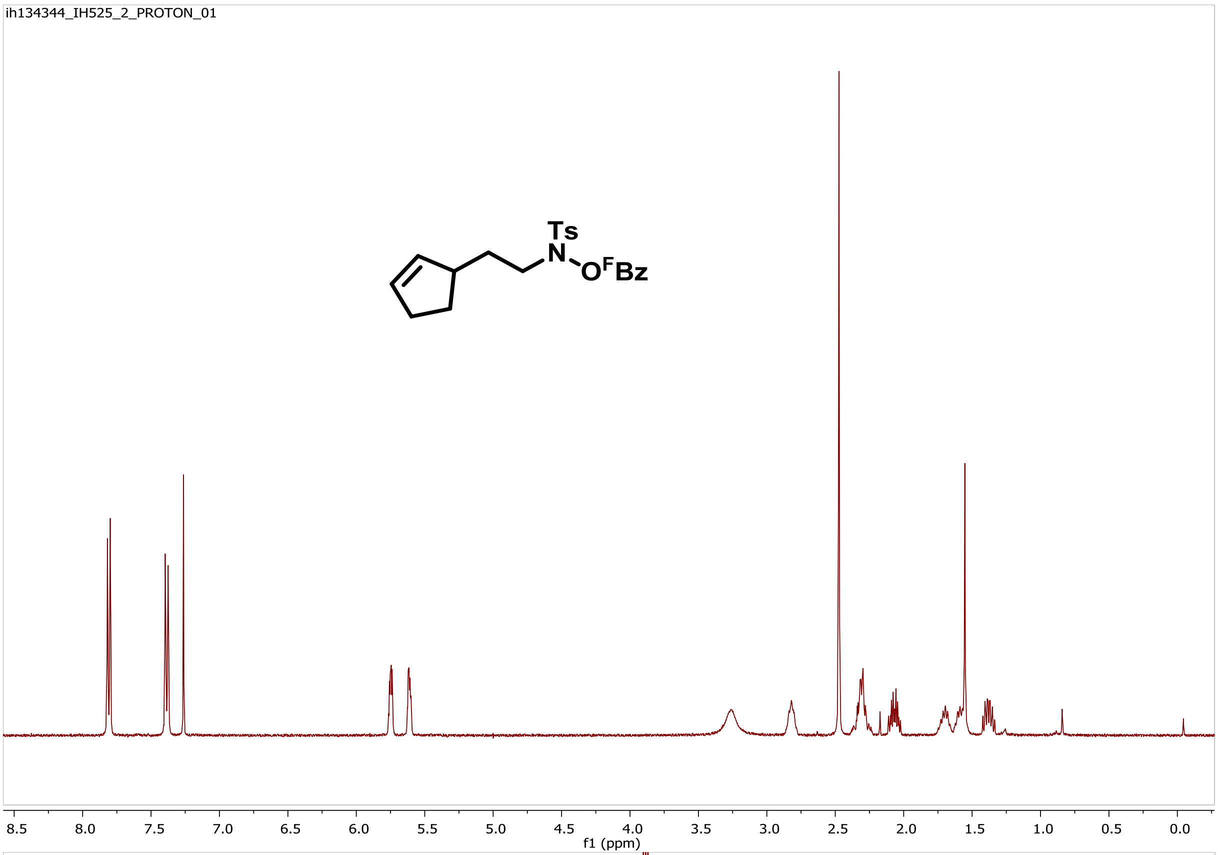
ih134344_IH525_2_PROTON_01
Ts
N
O
F
Bz
8.5 8.0 7.5 7.0 6.5 6.0 5.5 5.0 4.5 4.0 3.5 3.0 2.5 2.0 1.5 1.0 0.5 0.0
f1 (ppm)

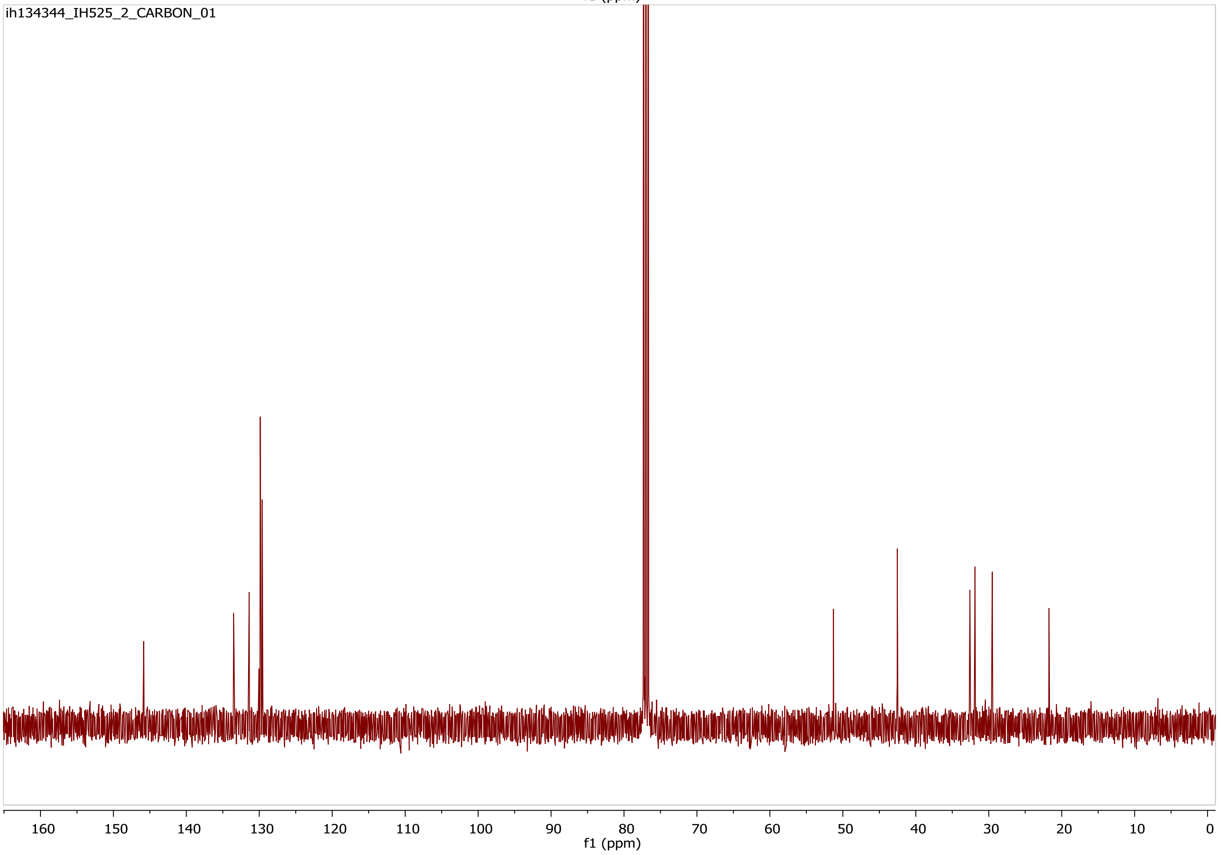
ih134344_IH525_2_CARBON_01
160 150 140 130 120 110 100 90 80 70 60 50 40 30 20 10 0
f1 (ppm)

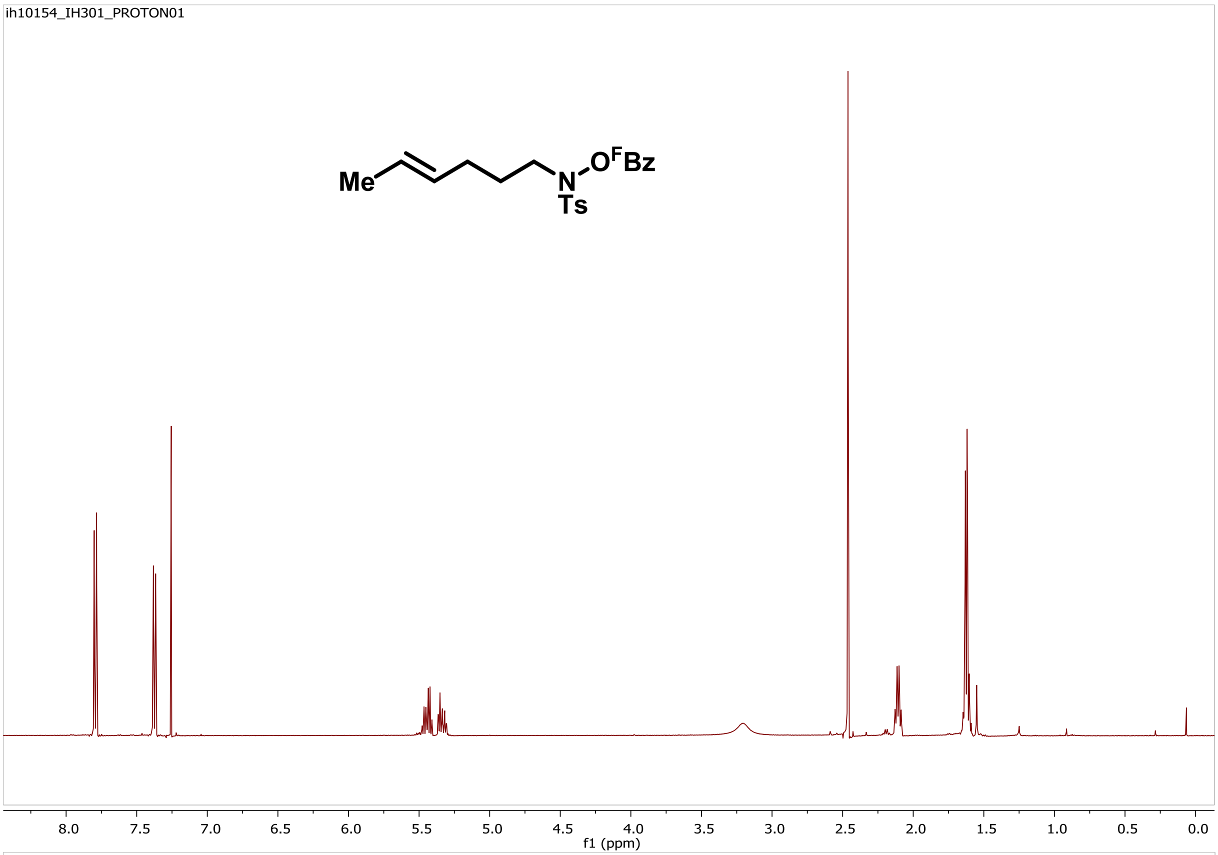
ih10154_IH301_PROTON01
Me
N
O
F
Bz
Ts
8.0
7.5
7.0
6.5
6.0
5.5
5.0
4.5
4.0
3.5
3.0
2.5
2.0
1.5
1.0
0.5
0.0
f1 (ppm)

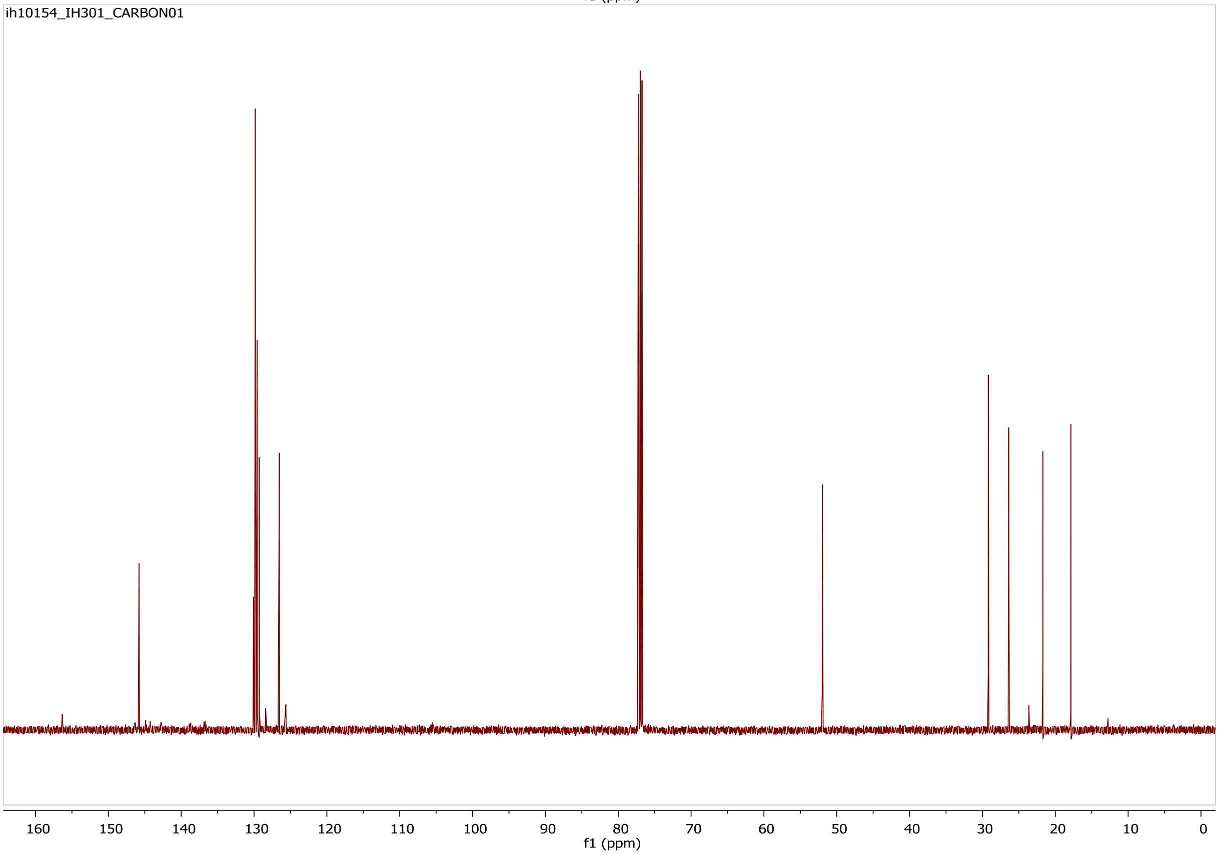
ih10154_IH301_CARBON01
160
150
140
130
120
110
100
90
80
70
60
50
40
30
20
10
0
f1 (ppm)

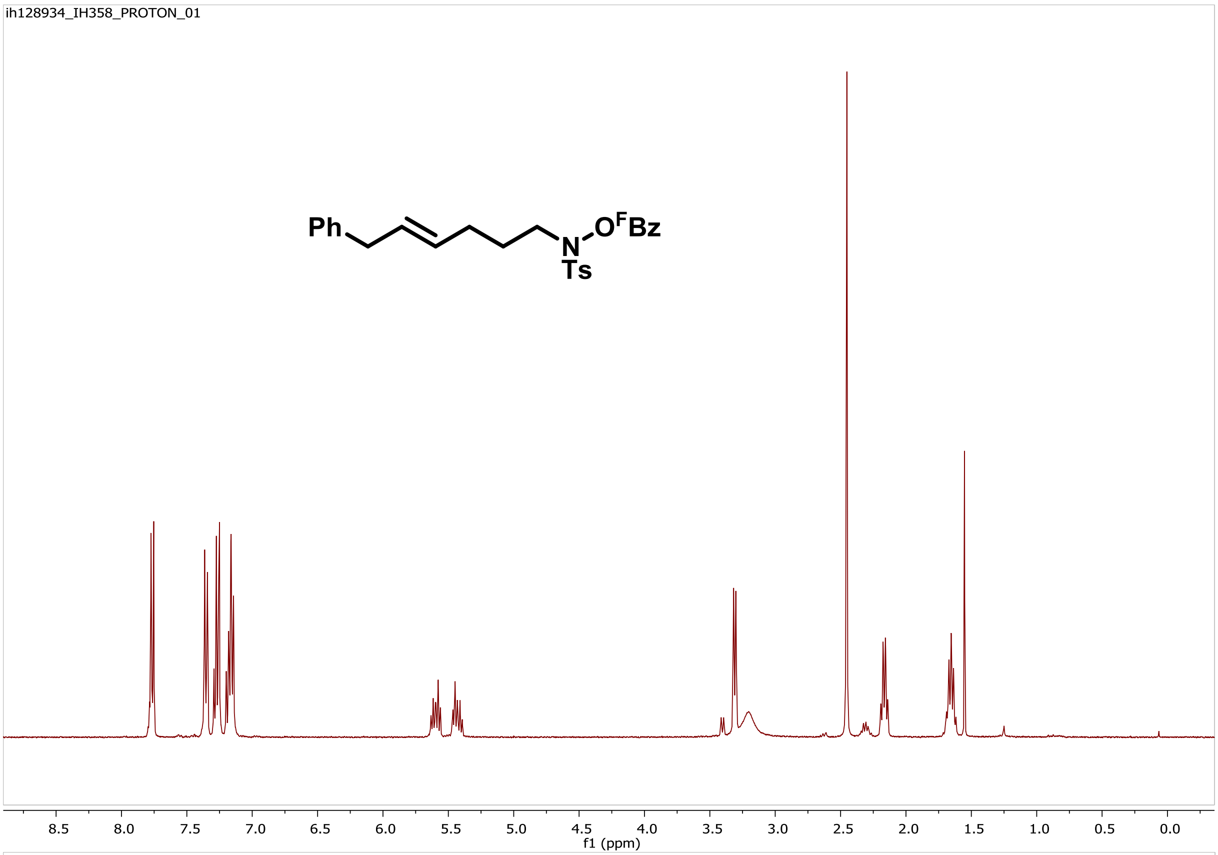
ih128934_IH358_PROTON_01
Ph
N
O
F
Bz
Ts
8.5
8.0
7.5
7.0
6.5
6.0
5.5
5.0
4.5
4.0
3.5
3.0
2.5
2.0
1.5
1.0
0.5
0.0
f1 (ppm)

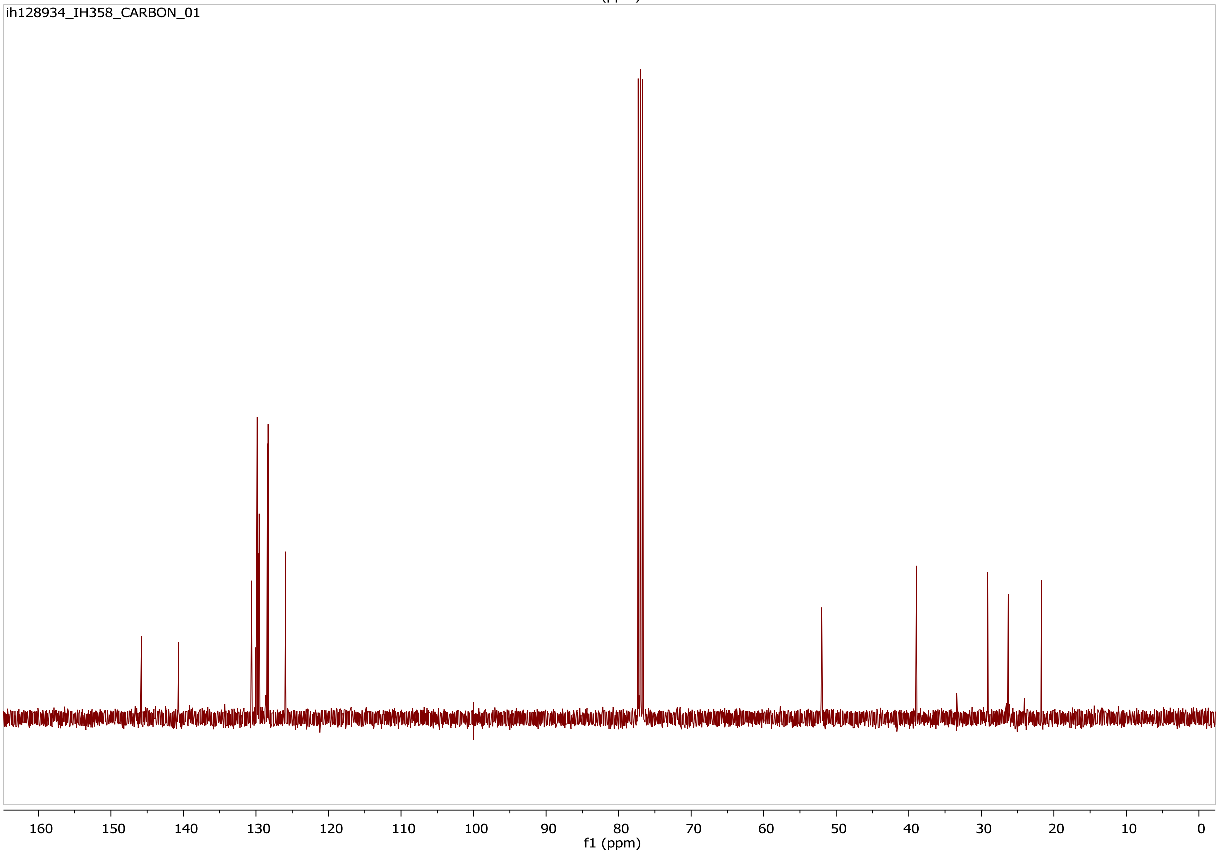
ih128934_IH358_CARBON_01
160
150
140
130
120
110
100
90
80
70
60
50
40
30
20
10
0
f1 (ppm)

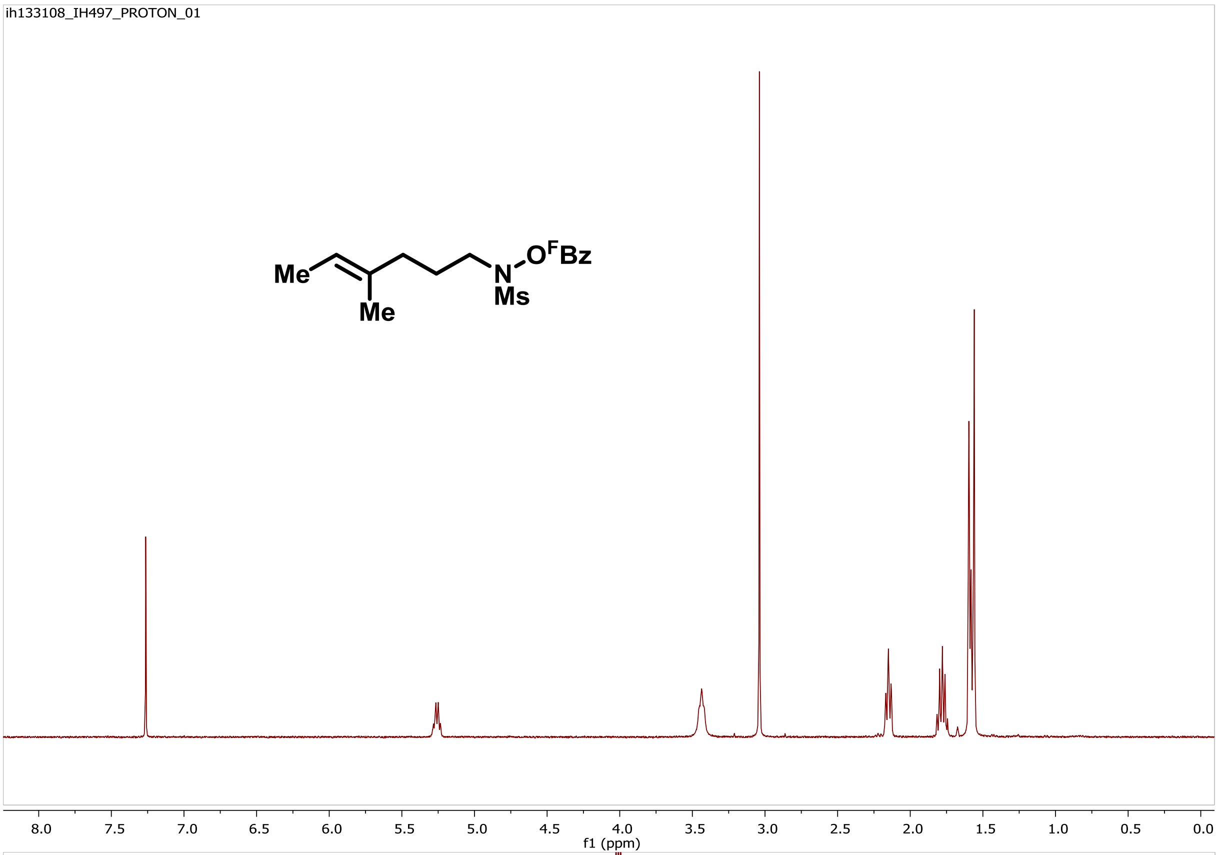
ih133108_IH497_PROTON_01
Me
Me
N
O
F
Bz
Ms
8.0
7.5
7.0
6.5
6.0
5.5
5.0
4.5
4.0
3.5
3.0
2.5
2.0
1.5
1.0
0.5
0.0
f1 (ppm)

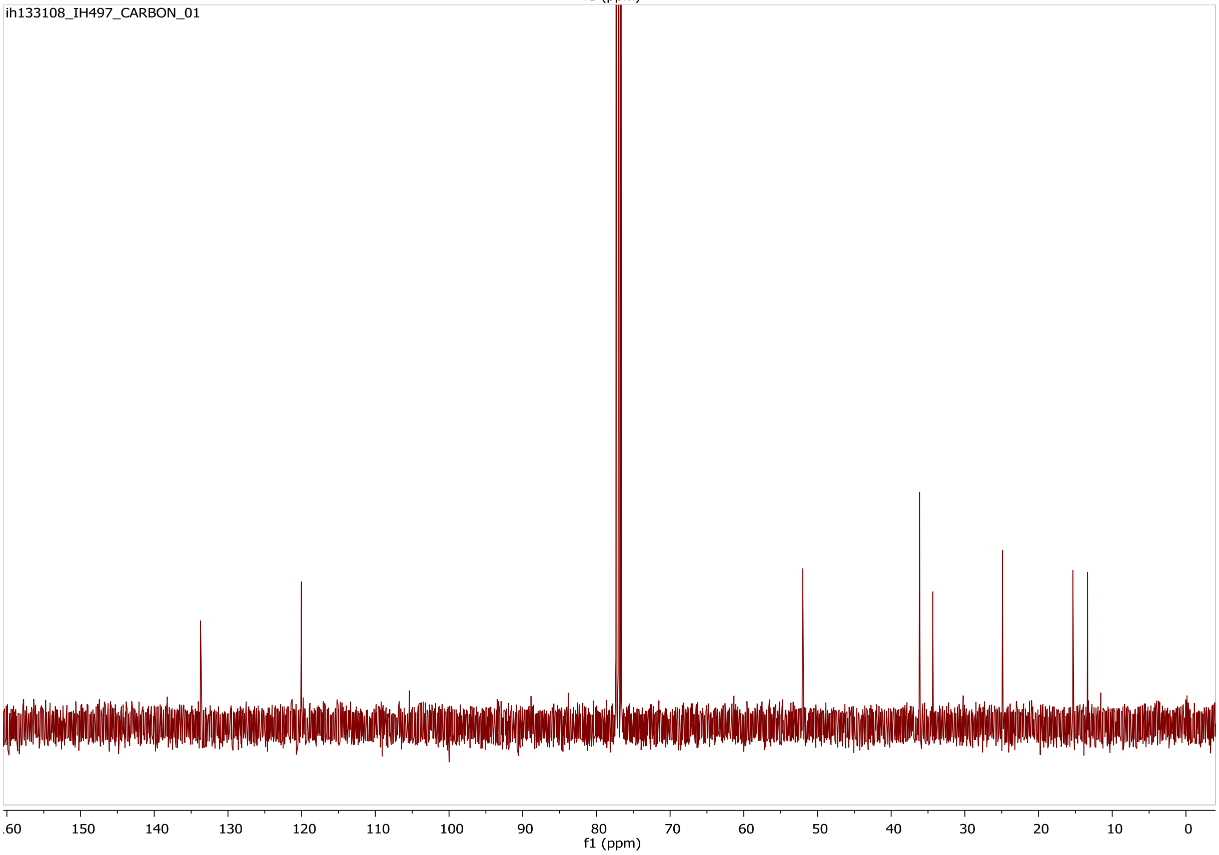
ih133108_IH497_CARBON_01
150
140
130
120
110
100
90
80
70
60
50
40
30
20
10
0
f1 (ppm)

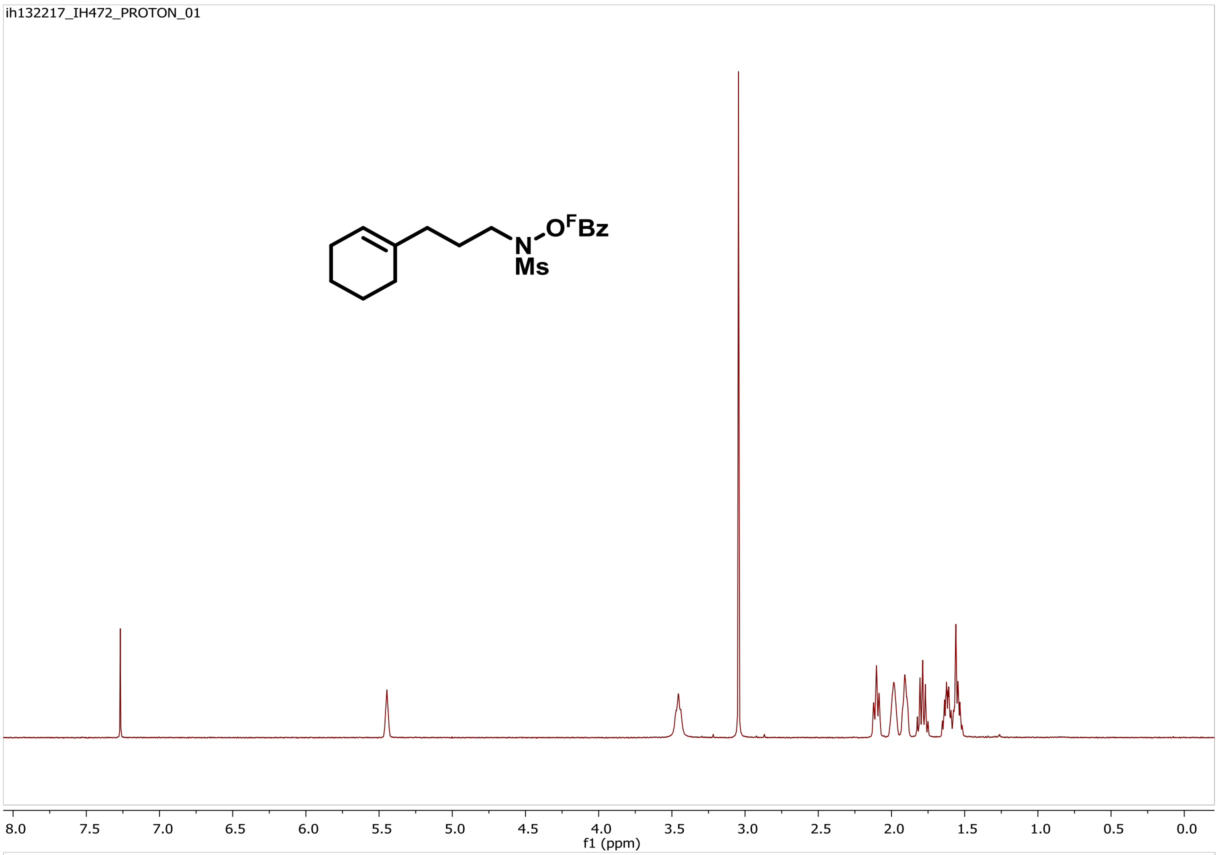
ih132217_IH472_PROTON_01
N
O
F
Bz
Ms
f1 (ppm)

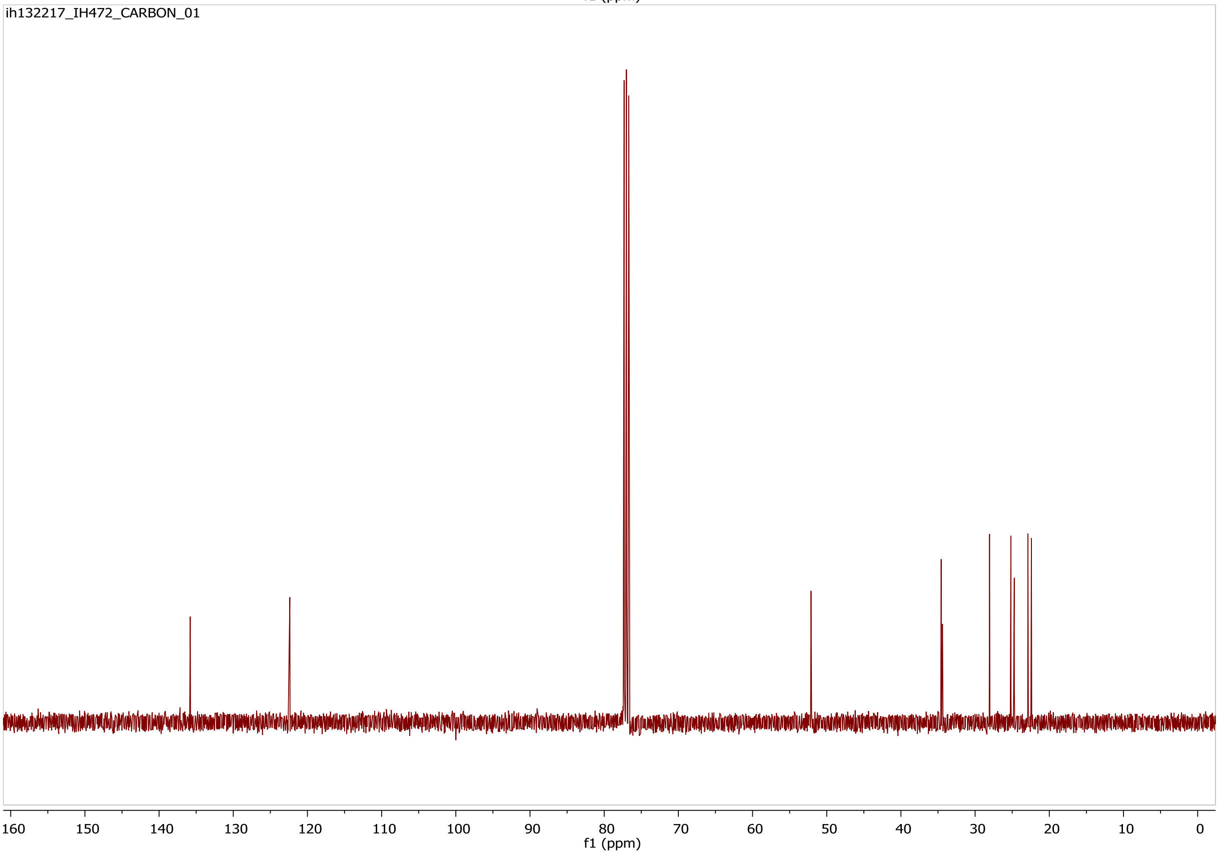
ih132217_IH472_CARBON_01
f1 (ppm)

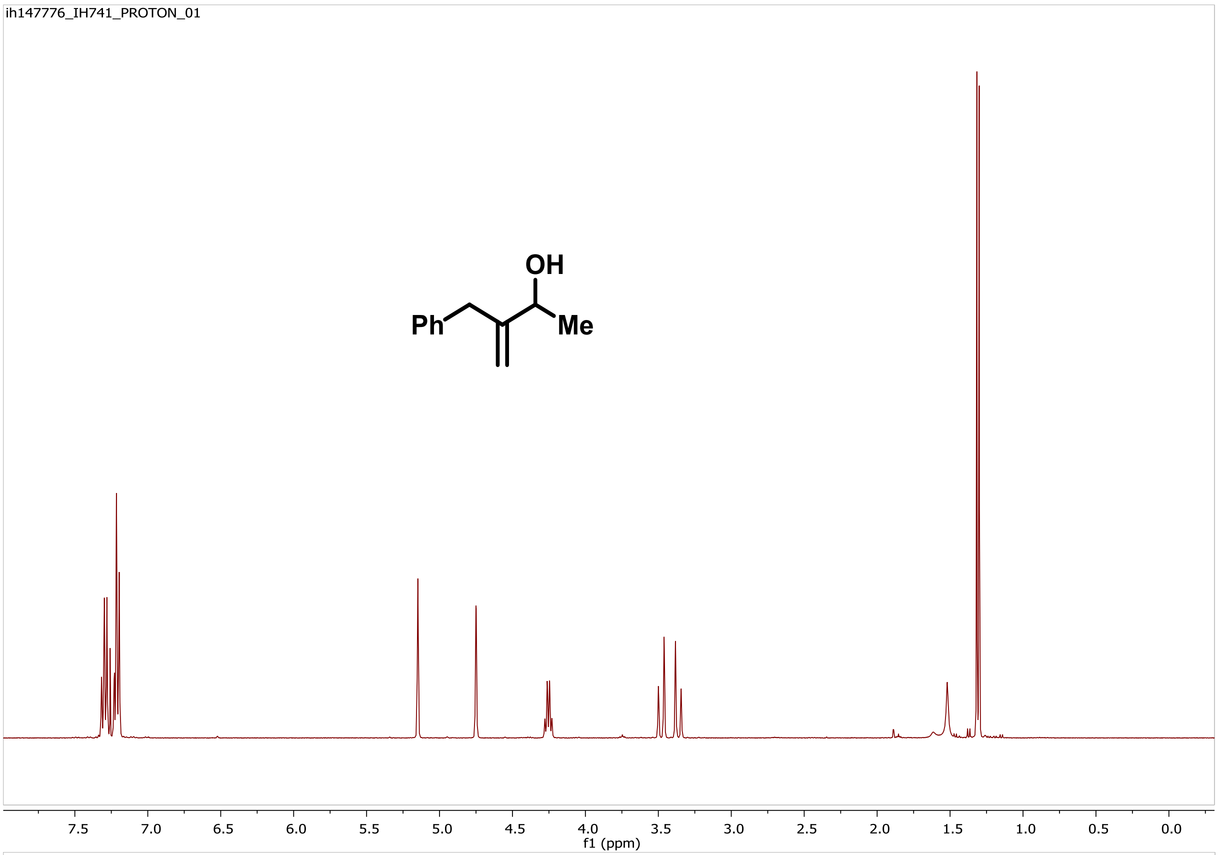
ih147776_IH741_PROTON_01
OH
Ph
Me
7.5
7.0
6.5
6.0
5.5
5.0
4.5
4.0
3.5
3.0
2.5
2.0
1.5
1.0
0.5
0.0
f1 (ppm)

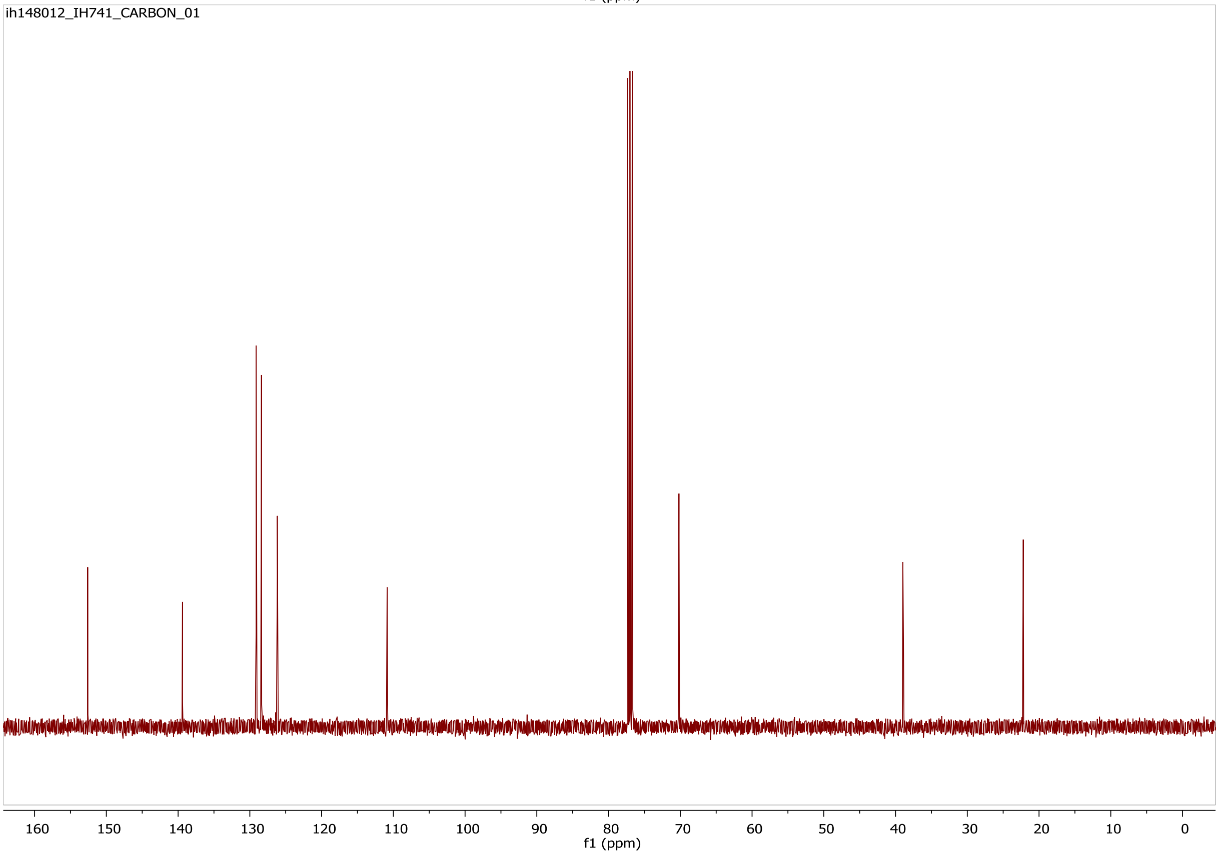
ih148012_IH741_CARBON_01
160
150
140
130
120
110
100
90
80
70
60
50
40
30
20
10
0
f1 (ppm)

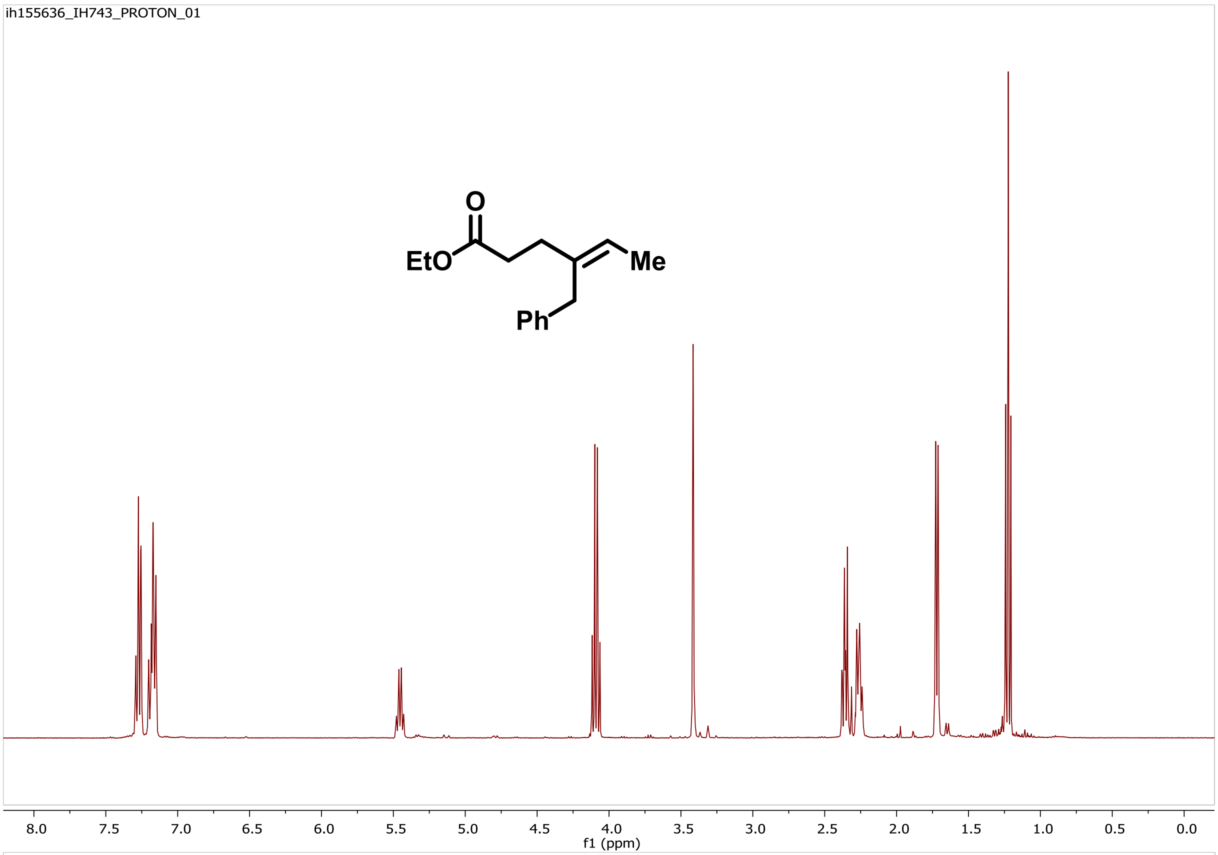
ih155636_IH743_PROTON_01
O
EtO
Me
Ph
8.0 7.5 7.0 6.5 6.0 5.5 5.0 4.5 4.0 3.5 3.0 2.5 2.0 1.5 1.0 0.5 0.0
f1 (ppm)

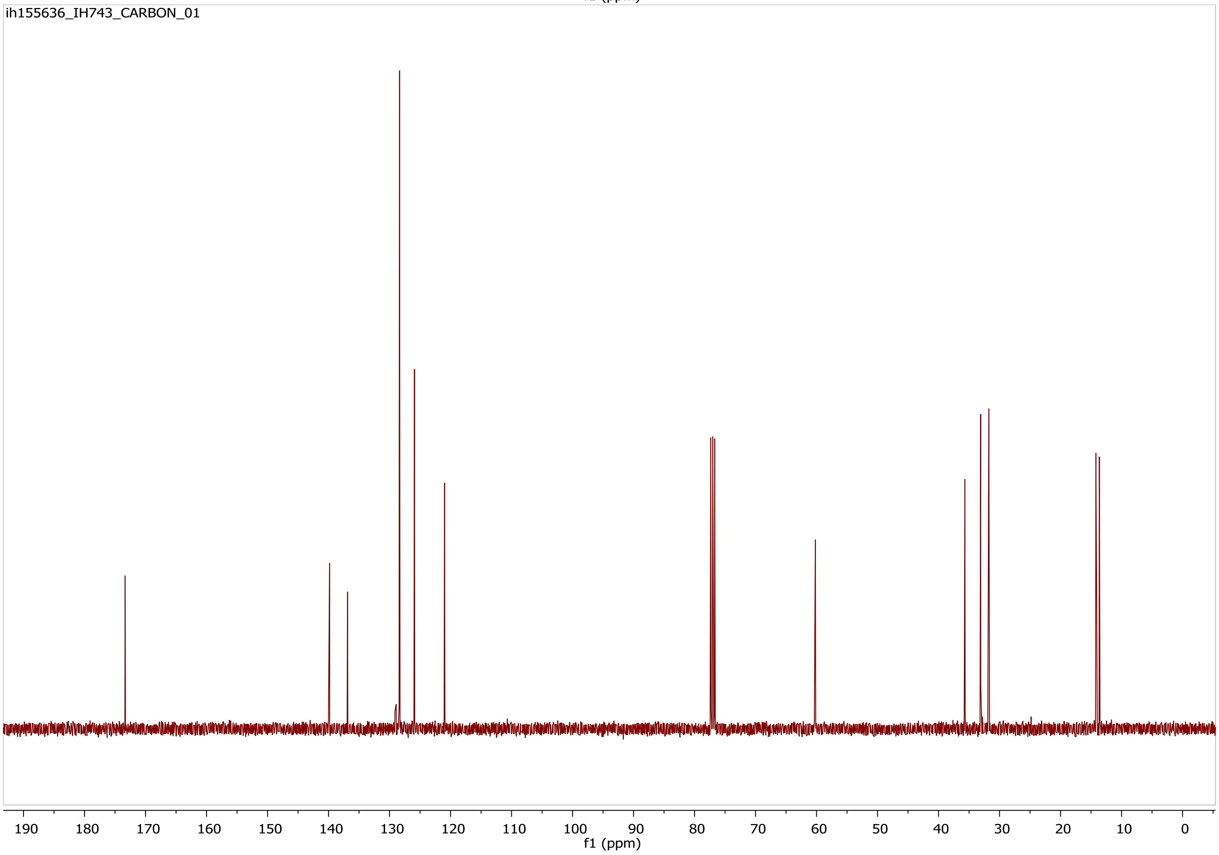
ih155636_IH743_CARBON_01
190 180 170 160 150 140 130 120 110 100 90 80 70 60 50 40 30 20 10 0
f1 (ppm)

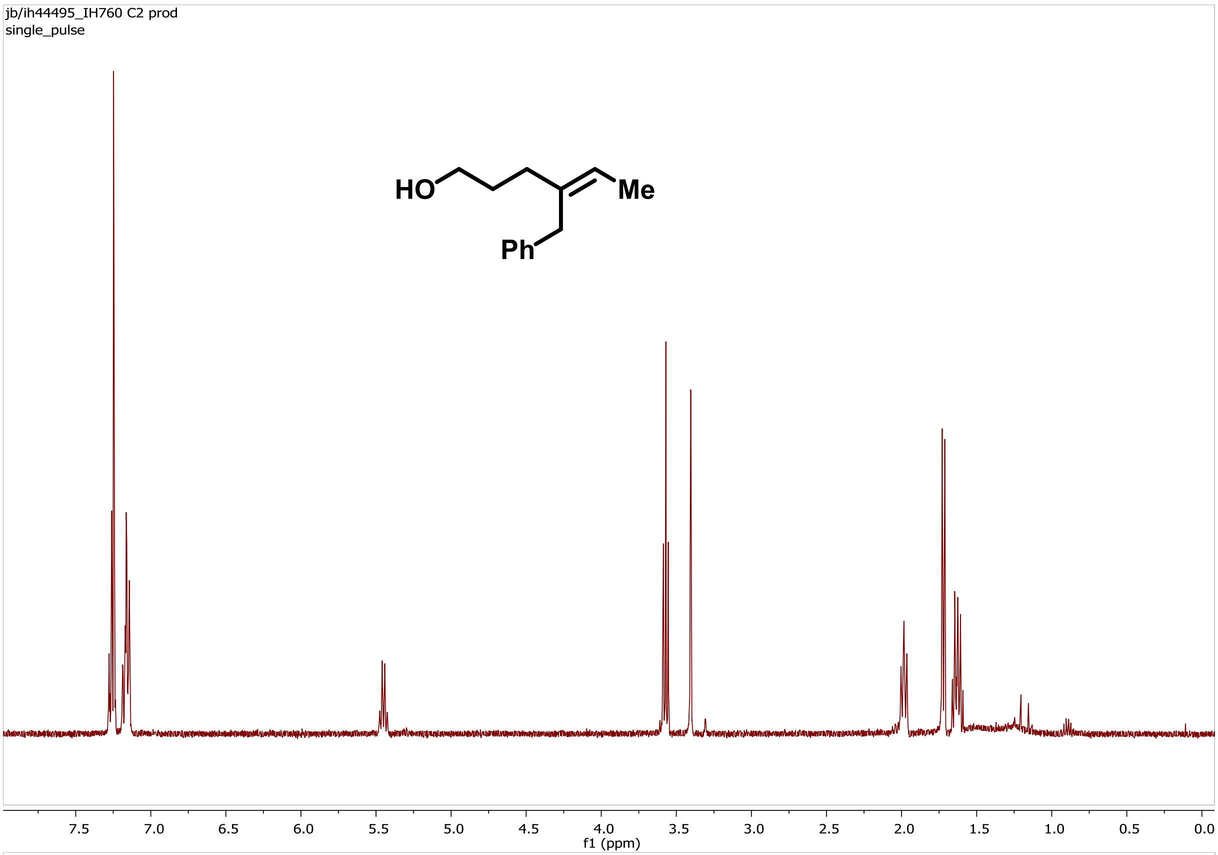
jb/ih44495_IH760 C2 prod
single_pulse
HO
Me
Ph
7.5
7.0
6.5
6.0
5.5
5.0
4.5
4.0
3.5
3.0
2.5
2.0
1.5
1.0
0.5
0.0
f1 (ppm)

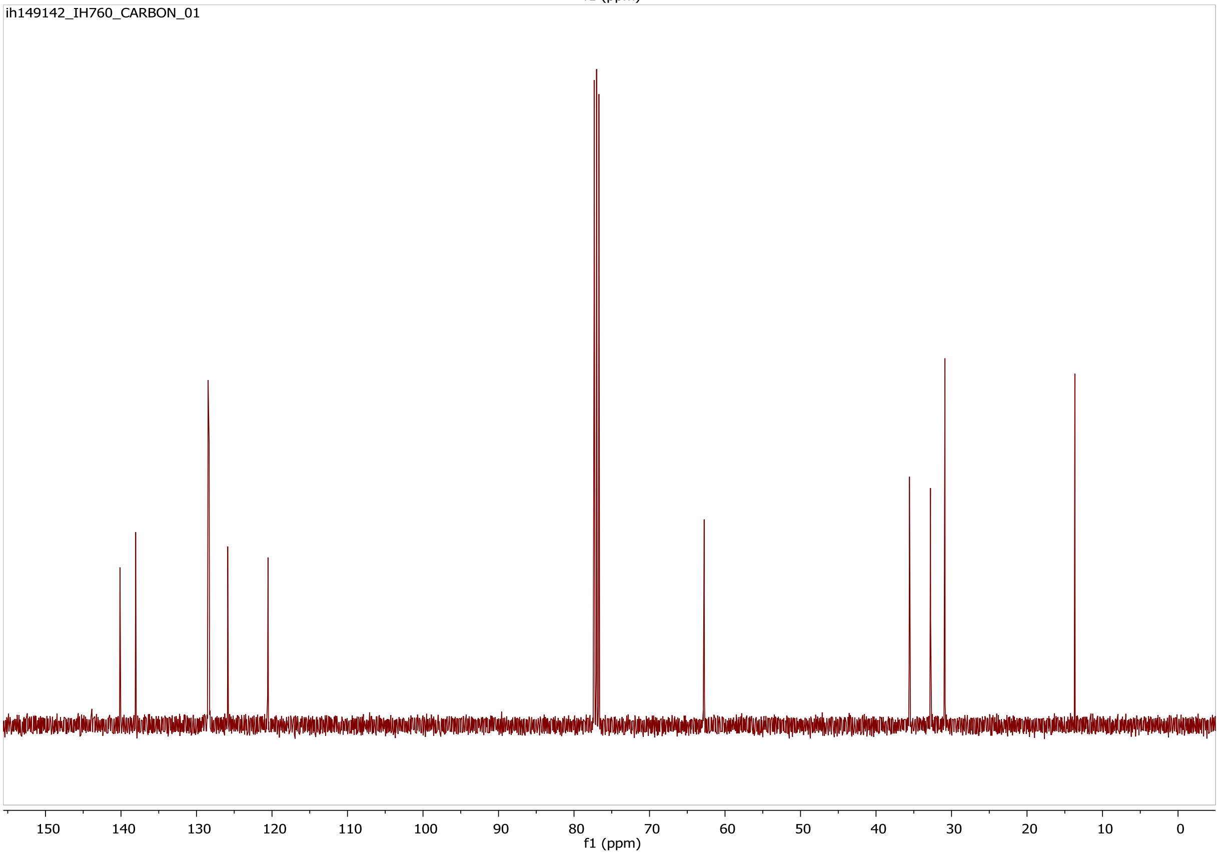
ih149142_IH760_CARBON_01
150
140
130
120
110
100
90
80
70
60
50
40
30
20
10
0
f1 (ppm)

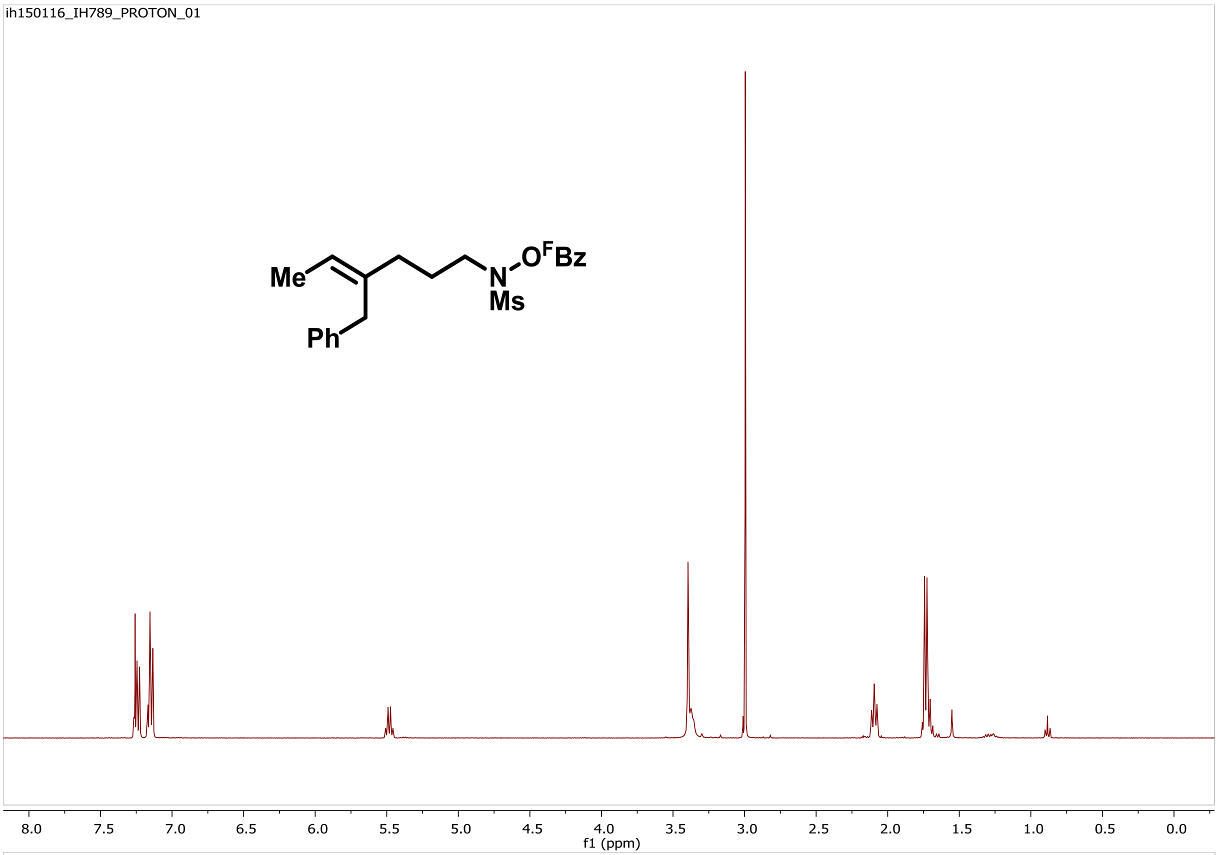
ih150116_IH789_PROTON_01
Me
Ph
N
O
F
Bz
Ms
f1 (ppm)

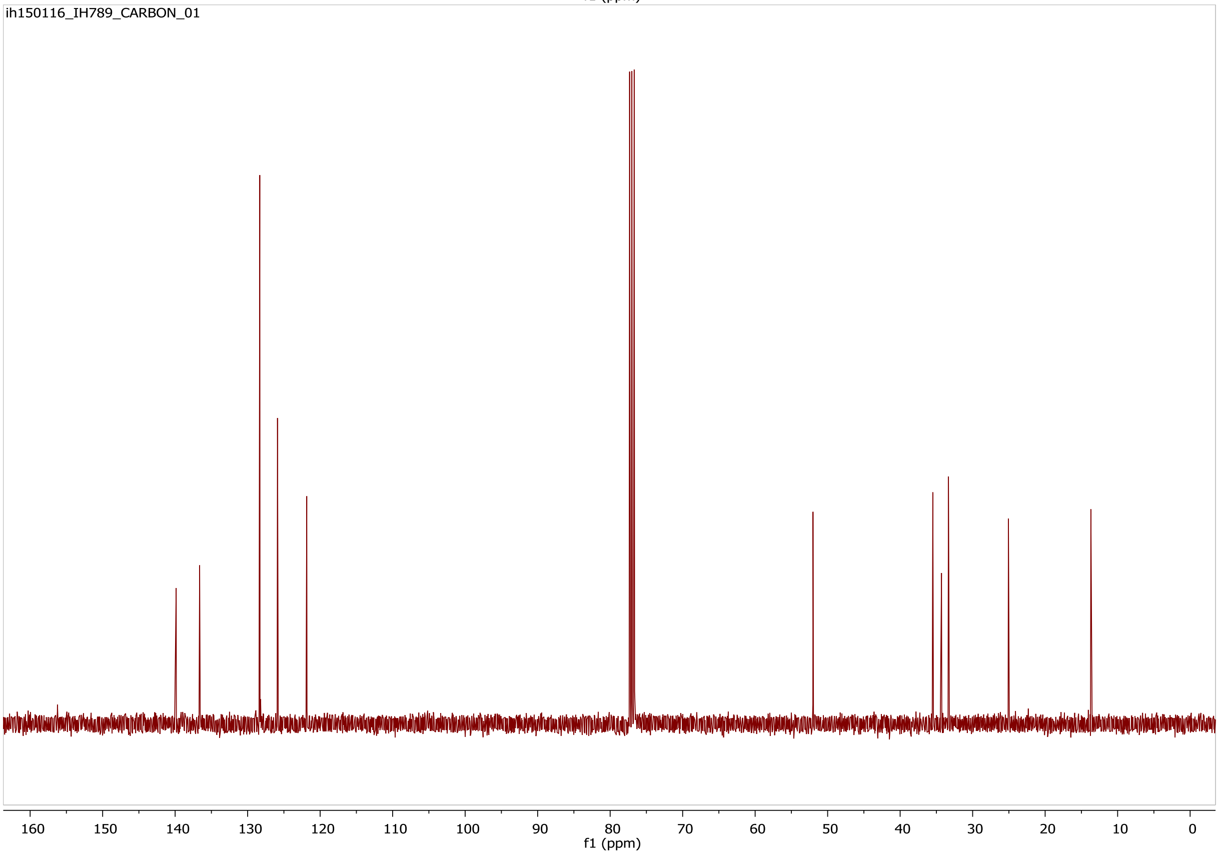
ih150116_IH789_CARBON_01
f1 (ppm)

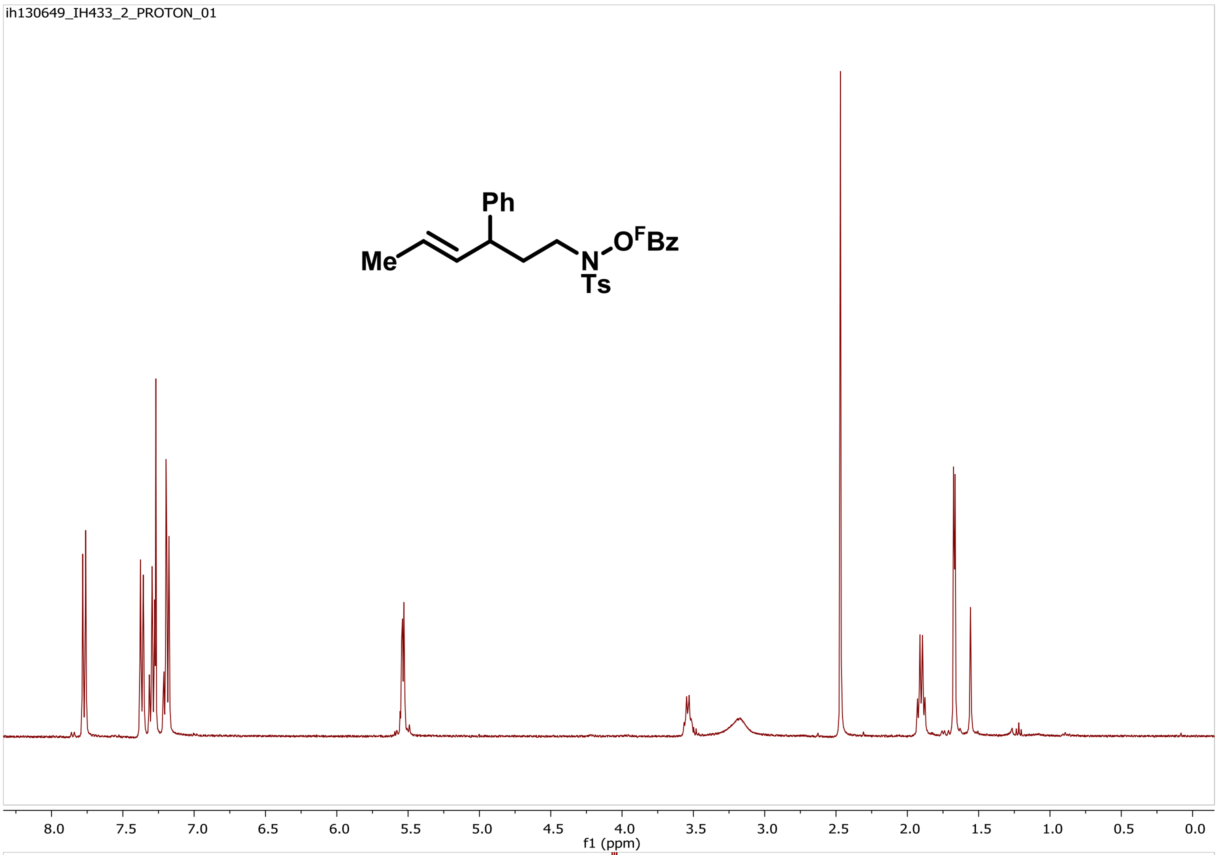
ih130649_IH433_2_PROTON_01
Ph
Me
N
O
F
Bz
Ts
f1 (ppm)

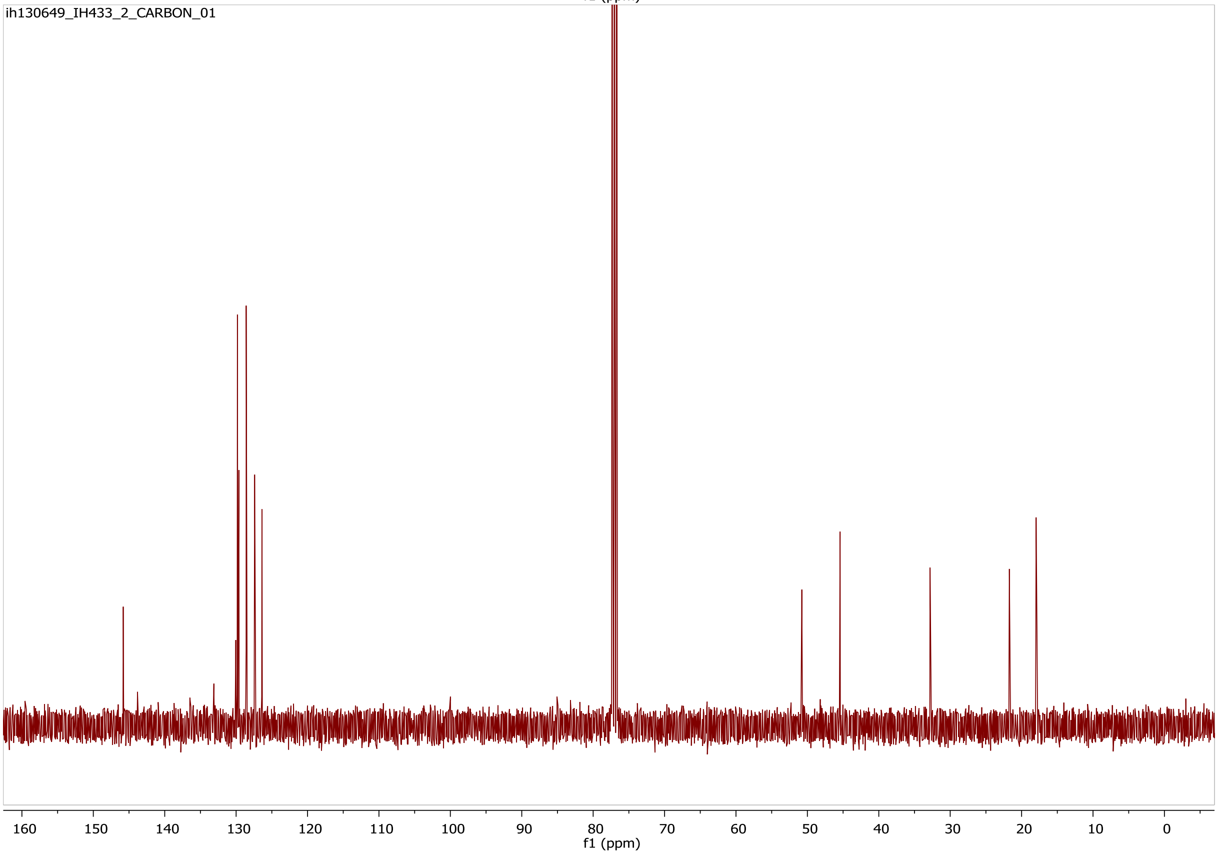
ih130649_IH433_2_CARBON_01
f1 (ppm)

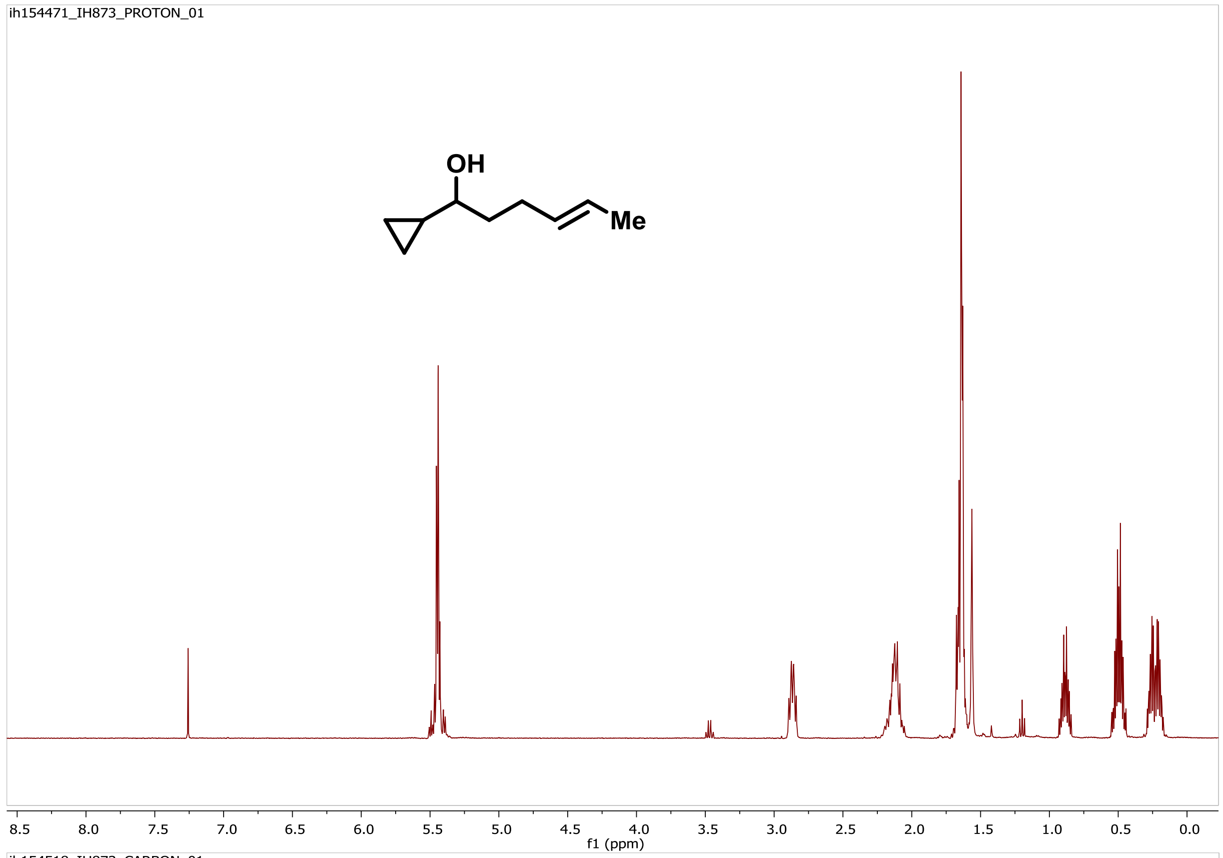
ih154471_IH873_PROTON_01
OH
Me
8.5 8.0 7.5 7.0 6.5 6.0 5.5 5.0 4.5 4.0 3.5 3.0 2.5 2.0 1.5 1.0 0.5 0.0
f1 (ppm)

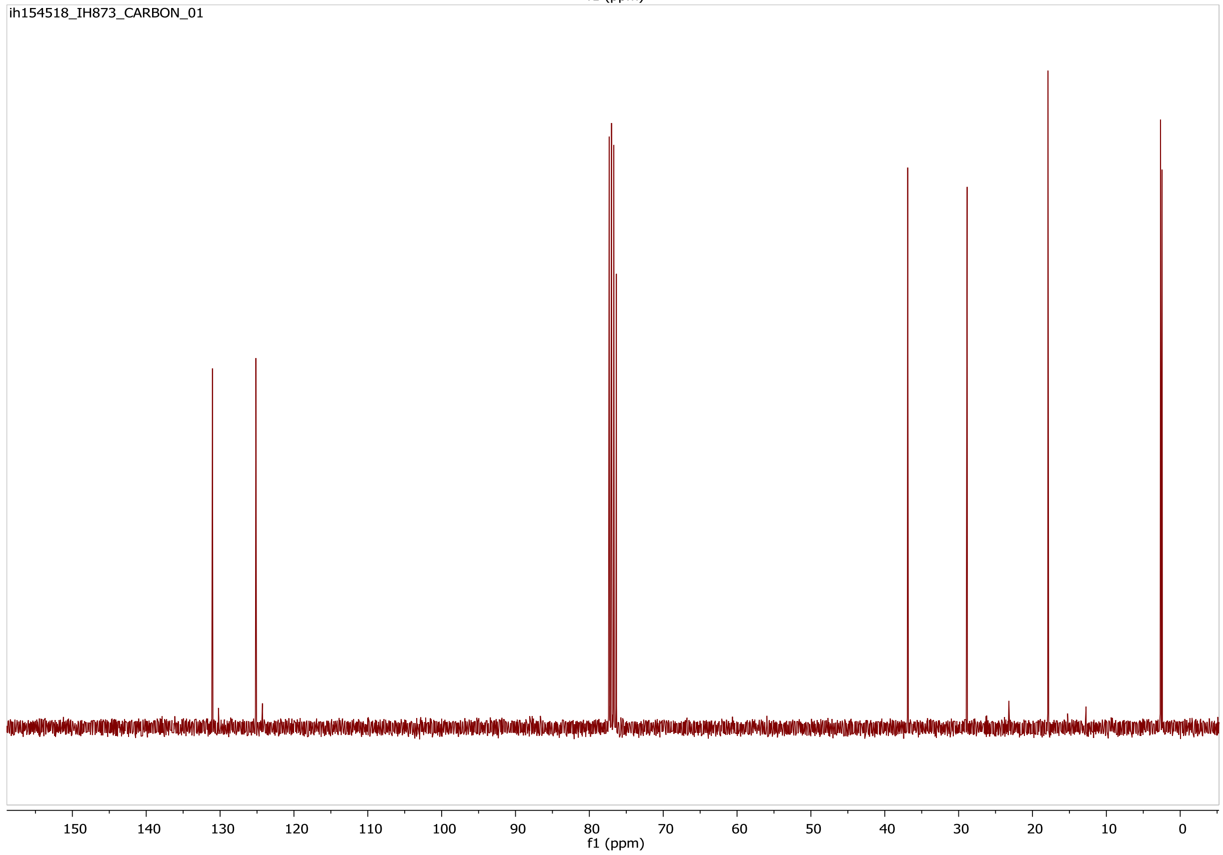
ih154518_IH873_CARBON_01
150 140 130 120 110 100 90 80 70 60 50 40 30 20 10 0
f1 (ppm)

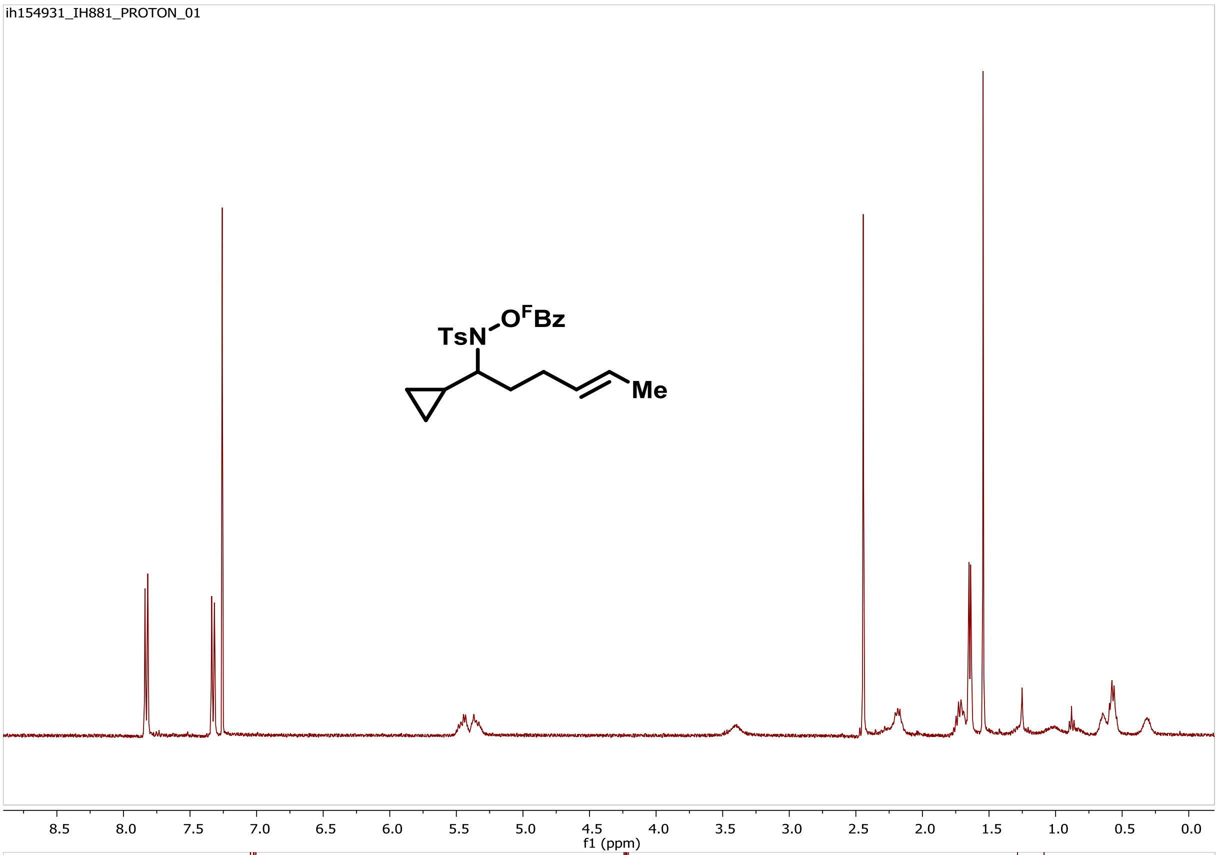
ih154931_IH881_PROTON_01
TsN
O^F Bz
Me
8.5 8.0 7.5 7.0 6.5 6.0 5.5 5.0 4.5 4.0 3.5 3.0 2.5 2.0 1.5 1.0 0.5 0.0
f1 (ppm)

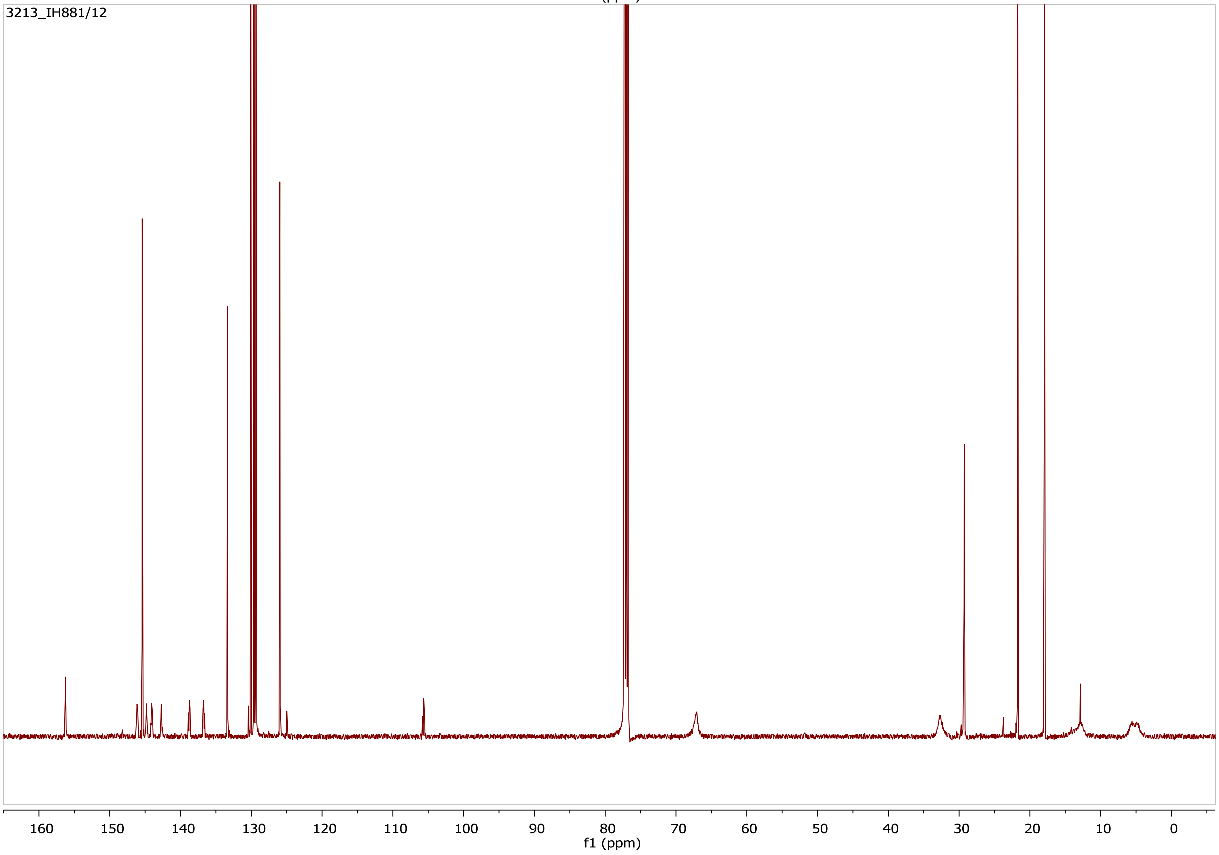
3213_IH881/12
160 150 140 130 120 110 100 90 80 70 60 50 40 30 20 10 0
f1 (ppm)

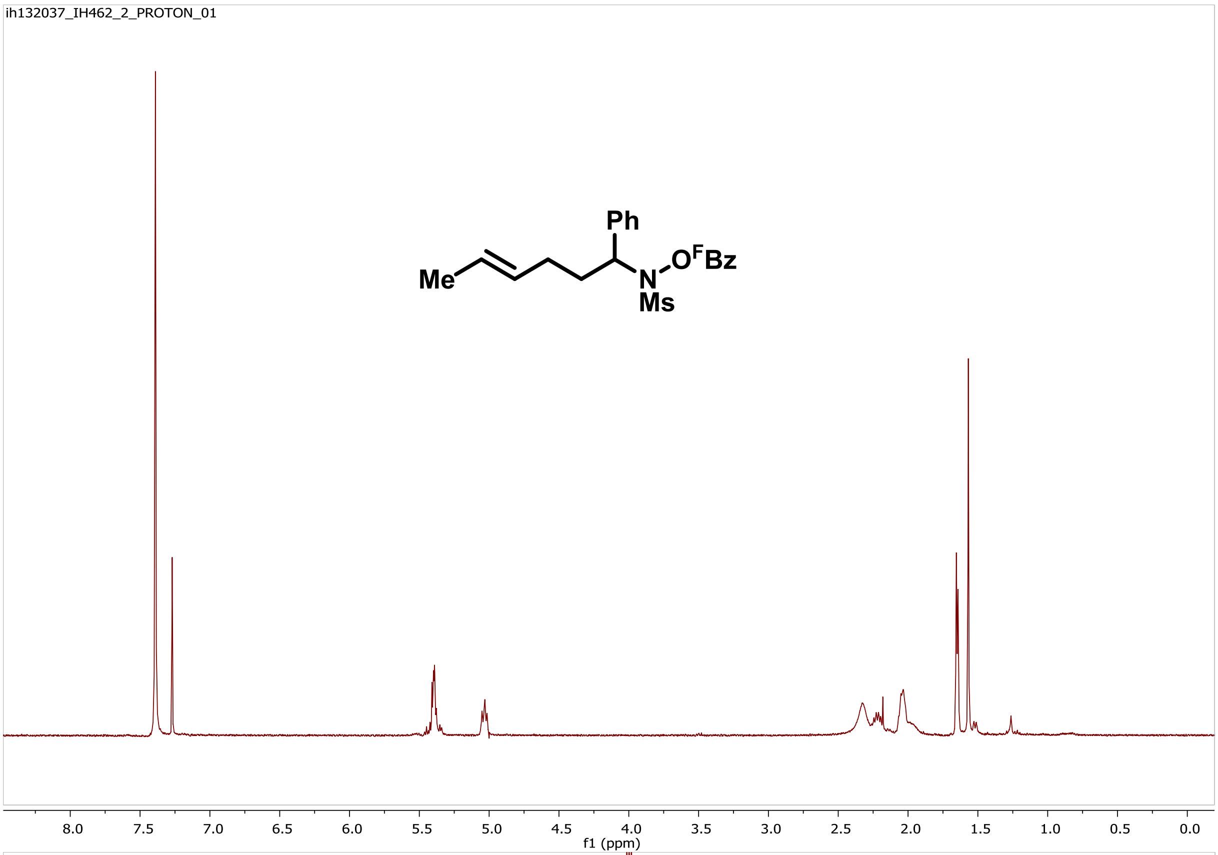
ih132037_IH462_2_PROTON_01
Ph
Me
N
O
F
Bz
Ms
8.0
7.5
7.0
6.5
6.0
5.5
5.0
4.5
4.0
3.5
3.0
2.5
2.0
1.5
1.0
0.5
0.0
f1 (ppm)

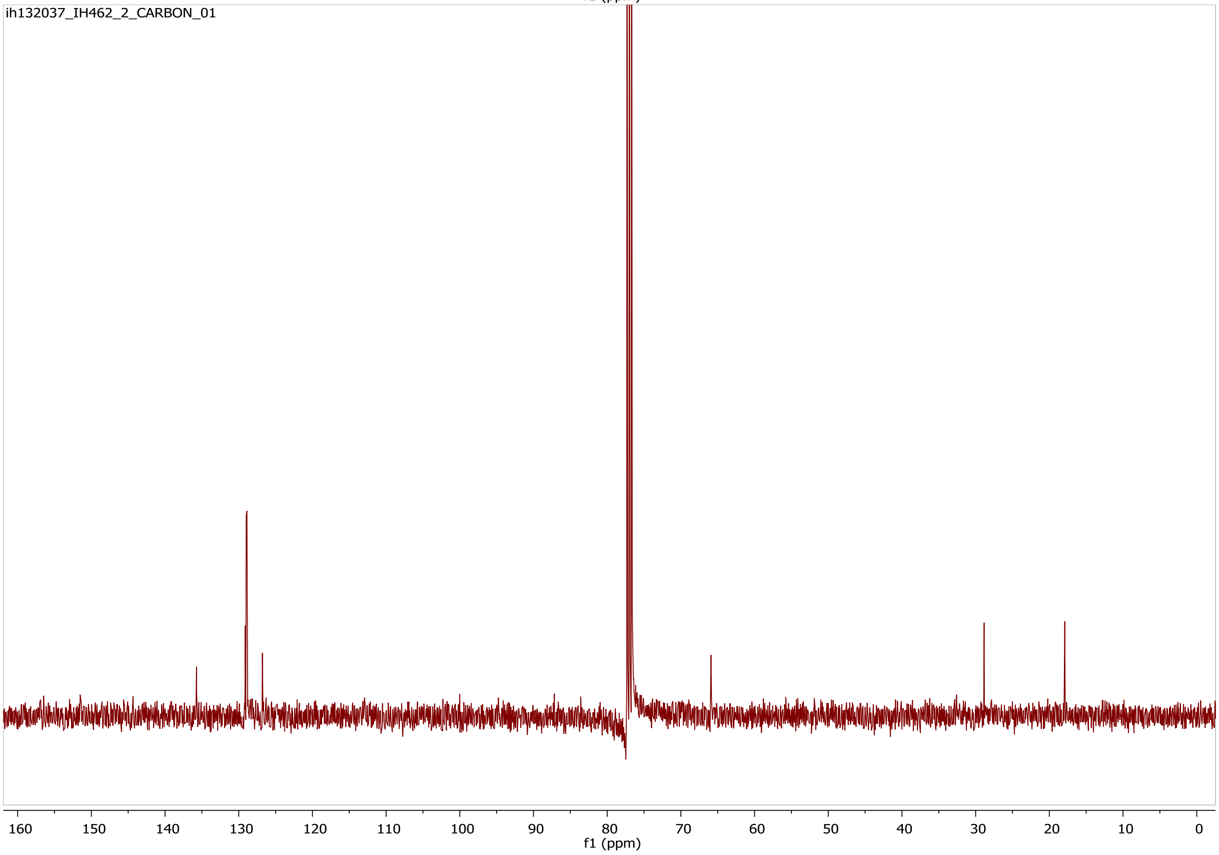
ih132037_IH462_2_CARBON_01
160
150
140
130
120
110
100
90
80
70
60
50
40
30
20
10
0
f1 (ppm)

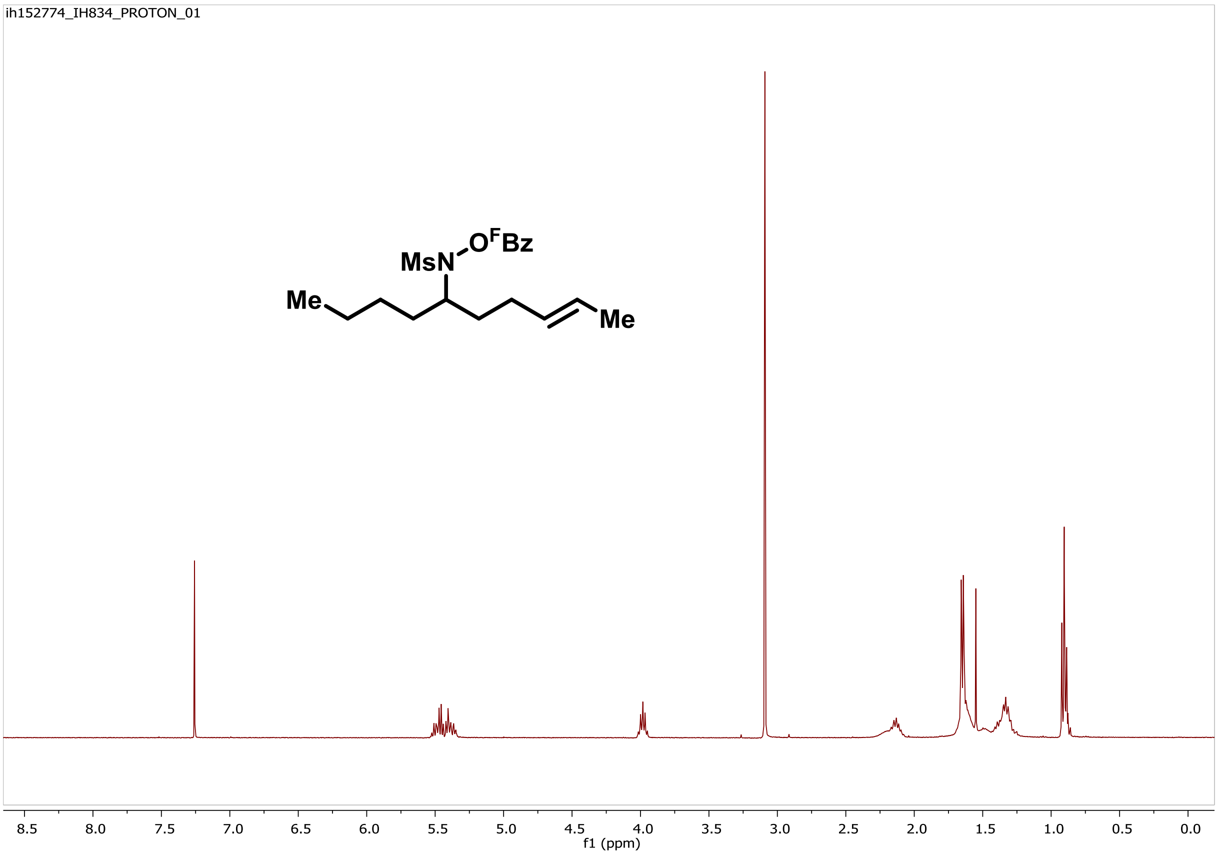
ih152774_IH834_PROTON_01
MsN
OFBz
Me
Me
8.5 8.0 7.5 7.0 6.5 6.0 5.5 5.0 4.5 4.0 3.5 3.0 2.5 2.0 1.5 1.0 0.5 0.0
f1 (ppm)

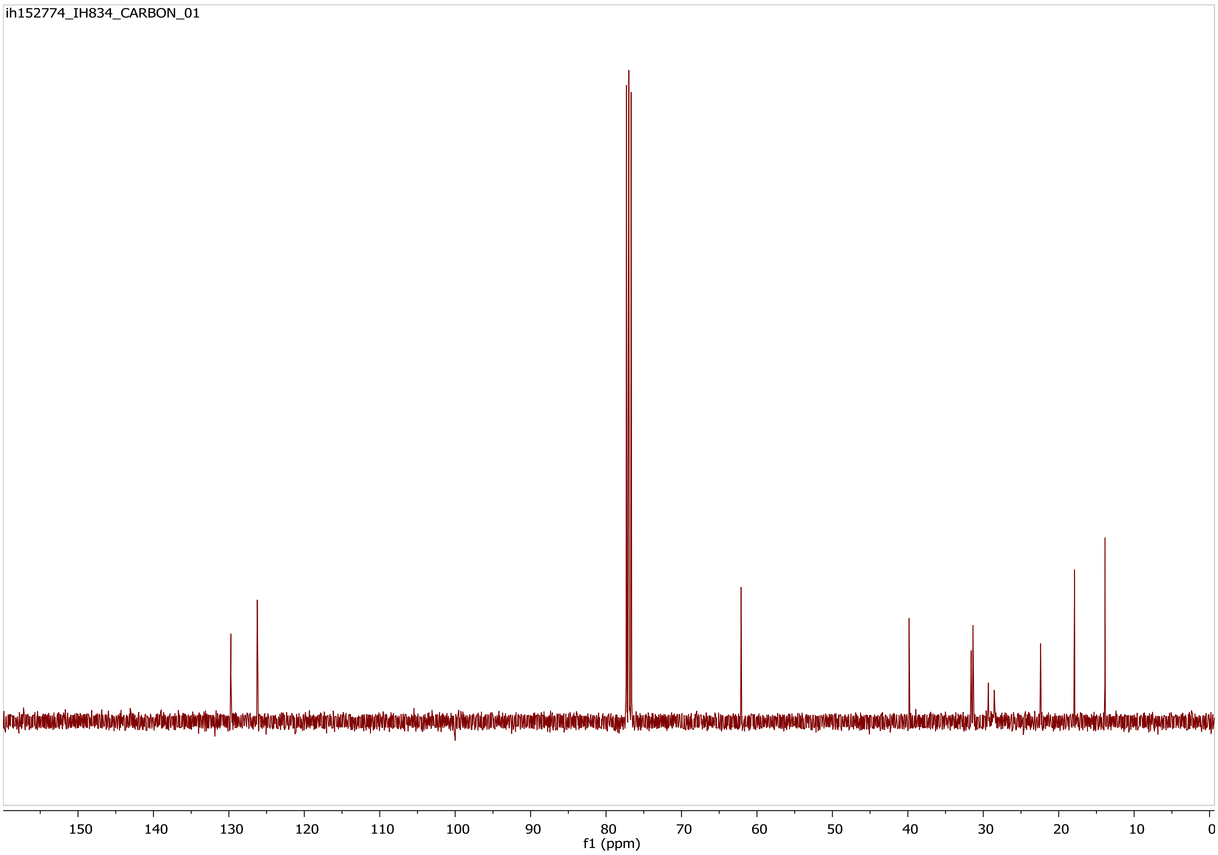
ih152774_IH834_CARBON_01
150 140 130 120 110 100 90 80 70 60 50 40 30 20 10 0
f1 (ppm)

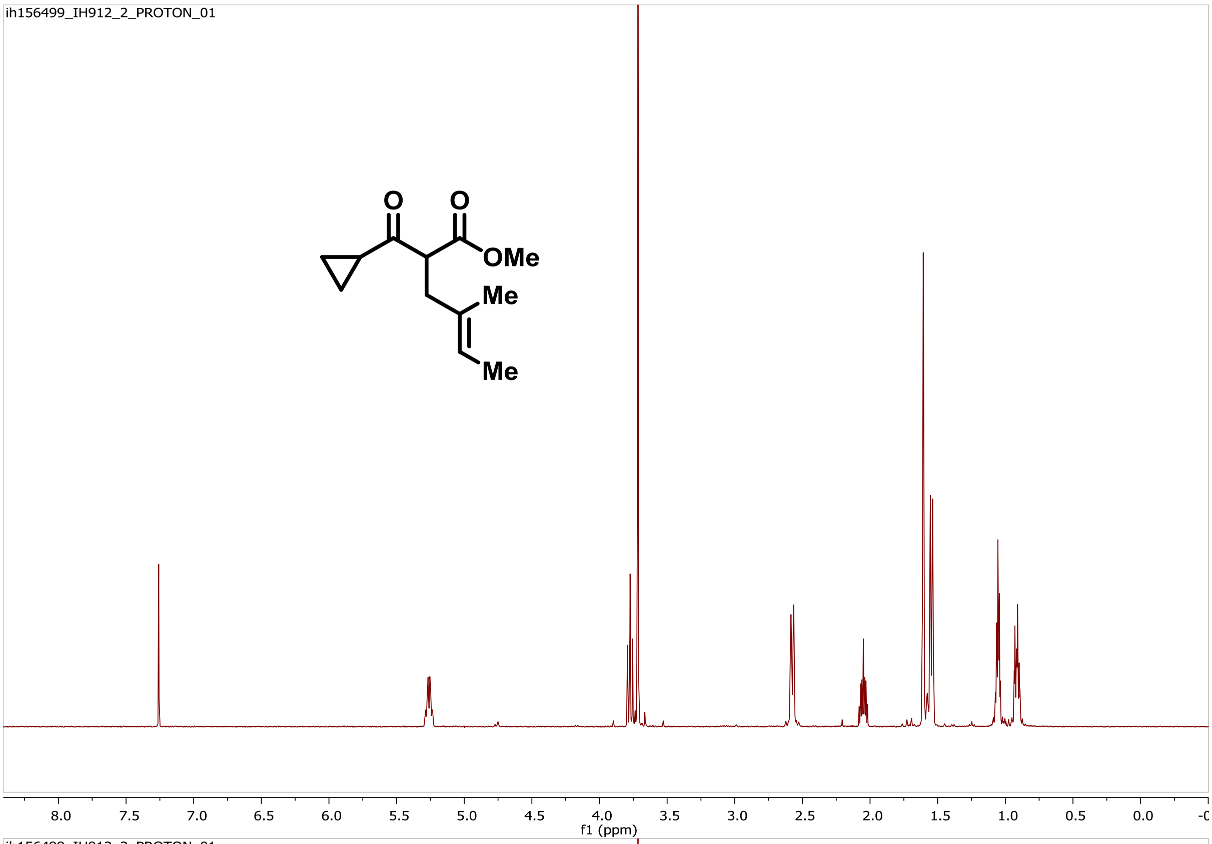
ih156499_IH912_2_PROTON_01
O
O
OMe
Me
Me
8.0 7.5 7.0 6.5 6.0 5.5 5.0 4.5 4.0 3.5 3.0 2.5 2.0 1.5 1.0 0.5 0.0
f1 (ppm)

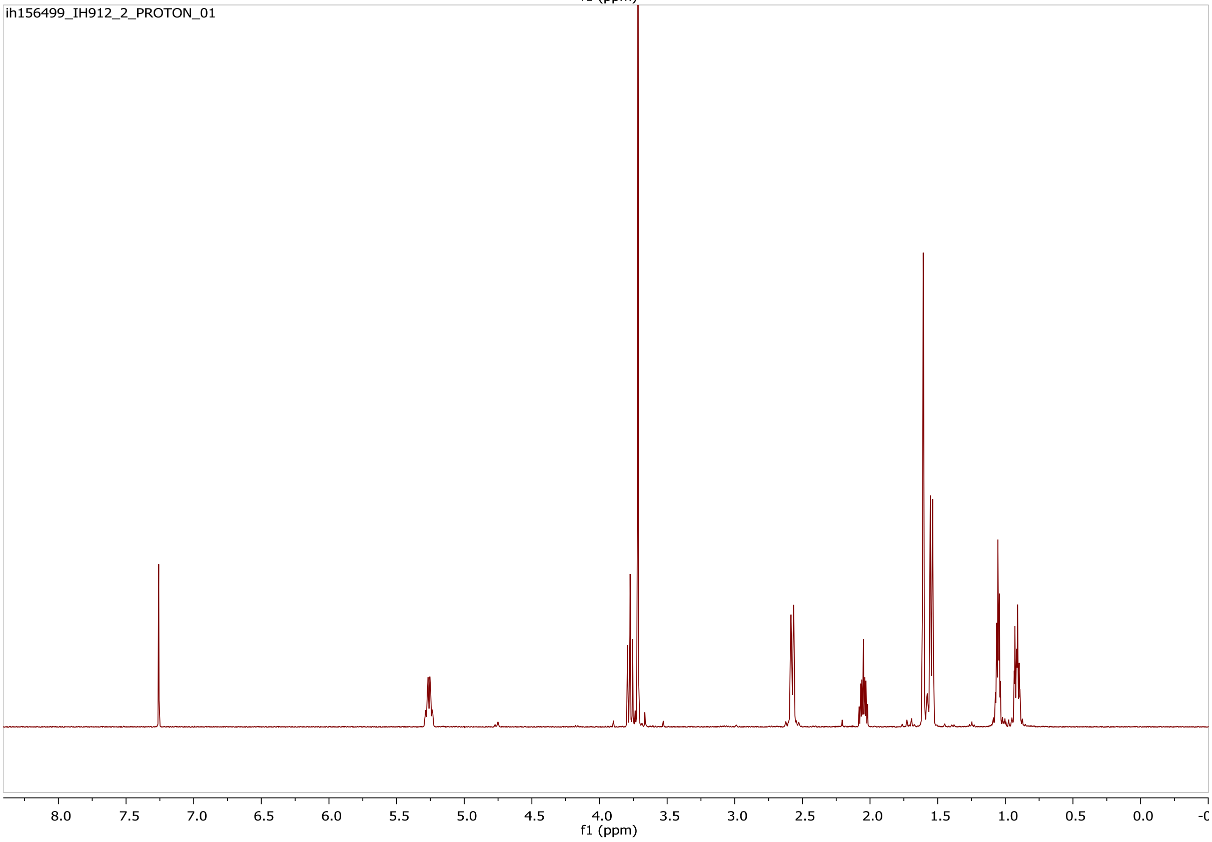
ih156499_IH912_2_PROTON_01
8.0 7.5 7.0 6.5 6.0 5.5 5.0 4.5 4.0 3.5 3.0 2.5 2.0 1.5 1.0 0.5 0.0
f1 (ppm)

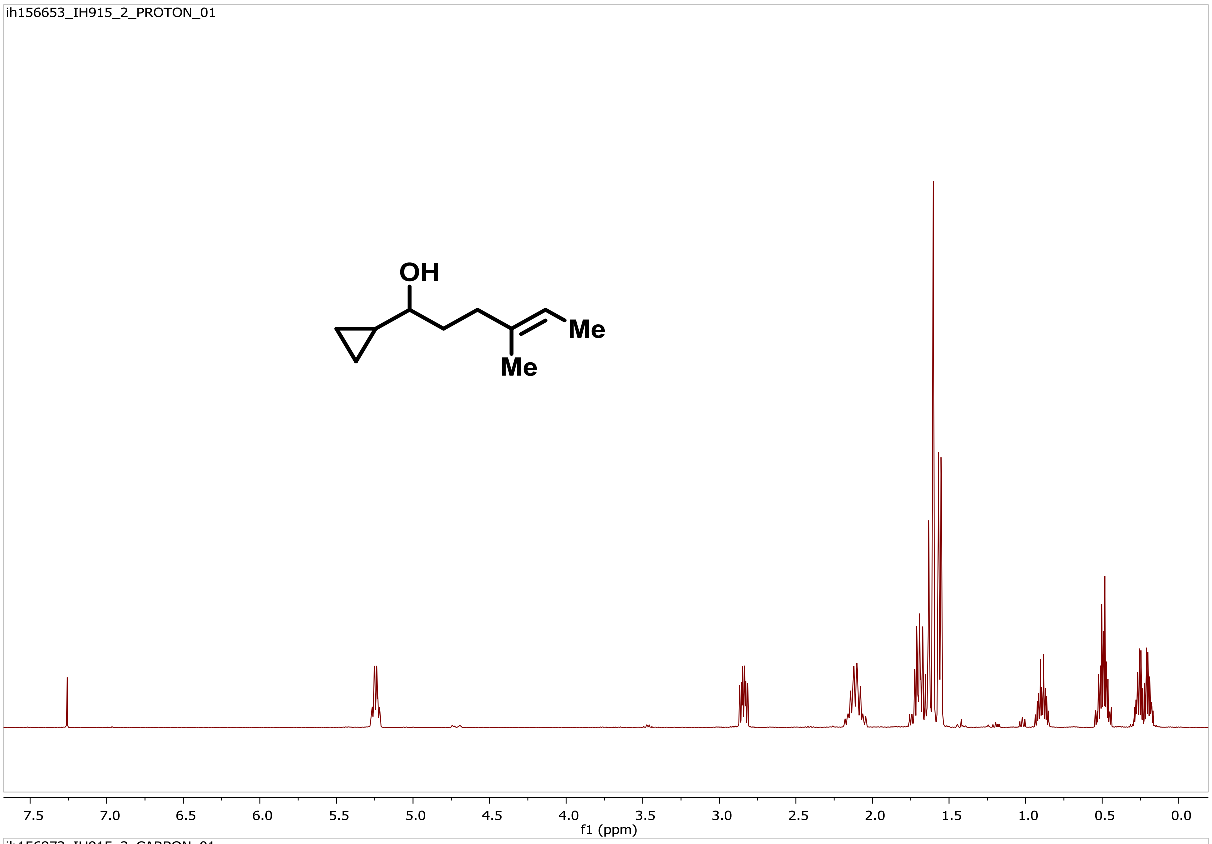
ih156653_IH915_2_PROTON_01
OH
Me
Me
7.5 7.0 6.5 6.0 5.5 5.0 4.5 4.0 3.5 3.0 2.5 2.0 1.5 1.0 0.5 0.0
f1 (ppm)

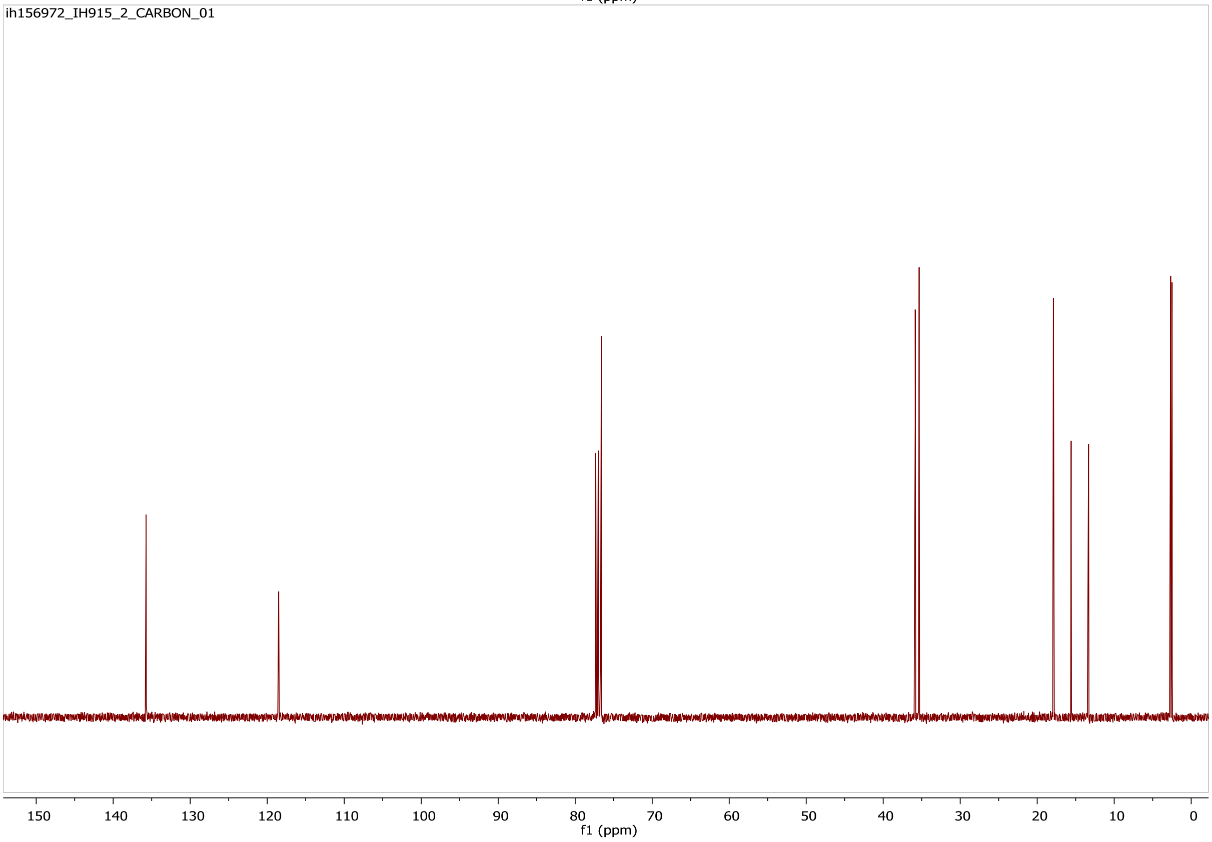
ih156972_IH915_2_CARBON_01
150 140 130 120 110 100 90 80 70 60 50 40 30 20 10 0
f1 (ppm)

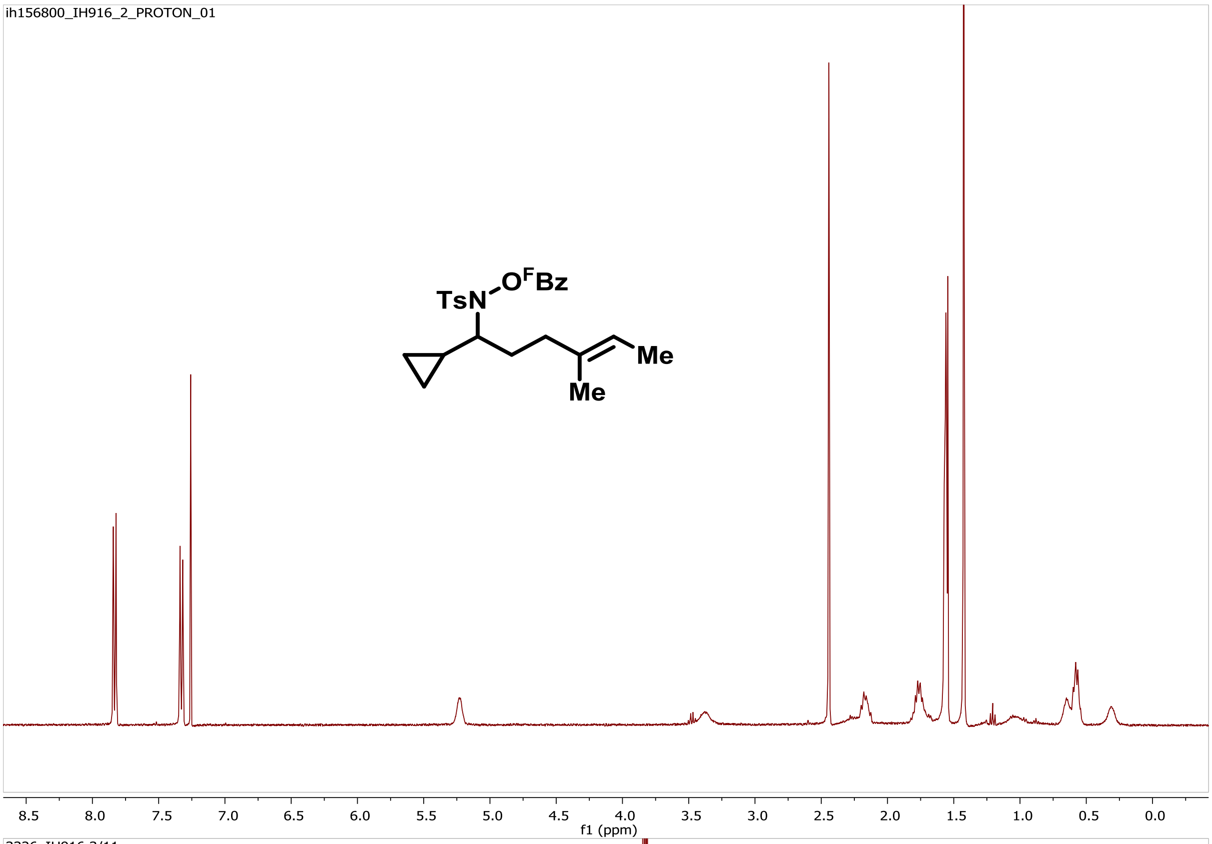
ih156800_IH916_2_PROTON_01
TsN
OFBz
Me
Me
8.5 8.0 7.5 7.0 6.5 6.0 5.5 5.0 4.5 4.0 3.5 3.0 2.5 2.0 1.5 1.0 0.5 0.0
f1 (ppm)

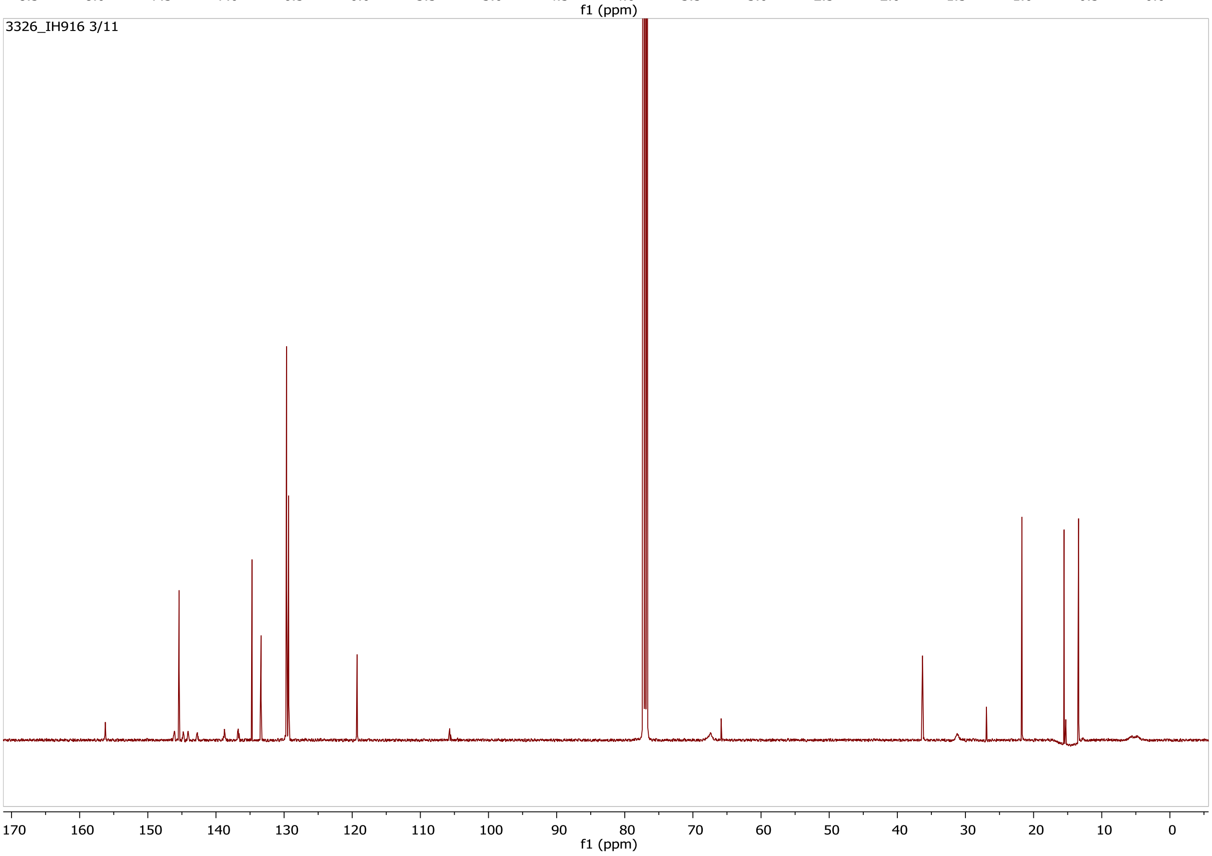
3326_IH916 3/11
170 160 150 140 130 120 110 100 90 80 70 60 50 40 30 20 10 0
f1 (ppm)

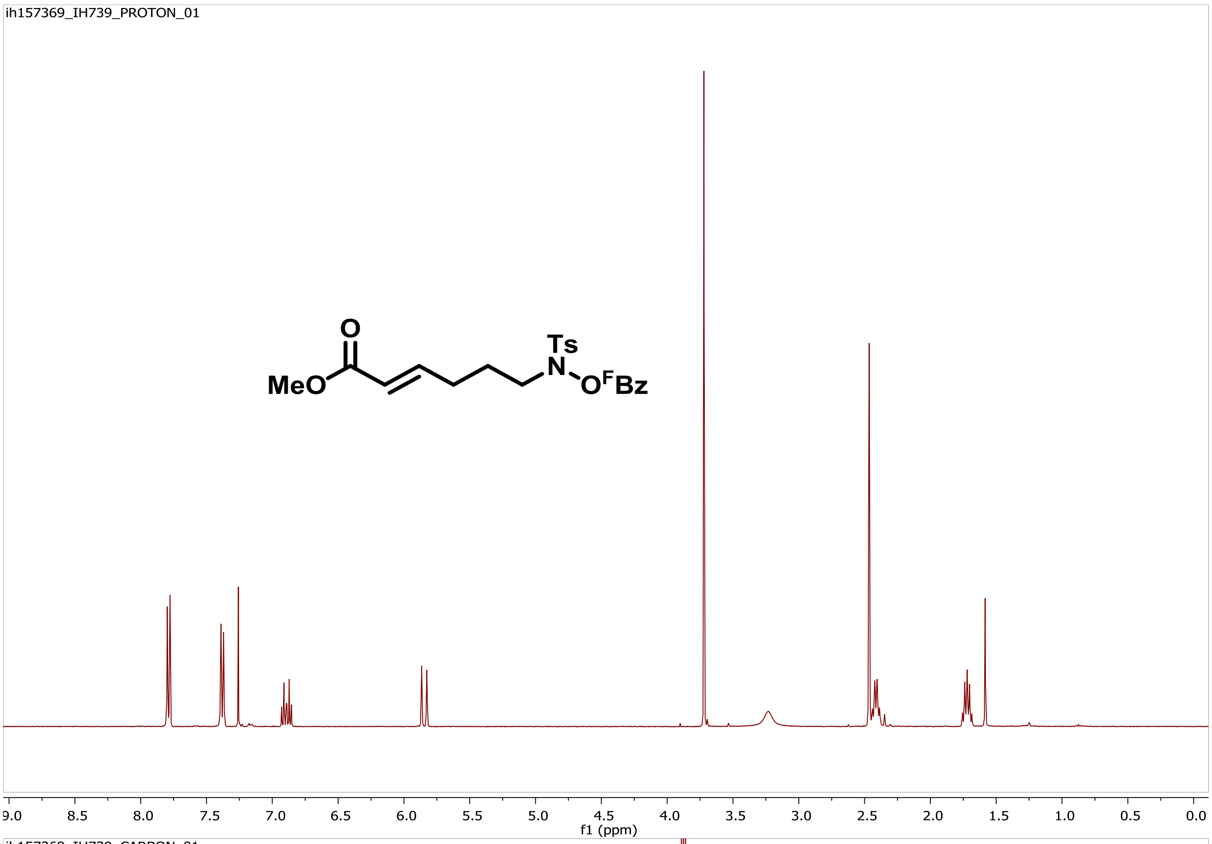
ih157369_IH739_PROTON_01
MeO
O
Ts
N
O^{F}Bz
9.0 8.5 8.0 7.5 7.0 6.5 6.0 5.5 5.0 4.5 4.0 3.5 3.0 2.5 2.0 1.5 1.0 0.5 0.0
f1 (ppm)

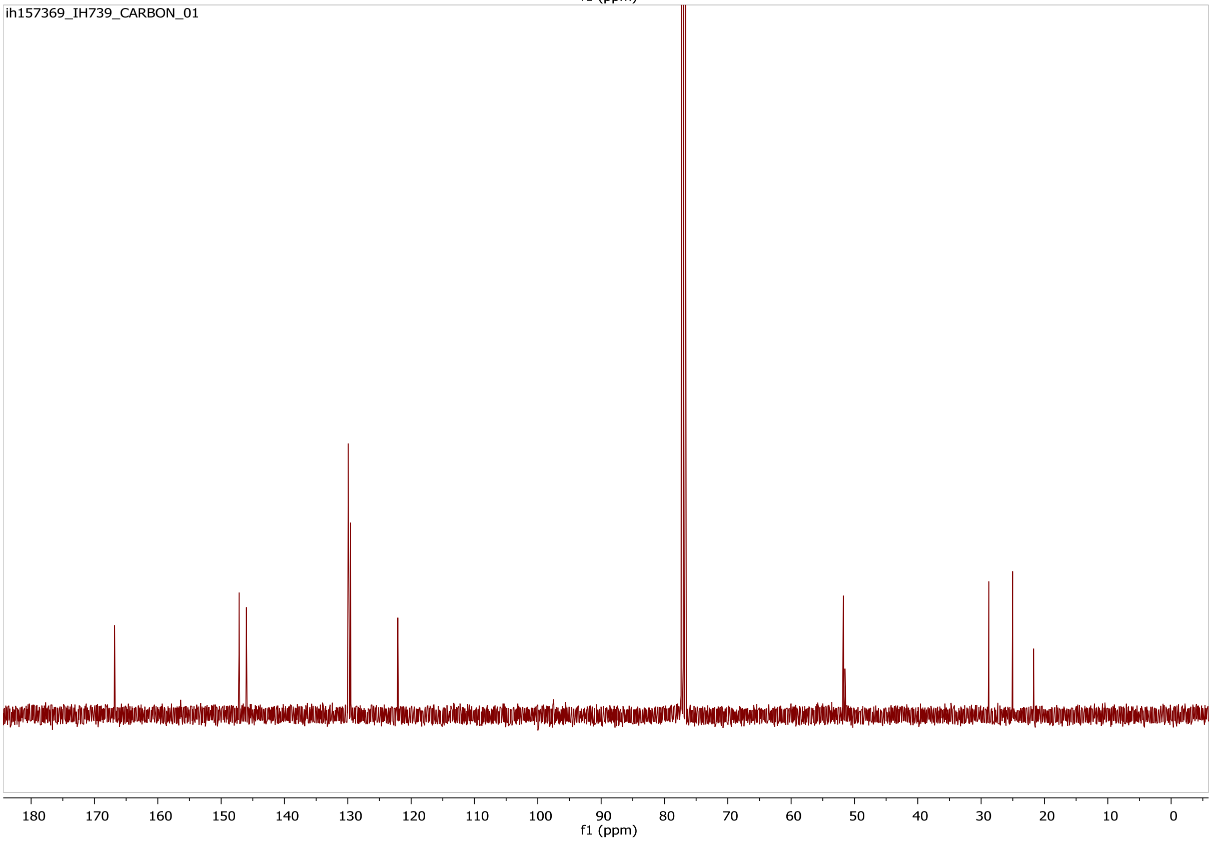
ih157369_IH739_CARBON_01
180 170 160 150 140 130 120 110 100 90 80 70 60 50 40 30 20 10 0
f1 (ppm)

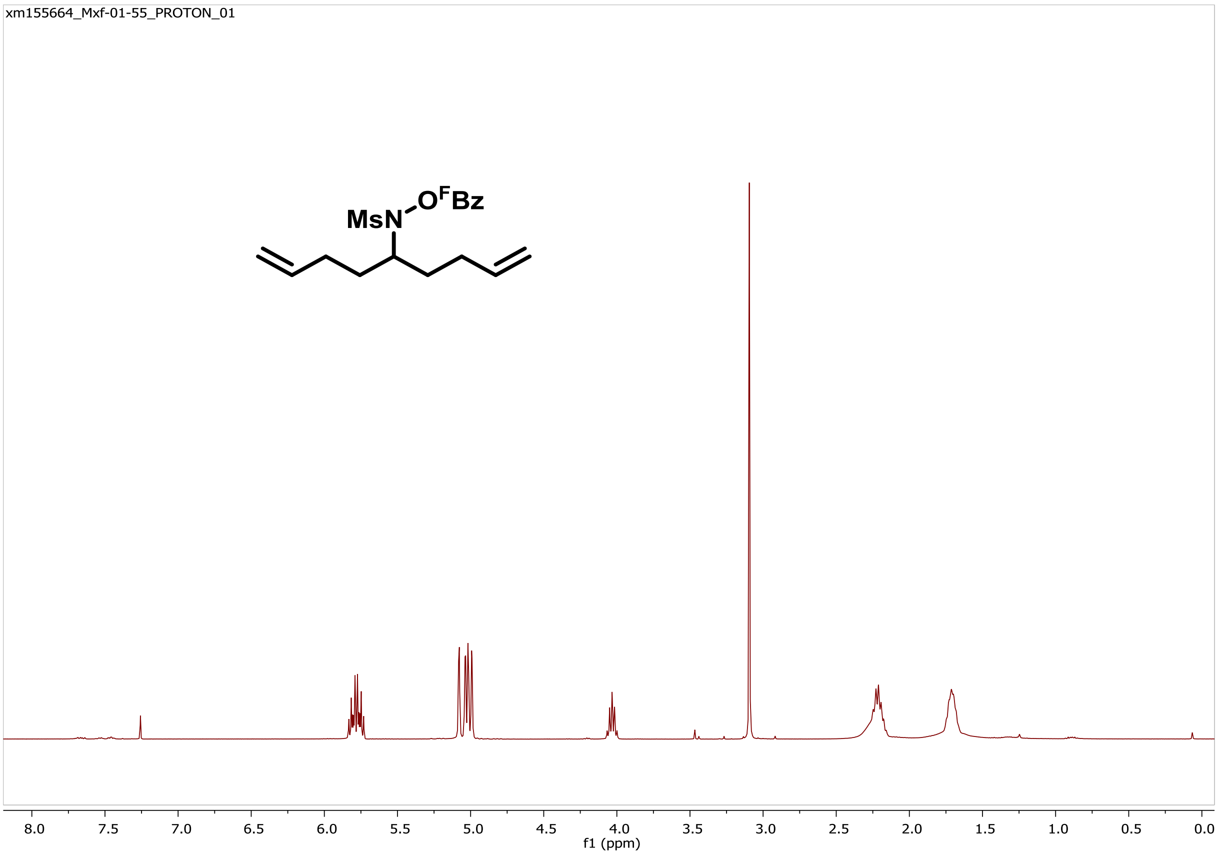
xm155664_Mxf-01-55_PROTON_01
MsN
O
F
Bz
8.0
7.5
7.0
6.5
6.0
5.5
5.0
4.5
4.0
3.5
3.0
2.5
2.0
1.5
1.0
0.5
0.0
f1 (ppm)

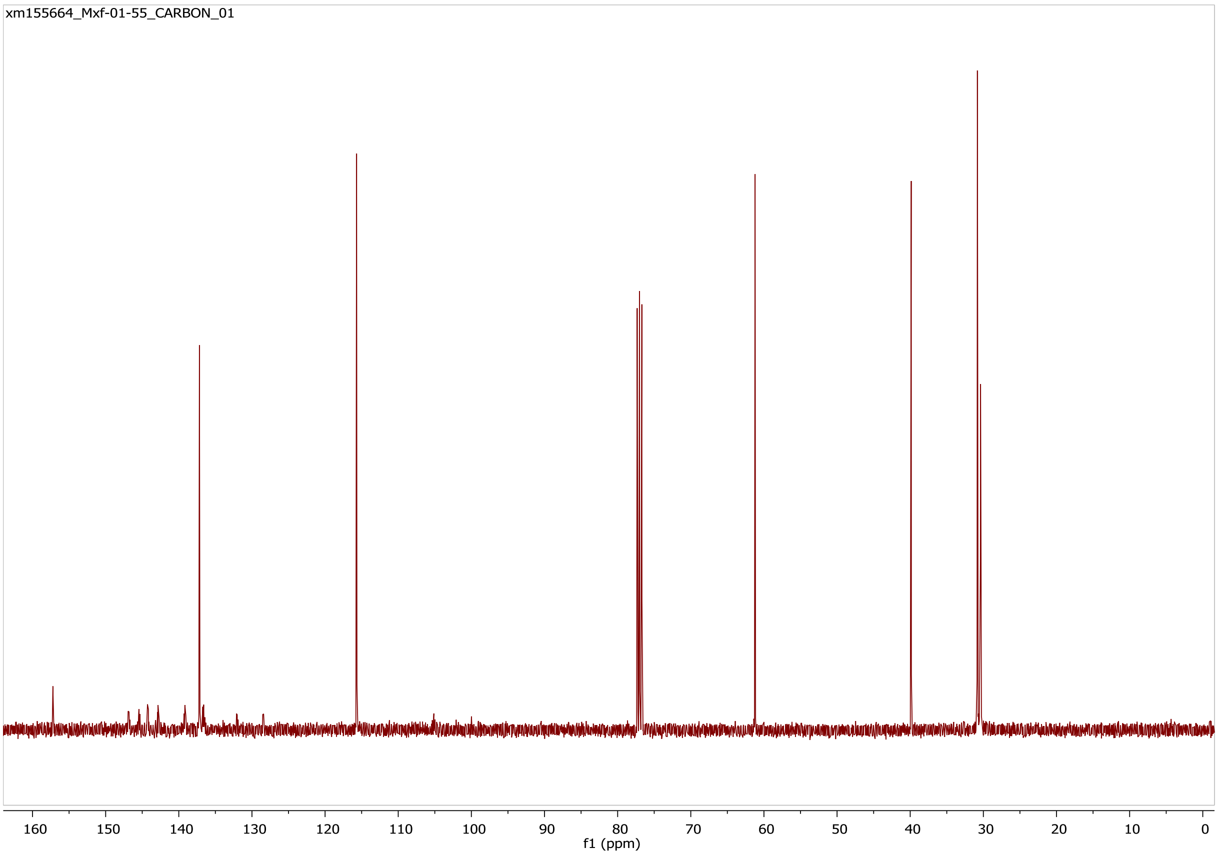
xm155664_Mxf-01-55_CARBON_01
160
150
140
130
120
110
100
90
80
70
60
50
40
30
20
10
0
f1 (ppm)

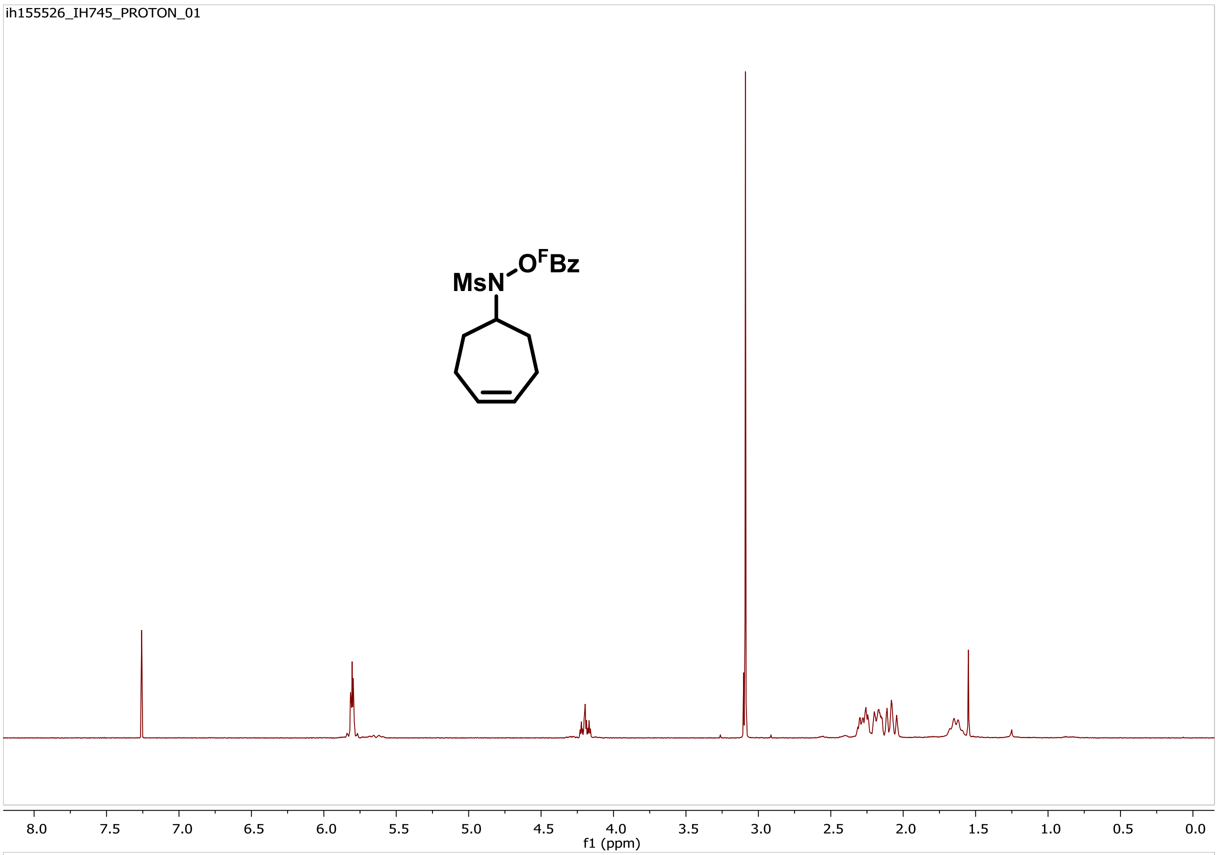
ih155526_IH745_PROTON_01
MsN
OFBz
8.0
7.5
7.0
6.5
6.0
5.5
5.0
4.5
4.0
3.5
3.0
2.5
2.0
1.5
1.0
0.5
0.0
f1 (ppm)

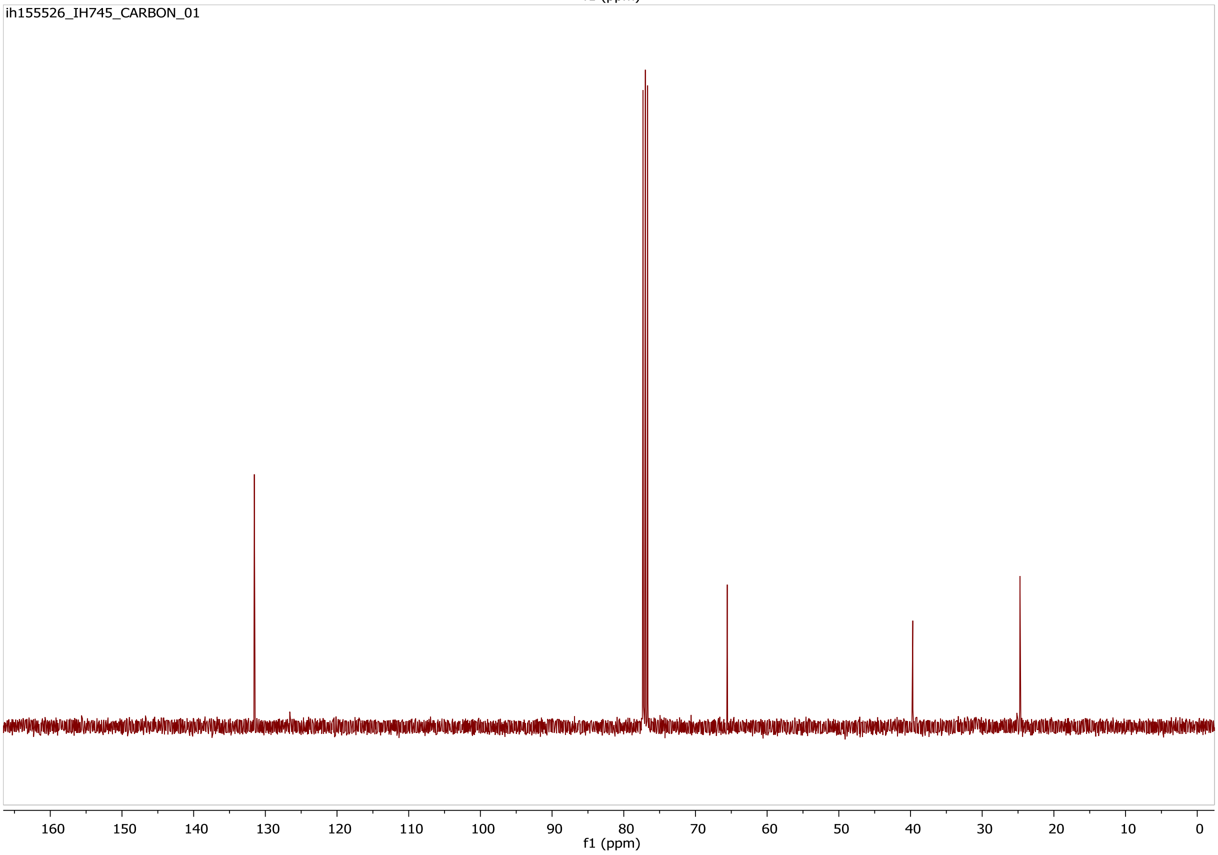
ih155526_IH745_CARBON_01
160
150
140
130
120
110
100
90
80
70
60
50
40
30
20
10
0
f1 (ppm)

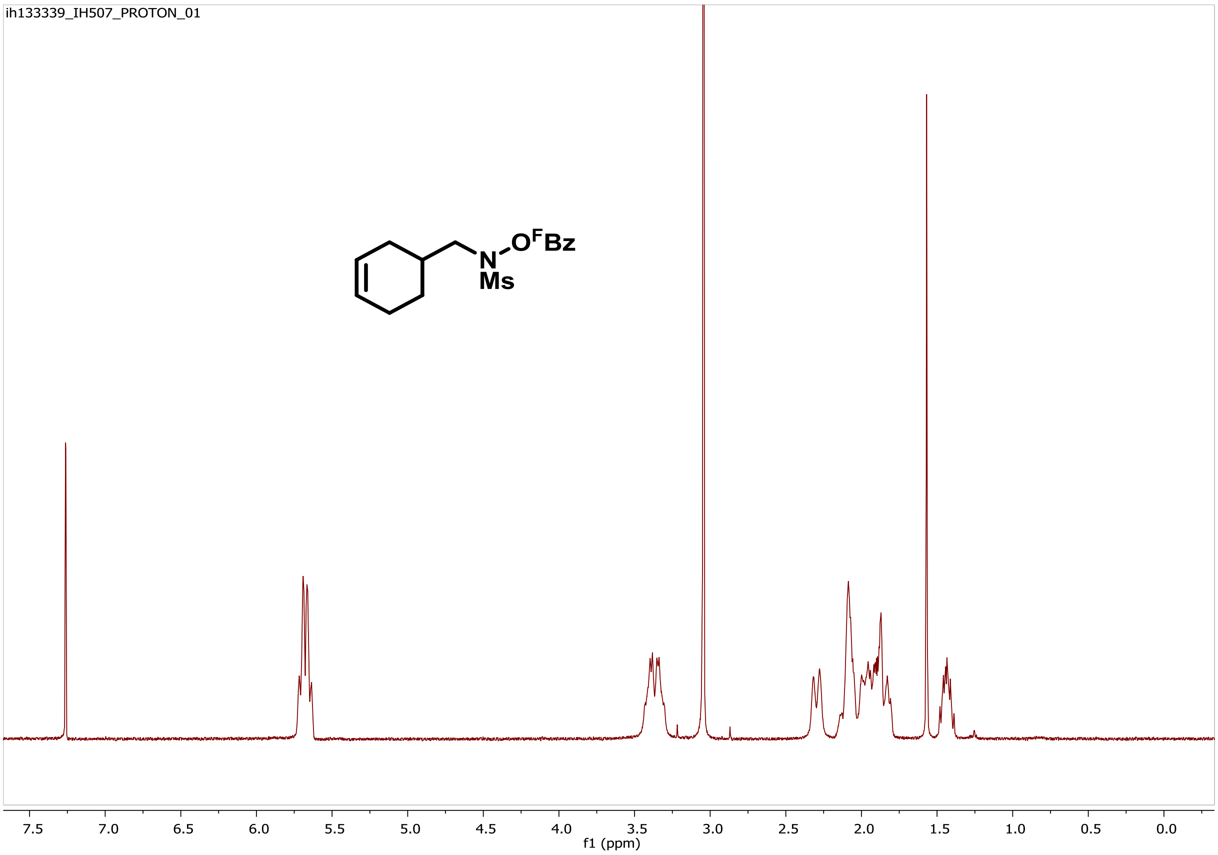
ih133339_IH507_PROTON_01
O
F
Bz
N
Ms
7.5
7.0
6.5
6.0
5.5
5.0
4.5
4.0
3.5
3.0
2.5
2.0
1.5
1.0
0.5
0.0
f1 (ppm)

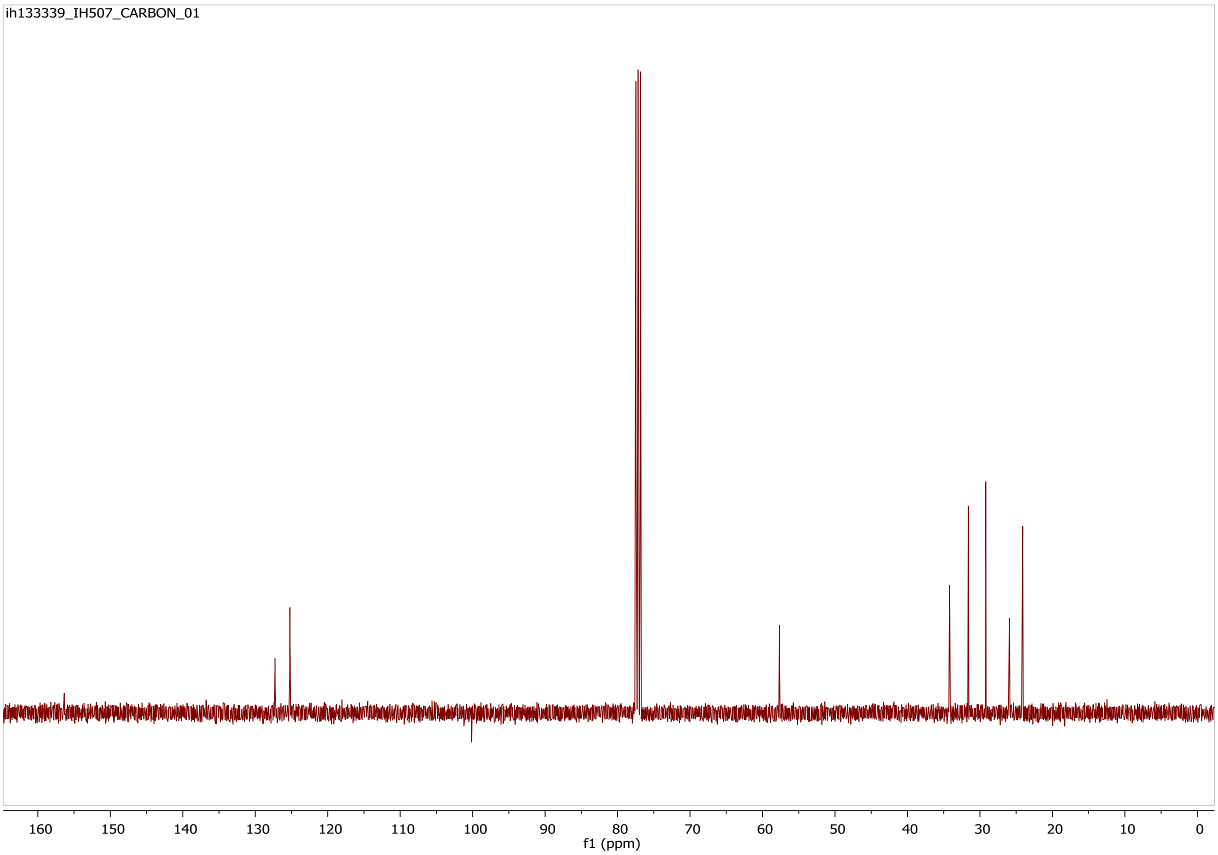
ih133339_IH507_CARBON_01
160
150
140
130
120
110
100
90
80
70
60
50
40
30
20
10
0
f1 (ppm)

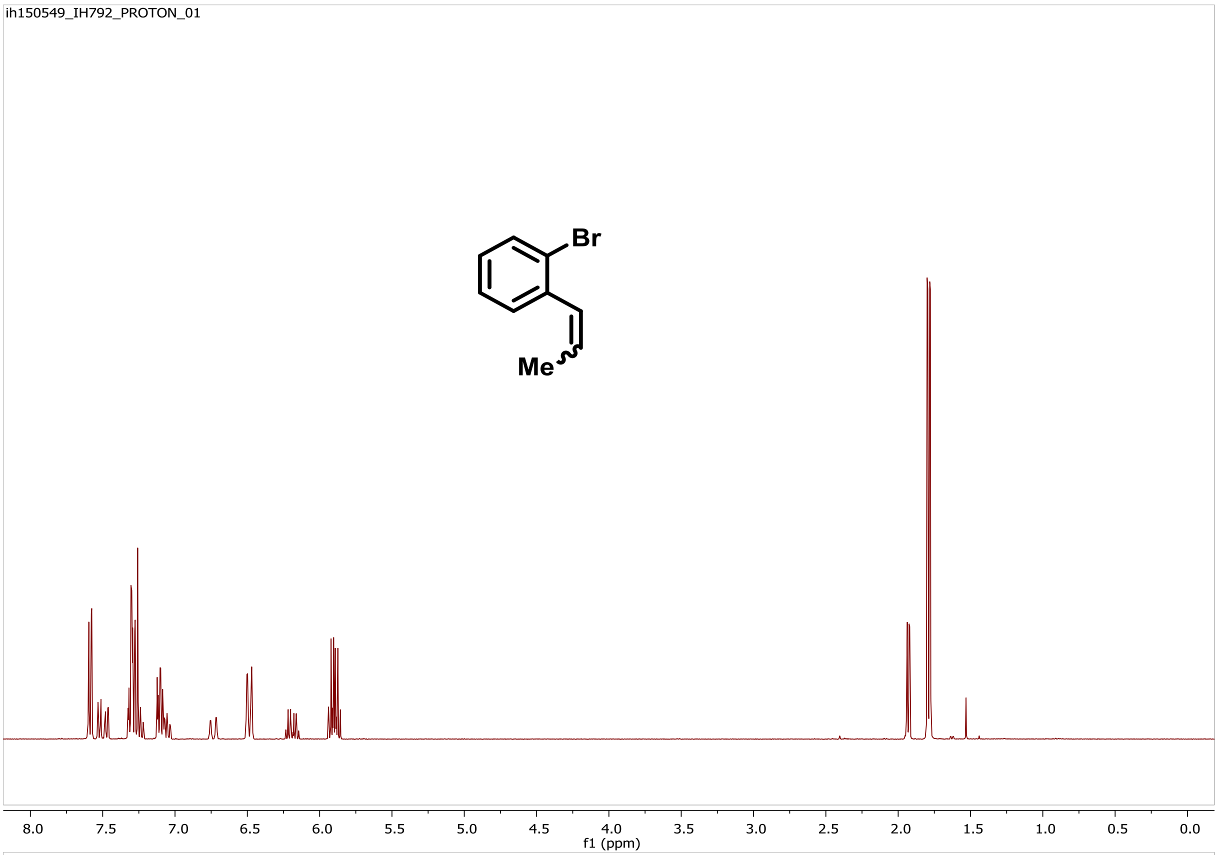
ih150549_IH792_PROTON_01
Br
Me
8.0 7.5 7.0 6.5 6.0 5.5 5.0 4.5 4.0 3.5 3.0 2.5 2.0 1.5 1.0 0.5 0.0
f1 (ppm)

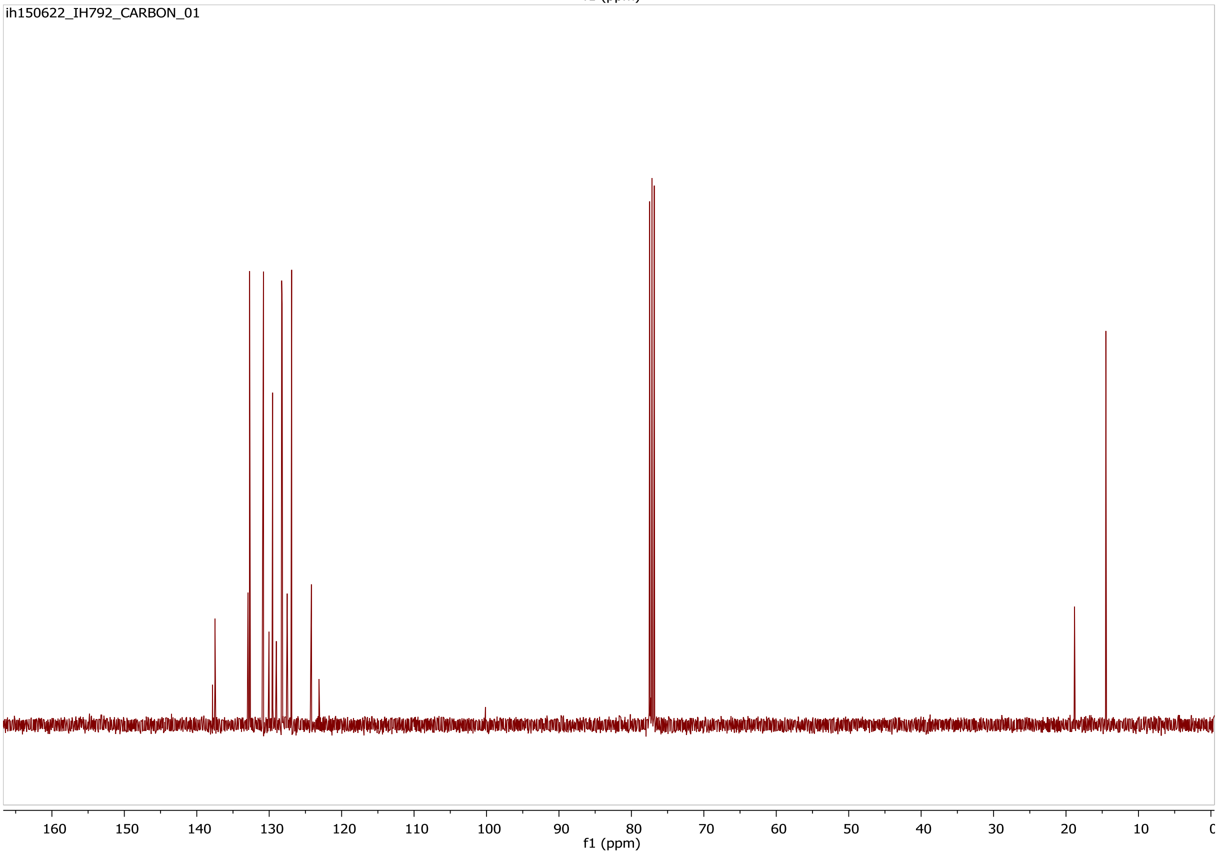
ih150622_IH792_CARBON_01
160 150 140 130 120 110 100 90 80 70 60 50 40 30 20 10
f1 (ppm)

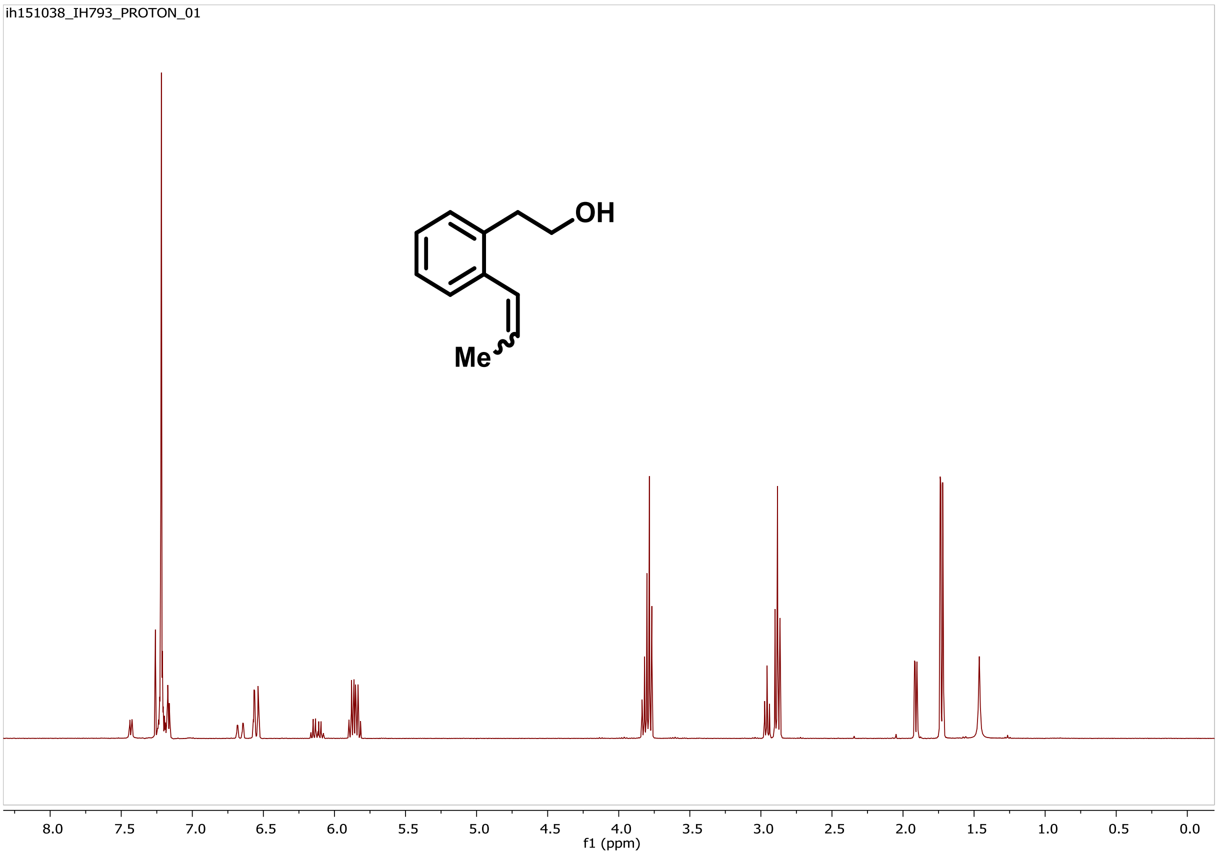
ih151038_IH793_PROTON_01
OH
Me
8.0 7.5 7.0 6.5 6.0 5.5 5.0 4.5 4.0 3.5 3.0 2.5 2.0 1.5 1.0 0.5 0.0
f1 (ppm)

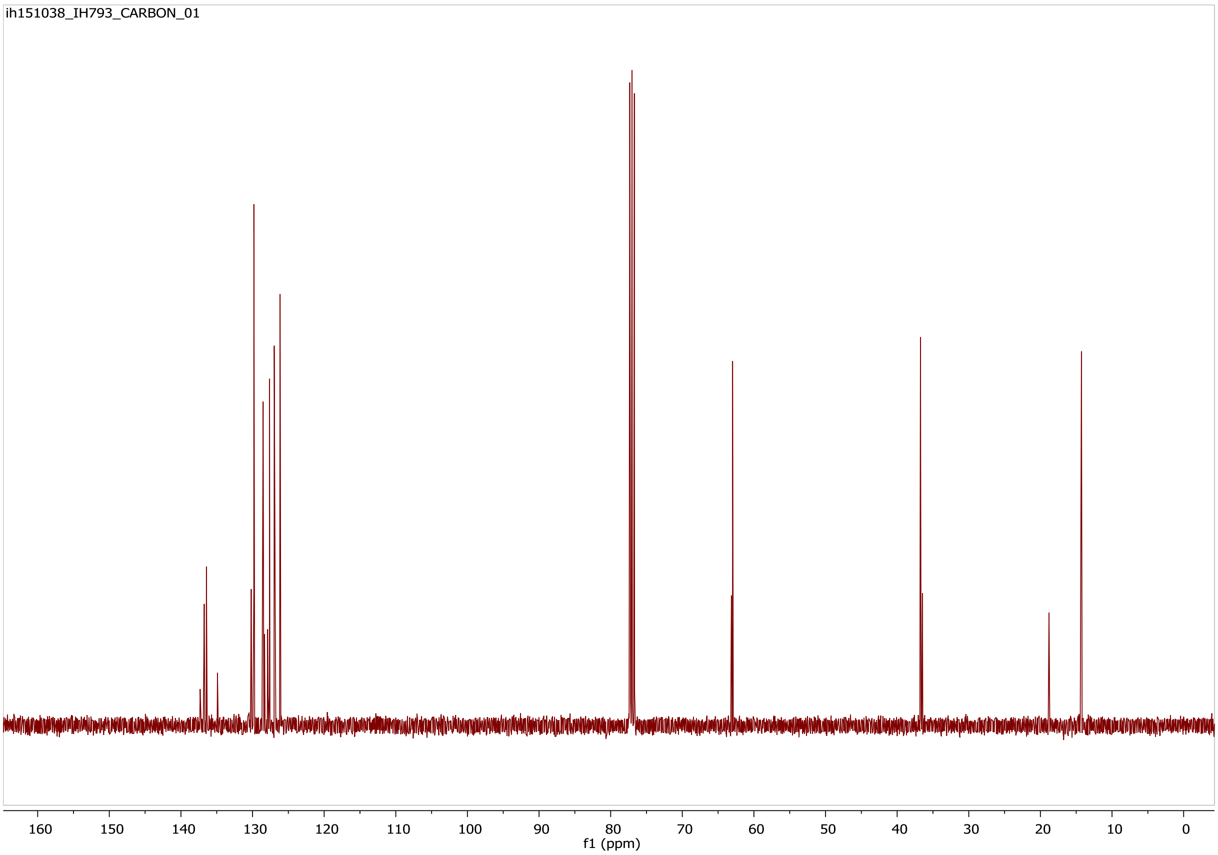
ih151038_IH793_CARBON_01
160 150 140 130 120 110 100 90 80 70 60 50 40 30 20 10 0
f1 (ppm)

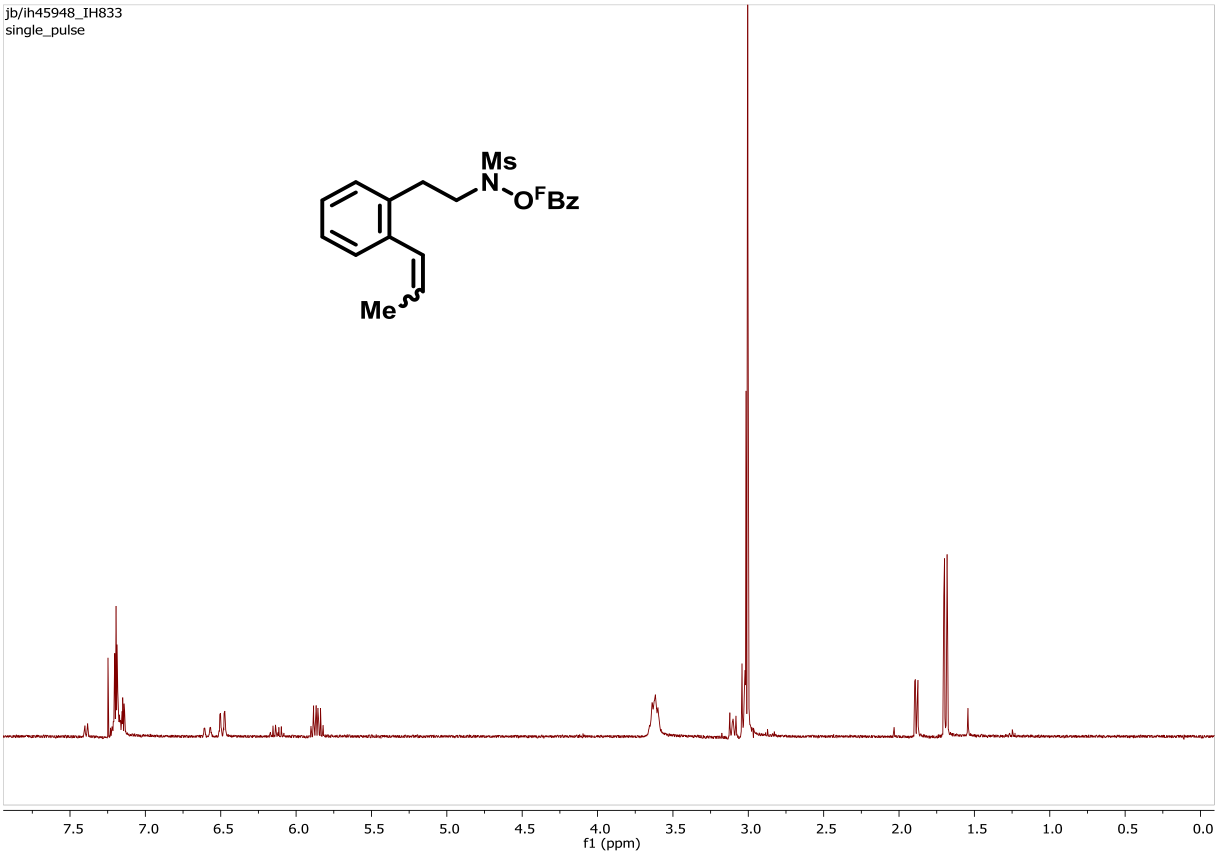
jb/ih45948_IH833
single_pulse
Ms
N
OFBz
Me
7.5 7.0 6.5 6.0 5.5 5.0 4.5 4.0 3.5 3.0 2.5 2.0 1.5 1.0 0.5 0.0
f1 (ppm)

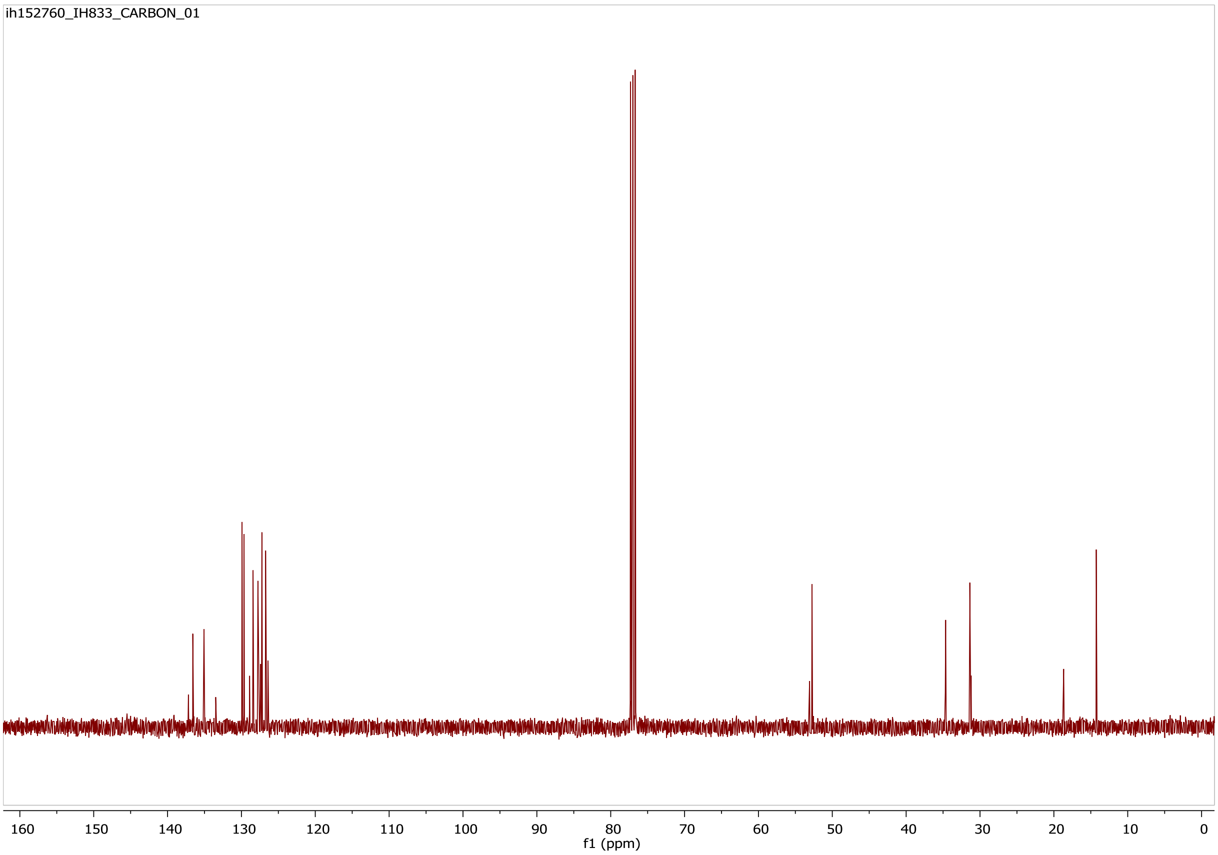
ih152760_IH833_CARBON_01
160 150 140 130 120 110 100 90 80 70 60 50 40 30 20 10 0
f1 (ppm)

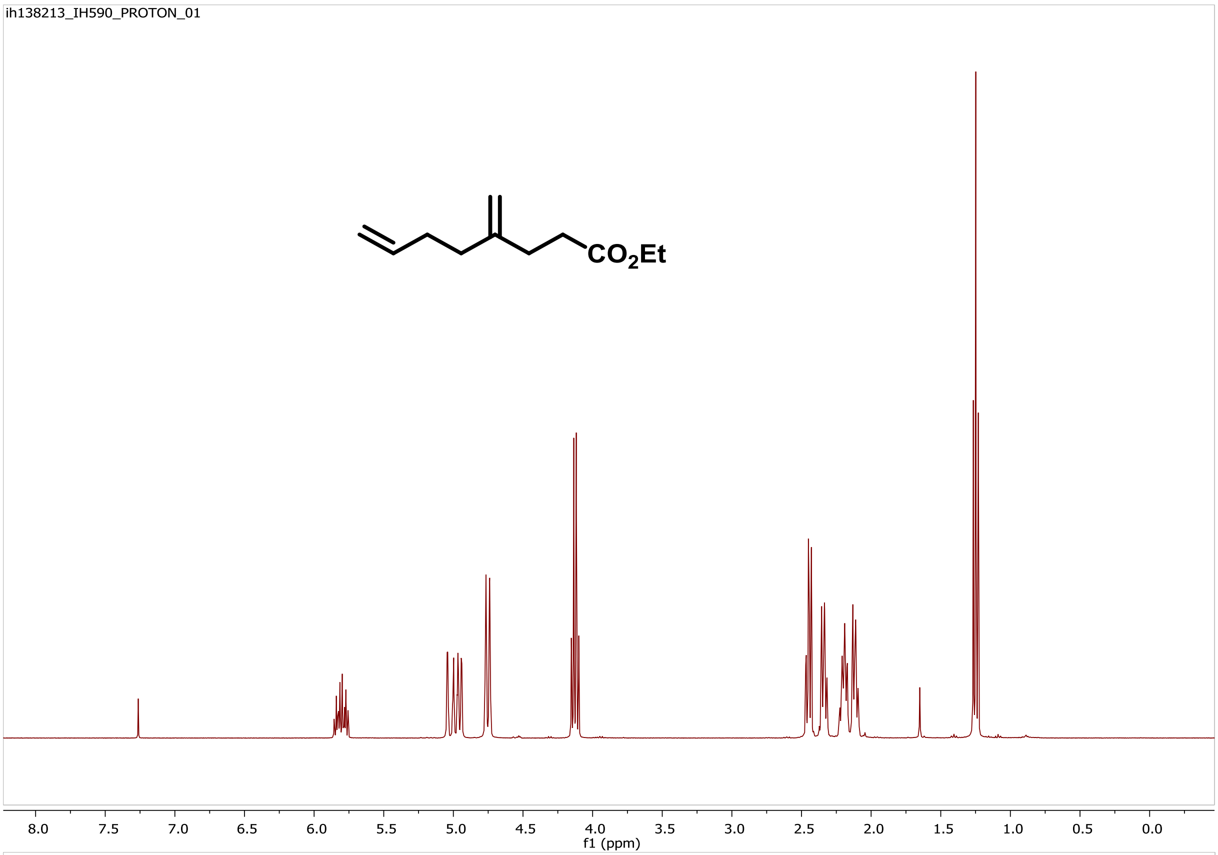
ih138213_IH590_PROTON_01
CO2Et
8.0 7.5 7.0 6.5 6.0 5.5 5.0 4.5 4.0 3.5 3.0 2.5 2.0 1.5 1.0 0.5 0.0
f1 (ppm)

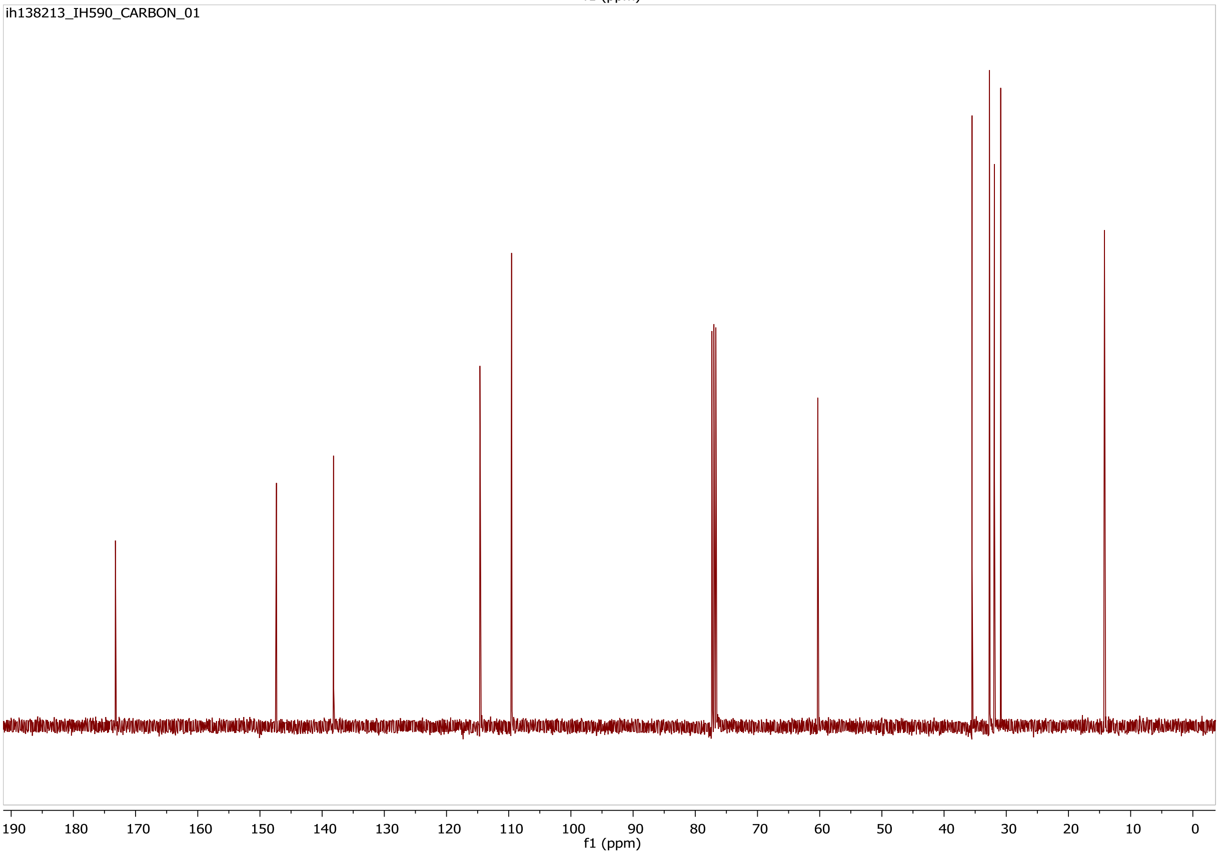
ih138213_IH590_CARBON_01
190 180 170 160 150 140 130 120 110 100 90 80 70 60 50 40 30 20 10 0
f1 (ppm)

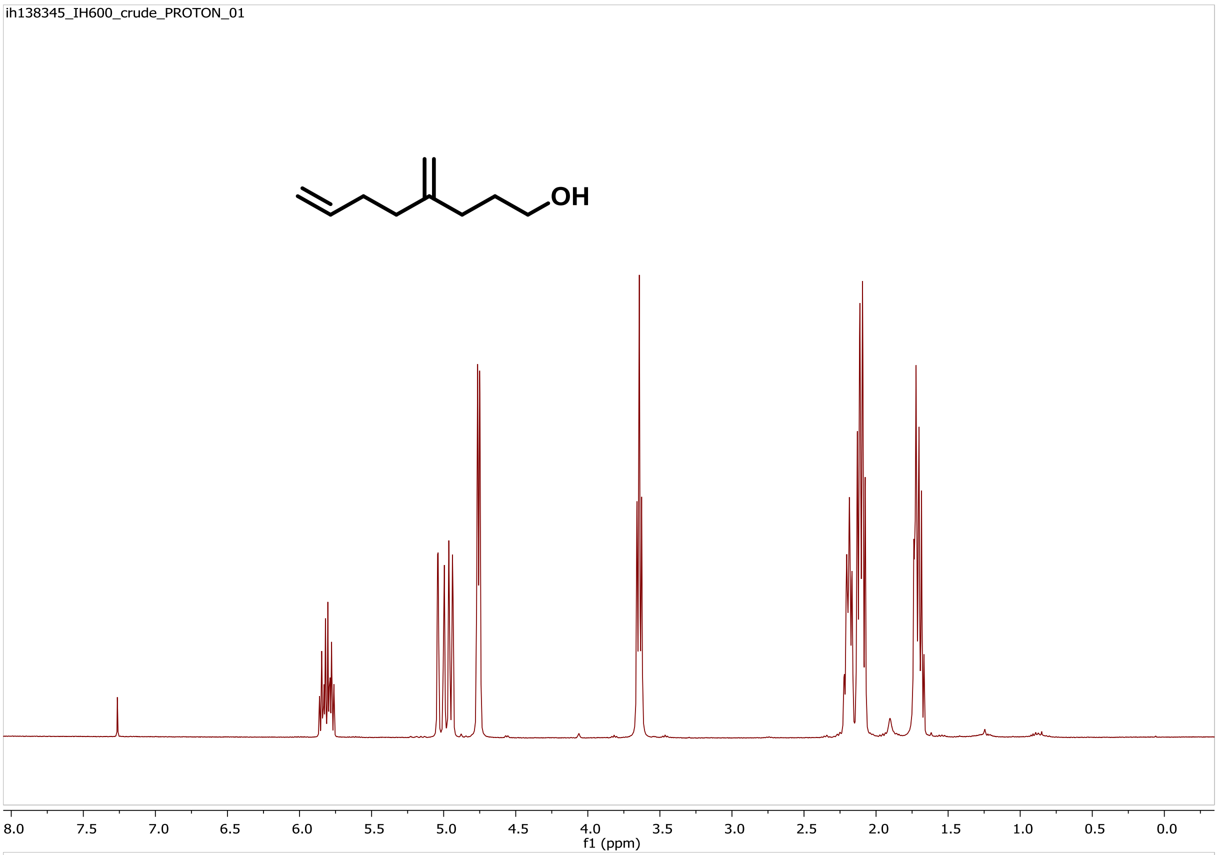
ih138345_IH600_crude_PROTON_01
OH
8.0 7.5 7.0 6.5 6.0 5.5 5.0 4.5 4.0 3.5 3.0 2.5 2.0 1.5 1.0 0.5 0.0
f1 (ppm)

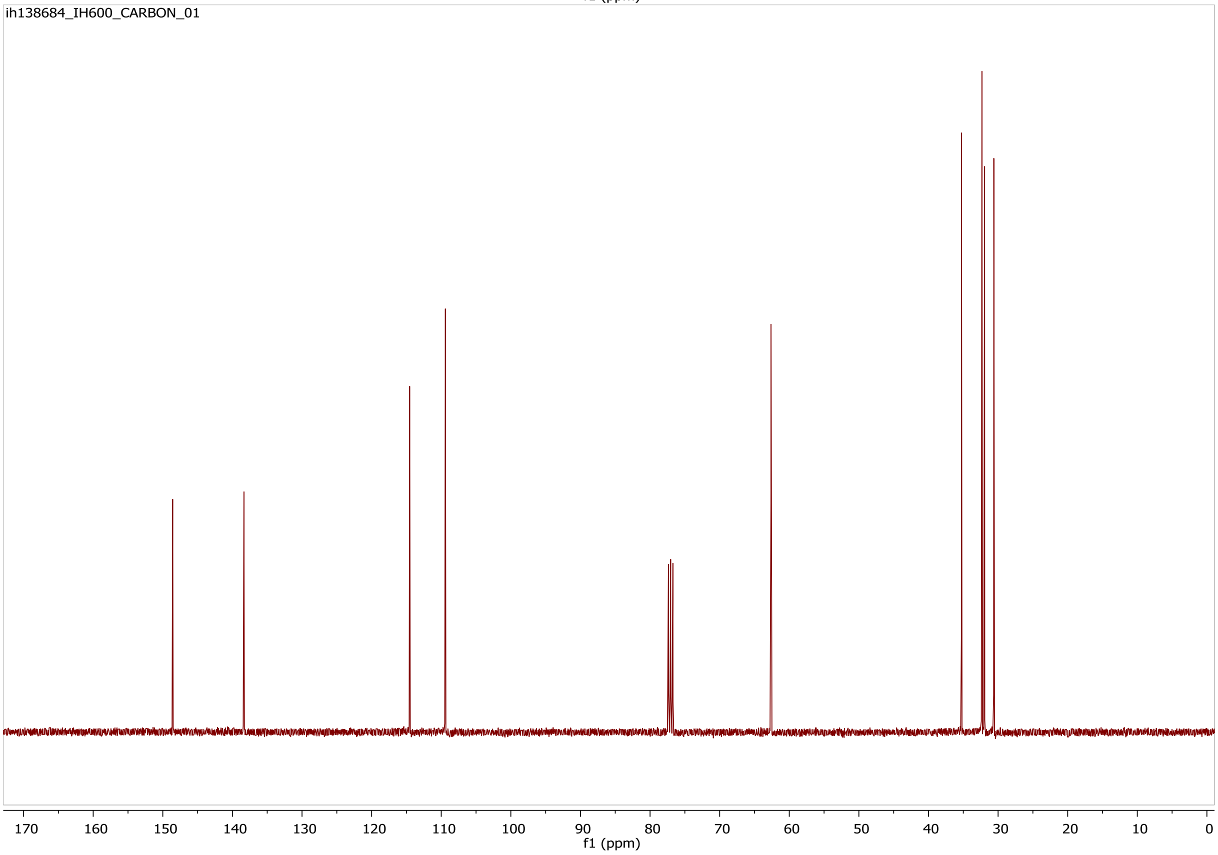
ih138684_IH600_CARBON_01
170 160 150 140 130 120 110 100 90 80 70 60 50 40 30 20 10 0
f1 (ppm)

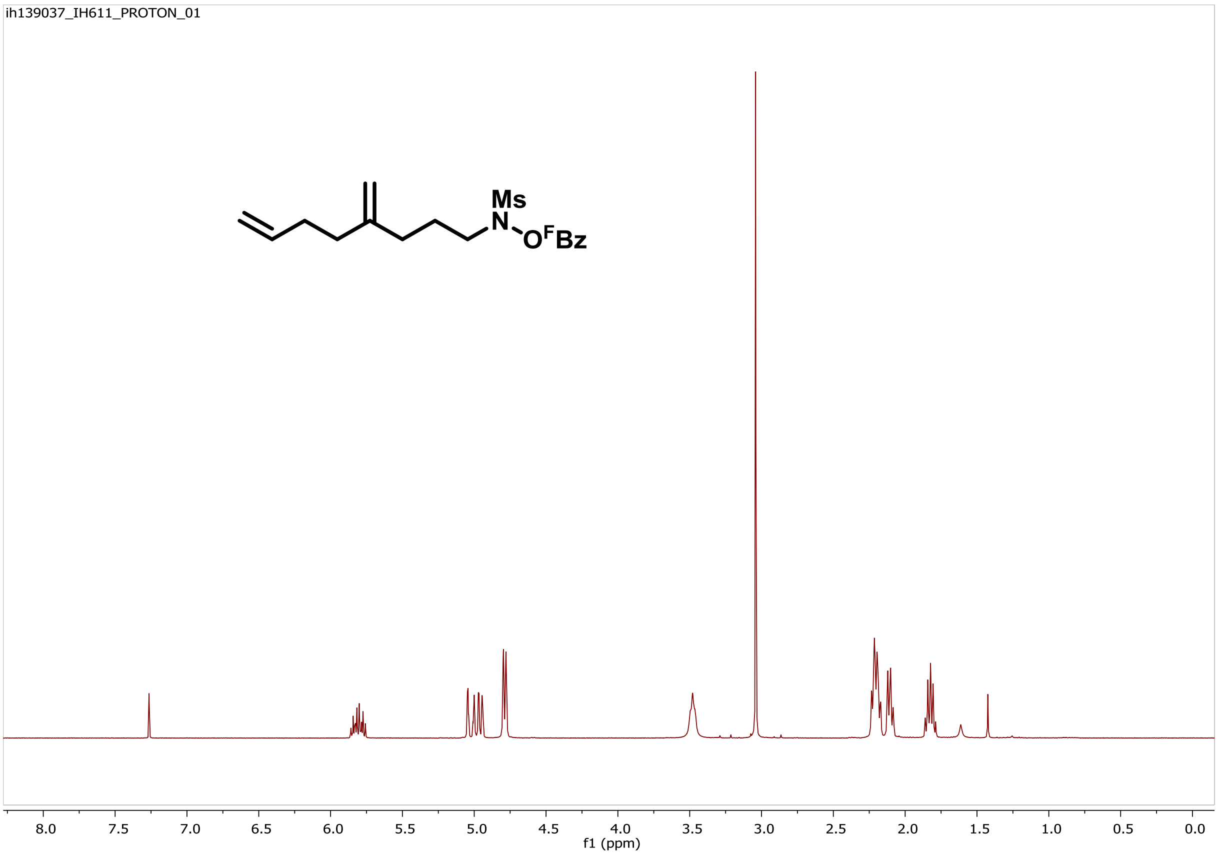
ih139037_IH611_PROTON_01
Ms
N
O
F
Bz
8.0 7.5 7.0 6.5 6.0 5.5 5.0 4.5 4.0 3.5 3.0 2.5 2.0 1.5 1.0 0.5 0.0
f1 (ppm)

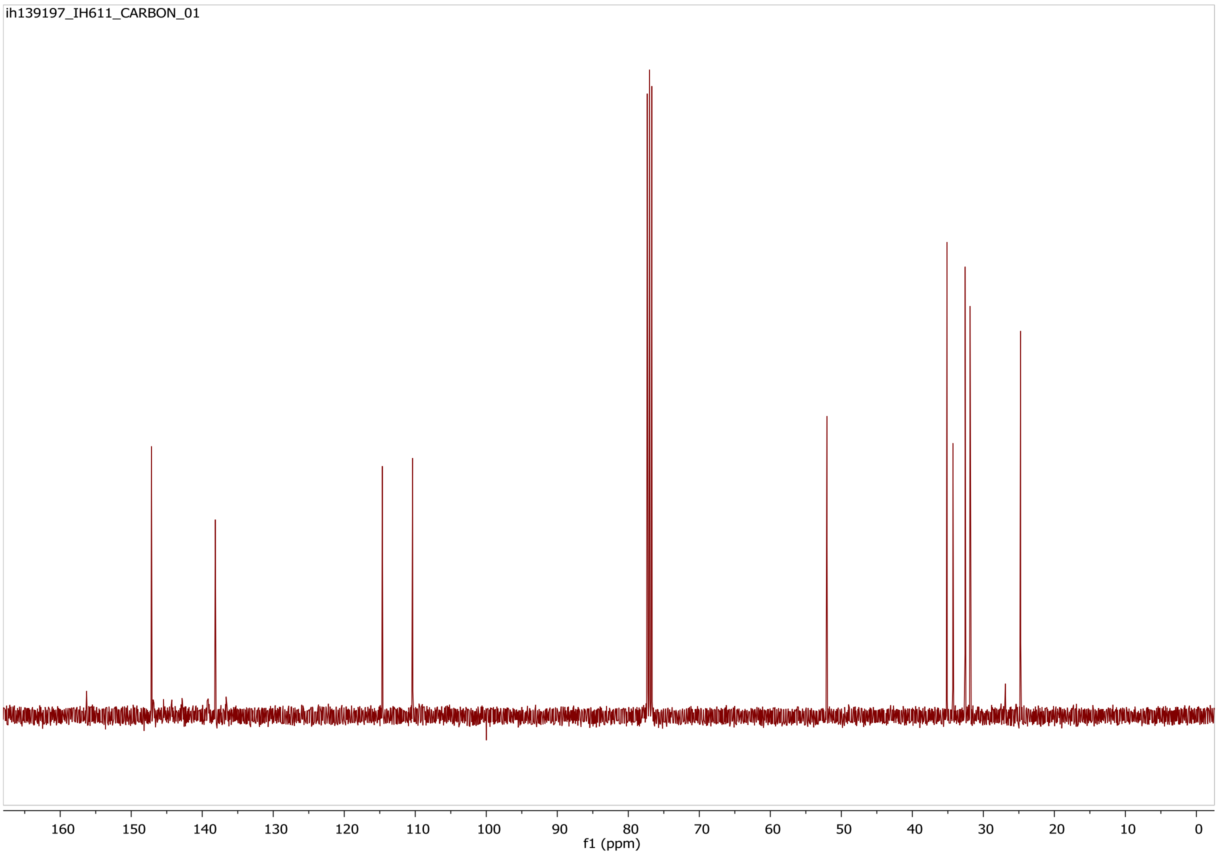
ih139197_IH611_CARBON_01
160 150 140 130 120 110 100 90 80 70 60 50 40 30 20 10 0
f1 (ppm)

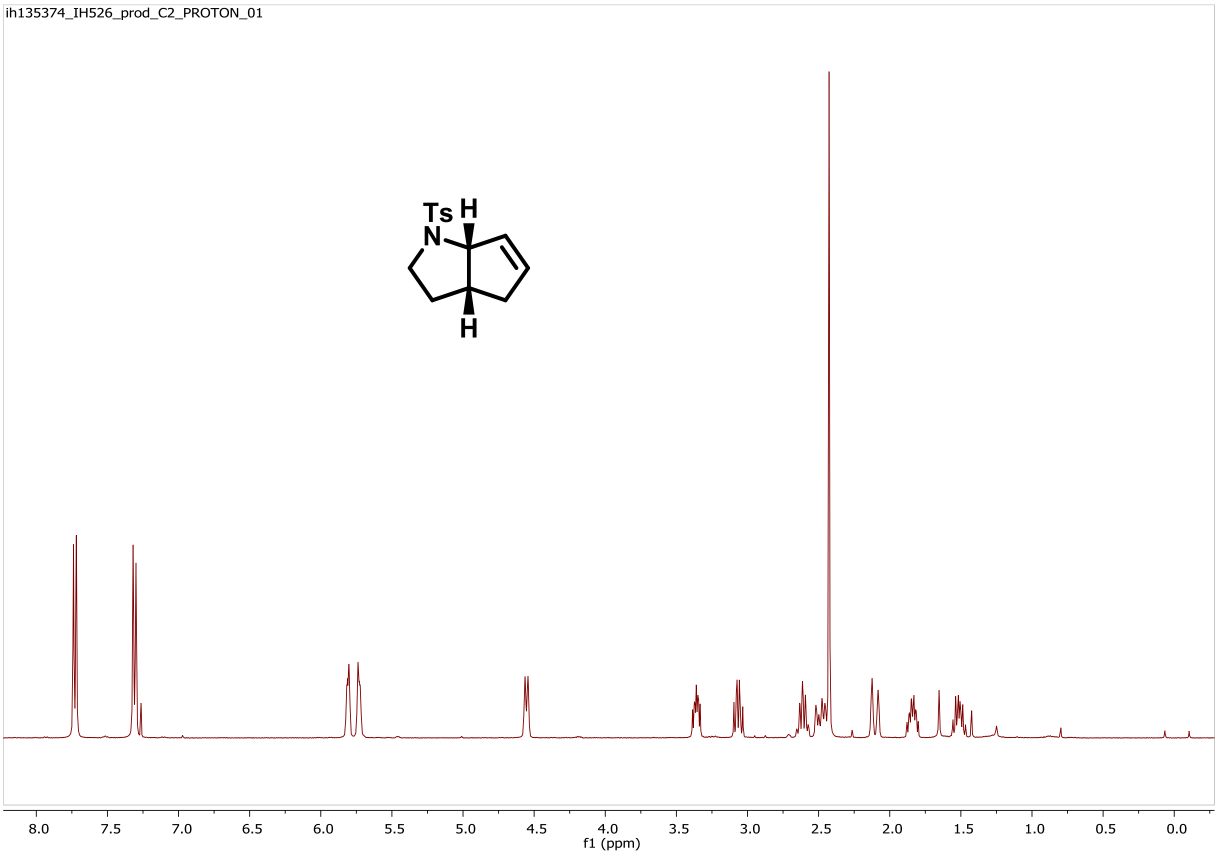
ih135374_IH526_prod_C2_PROTON_01
Ts
N
H
H
8.0
7.5
7.0
6.5
6.0
5.5
5.0
4.5
4.0
3.5
3.0
2.5
2.0
1.5
1.0
0.5
0.0
f1 (ppm)

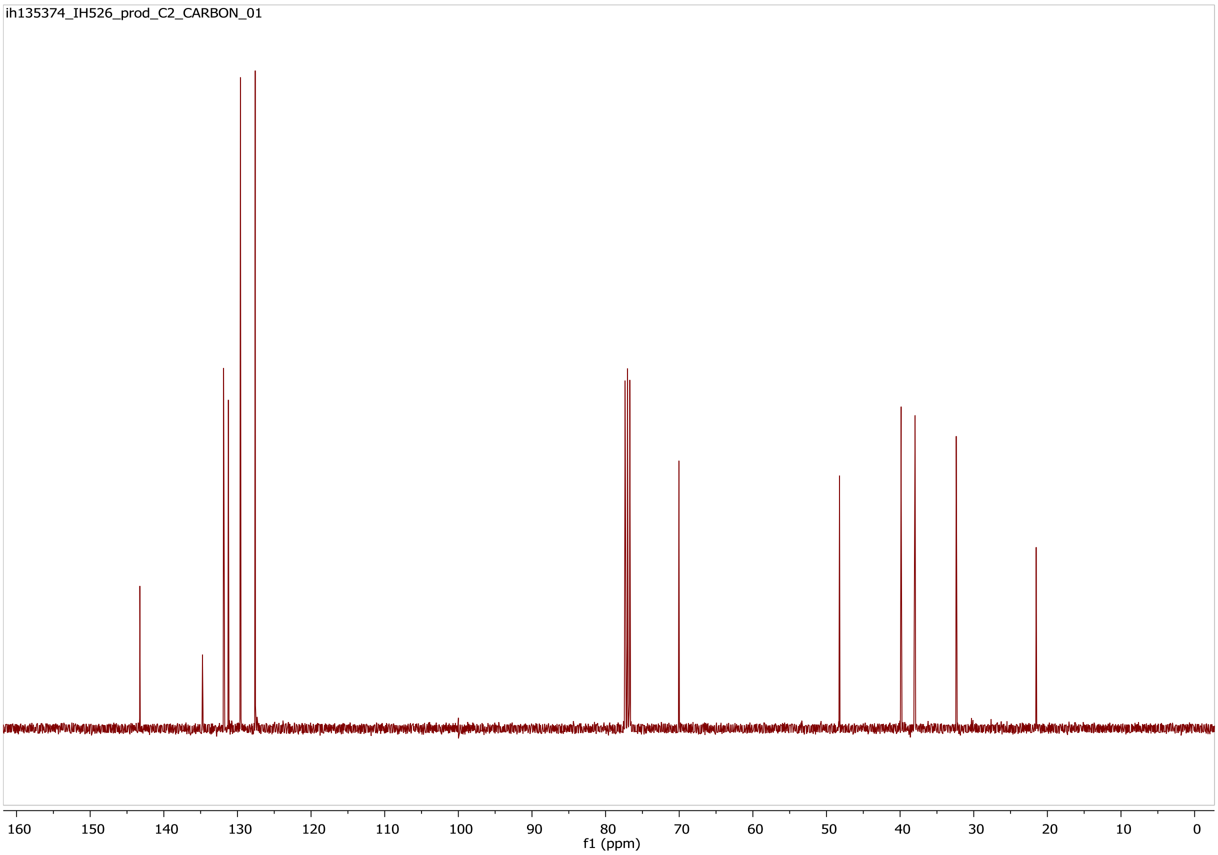
ih135374_IH526_prod_C2_CARBON_01
160
150
140
130
120
110
100
90
80
70
60
50
40
30
20
10
0
f1 (ppm)

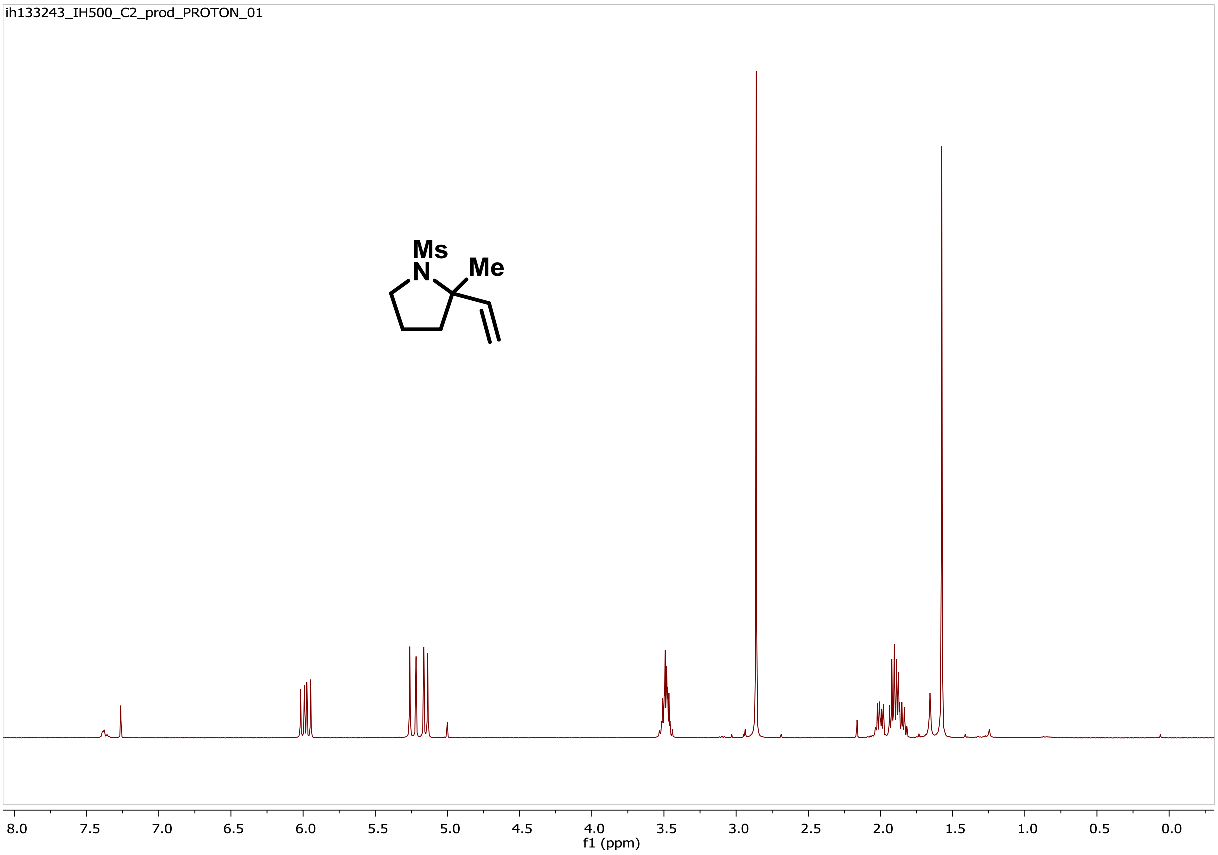
ih133243_IH500_C2_prod_PROTON_01
Ms
N
Me
8.0
7.5
7.0
6.5
6.0
5.5
5.0
4.5
4.0
3.5
3.0
2.5
2.0
1.5
1.0
0.5
0.0
f1 (ppm)

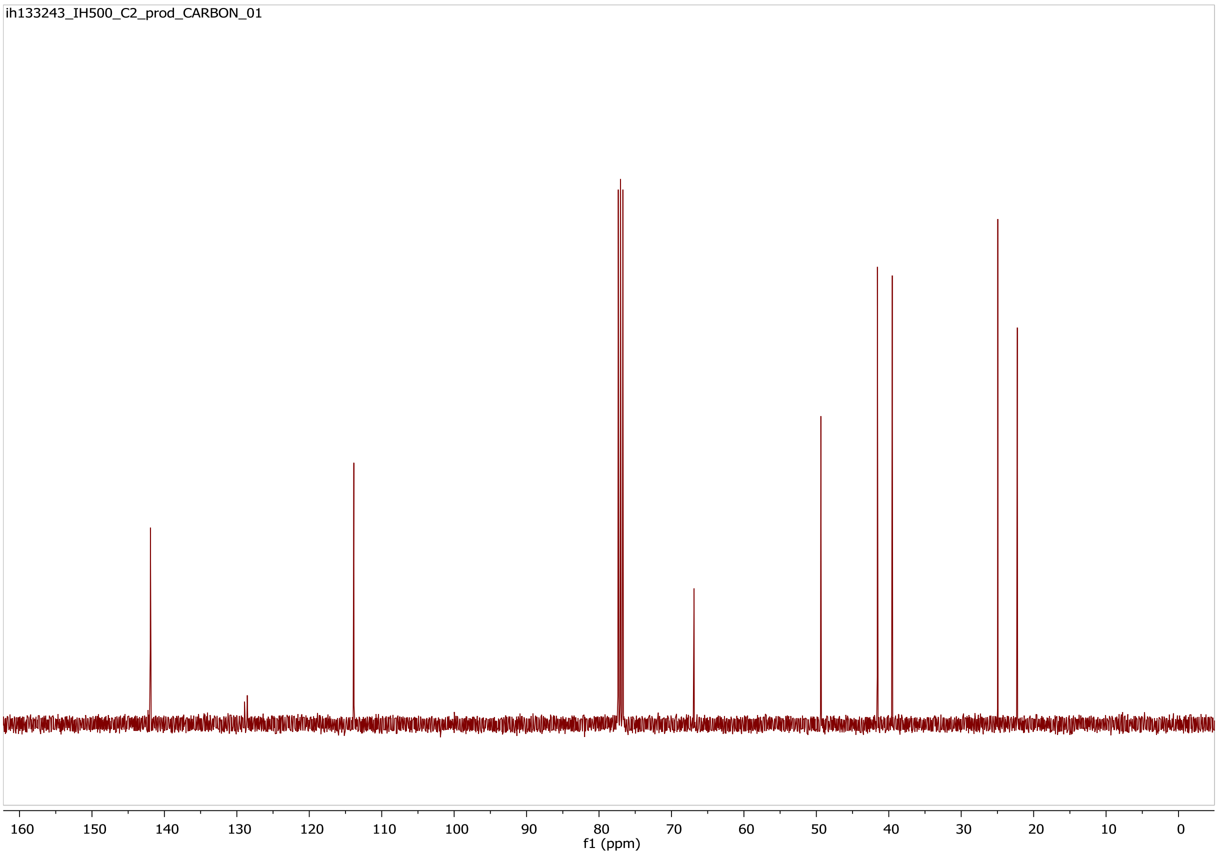
ih133243_IH500_C2_prod_CARBON_01
160
150
140
130
120
110
100
90
80
70
60
50
40
30
20
10
0
f1 (ppm)

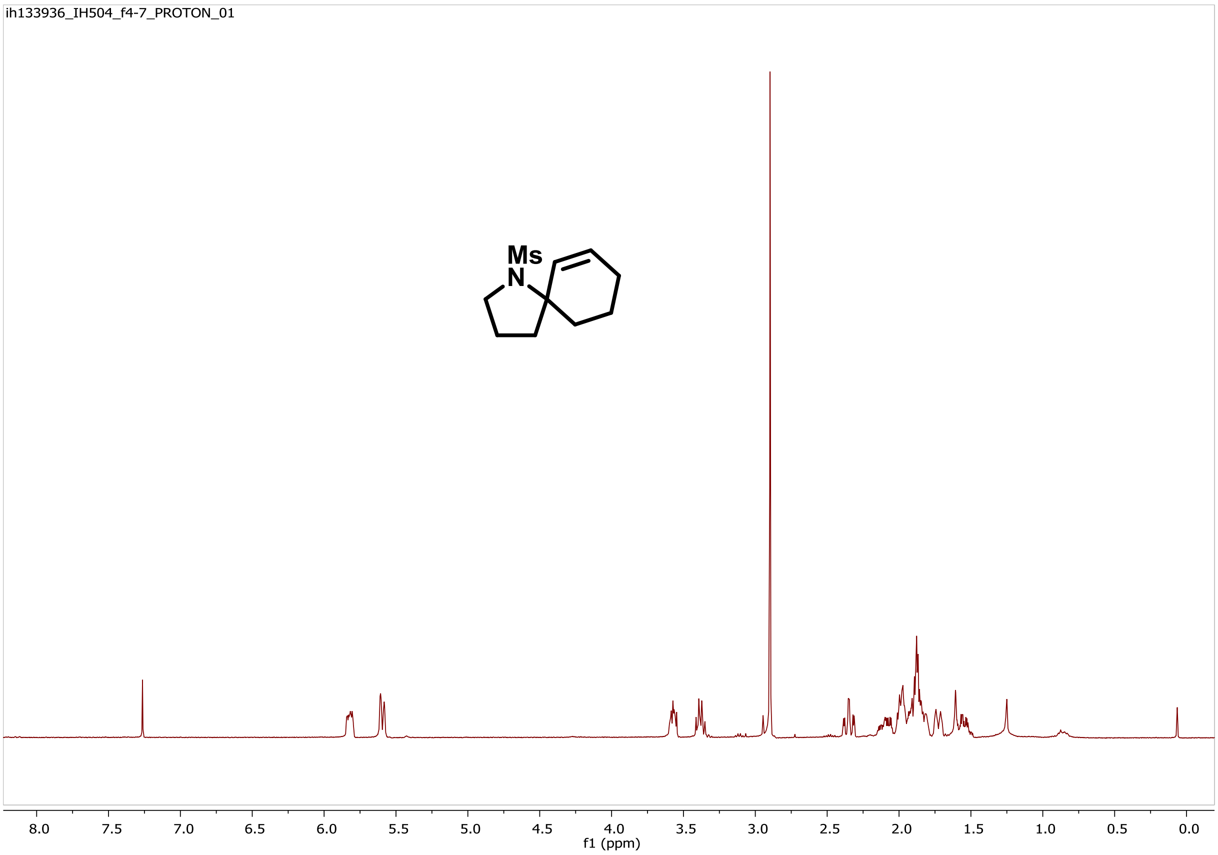
ih133936_IH504_f4-7_PROTON_01
Ms
N
8.0 7.5 7.0 6.5 6.0 5.5 5.0 4.5 4.0 3.5 3.0 2.5 2.0 1.5 1.0 0.5 0.0
f1 (ppm)

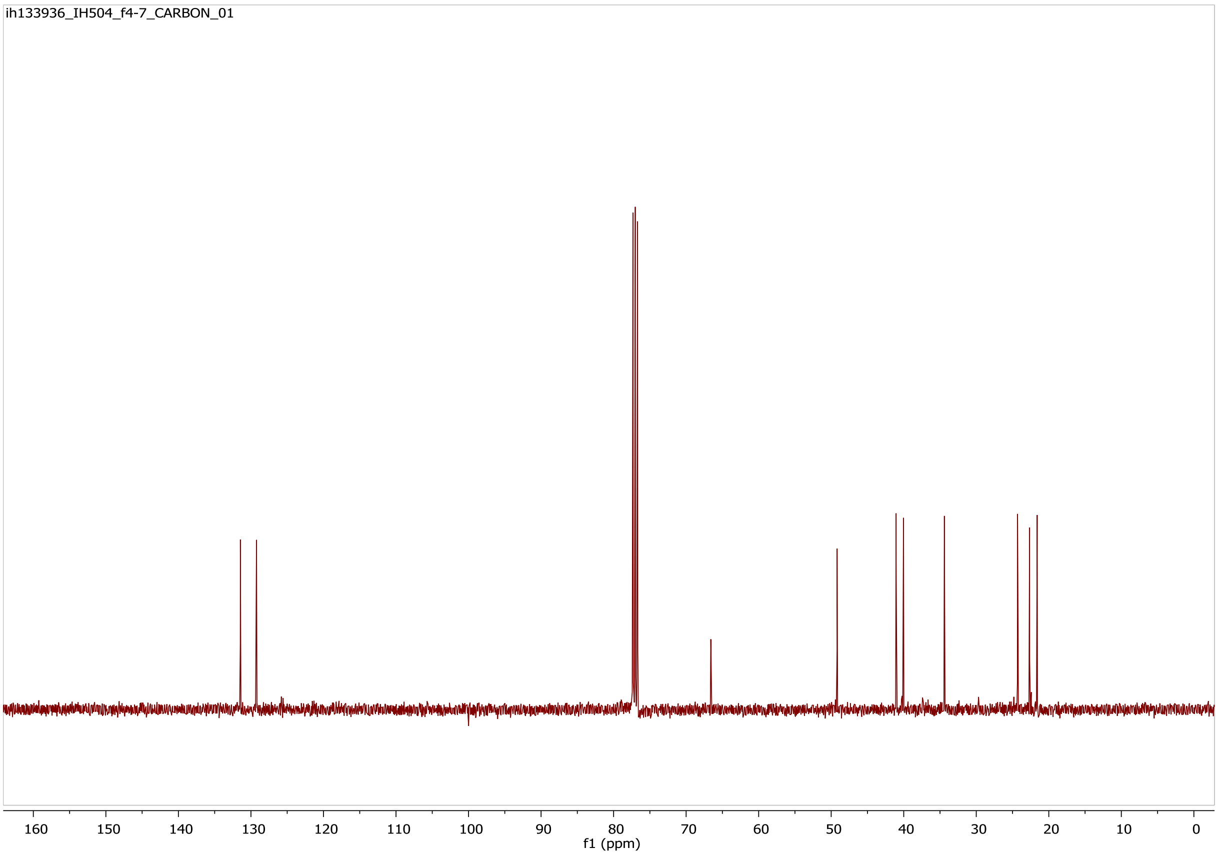
ih133936_IH504_f4-7_CARBON_01
160 150 140 130 120 110 100 90 80 70 60 50 40 30 20 10 0
f1 (ppm)

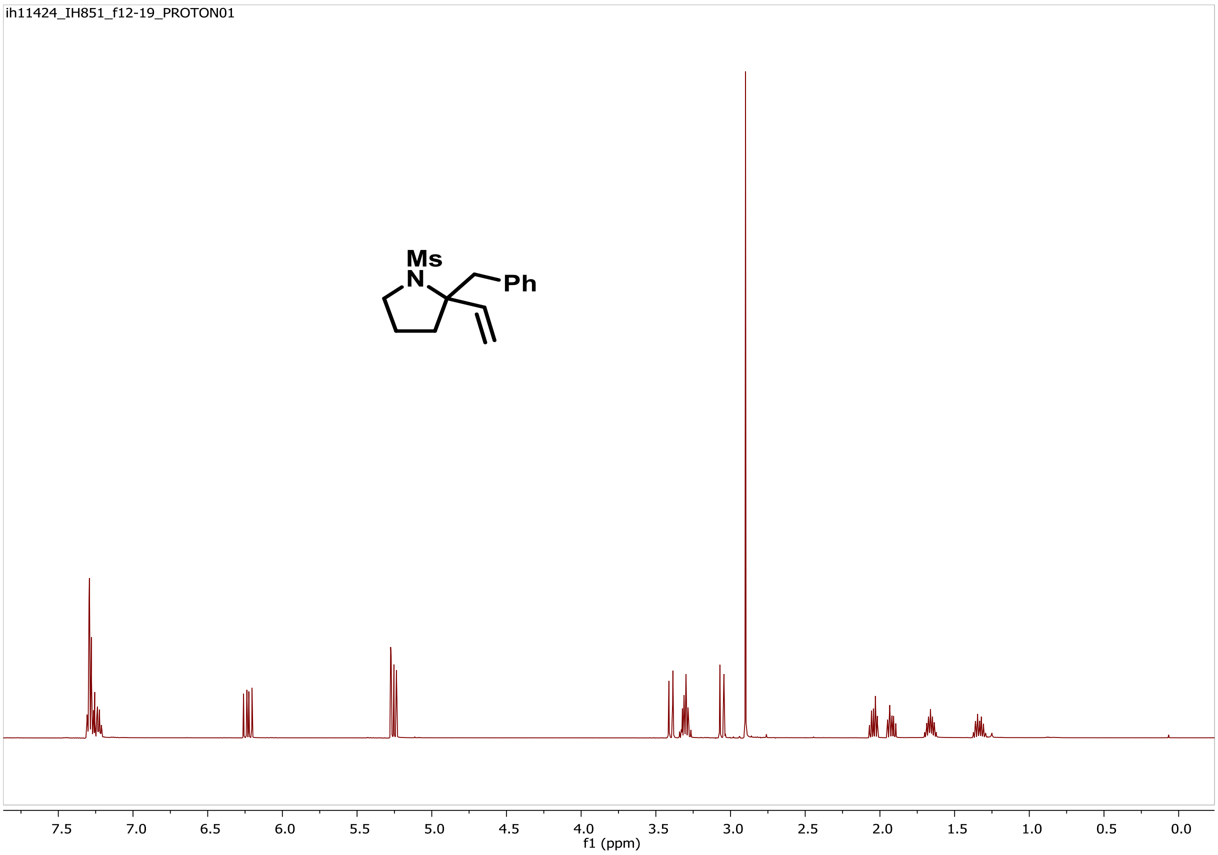
ih11424_IH851_f12-19_PROTON01
Ms
N
Ph
7.5 7.0 6.5 6.0 5.5 5.0 4.5 4.0 3.5 3.0 2.5 2.0 1.5 1.0 0.5 0.0
f1 (ppm)

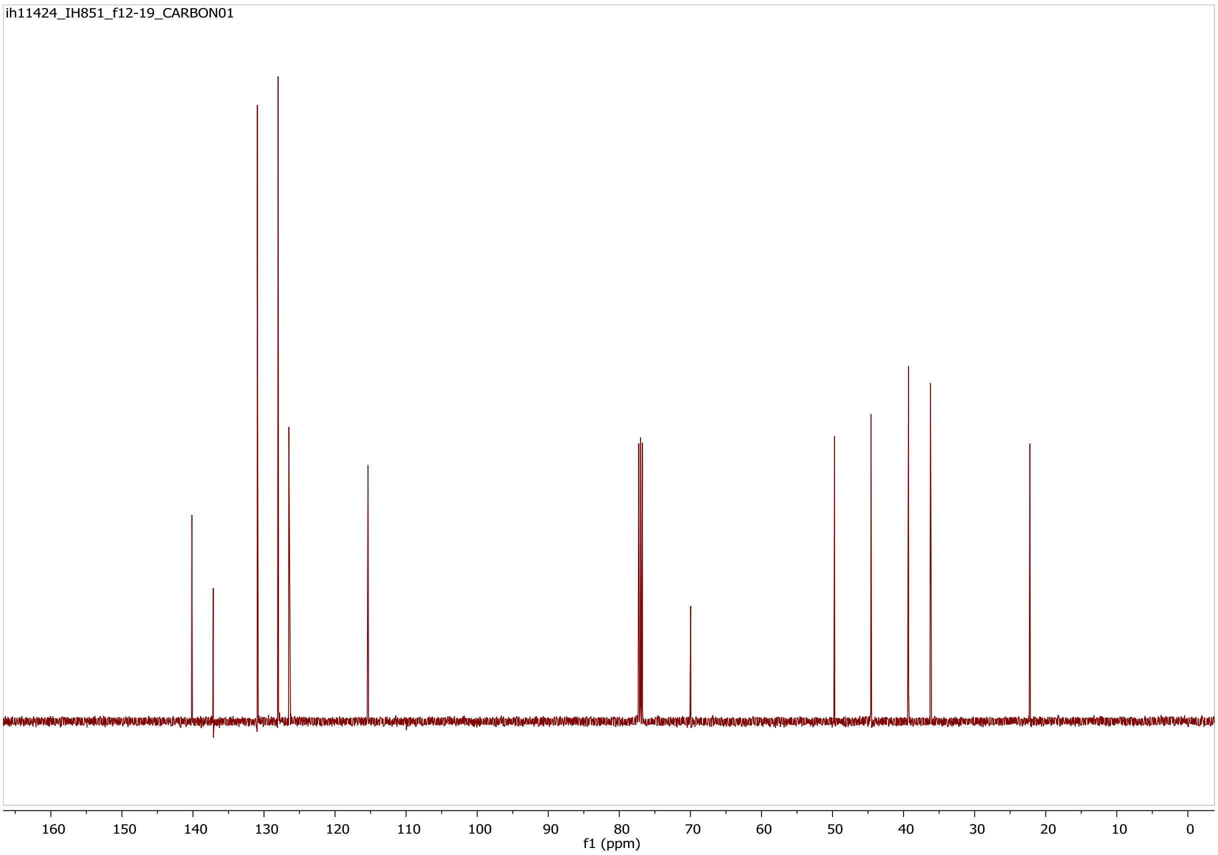
ih11424_IH851_f12-19_CARBON01
160 150 140 130 120 110 100 90 80 70 60 50 40 30 20 10 0
f1 (ppm)

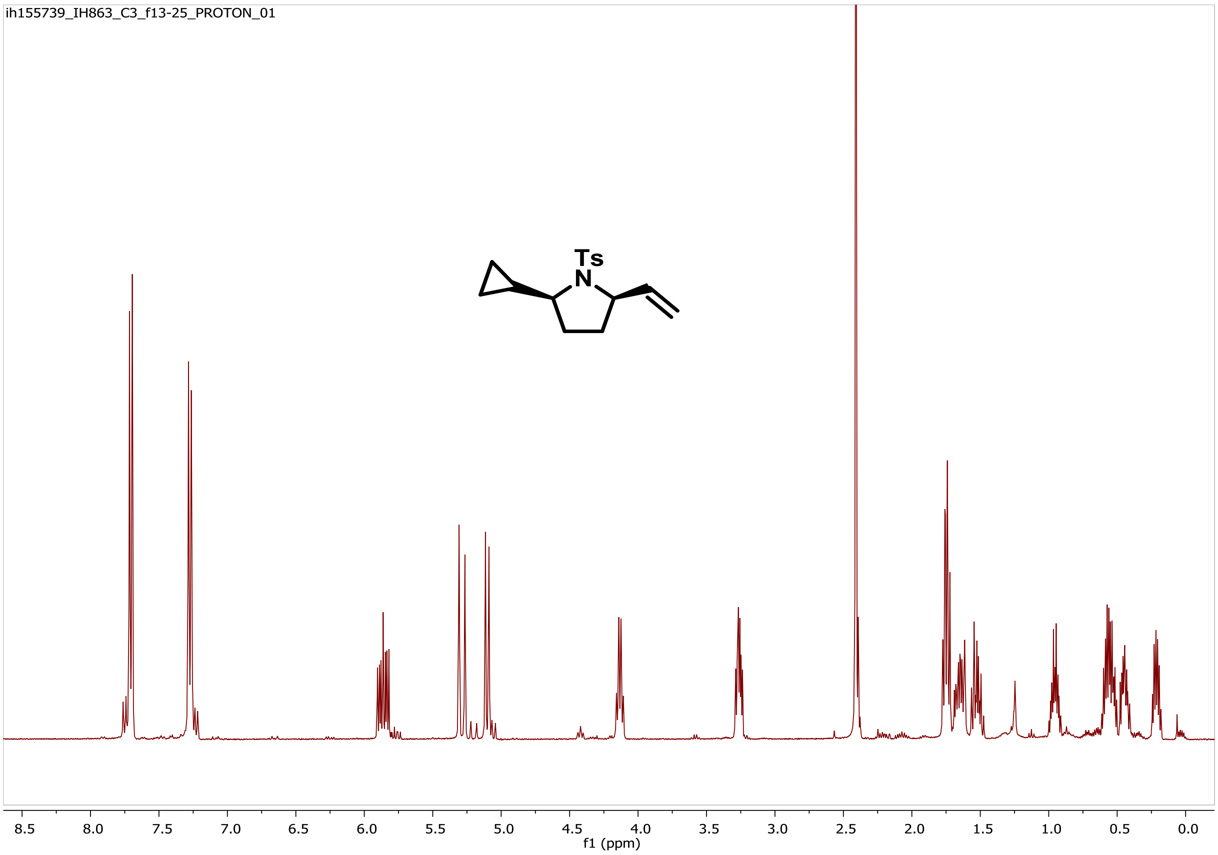
ih155739_IH863_C3_f13-25_PROTON_01
Ts
N
8.5
8.0
7.5
7.0
6.5
6.0
5.5
5.0
4.5
4.0
3.5
3.0
2.5
2.0
1.5
1.0
0.5
0.0
f1 (ppm)

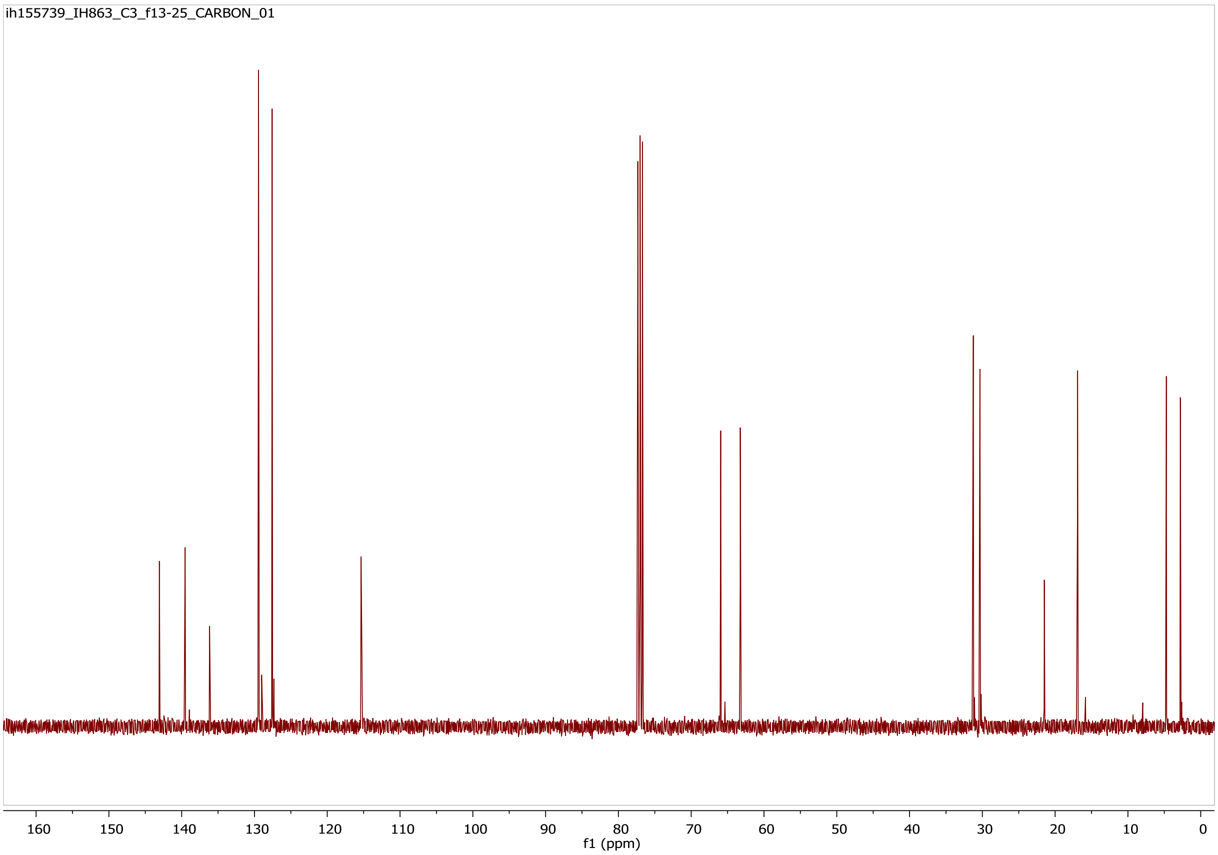
ih155739_IH863_C3_f13-25_CARBON_01
160
150
140
130
120
110
100
90
80
70
60
50
40
30
20
10
0
f1 (ppm)

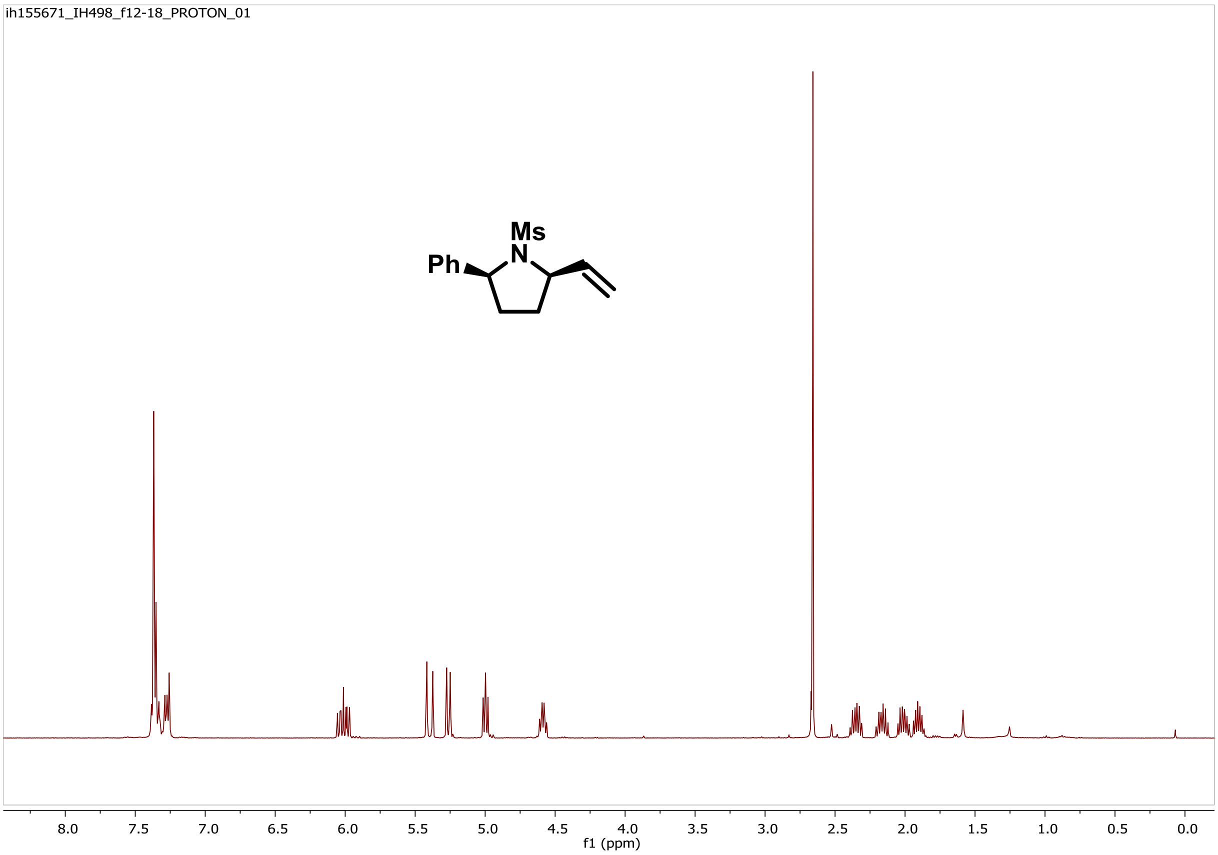
ih155671_IH498_f12-18_PROTON_01
Ms
N
Ph
f1 (ppm)

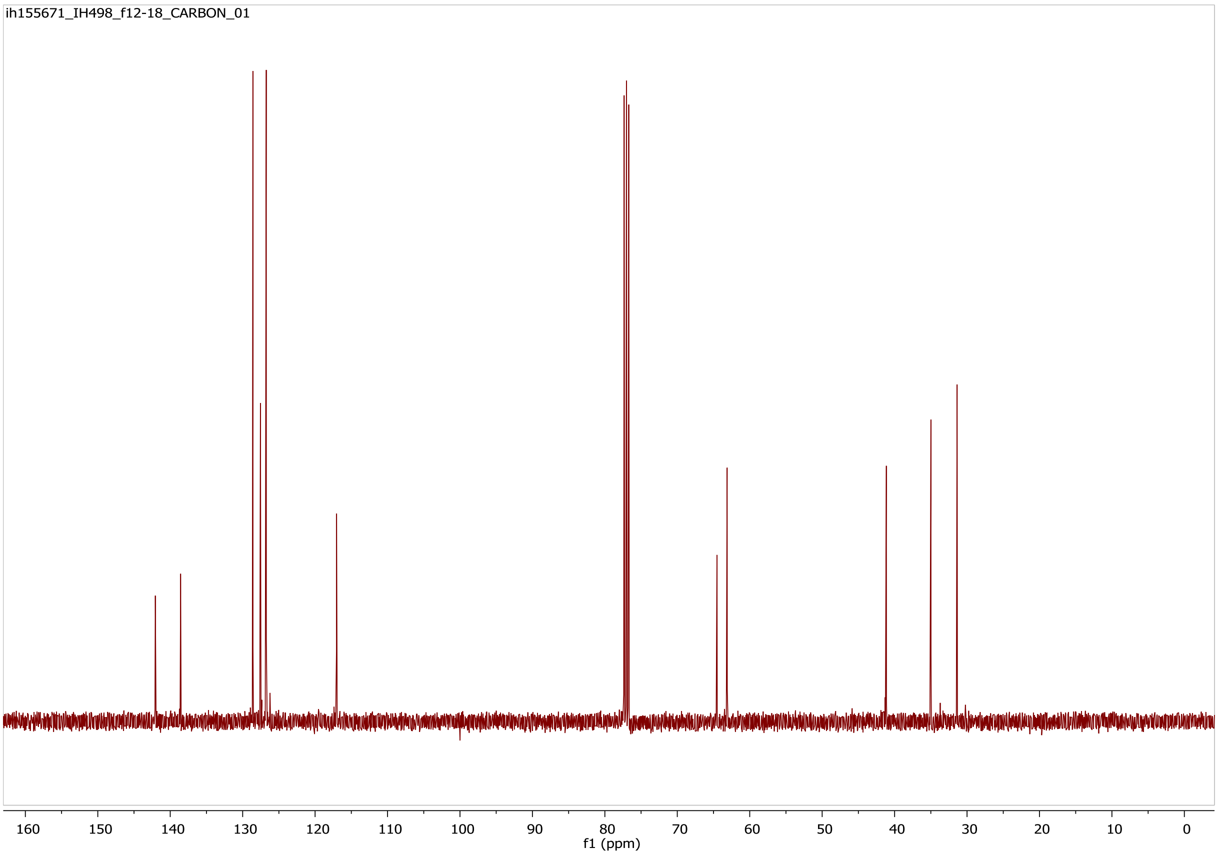
ih155671_IH498_f12-18_CARBON_01
f1 (ppm)

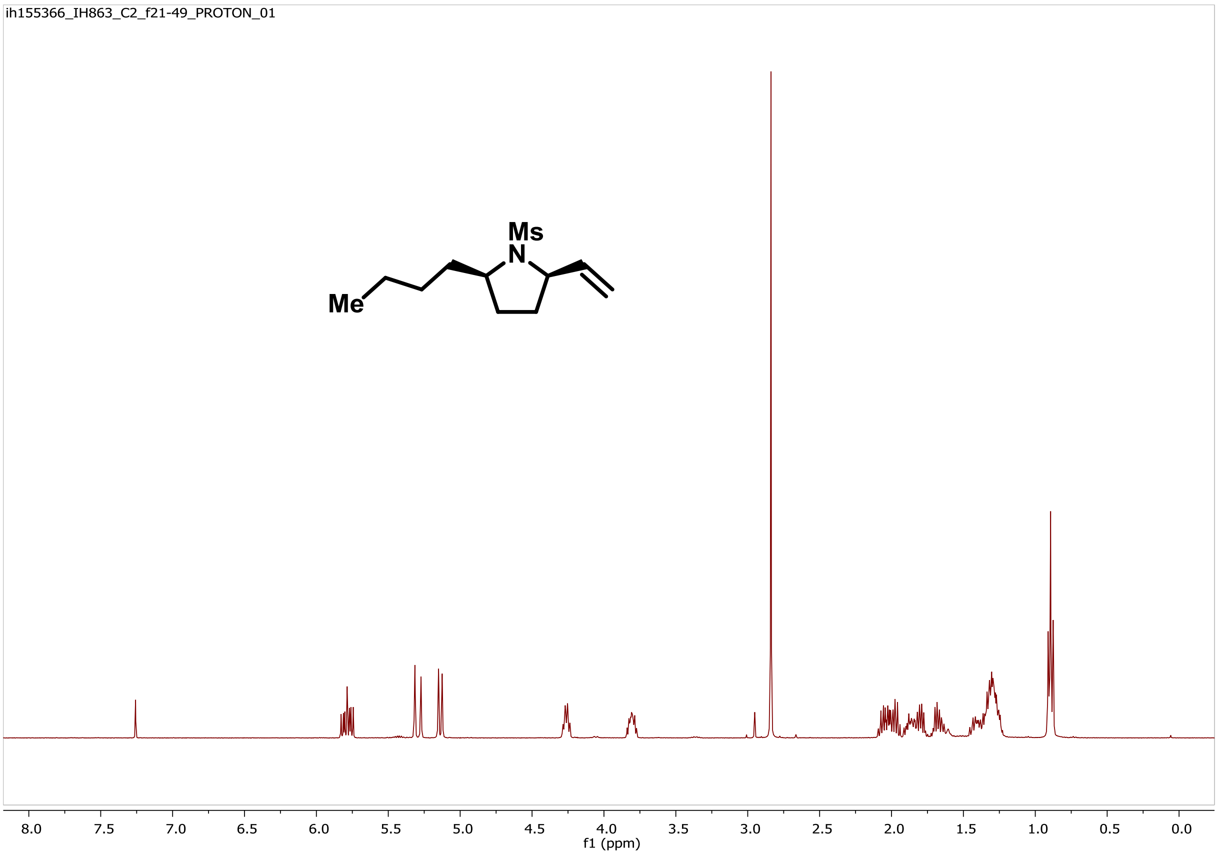
ih155366_IH863_C2_f21-49_PROTON_01
Ms
N
Me
8.0
7.5
7.0
6.5
6.0
5.5
5.0
4.5
4.0
3.5
3.0
2.5
2.0
1.5
1.0
0.5
0.0
f1 (ppm)

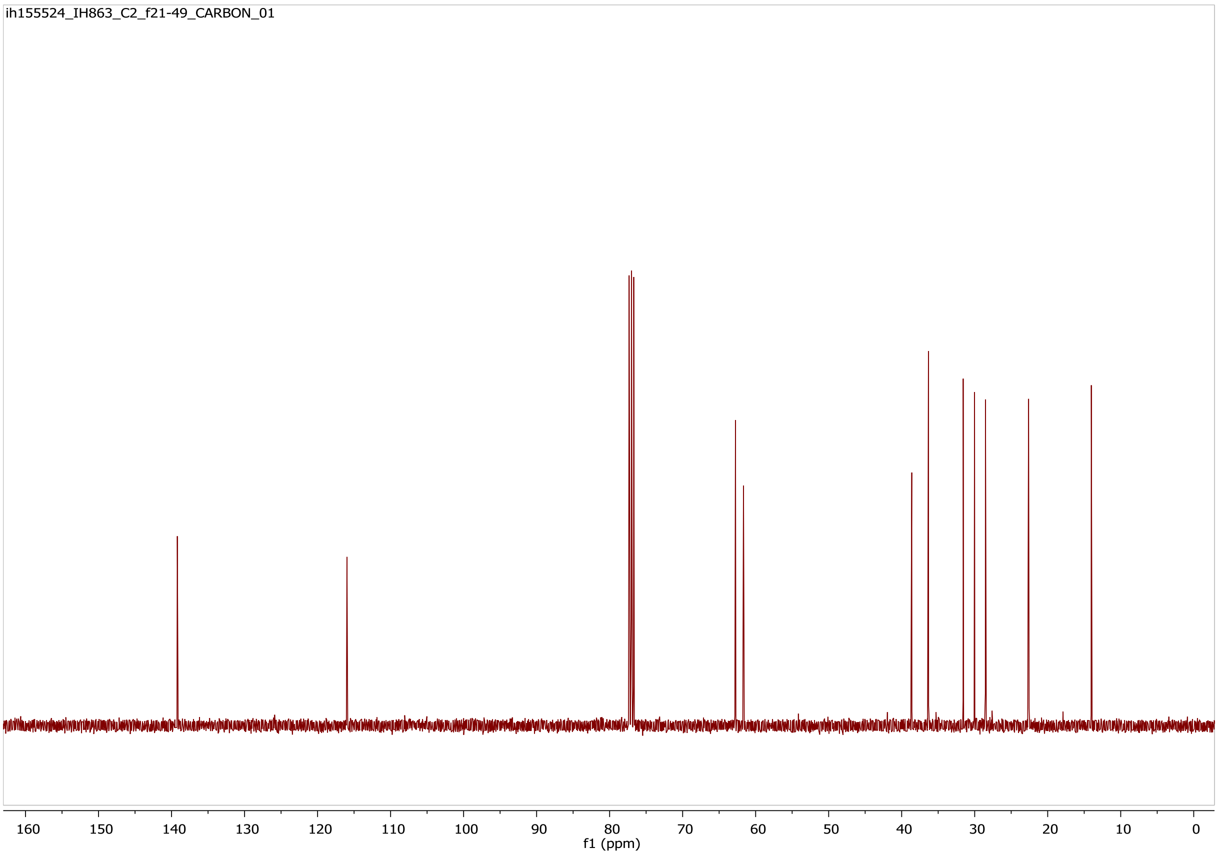
ih155524_IH863_C2_f21-49_CARBON_01
160
150
140
130
120
110
100
90
80
70
60
50
40
30
20
10
0
f1 (ppm)

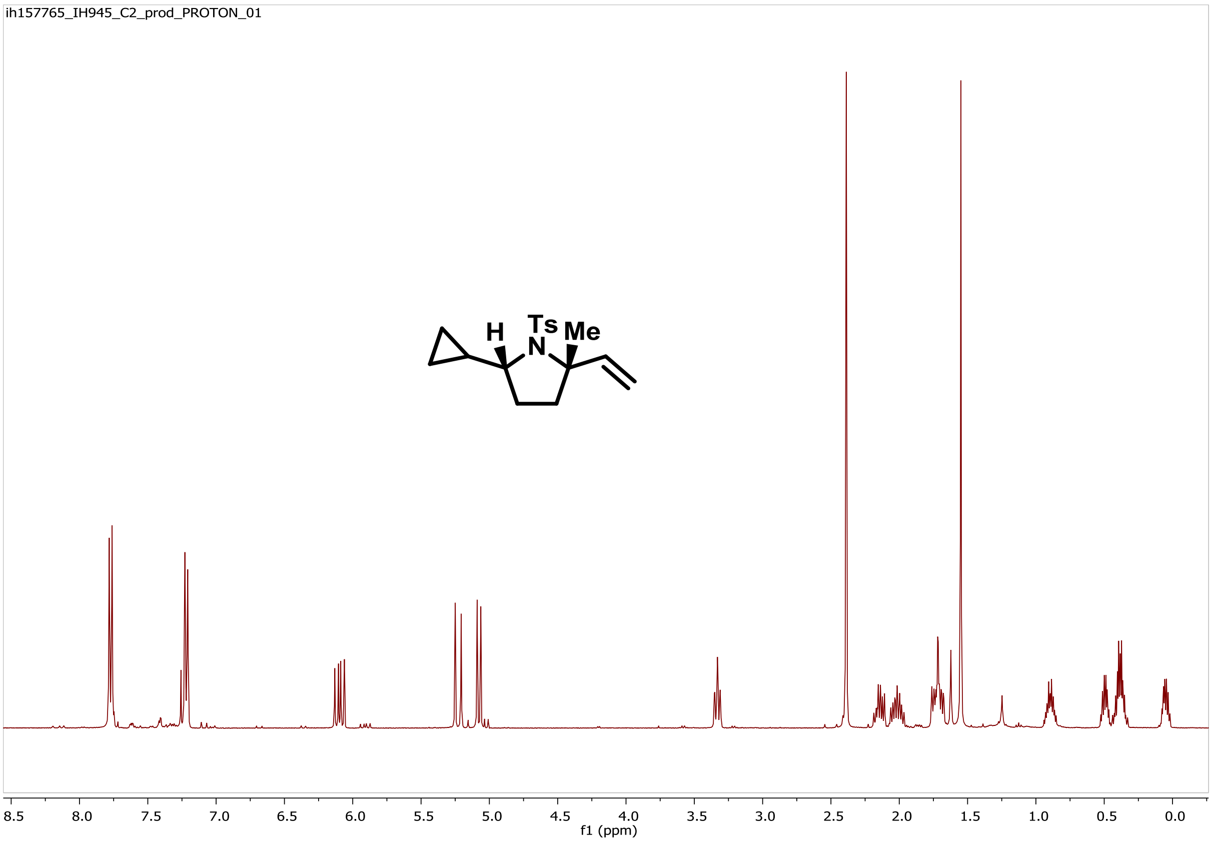
ih157765_IH945_C2_prod_PROTON_01
H
Ts
N
Me
8.5 8.0 7.5 7.0 6.5 6.0 5.5 5.0 4.5 4.0 3.5 3.0 2.5 2.0 1.5 1.0 0.5 0.0
f1 (ppm)

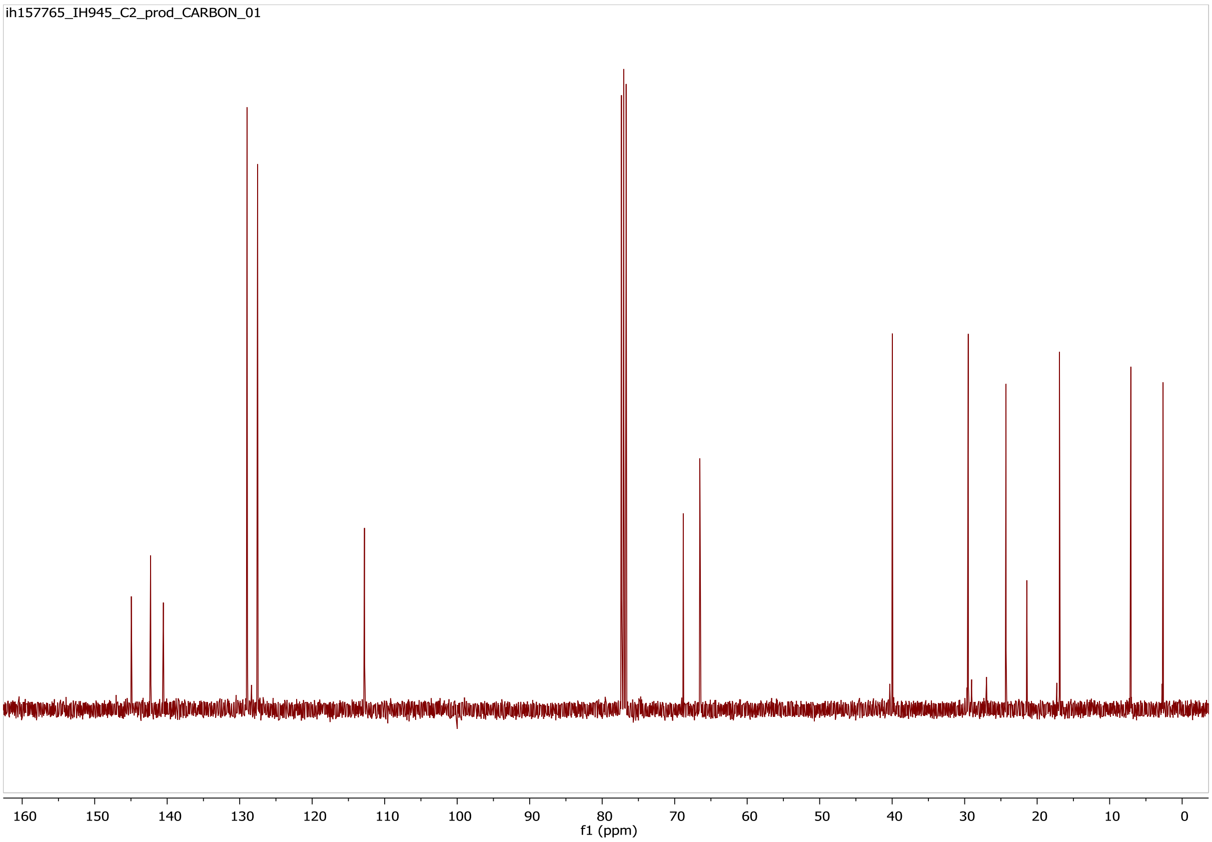
ih157765_IH945_C2_prod_CARBON_01
160 150 140 130 120 110 100 90 80 70 60 50 40 30 20 10 0
f1 (ppm)

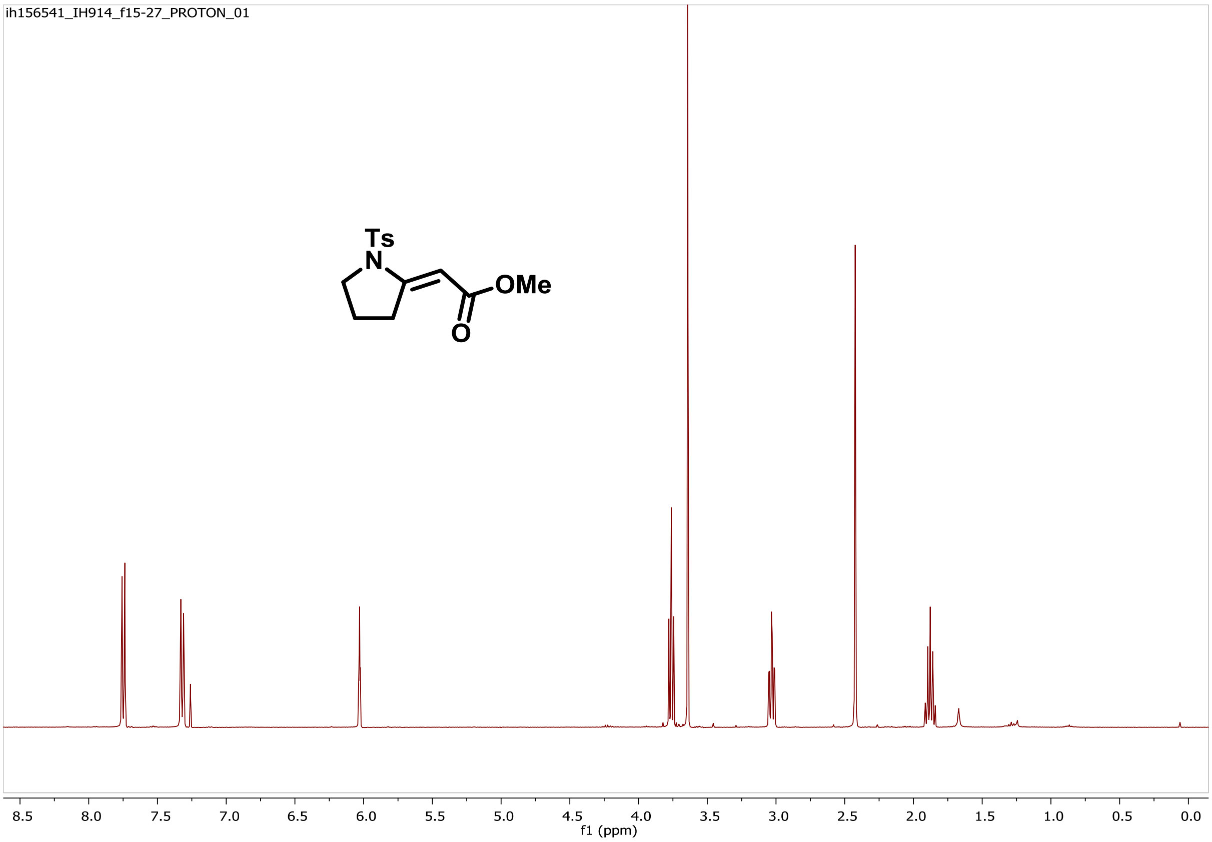
ih156541_IH914_f15-27_PROTON_01
Ts
N
OMe
O
8.5
8.0
7.5
7.0
6.5
6.0
5.5
5.0
4.5
4.0
3.5
3.0
2.5
2.0
1.5
1.0
0.5
0.0
f1 (ppm)

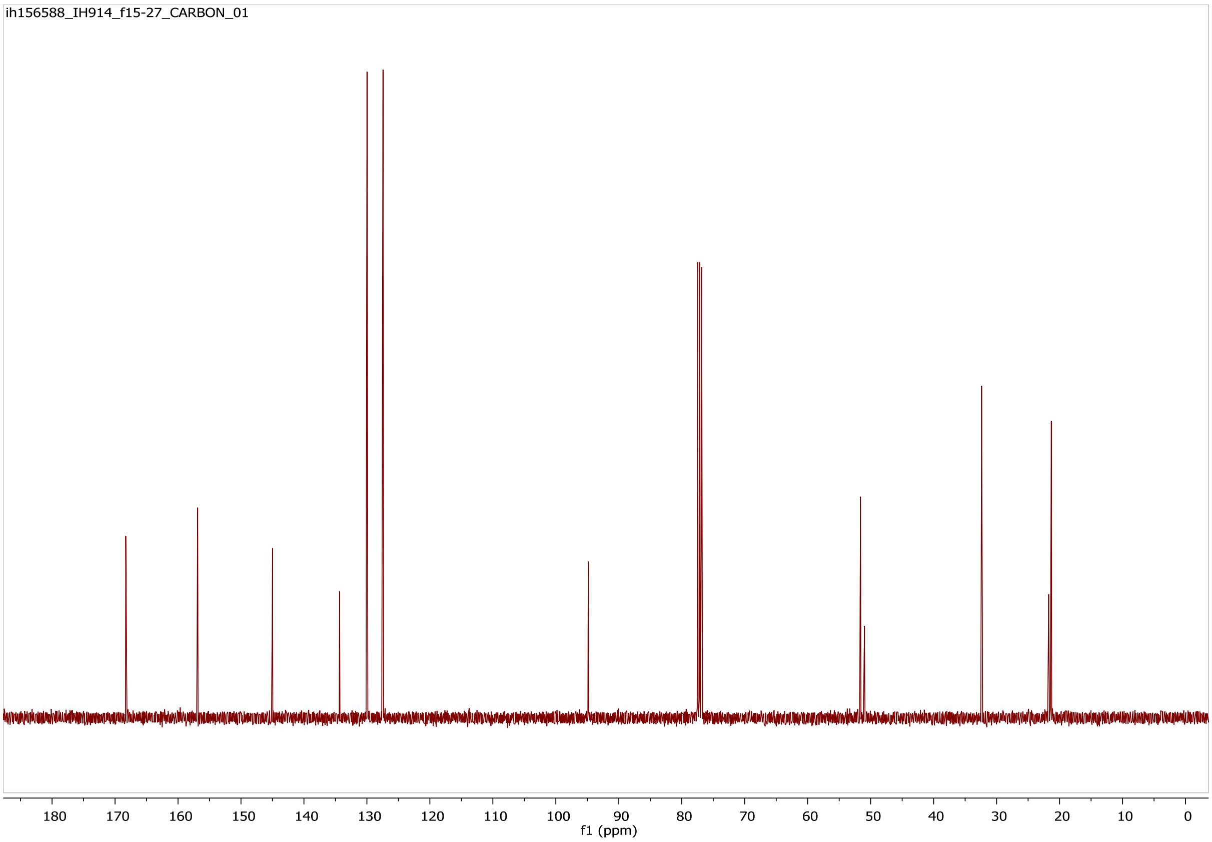
ih156588_IH914_f15-27_CARBON_01
180
170
160
150
140
130
120
110
100
90
80
70
60
50
40
30
20
10
0
f1 (ppm)

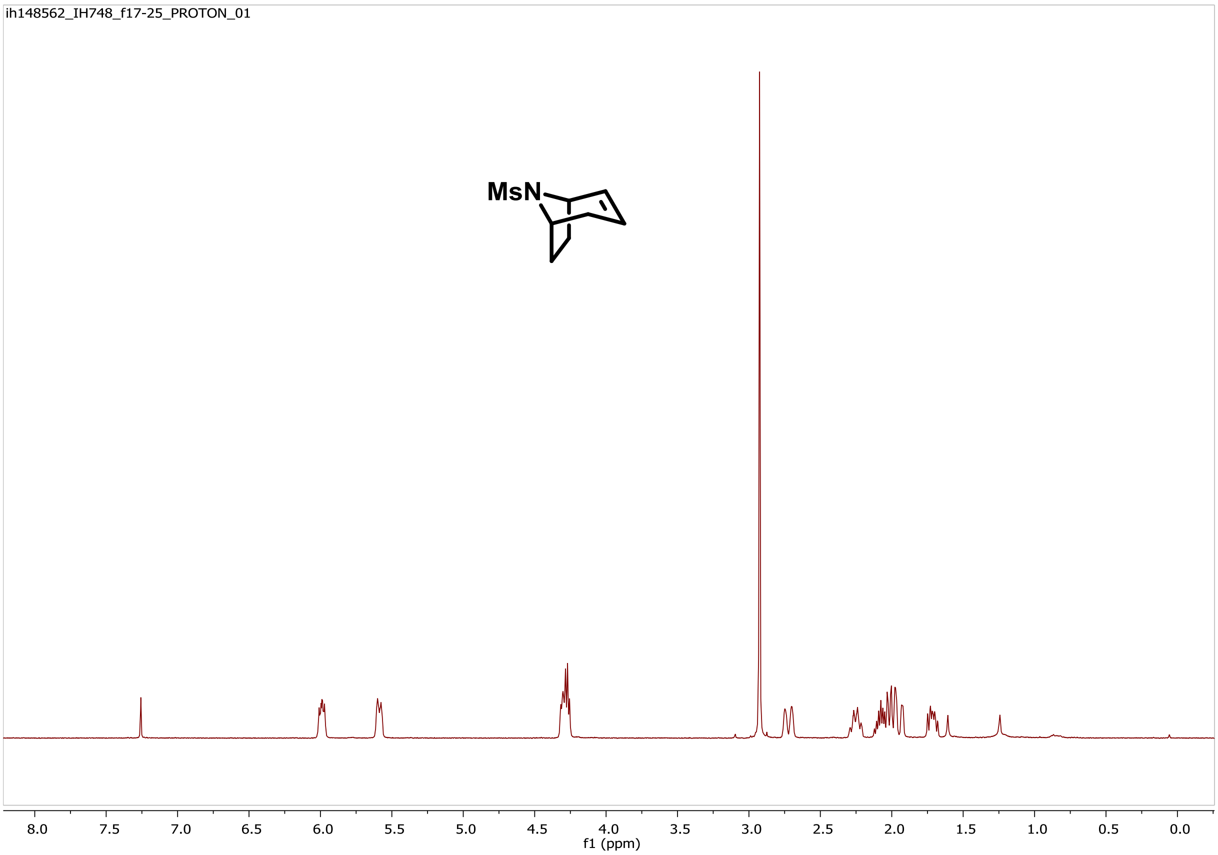
ih148562_IH748_f17-25_PROTON_01
MsN
8.0 7.5 7.0 6.5 6.0 5.5 5.0 4.5 4.0 3.5 3.0 2.5 2.0 1.5 1.0 0.5 0.0
f1 (ppm)

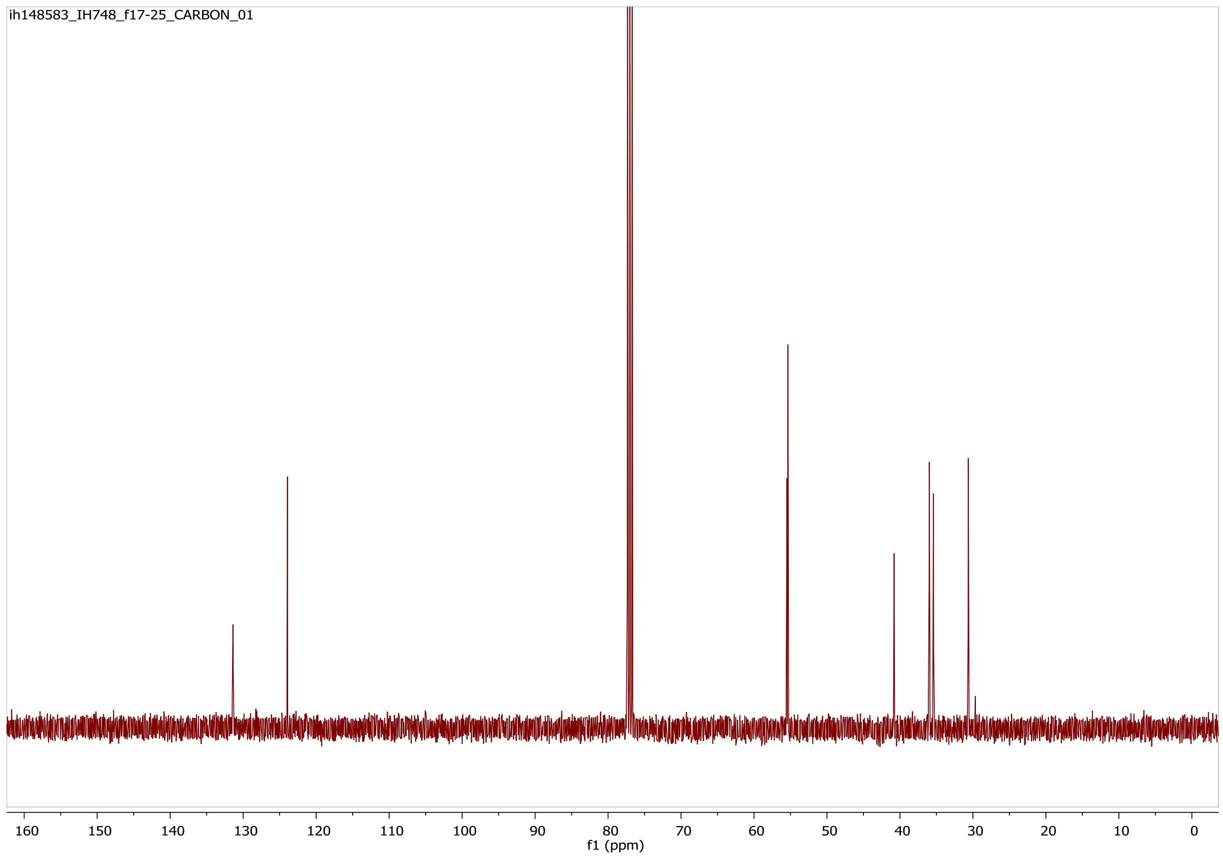
ih148583_IH748_f17-25_CARBON_01
160
150
140
130
120
110
100
90
80
70
60
50
40
30
20
10
0
f1 (ppm)

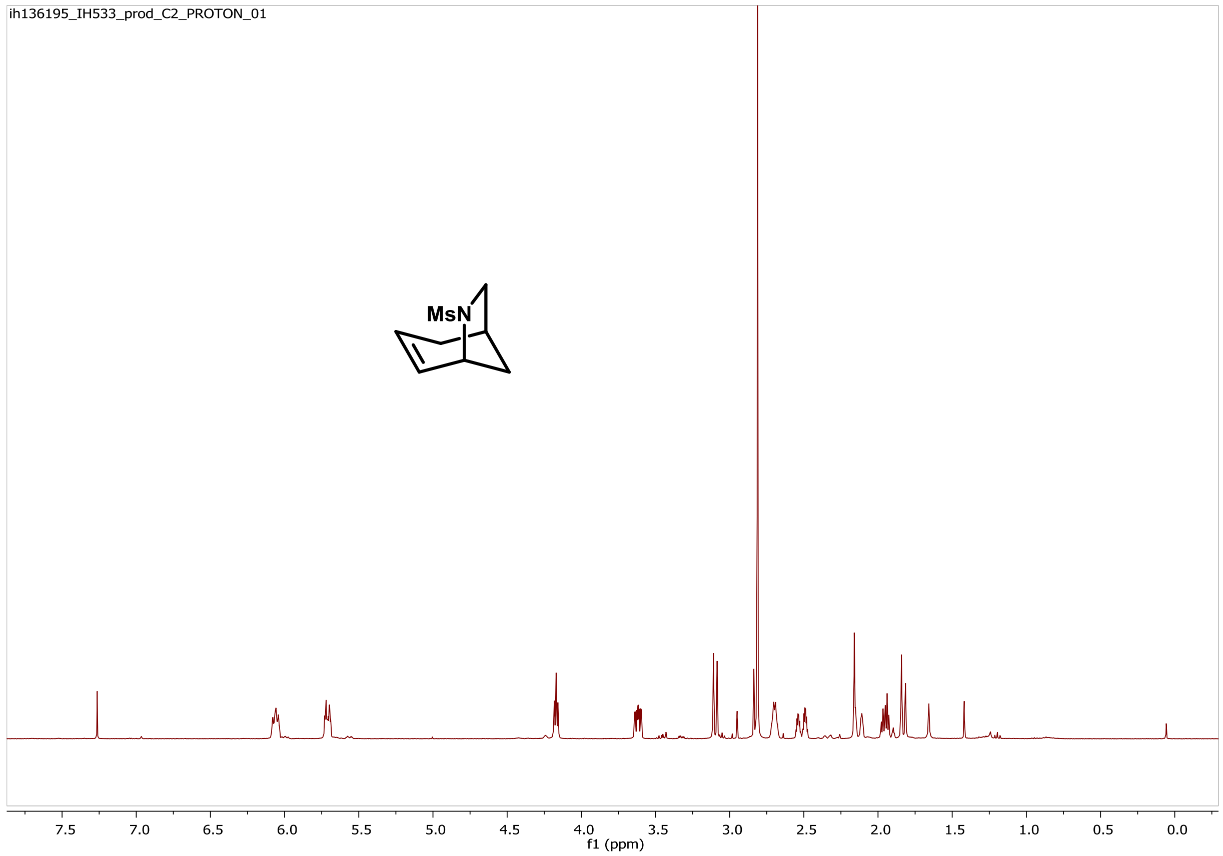
ih136195_IH533_prod_C2_PROTON_01
MsN
7.5
7.0
6.5
6.0
5.5
5.0
4.5
4.0
3.5
3.0
2.5
2.0
1.5
1.0
0.5
0.0
f1 (ppm)

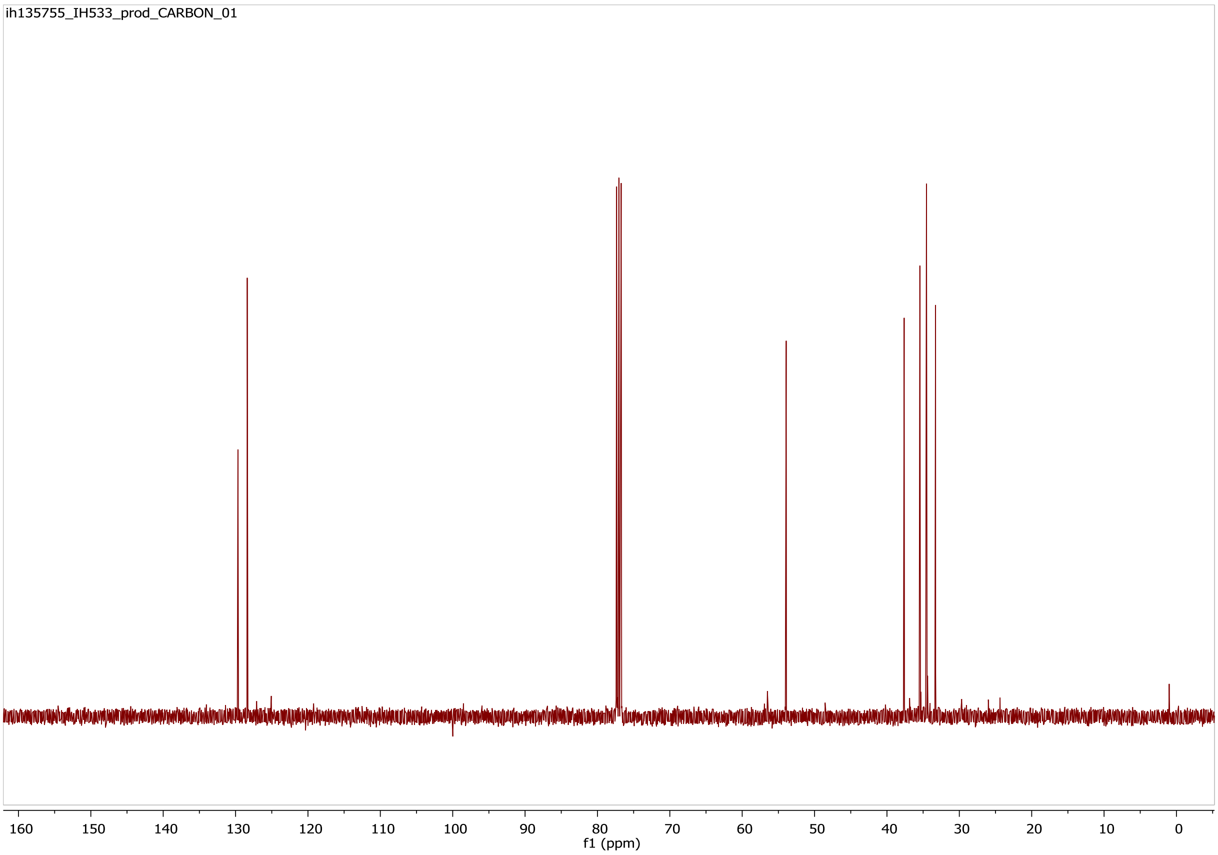
ih135755_IH533_prod_CARBON_01
160 150 140 130 120 110 100 90 80 70 60 50 40 30 20 10 0
f1 (ppm)

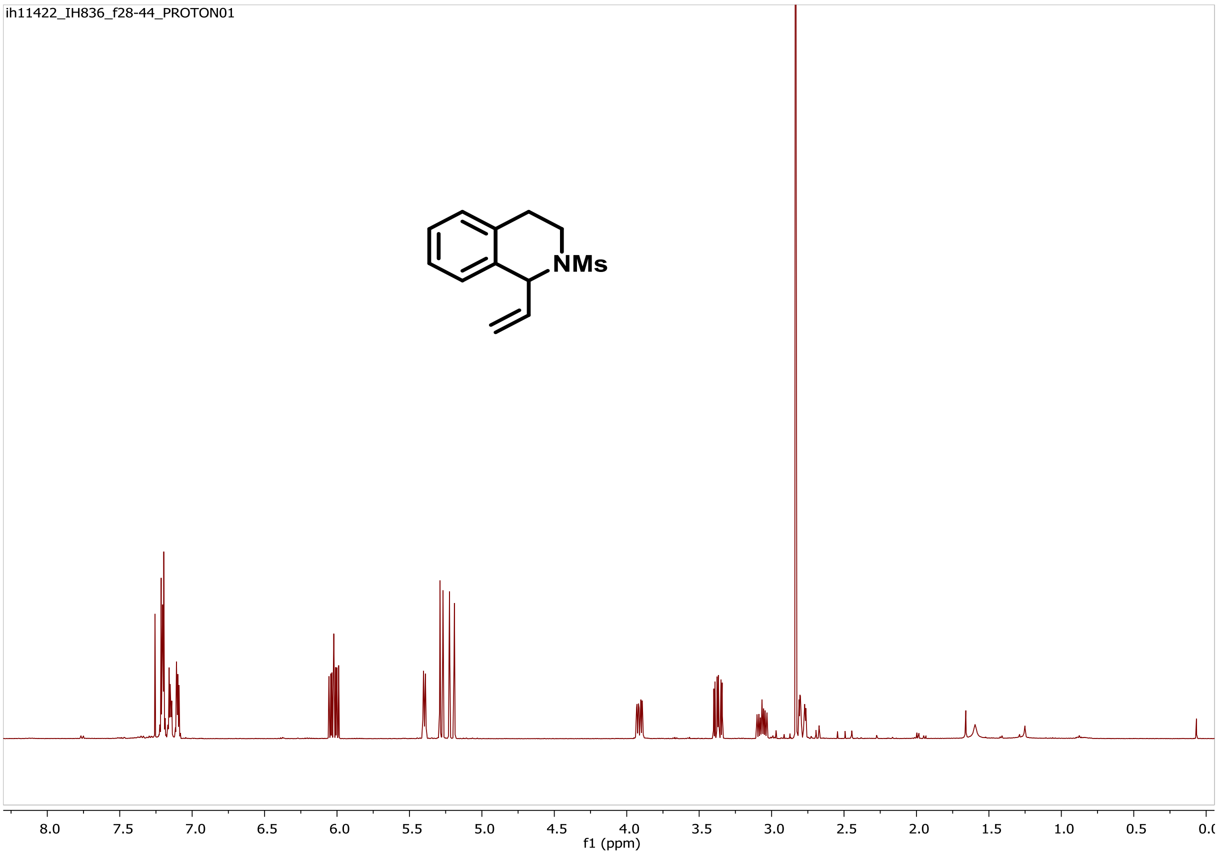
ih11422_IH836_f28-44_PROTON01
NMs
8.0 7.5 7.0 6.5 6.0 5.5 5.0 4.5 4.0 3.5 3.0 2.5 2.0 1.5 1.0 0.5
f1 (ppm)

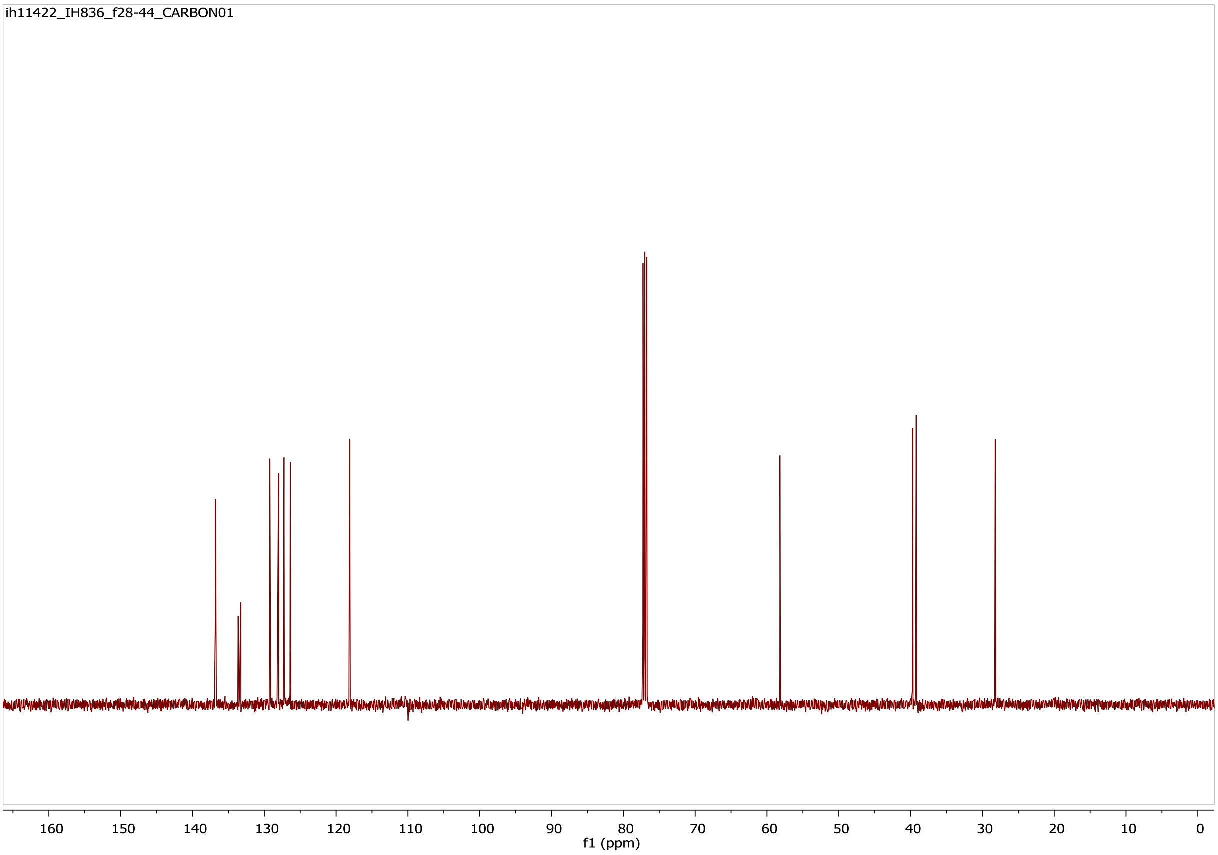
ih11422_IH836_f28-44_CARBON01
160 150 140 130 120 110 100 90 80 70 60 50 40 30 20 10 0
f1 (ppm)

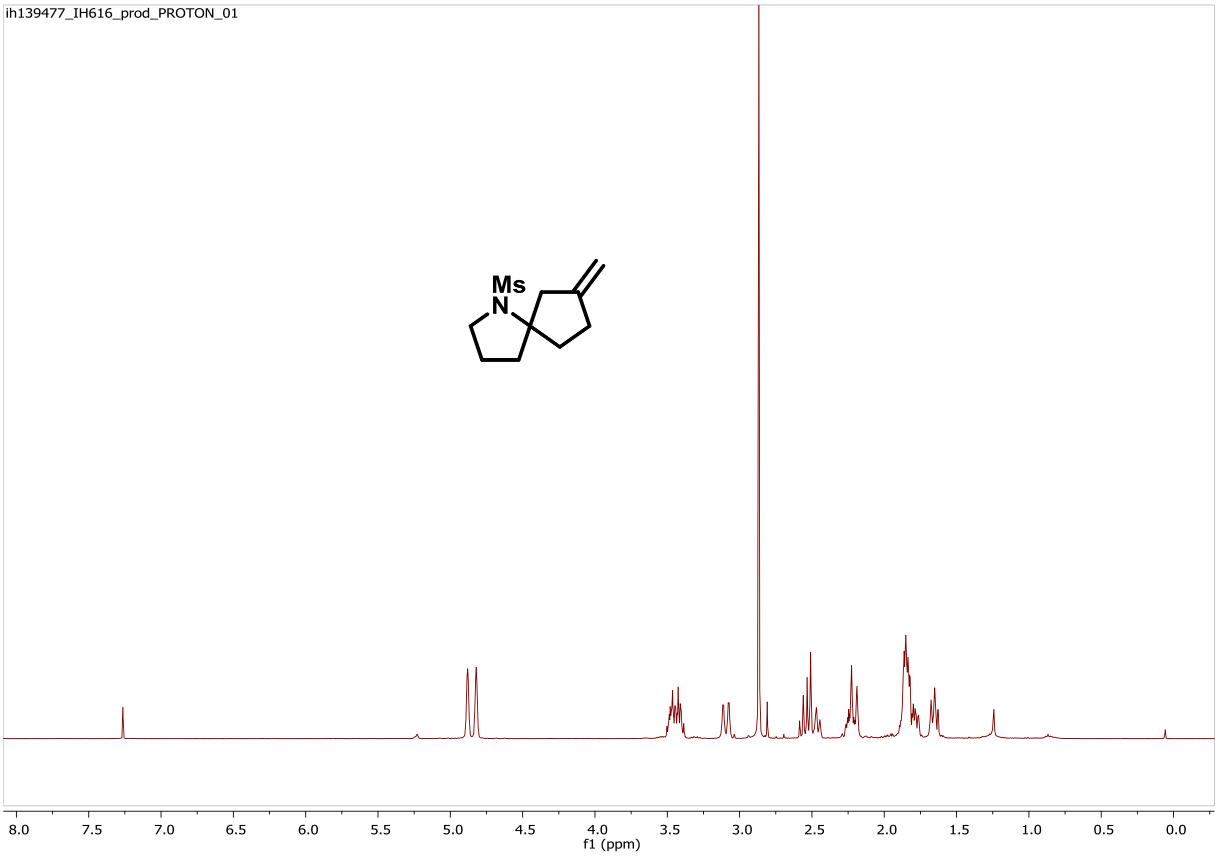
ih139477_IH616_prod_PROTON_01
Ms
N
8.0 7.5 7.0 6.5 6.0 5.5 5.0 4.5 4.0 3.5 3.0 2.5 2.0 1.5 1.0 0.5 0.0
f1 (ppm)

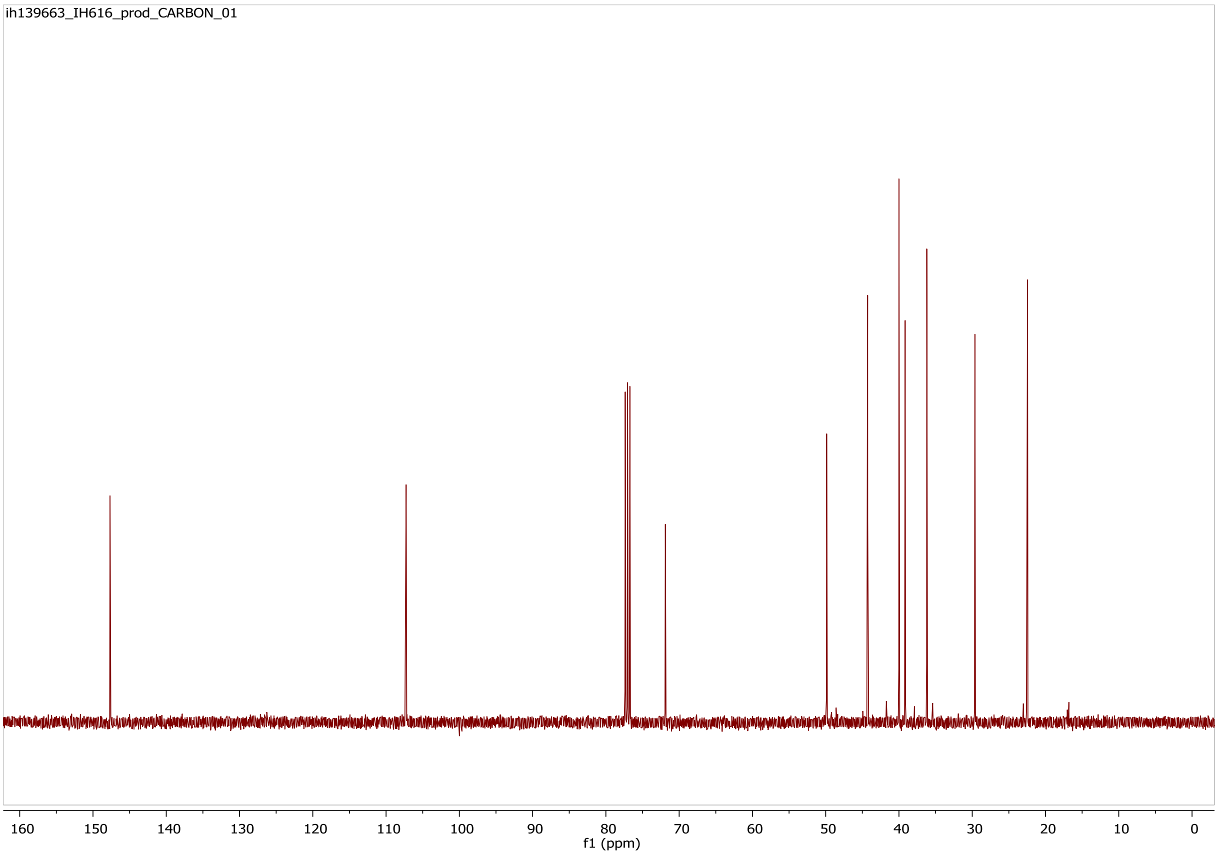
ih139663_IH616_prod_CARBON_01
160 150 140 130 120 110 100 90 80 70 60 50 40 30 20 10 0
f1 (ppm)

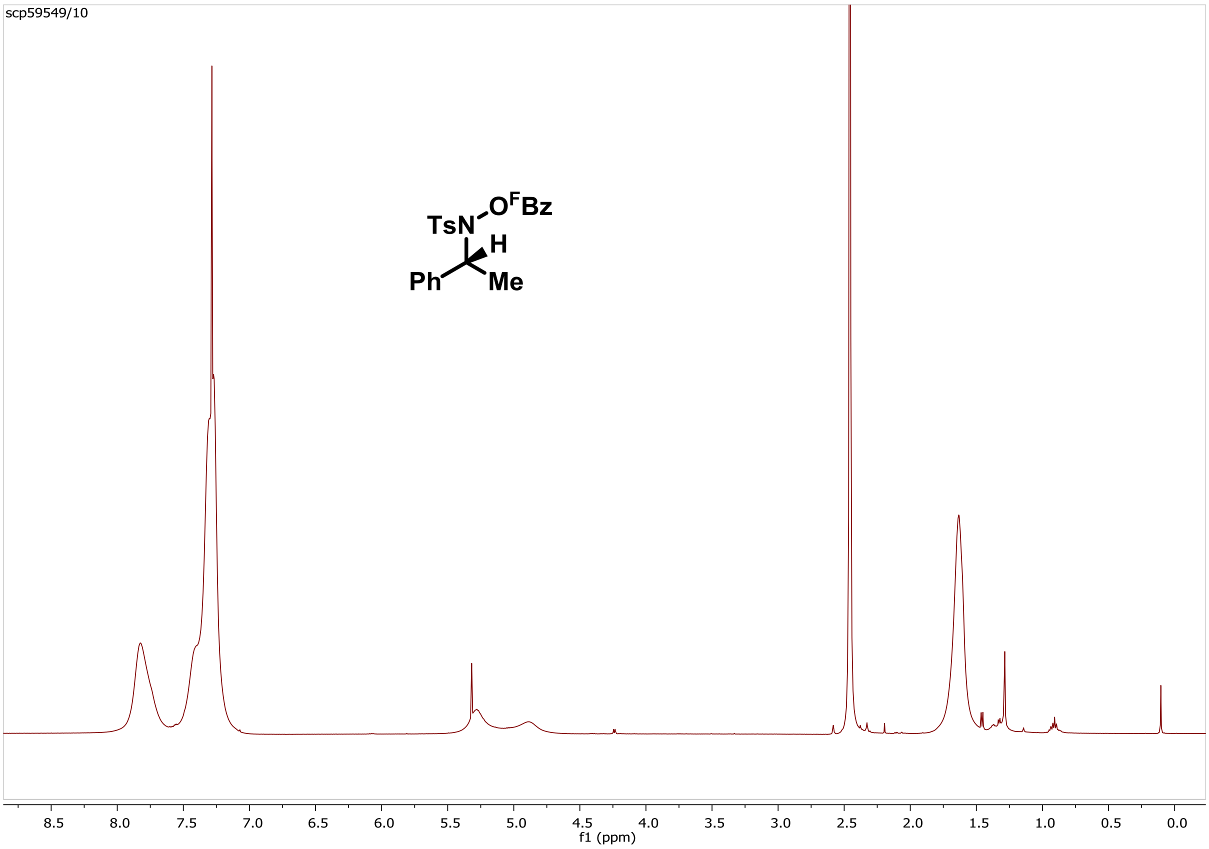
scp59549/10
TsN
O
F
Bz
H
Ph
Me
8.5 8.0 7.5 7.0 6.5 6.0 5.5 5.0 4.5 4.0 3.5 3.0 2.5 2.0 1.5 1.0 0.5 0.0
f1 (ppm)

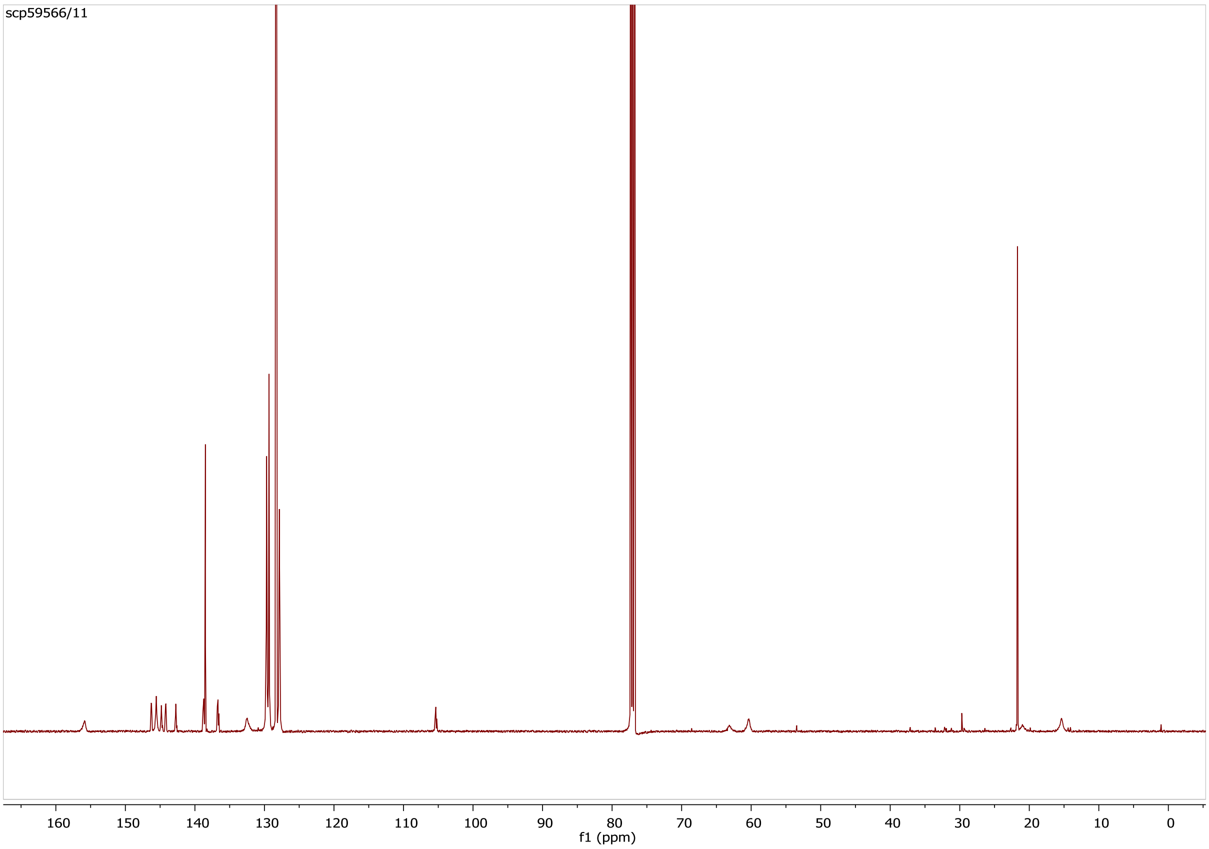
scp59566/11
160
150
140
130
120
110
100
90
80
70
60
50
40
30
20
10
0
f1 (ppm)
